# Prevalence, incidence, risk, and protective factors for soft tissue dehiscences at implant sites in the absence of disease: An AO/AAP systematic review and meta‐regression analysis

**DOI:** 10.1002/JPER.24-0119

**Published:** 2025-06-09

**Authors:** Lorenzo Tavelli, Shayan Barootchi

**Affiliations:** ^1^ Department of Oral Medicine Infection, and Immunity Division of Periodontology Harvard School of Dental Medicine Boston Massachusetts USA; ^2^ School of Dentistry Universidad Catolica de Guayaquil Guayaquil Ecuador; ^3^ Center for Clinical Research and Evidence Synthesis in Oral Tissue Regeneration (CRITERION) Boston USA; ^4^ Department of Periodontics & Oral Medicine University of Michigan School of Dentistry Ann Arbor Michigan USA

**Keywords:** complication, dental implants, epidemiology, gingival recession, risk factor, soft tissue

## Abstract

**Background:**

The aim of the present review was to evaluate the prevalence and incidence of soft tissue dehiscences at implant sites in absence of disease, together with the related risk and protective factors.

**Methods:**

A systematic search was conducted to identify cross‐sectional and prospective studies reporting information on soft tissue dehiscences. Mixed‐effects uni‐ and multi‐level regression analyses were performed to identify predictive factors associated with these conditions.

**Results:**

A total of 221 eligible studies were included. Soft tissue dehiscences (“recessions”) were identified as peri‐implant soft tissue dehiscences (PSTDs), Mucosal level (ML) apical shifts, and mucosal recessions (MRECs). The mean prevalence of PSTD and MREC was 46.2% and 23.1%, respectively. The incidence of PSTD, MREC, and apical shift of ML within 5 years following loading was up to 38.3%, 47.8%, and 23.6%, respectively. Limited mucosal thickness (MT), immediate implant therapy, and lack of peri‐implant soft tissue augmentation were risk factors for PSTD, while limited MT, lack of/limited keratinized mucosa (KM) width, and immediate implant therapy were risk factors for ML apical shift. Guided implant surgery, bone grafting at implant placement, soft tissue augmentation, and adequate KM and MT were protective factors for the stability of ML. Lack of/limited KM width and interproximal marginal bone loss were risk factors for MREC.

**Conclusions:**

Soft tissue dehiscences are commonly observed at implant sites. Risk and protective factors associated with PSTD, ML apical shift, and MREC were identified. A new diagnostic system and format for assessing and reporting soft tissue dehiscence at implant sites was proposed.

**Plain Language Summary:**

The aim of the present review was to evaluate the prevalence and incidence of soft tissue recession (“gum loss”) at healthy implant sites. A systematic search was conducted to identify studies reporting information on soft tissue recession at implant sites. The statistical analysis also explored correlations of different factors with soft tissue recession. A total of 221 studies were included. Soft tissue dehiscences (“recession”) were identified as peri‐implant soft tissue dehiscences (PSTDs), Mucosal Level (ML) apical shift, and mucosal recessions (MRECs). The prevalence of soft tissue recession was, on average, 46.2%. The factors associated with this condition were thin soft tissue, lack of a band of keratinized tissue, and lack of a soft tissue graft surgery at the time of implant placement. On the other hand, placing dental implants using a surgical guide, performing bone grafting and soft tissue grafting at the time of implant placement, and having adequate thickness and keratinization of the soft tissue were protective factors reducing the risk of recession.

## 1 INTRODUCTION

Thousands of implants are placed every day in the United States to facilitate oral rehabilitations.^1^, ^2^ While there is no doubt that dental implants have had a tremendous positive impact on the life of many patients, implant‐related complications also occur.^3^, ^4^, ^5^ In particular, soft tissue dehiscences have become an increasing concern due to the patients’ high esthetic expectations.^6^, ^7^, ^8^

Soft tissue dehiscences at implant sites have been initially compared and considered the counterpart of gingival recessions in the natural dentition, with some authors speculating that these conditions may share several etiological factors.^9^ According to Roccuzzo et al., unlike teeth where shallow recessions are not always associated with esthetic concerns, even a minimal soft tissue dehiscences exposing the titanium surface of the implant/abutment may jeopardize a patient's perception of implant therapy.^6^

Early studies defined soft tissue dehiscence merely based on the presence of an exposure of the abutment or implant fixture at the buccal aspect.^10^, ^11^, ^12^ Others challenged this definition, claiming that implants with long crowns should still be considered as having a peri‐implant soft tissue dehiscence (PSTD), that is, an esthetic complication, regardless of whether the exposure included either abutment and/or the implant fixture.^7^, ^8^, ^13^ Consistent with the concept that dental implants mimic the appearance of the natural dentition, these authors recommended that a diagnosis of a PSTD should be given to any implant where the height of its soft tissue margin is more apical than the cemento‐enamel junction (CEJ) of the homologous contralateral tooth.^7^, ^8^, ^13^, ^14^, ^15^ Similarly, several esthetic score systems involve the evaluation of the level of the soft tissue margin compared to the contralateral homologous tooth.^16^, ^17^, ^18^ Recent classifications of PSTD at implant sites also highlight the importance of the interproximal soft tissue and the bucco‐lingual position of the crown/implant shoulder.^13^, ^19^, ^20^ In this scenario, with different definition and classification systems being utilized to diagnose soft tissue dehiscence (“recession”), the prevalence, incidence and factors associated with this condition have not been fully elucidated.

Therefore, in alignment with the purpose of the American Academy of Periodontology (AAP) and Academy of Osseointegration (AO) Best Evidence Consensus (BEC) on prevention and management of peri‐implant diseases and conditions, the present systematic review aimed at evaluating the prevalence and incidence of soft tissue dehiscences (“recessions”) at implant sites in the absence of disease, together with the risk and protective factors associated with these conditions.

## 2 MATERIALS AND METHODS

### 2.1 Protocol registration and reporting format

The protocol for the present review was designed according to the Cochrane guidelines^21^ and reported with the Preferred Reporting Items for Systematic reviews and Meta‐Analysis Extension (PRISMA)^22^ – 2020 statement for systematic reviews incorporating network meta‐analyses for health care interventions.^23^, ^24^ The study protocol was registered and allocated in the PROSPERO database (identification number CRD42024497637).

### 2.2 PECOT question

**FIGURE 1:**
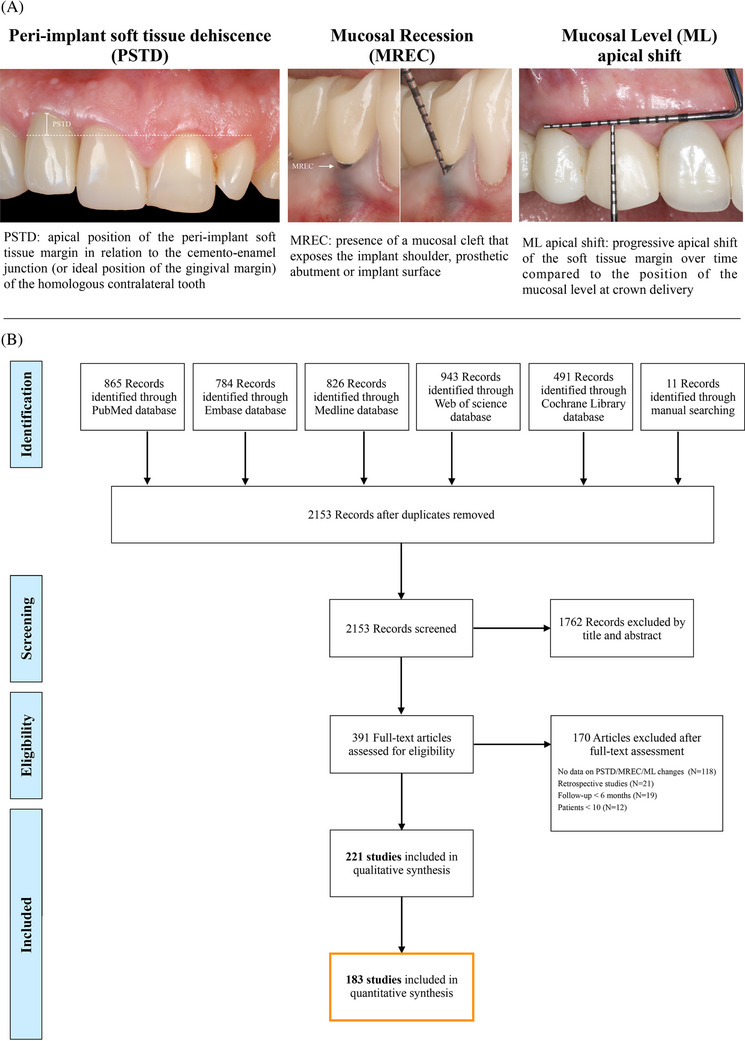
(A) Definitions of soft tissue dehiscence in terms of peri‐implant soft tissue dehiscence (PSTD), mucosal recession (MREC), and mucosal level (ML) apical shift. (B) Preferred Reporting Items for Systematic reviews and Meta‐Analysis Extension (PRISMA) flowchart.

The following Population, Exposure, Comparison, Outcomes, and Time framework (PECOT) ^25^ was used to guide the inclusion and exclusion of studies for the above‐mentioned focused questions:

- **Population (P)**: Patients ≥ 18 years old presenting with at least 1 single‐site dental implant and/or at least 1 single‐site edentulous area and seeking for dental implant rehabilitation.
- **Exposure (E)**: Conventional, immediate, or early implant placement, with or without bone augmentation, with or without soft tissue grafting, with or without immediate provisionalization, with or without immediate loading; and soft tissue augmentation at osseointegrated implant sites.
- **Comparison (C)**: Different timing of implant placement, bone augmentation, soft tissue augmentation, immediate provisionalization, immediate loading, and other pertinent patient‐ and implant‐related factors.
- **Outcomes (O)**: The primary outcome was to assess the prevalence and incidence of soft tissue dehiscence (“recession”) at implant sites in the absence of peri‐implant diseases, defined as follow or as defined in the individual studies:
  - Peri‐implant soft tissue dehiscence (PSTD): apical position of the peri‐implant soft tissue margin in relation to the cemento‐enamel junction (CEJ) of the homologous contralateral tooth.^13^ This definition was recommended for implants in anterior sites.^13^
  - Mucosal recession (MREC): presence of a mucosal cleft that exposes the implant shoulder, prosthetic abutment or implant surface.^26^
  - Apical shift of the mucosal level (ML). Progressive apical shift of the soft tissue margin over time compared to the position of the mucosal level at crown delivery. ML changes are assessed using reference points (i.e., using a stent).^27^, ^28^ Because of this outcome requires observation at multiple time points, ML changes were reported in prospective studies only, while PSTD and MREC were described both in cross‐sectional and prospective studies (Figure 1A).
  Secondary outcomes included the assessment of the risk indicators and risk factors associated with the prevalence, incidence, and severity of these conditions. Prevalence was assessed from the included cross‐sectional studies as the proportion of implant displaying soft tissue dehiscence and was expressed in percentage. Incidence was assessed from the included prospective studies as the proportion of new implant sites displaying soft tissue dehiscence over a specified time period, and was expressed in percentage. Additional information on the data of interest retrieved from the eligible study are listed in the Appendix (see Supplementary Appendix in online *Journal of Periodontology*).
- **Study Design (S)**: Human cross‐sectional and prospective clinical studies, including randomized controlled trials (RCTs), non‐randomized case‐control studies, and case series with at least 10 patients in an arm. For prospective clinical studies, only articles reporting at least 2 time points, with the last time points being at least 6 months from implant functional loading were considered. Retrospective studies/analysis, case reports, or case series with less than 10 treated individuals were not included due to the scarcity of information.
- **Time (T)**: Minimum follow‐up of 6 months from implant functional loading

Additional information regarding eligible studies, information sources and search strategy, article selection process and data extraction, methodological quality, and risk of bias assessment are reported in the Appendix (see Supplementary Appendix in online *Journal of Periodontology*).

### 2.3 Synthesis of quantitative results

The retrieved data were analyzed first descriptively and reported as frequencies and percentages for categorical variables and as means (±) standard deviation for continuous outcomes. A series of mixed‐effects uni‐ and multilevel regression analyses were performed to identify predictive factors and explore associations with the outcomes of MREC (in mm), reflecting the mean mucosal recessions depth across study arms; ML changes (in mm), as the change in the levels of peri‐implant buccal mucosal levels over time (which convey stability or its lack thereof), and the incidence (in percentage), and depth (in mm) of PSTD. Study arms were weighted by the treated and analyzed sample size (i.e., the number of treated implants) and clustered by publication. For studies that utilized the same patient population (i.e., different follow‐up reports of the same original research), only 1 report with the most informative and complete data was utilized in the analyses. Relevant baseline demographics, clinical characteristics of the implant therapy (i.e., implant type, implant surgery, guided vs. free‐hand implant therapy, bone augmentation, soft tissue augmentation, etc.) and patients characteristics were all accounted for in all models by inclusion of fixed covariates, and their influence on each outcome assessed. Random effects were also included to capture unique intercepts for study, study arms, as well as random slopes for study by time, and study arm by time. The robustness of the models was tested through sensitivity analyses to observe for any meaningful changes in the estimates of the outcomes, as well as testing model assumptions. Confidence intervals (CIs) were produced, and a *p*‐value threshold of below 0.05 was set for statistical significance. The statistical analyses were performed by an author with experience in linear mixed models (S.B.), using a specified software* and the statistical packages lme4,^29^ lmerTest,^30^ dplyr,^31^ and tidyr.^32^

## 3 RESULTS

### 3.1 Search results and study selection

The literature search flow diagram is shown in Figure 1B. Based on our predetermined inclusion criteria, 221 studies ^6^, ^10^, ^11^, ^14^, ^15^, ^27^, ^28^, ^33^, ^34^, ^35^, ^36^, ^37^, ^38^, ^39^, ^40^, ^41^, ^42^, ^43^, ^44^, ^45^, ^46^, ^47^, ^48^, ^49^, ^50^, ^51^, ^52^, ^53^, ^54^, ^55^, ^56^, ^57^, ^58^, ^59^, ^60^, ^61^, ^62^, ^63^, ^64^, ^65^, ^66^, ^67^, ^68^, ^69^, ^70^, ^71^, ^72^, ^73^, ^74^, ^75^, ^76^, ^77^, ^78^, ^79^, ^80^, ^81^, ^82^, ^83^, ^84^, ^85^, ^86^, ^87^, ^88^, ^89^, ^90^, ^91^, ^92^, ^93^, ^94^, ^95^, ^96^, ^97^, ^98^, ^99^, ^100^, ^101^, ^102^, ^103^, ^104^, ^105^, ^106^, ^107^, ^108^, ^109^, ^110^, ^111^, ^112^, ^113^, ^114^, ^115^, ^116^, ^117^, ^118^, ^119^, ^120^, ^121^, ^122^, ^123^, ^124^, ^125^, ^126^, ^127^, ^128^, ^129^, ^130^, ^131^, ^132^, ^133^, ^134^, ^135^, ^136^, ^137^, ^138^, ^139^, ^140^, ^141^, ^142^, ^143^, ^144^, ^145^, ^146^, ^147^, ^148^, ^149^, ^150^, ^151^, ^152^, ^153^, ^154^, ^155^, ^156^, ^157^, ^158^, ^159^, ^160^, ^161^, ^162^, ^163^, ^164^, ^165^, ^166^, ^167^, ^168^, ^169^, ^170^, ^171^, ^172^, ^173^, ^174^, ^175^, ^176^, ^177^, ^178^, ^179^, ^180^, ^181^, ^182^, ^183^, ^184^, ^185^, ^186^, ^187^, ^188^, ^189^, ^190^, ^191^, ^192^, ^193^, ^194^, ^195^, ^196^, ^197^, ^198^, ^199^, ^200^, ^201^, ^202^, ^203^, ^204^, ^205^, ^206^, ^207^, ^208^, ^209^, ^210^, ^211^, ^212^, ^213^, ^214^, ^215^, ^216^, ^217^, ^218^, ^219^, ^220^, ^221^, ^222^, ^223^, ^224^, ^225^, ^226^, ^227^, ^228^, ^229^, ^230^, ^231^, ^232^, ^233^, ^234^, ^235^, ^236^, ^237^, ^238^, ^239^, ^240^, ^241^, ^242^, ^243^, ^244^, ^245^, ^246^ were included in the present review. The reason for the exclusion of the other 170 articles is reported in the Appendix (see Tables S1 and S2 in online *Journal of Periodontology*). The inter‐examiner reliability in the screening and inclusion process, as assessed with Cohen's κ, corresponded to 0.91 for the initial screening by title and abstract and 0.93 for the full text evaluation.

### 3.2 Characteristics of the included studies

Characteristics of the included studies are reported in detail in Tables S3–S13 (see Tables S3–S13 in online *Journal of Periodontology*). Out of the 221 included studies, 91 were RCTs,† 104 prospective non‐RCTs,‡ and 26 cross‐sectional studies.§ Soft tissue dehiscences (“recessions”) were described in terms of PSTD, MREC, and/or apical shift of the ML in the included studies. In total, 221 studies contributed to the qualitative analysis, while 183 studies¶ provided the data for the quantitative analysis using uni‐ and multilevel regression models aimed at assessing risk factors for soft tissue dehiscences.

### 3.3 Risk of bias assessment

The assessment risk of bias is reported in detail in the Appendix (see Tables S14–S16 in online *Journal of Periodontology*). Overall, 120 studies were rated as low risk of bias, while 84 and 17 studies were considered at unclear and high risk of bias, respectively.

### 3.4 Prevalence and risk indicators of PSTD and MREC

Based on data from cross‐sectional studies only, the mean prevalence of PSTD and MREC was 46.2% and 23.1%, respectively (Table 1 and Figure 2A). Parameters that were found to be significantly correlated with a higher prevalence of PSTD (“risk indicators*”*) among the included cross‐sectional studies were: the position of the implant (buccal position of the implant shoulder,^82^, ^243^ angulation of the implant,^172^ and implant depth^172^), soft tissue related parameters (absence of/limited keratinized mucosa [KM] width,^220^ limited mucosal thickness [MT],^220^ and thin soft tissue phenotype^172^), bone‐related parameters (pre‐implant bone augmentation procedures,^243^ buccal bone dehiscence,^172^, ^220^ and interproximal bone levels^172^), years in function,^220^ and missing adjacent teeth ^220^ (Table 2). Correlations between buccal bone thickness and PSTD were not described.^220^ One article found that PSTD was significantly correlated with the papilla esthetic index and the subjective esthetic index.^130^ On the other hand, the following risk indicators were found for MREC: (i) implants buccally positioned,^195^, ^201^ (ii) absence of /limited KM width,^34^, ^147^, ^161^, ^175^, ^190^, ^201^, ^226^ (iii) thin soft tissue phenotype,^195^, ^226^ (iv) buccal bone dehiscence,^201^ (v) lack of prosthetic abutment,^195^ and (vi) lack of adjacent teeth.^195^ One article also reported that implants with MREC were significantly correlated with bleeding on probing^171^ (Table 2). Table S10 depicts in detail the above‐mentioned correlations, along with the respective odds ratio (OR), 95% CI, and *p*‐value (see Table S10 in online *Journal of Periodontology*).

**TABLE 1:** Prevalence, incidence, and average depth of peri‐implant soft tissue dehiscence (PSTD), mucosal recession (MREC), and mucosal level (ML) changes

Outcome	Time	PSTD (mean ± SD)	MREC (mean ± SD)	ML apical shift (mean ± SD)
Prevalence (%)	/	46.2 ± 10.2^a^	23.1 ± 10.6^h^	/
Incidence (%)	*T*_1_	33.3 ± 9.4^b^	N/A	15.3 ± 26.7^o^
	*T*_1_–*T*_3_	37.5 ± 19.1^c^	47.8 ± 26.0^i^	23.3 ± 16.2^p^
	*T*_3_–*T*_4_	38.3 ± 11.0^d^	45.7 ± 19.6^j^	23.6 ± 9.7^q^
	>*T*_5_	64.5 ± 26.6^e^	N/A	35.1 ± 28.1^r^
Depth (mm)	*T*_1_	0.33 ± 0.30^f^	0.39 ± 0.36^k^	0.37 ± 0.32^s^
	*T*_1_–*T*_3_	0.43 ± 0.31^g^	0.52 ± 0.36^l^	0.41 ± 0.37^t^
	*T*_3_–*T*_5_	N/A	0.71 ± 0.52^m^	0.63 ± 0.39^u^
	>*T*_5_	N/A	0.75 ± 0.71^n^	0.65 ± 0.28^v^

Abbreviations: NA, not available; SD, standard deviation; *T*_1_, within the first year after loading; *T*_3_, within 3 years from loading; T_5_, within 5 years from loading; >*T*_5_: any time point after 5 years from loading; Note that incidence at *T*_1_, *T*_1_–*T*_3_, *T*_3_–*T*_5_, and >*T*_5_ was provided in comparison to baseline (crown delivery).

^a^ Based on 5 studies reporting on 378 patients with 396 implants.

^b^ Based on 7 studies reporting on 297 patients with 297 implants.

^c^ Based on 6 studies reporting on 313 patients with 319 implants.

^d^ Based on 5 studies reporting on 361 patients with 361 implants.

^e^ Based on 2 studies reporting on 52 patients with 76 implants.

^f^ Based on 9 studies reporting on 267 patients with 278 implants.

^g^ Based on 3 studies reporting on 125 patients with 131 implants.

^h^ Based on 4 studies reporting on 323 patients with 1012 implants.

^i^ Based on 5 studies reporting on 348 patients with 580 implants.

^j^ Based on 6 studies reporting on 494 patients with 761 implants.

^k^ Based on 20 studies reporting on 856 patients with 1437 implants.

^l^ Based on 16 studies reporting 1363 patients with 2762 implants.

^m^ Based on 16 studies reporting 1367 patients with 2353 implants.

^n^ Based on 11 studies reporting 453 patients with 875 implants.

^o^ Based on 18 studies reporting on 657 patients with 798 implants.

^p^ Based on 10 studies reporting on 541 patients with 658 implants.

^q^ Based on 6 studies reporting on 309 patients with 317 implants.

^r^ Based on 4 studies reporting on 186 patients with 210 implants.

^s^ Based on 84 studies reporting on 3967 patients with 4175 implants.

^t^ Based on 25 studies reporting on 1516 patients with 1594 implants.

^u^ Based on 14 studies reporting on 686 patients with 733 implants.

^v^ Based on 5 studies reporting on 170 patients with 170 implants.

FIGURE 2(A) Prevalence of peri‐implant soft tissue dehiscence (PSTD) and mucosal recession (MREC), and weighted MREC depth from the included cross‐sectional studies. (B) Risk and protective factors for PSTD, mucosal level (ML) changes, and MREC identified with the multilevel regression analyses.

**Figure:**
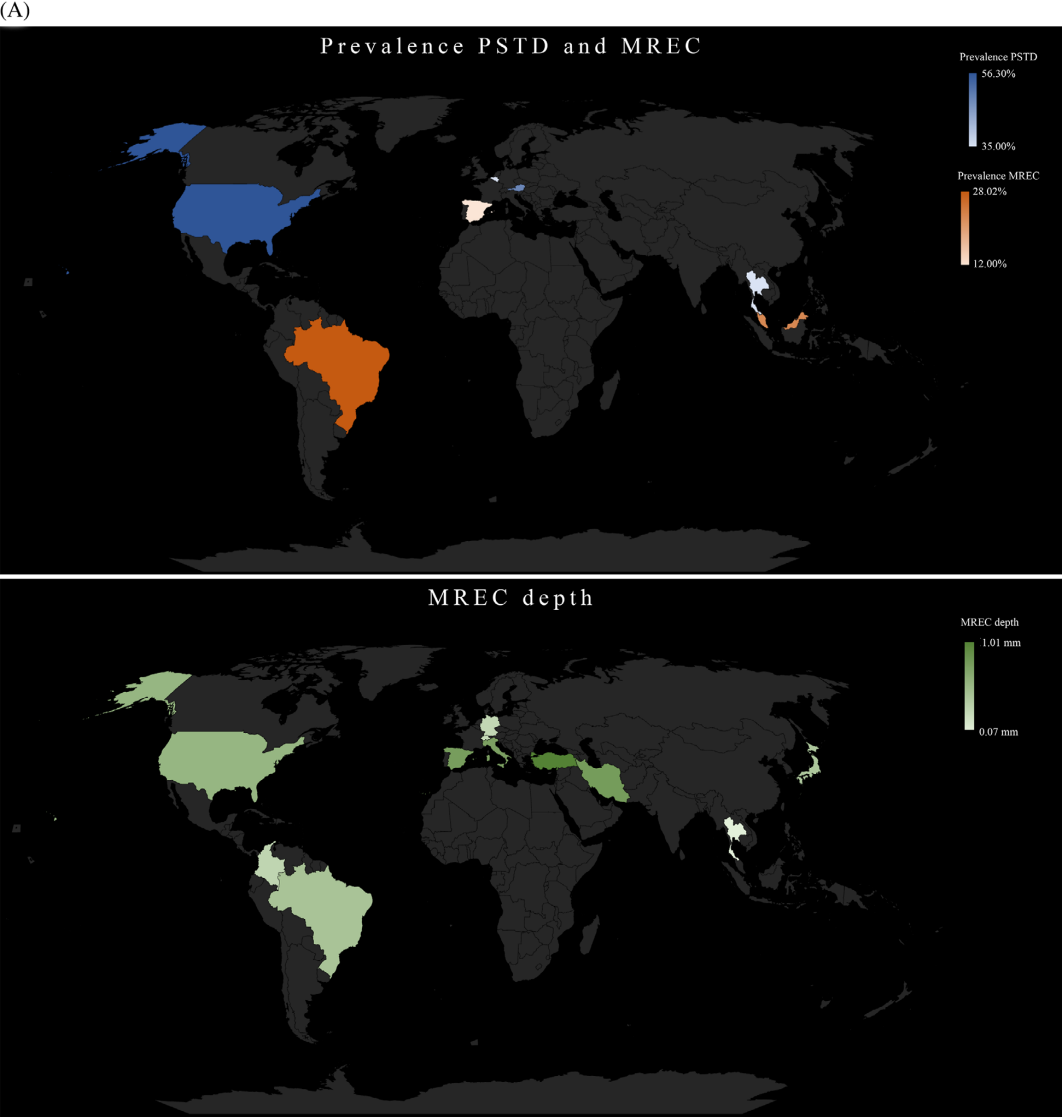

**Figure:**
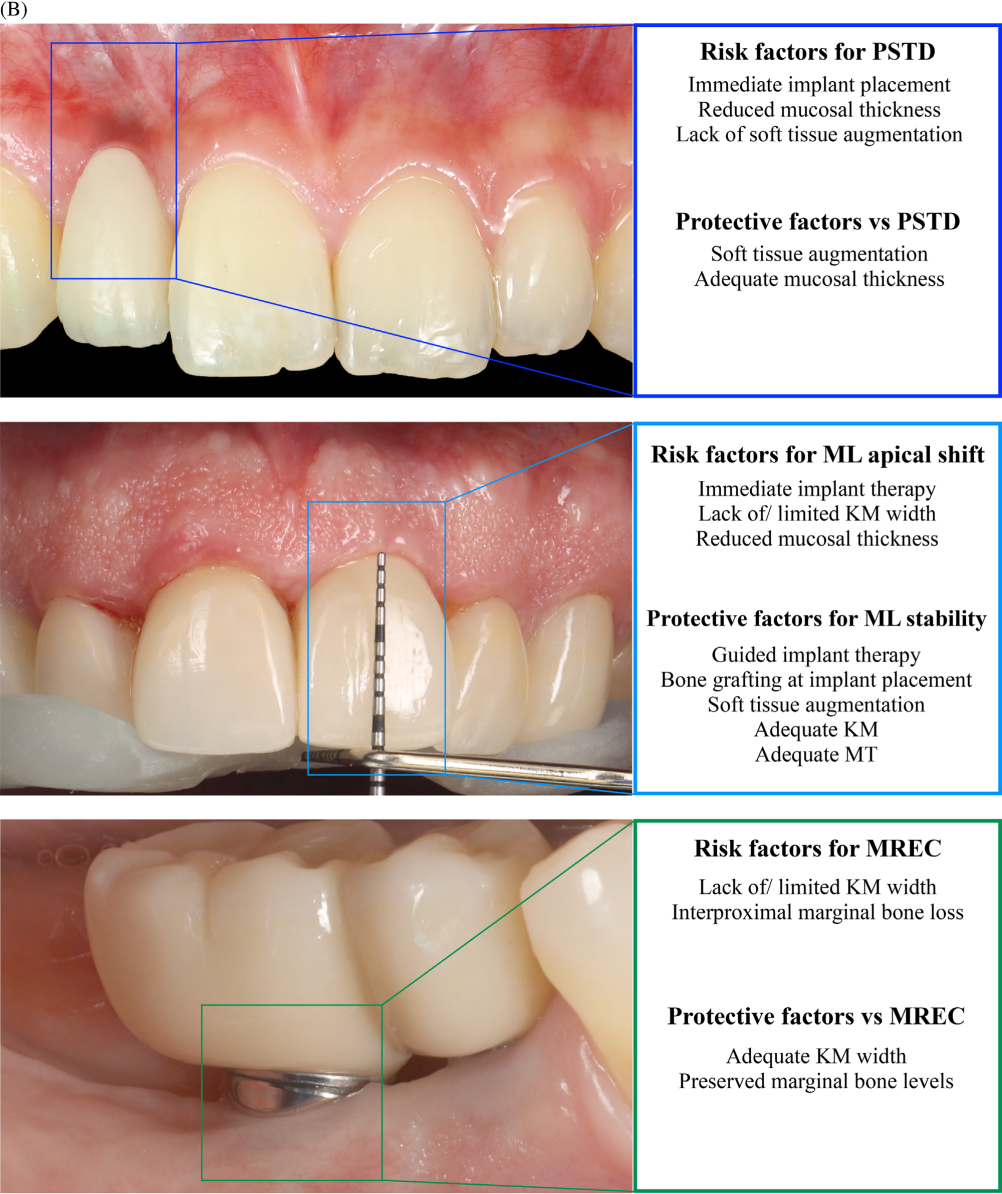

**TABLE 2:** Putative risk indicators and risk factor for peri‐implant soft tissue dehiscence (PSTD), mucosal level (ML) apical shift, and mucosal recession (MREC) reported in the included studies

Parameter	PSTD	MREC	ML apical shift
**Risk indicators**	Implant malposition (implant buccally positioned, implant proclined, an implant too deep)^82^, ^172^, ^243^ Soft tissue‐related parameters (absence of/limited KM width, limited MT, and thin soft tissue phenotype)^172^, ^220^ Bone‐related parameters (buccal bone dehiscence, and interproximal bone levels)^172^, ^220^ Year in function, and presence of adjacent implant(s)^220^	Implant buccally positioned^195^, ^201^ Soft tissue‐related parameters (absence of/limited KM width, limited MT, and thin soft tissue phenotype)^34^, ^147^, ^159^, ^161^, ^175^, ^190^, ^195^, ^201^, ^226^ Buccal bone dehiscence^201^ One‐piece implant, missing adjacent teeth, lack of abutment, and bleeding on probing^171^, ^195^, ^201^	N/A
**Risk factors**	Buccally positioned implant^68^ Soft‐tissue related parameters (non‐soft tissue‐grafted sites, absence of keratinized mucosa, and thin phenotype)^111^, ^129^, ^167^ Interproximal crestal bone levels^160^ Time after implant placement^129^	Soft‐tissue related parameters (lack of KM, and KM width <2 mm)^53^, ^84^, ^205^	Surgical‐related parameters (Non‐grafted sockets, flap approach, buccally positioned implant, High insertion torque, No antibiotics)^28^, ^42^, ^132^, ^162^, ^186^, ^236^ Soft‐tissue related parameters (non‐soft tissue‐grafted sites, limited MT, thin phenotype)^28^, ^141^, ^145^, ^149^, ^235^ Bone‐related parameters (bone morphology, BBD depth and width)^143^, ^149^, ^168^ Convex emergence profile of the crown^28^, ^60^, ^208^ Central incisor position, and mandibular sites^28^, ^162^ Age and smoking habit^137^, ^189^

Abbreviations: BBD, buccal bone dehiscence; KM, keratinized mucosa; ML, mucosal level; MREC, mucosal recession; MT, mucosal thickness; N/A, not applicable; PSTD, peri‐implant soft tissue dehiscence.

### 3.5 Incidence and risk factors for PSTD, MREC, and ML changes

#### 3.5.1 Qualitative analysis

Table 1 reports the mean incidence of PSTD, MREC, and ML apical shift at different time points based on data from prospective studies only. The mean incidence of PSTD after 1 year was 33.3%, and between 3 and 5 years, it was found to be 38.3%. After 5 years, the mean incidence of PSTD was 64.5%. The mean incidence of MREC between 1 and 3 years, and between 3 and 5 years was 47.8% and 45.7%, respectively. The mean incidence of ML changes was 15.3% within the first year, and 35.1% for follow‐up time points more than 5 years (Table 1). The mean depth of PSTD, MREC, and ML changes within 1 year was 0.33 mm, 0.39 mm and 0.37 mm, respectively, while their mean depth for follow‐up time points between 1 and 3 years was 0.43 mm, 0.52 mm, and 0.41 mm, respectively (Table 1). Tables 2 and S11 of the Appendix described in detail the putative risk factors associated with PSTD, MREC, and ML apical shift (see Table S11 in online *Journal of Periodontology*).

#### 3.5.2 Quantitative assessment of risk and protective factors for PSTD

For the outcome of PSTD, an immediate implant placement (27.42 (95% CI [24.49, 30.35], *p* < 0.001) showed to be significantly associated with an increased incidence of PSTD when compared to conventional therapy. Having received soft tissue augmentation procedure (−15.01 (95% CI [−22.68, ‐7.32]), *p* = 0.01), demonstrated a negative association with incidence of PSTD. When considering the depth of PSTD (in mm) when reported among study arms, we found that similar factors of immediate implant placement (−0.403 (95% CI [−0.75, −0.05]), *p* = 0.02), as well as soft tissue augmentation (−0.302 (95% CI [−0.53, −0.07]), *p* = 0.01) were statistically significant in the models. In addition, for this outcome MT also exhibited a significant association (−0.63 (95% CI [−0.87, −0.401]), *p* < 0.01).

#### 3.5.3 Quantitative assessment of risk and protective factors for ML apical shift

With regards to ML apical shift (in mm), after initial univariate assessment of variables (as with the outcome of MREC), the multilevel regression showed that immediate implant therapy (as compared to conventional implant placement, with a coefficient of 0.12 (95% CI [0.01, 0.22]), *p* = 0.01), among study arms was significantly associated with an increase in this outcome and thus instability and changes at the peri‐implant facial soft tissue margin. Whereas, having undergone a guided implant therapy (−0.09 (95% CI [−0.15, −0.04]), *p* = 0.01), having received bone grafting at the time of implant placement (−0.12 (95% CI [−0.22, −0.018], *p* = 0.02)), as well as soft tissue augmentation (−0.31 (95% CI [−0.45, −0.16], *p* < 0.001)) were significantly associated with stability and reduced changes in the peri‐implant mucosal levels. Furthermore, among site factors, mean KM width (−0.108 (95% CI [−0.18, −0.03]), *p* = 0.02), and MT (−0.14 (95% CI [−0.27, −0.01]), *p* < 0.01) also demonstrated statistically significant associations with this outcome, while increasing mean values of buccal bone thickness did not show significant associations in the model (−0.11 (95% CI [−0.36, 0.13], *p* = 0.35)).

#### 3.5.4 Quantitative assessment of risk and protective factors for MREC

For the outcome of MREC (in mm), the univariate analysis showed that age (−0.03 (95% CI [−0.06, −0.007], *p* = 0.03), time from loading (0.006 (95% CI [0.002, 0.01]), *p* < 0.01), radiographic MBL (0.11 mm (95% CI [0.06, 0.17]), *p* = 0.02), and KM width (−0.17 (95% CI [−0.25, −0.09]), *p* = 0.021), as well as the location of the implant in the arch (maxilla vs. mandible −0.38 (95% CI [0.14, 0.62]), *p* < 0.01) had a statistically significant effect on its values, whereas the variable of flap versus flapless (0.38 (95% CI [−0.006, 0.77]), *p* = 0.081), and single versus multiple implant placement (−0.13 (95% CI [−0.03, 0.305]), *p* = 0.11), failed to show a significant association with MREC. The multi‐level model, however, demonstrated that only KM width (−0.14 (95% CI [−0.19, −0.104], *p* = 0.01), and radiographic MBL (0.73 (95% CI [0.43, 1.03]), *p* < 0.01) remained statistically significant when other factors were accounted for.

Figure 2B summarizes the risk and protective factors for PSTD, ML apical shift and MREC identified with the multilevel regression analyses.

### 3.6 Outcomes of PSTD/MREC treatment and soft tissue augmentation

**FIGURE 3:**
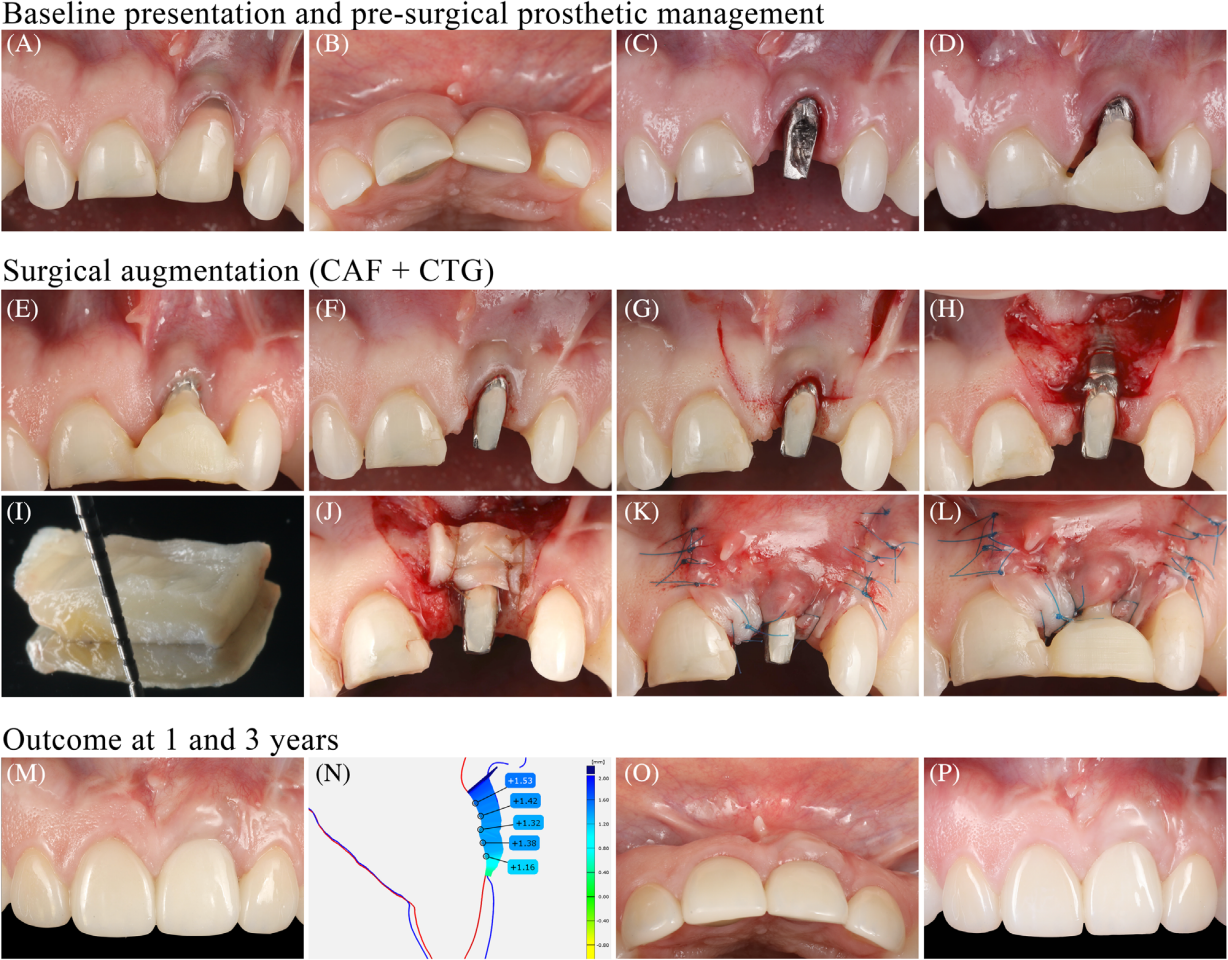
Management of a peri‐implant soft tissue dehiscence (PSTD) with a combined prosthetic‐surgical approach and the use of a connective tissue graft (CTG). (A,B) Baseline presentation. (C) Removal of the pre‐existing crown. (D) Delivery of a temporary crown, shorter and narrower compared to the previous crown. (E,F) Healing of the peri‐implant soft tissue 2 months after the delivery of the temporary crown. (G) Coronally advanced flap (CAF). (H) Split‐thickness flap elevation. (I) CTG harvested from the palate as an epithelialized soft tissue graft. (J) Graft sutured to the de‐epithelialized papillae and to the periosteum. (K) Flap coronally advanced and sutured. (L) Delivery of a temporary crown that is not compressing the flap. (M) Outcome after 1 year. Notice that a new crown was delivered on the implant and the right central incisors, while veneers were performed on the lateral incisors. Restorative therapy performed by Dr. Junying Li. (N) Volumetric digital analysis from the superimposition of the STL files at baseline and at 1 year, showing the volumetric gain at the treated implant sites. (O,P) Outcome at the 3‐year visit.

Treatment outcomes of PSTD/MREC management and peri‐implant soft tissue augmentation are displayed in detail in Table S17 (see Table S17 in online *Journal of Periodontology*). All the included studies reporting the outcomes of PSTD/MREC treatment involved the use of soft tissue grafting procedures (Figure 3). Coronally advanced flap (CAF) with connective tissue graft (CTG, obtained as an epithelialized soft tissue graft, and then de‐epithelialized) was the most common approach for the treatment of PSTD/MREC, and it achieved, on average, a PSTD coverage and MREC coverage of 92.7% and 85.5%, respectively. CAF with sub‐epithelial connective tissue graft (SCTG, obtained from the deepest layer of the palate) obtained a mean PSTD coverage and MREC coverage of 66% and 40%, respectively, while tunnel technique with CTG showed an average PSTD coverage of 59.8%. CAF in combination with acellular dermal matrix obtained a mean MREC coverage of 28%. CAF + CTG and tunnel technique + CTG exhibited a mean gain in KM width of 1.4 mm and 1.6 mm, respectively, while the MT gain obtained with all the above‐mentioned interventions ranged from 1 to 1.6 mm.

## 4 DISCUSSION

“Recession” is a term that has been extensively used to describe the condition of the soft tissue at implant sites. Based on the definition provided in the included studies, the present review recommended to categorize soft tissue dehiscence (“recession”) at implant sites as PSTD, ML apical shift, and MREC. PSTD and ML apical shift refer to implant esthetic complications, while MREC involves the exposure of the abutment/implant to the oral cavity, which may increase the risk of future disease.^247^

These conditions are not rare. The estimated prevalence of PSTD was 46.2%, while its incidence within the first 5 years from loading ranged between 33.3% and 38.3%. The incidence of ML apical shift within the first 5 years was estimated to be up to 23.6%. MREC had a prevalence of 23.1% and an overall incidence within the first 5 years from loading of 46‐48%.

The relatively high prevalence of PSTD in the anterior region has been previously highlighted by several authors.^8^, ^13^, ^82^, ^130^, ^220^ This observation may be attributed to the large variety of factors that may contribute to this condition. “Putative” risk indicators of PSTD that have been identified in the included studies encompassed implant malpositioned,^82^, ^172^, ^243^ lack of/limited KM width,^220^ reduced MT,^172^, ^220^ interproximal bone levels,^172^ buccal bone dehiscence,^220^ years in function,^220^ and presence of adjacent implants.^220^ Similarly, the “putative” risk factors that were found associated with PSTD in the included studies comprised time after implant placement,^129^ lack of KM,^129^ implants buccally positioned,^68^ interproximal marginal bone loss,^160^ thin phenotype,^167^ and non‐soft tissue‐augmented sites.^111^ Our regression analysis demonstrated that immediate implant placement, limited MT, and lack of soft tissue augmentation are risk factors for PSTD, and that, on the other hand, soft tissue augmentation procedures and having an adequate MT are protective factors against PSTD. The key role of soft tissue phenotype on the stability of the gingival margin in natural dentition has been well established.^247^, ^248^ Barootchi and colleagues demonstrated that a gingival thickness of at least 1.46 mm, in presence of at least 1.5 mm of keratinized tissue width, is necessary to maintain the stability of the gingival margin over 10 years following root coverage procedures.^247^ At implant sites, MT has been initially investigated in relation to the color of the peri‐implant soft tissue.^249^, ^250^, ^251^ Recent evidence has highlighted the role of MT on the stability of the peri‐implant soft and hard structures,^220^, ^252^, ^253^ and on the risk of developing peri‐implant diseases.^247^, ^254^ The impact of soft tissue augmentation seems to be even more crucial for immediate implant therapy, with several authors demonstrating stable soft tissue margin at post‐extraction dental implant augmented with CTG.^111^, ^235^, ^244^

The present study corroborated the importance of adequate MT and soft tissue augmentation as protective factors for PSTD, further emphasizing that the level of the peri‐implant soft tissue margin is mainly affected by soft tissue‐related parameters, and not by buccal bone dimensions. Therefore, it is not surprising that the most commonly performed augmentation technique for treating PSTDs involves the use of CTG,^6^, ^15^, ^221^, ^242^ which demonstrated stable clinical outcomes up to 5 years, regardless of the position and dimension of the buccal bone.^193^, ^241^ Soft tissue phenotype components (KM width and MT) were also found to be risk factors for ML apical shift. The regression analysis also revealed that immediate implant therapy had a higher risk of ML apical shift compared to conventional implant therapy, which is in line with previous studies.^68^, ^255^, ^256^, ^257^ This may be due to buccal shifting of the implant position during immediate implant therapy.^258^ Therefore, it is not surprising that guided implant therapy was identified as one of the protective factors for ML stability, together with bone grafting at implant placement, soft tissue augmentation, and adequate KM width and MT. There are no doubts that digital planning and guided implant surgery can facilitate the accuracy of implant placement in the ideal biological and prosthetically‐driven position,^259^, ^260^, ^261^ which can significantly reduce the risk of implant esthetic complications.^68^, ^201^, ^262^ Taking all these findings together, we believe that immediate implant placement should not be considered associated with a higher risk of implant esthetic complications when the procedure is properly planned and executed by expert operators.

When soft tissue dehiscence was reported in terms of MREC, based on the exposure of the implant shoulder, prosthetic abutment, or implant fixture to the oral cavity, our multi‐level model demonstrated that KM width and radiographic interproximal MBL were the only 2 risk factors associated with the condition. Although a shallow dehiscence of the soft tissue exposing a minimal portion of the abutment at posterior implants is usually not considered a main concern, a MREC with concomitant exposure of the rough surface of the implant fixture may jeopardize the health of the implant. Bacteria colonization of the implant fixture and subsequent biofilm formation on the implant surface can trigger the pathological inflammatory processes that lead to peri‐implant diseases.^263^, ^264^, ^265^ An adequate band of thick and adherent KM surrounding the implant‐supported crown may provide a soft tissue seal that facilitates patient's oral hygiene procedures, and protect the underlying peri‐implant tissues.^247^, ^266^, ^267^, ^268^, ^269^ Having an adequate band of KM has previously shown to promote better peri‐implant health‐related parameters compared to sites lacking/with inadequate KM width, which may have been also due to the protective effect of KM width against MREC.^220^, ^247^, ^270^, ^271^, ^272^ It has been speculated that this effect is due to the composition of the keratin layer, which is a highly insoluble and flexible band, that provides mechanical stability and protection to the epithelial cells and underneath connective tissue.^247^, ^267^, ^272^, ^273^

The limitations of the present review, namely, the relatively limited number of cross‐sectional studies and the scant information on the 3‐dimension implant position, reason for tooth loss, preoperative bone dimensions, as well as overcontoured prosthesis and wider implant placements relative to their ridge dimensions, should be mentioned. In addition, individual patient‐level data would have been beneficial to explore further correlations with the parameters of interest.

## 5 CLINICAL RECOMMENDATIONS

Based on the findings of the present review and on the existing literature, we recommend describing and classifying soft tissue dehiscence at healthy anterior implant sites (first/second premolar to first/second premolar) using the CEJ of the homologous contralateral tooth/ ideal position of the gingival margin as the reference.^13^ Healthy implants characterized by an apical shift of the peri‐implant soft tissue margin compared to this reference must be diagnosed as sites with PSTD. The classification of PSTD introduced by Zucchelli and coworkers further categorizes PSTDs in classes and subclasses,^13^ and it has been recently shown to be reproducible among operators with different background and experience^274^; therefore, it should be utilized as the classification system of reference for these conditions. In addition to PSTD classes and subclasses, we also recommend identifying the presence/absence of concomitant exposure of the implant fixture (“PSTD+” in case of exposure), as this scenario may not only be associated with esthetic concern, but may also put the implant on a higher risk of developing peri‐diseases.

For healthy posterior implant sites and for non‐esthetic cases (e.g., overdentures, full‐arch cases), the definition of MREC, which is based on the presence/absence of a mucosal cleft that exposes the implant shoulder, prosthetic abutment or implant surface,^26^ should be preferred. The exposure of the implant fixture in the oral cavity should be identified as MREC+, and it should be closely monitored given as it may expose the site to a higher risk of future disease.

Although ML change over time is an interesting outcome measure of implant therapy, we strongly recommend reporting this outcome in combination with PSTD (for anterior esthetic cases) or MREC (for posterior or non‐esthetic cases).

Studies assessing the outcomes of implant therapy should always report the prevalence (cross‐sectional studies) and incidence (prospective studies) of PSTD/MREC, together with their related clinical, esthetic, and patient‐reported outcomes of interest (Figure 4A). Following these above‐mentioned recommendations may contribute to promote a more homogenous and standardized format of reporting soft tissue‐related data and esthetic findings at implant sites. Figure 4B and C depict the above‐mentioned clinical recommendations for assessing and reporting soft tissue related outcomes of implant therapy. Lastly, readers should be aware that the findings and recommendations of this review are valid for implants in the absence of disease, and that these guidelines should not be applied to diseased implants until the pathological condition is solved.

FIGURE 4(A) Recommended diagnostic system and format for assessing and reporting soft tissue dehiscence at implant sites. Legend. AM: adherent mucosa; CEJ: cemento‐enamel junction; IDES: implant dehiscence coverage esthetic score; KM: keratinized mucosa; mPES: modified pink esthetic score; MREC: mucosal recession; MT: mucosal thickness; PD: probing depth; PES: pink esthetic score. PROMs: patient‐reported outcome measures; PSTD: peri‐implant soft tissue dehiscence; Pt EST: patient‐reported esthetics. (B) Clinical scenarios depicting the application of the new proposed system for diagnosing and reporting peri‐implant soft tissue dehiscence (PSTD) at anterior implant sites. Legend. AM: adherent mucosa; KM: keratinized mucosa; MT: mucosal thickness; NA: not applicable. PD: probing depth; PES: pink esthetic score. Pt EST: patient‐reported esthetics. VAS: visual analogue scale. (C) Clinical scenarios depicting the application of the new proposed system for diagnosing and reporting mucosal recession (MREC) at implants in the posterior and/or non‐esthetic regions. Legend. AM: adherent mucosa; KM: keratinized mucosa; MT: mucosal thickness; PD: probing depth; PES: pink esthetic score. Pt EST: patient‐reported esthetics. VAS: visual analogue scale.

**Figure:**
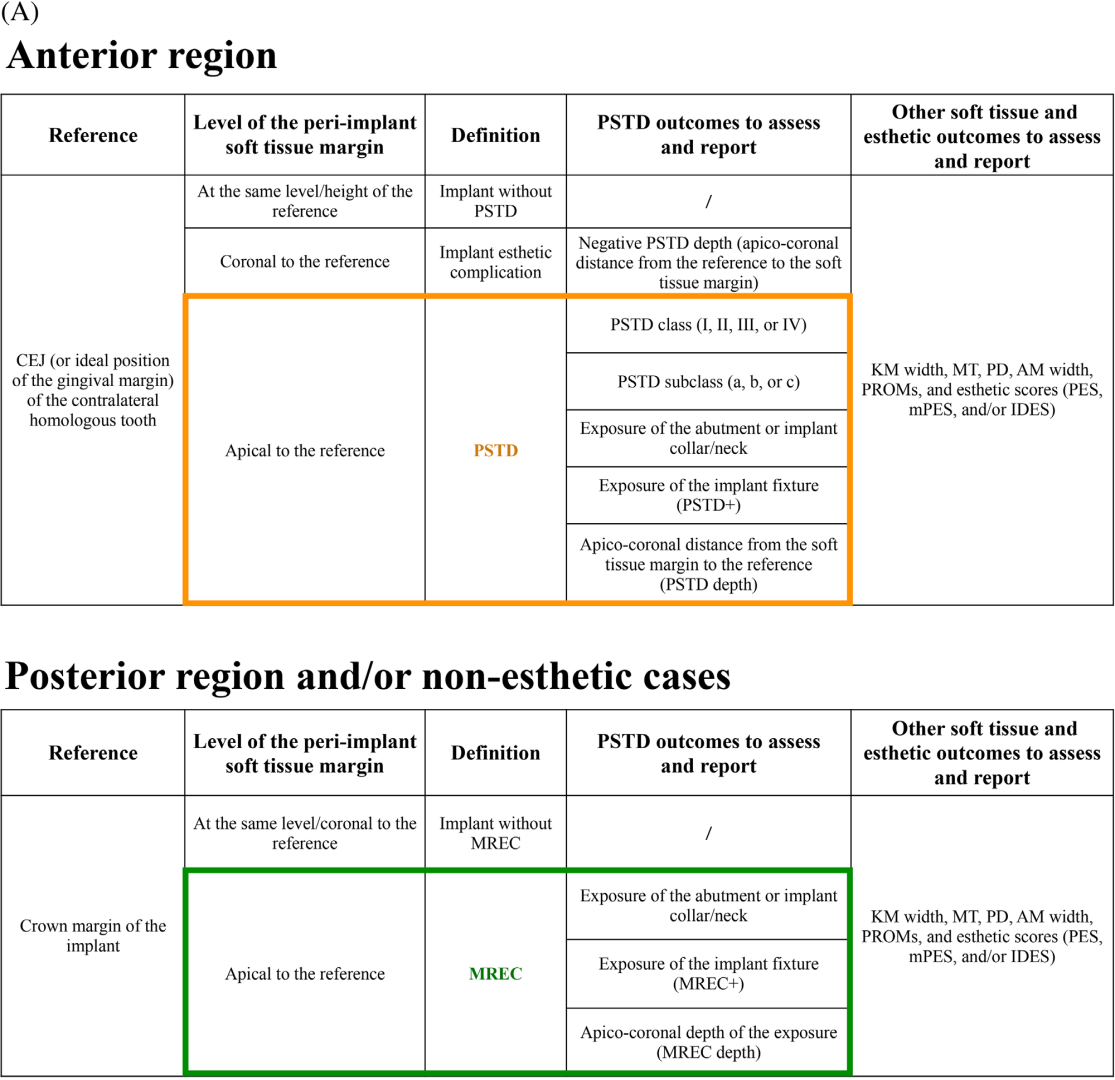

**Figure:**
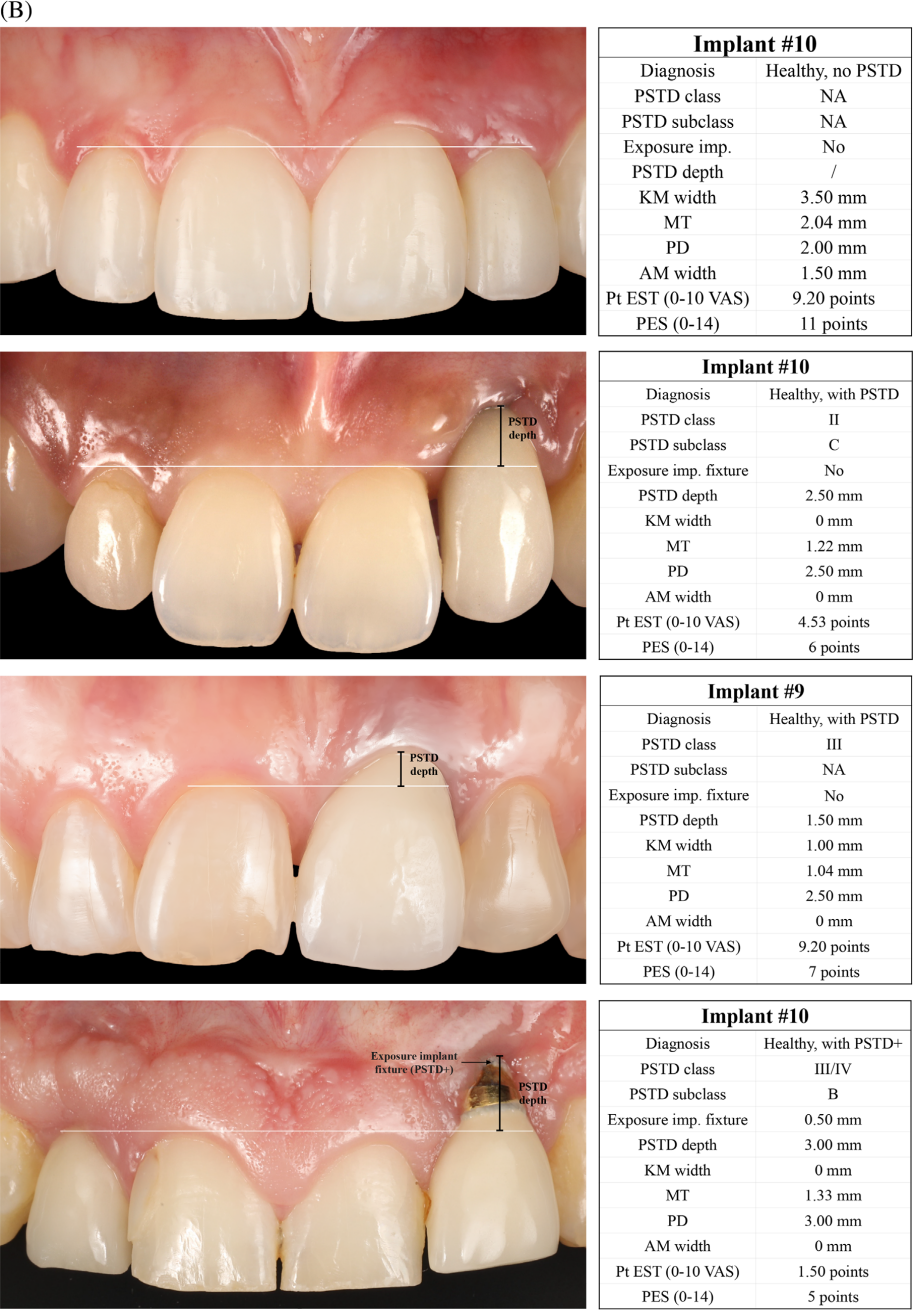

**Figure:**
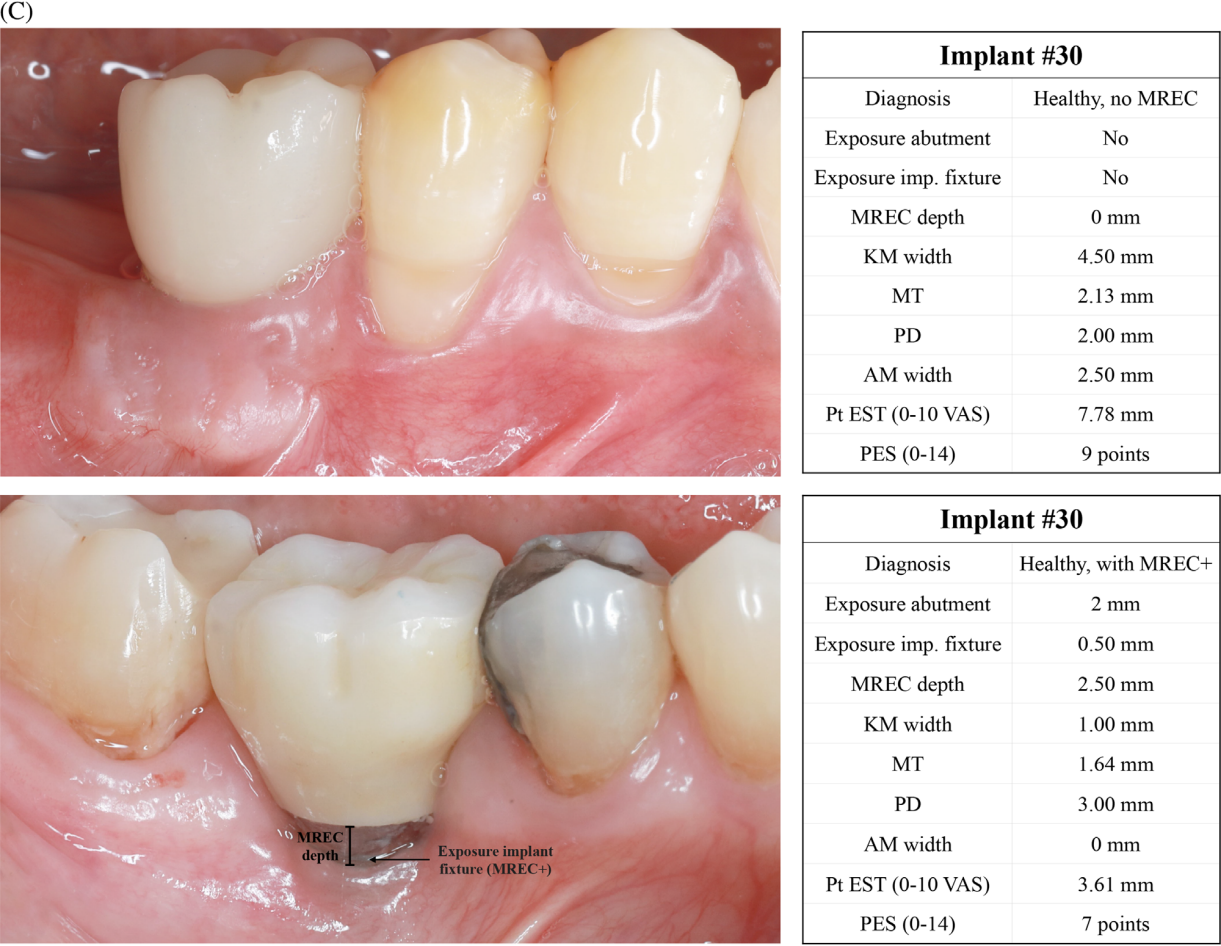

## 6 CONCLUSIONS

Based on the current available evidence, and within the limitations of this study, the following conclusions can be drawn:

1. Soft tissue dehiscences at implant sites in the absence of disease have been assessed and reported as PSTD, ML apical shift, and MREC.
2. These conditions are not rare findings. The estimated prevalence of PSTD and MREC was 46.2% and 23.1%, while the estimated incidence of PSTD, MREC, and ML apical shift within the first 5 year from loading was up to 38.3%, 47.8%, and 23.6%, respectively.
3. Risk factors for PSTD included reduced MT, immediate implant placement, and lack of soft tissue augmentation.
4. Risk factors for ML apical shift included reduced MT, lack of/limited KM width, and immediate implant therapy, while guided implant surgery, bone grafting at implant placement, soft tissue augmentation, and adequate KM and MT were protective factors for the stability of ML.
5. Risk factors for MREC included lack of/limited KM width, and interproximal marginal bone loss.
6. Several “putative” risk indicators and risk factors of PSTD, ML apical shift, and MREC were reported in the included studies, and involved implant malpositioning, surgical factors, soft tissue‐related parameters, bone‐related parameters, and prosthetic factors.
7. Connective tissue graft‐based soft tissue augmentation procedures were the most common and successful therapies for the management of PSTDs and MRECs.
8. Based on the findings of the present review, a new diagnostic system and format for assessing and reporting soft tissue dehiscence at anterior and posterior implant sites was proposed.

## AUTHOR CONTRIBUTIONS

Lorenzo Tavelli and Shayan Barootchi designed the study; Lorenzo Tavelli and Shayan Barootchi performed the literature search, initial screening, article selection, data extraction, and risk of bias assessment; Lorenzo Tavelli performed the qualitative analysis; Shayan Barootchi contributed to the study methodology and conducted the statistical analysis; Lorenzo Tavelli led the writing. Shayan Barootchi revised the manuscript.

## CONFLICT OF INTEREST STATEMENT

The authors report no conflict of interest related to the conduction of this systematic review.

## FUNDING INFORMATION

The authors received no specific funding for this work.

## Supporting information



Supporting Information

## Data Availability

The data that support the findings of this study are available in the Supplementary Appendix.

## Footnotes

* RStudio, Version 1.3.959, Boston, MA, USA

† See refs. ^15^, ^35^, ^41^, ^42^, ^46^, ^47^, ^48^, ^49^, ^50^, ^52^, ^54^, ^60^, ^62^, ^63^, ^64^, ^65^, ^66^, ^70^, ^72^, ^73^, ^75^, ^78^, ^84^, ^86^, ^89^, ^91^, ^92^, ^95^, ^96^, ^97^, ^98^, ^99^, ^103^, ^105^, ^108^, ^109^ ^,^ ^111^, ^113^, ^115^, ^116^, ^117^, ^124^, ^126^, ^127^, ^132^, ^133^, ^134^, ^137^, ^138^, ^146^, ^149^, ^150^, ^152^, ^157^, ^158^, ^162^, ^163^, ^164^, ^166^, ^167^, ^169^, ^176^, ^179^, ^180^, ^184^ ^,^ ^192^, ^196^, ^197^, ^199^, ^202^, ^204^, ^206^, ^207^, ^208^, ^209^, ^210^, ^211^, ^212^, ^215^, ^216^, ^217^, ^228^, ^229^, ^231^, ^235^, ^236^, ^239^, ^240^, ^244^, ^245^, ^246^

‡ See refs. ^6^, ^10^, ^11^, ^14^, ^27^, ^28^, ^36^, ^38^, ^39^, ^40^, ^43^, ^44^, ^45^, ^51^, ^53^, ^55^, ^56^, ^57^, ^58^, ^59^, ^61^, ^68^, ^69^, ^71^, ^74^, ^76^, ^79^, ^80^, ^81^, ^83^, ^85^, ^87^, ^88^, ^90^, ^93^ ^,^ ^94^, ^101^, ^102^, ^104^, ^106^, ^107^, ^110^, ^112^, ^114^, ^118^, ^119^, ^120^, ^121^, ^122^, ^123^, ^125^, ^128^, ^129^, ^131^, ^136^, ^139^, ^140^, ^141^, ^142^, ^143^, ^144^, ^145^, ^148^, ^151^, ^153^, ^154^, ^155^, ^156^, ^160^, ^165^, ^168^, ^170^ ^,^ ^173^, ^177^, ^181^, ^182^, ^183^, ^185^, ^186^, ^187^, ^188^, ^189^, ^191^, ^193^, ^194^, ^198^, ^200^, ^203^, ^205^, ^213^, ^214^, ^219^, ^221^, ^223^, ^224^, ^225^, ^230^, ^232^, ^233^, ^234^, ^237^, ^238^ ^,^ ^241^, ^242^

§ See refs. ^33^, ^34^, ^37^, ^67^, ^77^, ^82^, ^100^, ^130^, ^135^, ^147^, ^159^, ^161^, ^171^, ^172^, ^174^, ^175^, ^178^, ^190^, ^195^, ^201^, ^218^, ^220^, ^222^ ^,^ ^226^, ^227^, ^243^

¶ See refs. ^10^, ^11^, ^27^, ^28^, ^36^, ^38^, ^39^, ^40^, ^41^, ^42^, ^43^, ^44^, ^45^, ^46^, ^47^, ^48^, ^49^, ^50^, ^51^, ^52^, ^53^, ^54^, ^55^, ^56^, ^57^, ^58^, ^59^, ^60^, ^61^, ^62^, ^63^, ^64^, ^65^, ^66^, ^68^, ^69^, ^70^, ^71^, ^72^, ^73^, ^74^, ^75^, ^76^, ^78^, ^79^, ^80^, ^81^, ^83^, ^84^, ^85^, ^86^, ^87^, ^88^, ^89^, ^90^, ^91^, ^92^, ^93^, ^94^, ^95^, ^96^, ^97^, ^98^, ^99^, ^101^, ^102^, ^103^, ^104^, ^105^, ^106^, ^107^, ^108^, ^109^, ^110^, ^111^, ^112^, ^113^, ^114^, ^115^, ^116^, ^117^, ^118^, ^119^, ^120^, ^121^, ^122^, ^123^, ^124^, ^125^, ^126^, ^127^, ^128^, ^129^, ^131^, ^132^, ^133^, ^134^, ^136^, ^137^, ^138^, ^139^, ^140^, ^141^, ^142^, ^143^, ^144^, ^145^, ^146^, ^148^, ^149^, ^150^, ^151^, ^152^, ^153^, ^154^, ^155^, ^156^, ^158^, ^160^ ^,^ ^162^, ^163^, ^164^, ^165^, ^166^, ^167^, ^168^, ^169^, ^170^, ^173^, ^177^, ^179^, ^180^, ^181^, ^182^, ^183^, ^184^, ^185^, ^186^, ^187^, ^188^, ^189^, ^191^, ^192^, ^194^, ^196^, ^197^, ^198^, ^199^, ^200^, ^202^, ^204^, ^205^, ^206^, ^207^, ^208^, ^209^, ^210^, ^211^, ^212^, ^213^, ^214^, ^215^, ^216^, ^217^, ^219^, ^223^, ^224^, ^225^, ^228^, ^229^, ^230^, ^231^, ^232^, ^233^, ^234^, ^235^, ^236^, ^237^, ^238^, ^240^, ^244^, ^245^, ^246^
