# Supplementary material for: Prevalence, incidence, risk, and protective factors for soft tissue dehiscences at implant sites in the absence of disease: An AO/AAP systematic review and meta‐regression analysis
Source: J Periodontol. 2025 Jun 9;96(6):562–86. doi: 10.1002/JPER.24-0119 (PMC12273775; doi:10.1002/JPER.24-0119)
Supplement: Supplementary file 1 — Supporting Information [file JPER-96-562-s001.docx]

**Prevalence, incidence, and risk and protective factors for soft tissue dehiscences at implant sites in the absence of disease: An American Academy of Periodontology and Academy of Osseointegration Best Evidence Systematic Review and Meta-regression analysis**

Lorenzo Tavelli and Shayan Barootchi

**Supplementary Appendix**

**Content of the Supplementary Appendix**

| Page 3 | Additional information on eligible studies. |
| --- | --- |
| Page 4 | Search strategy, article selection, and data extraction. |
| Page 5 | Methodological quality and risk of bias assessment. |
| Page 6 | Supplementary Table 1. Reference and the reasons for the excluded articles. |
| Page 7 | Supplementary Table 2. References of the included studies. |
| Page 8 | Supplementary Table 3. Study characteristics at baseline. |
| Page 25 | Supplementary Table 4. General characteristics of the included cross sectional studies. |
| Page 28 | Supplementary Table 5. Prevalence and amount/depth of PSTD and MREC reported in the included cross sectional studies. |
| Page 32 | Supplementary Table 6. Clinical, radiographic, esthetic, and patient-reported outcomes of the included cross sectional studies. |
| Page 35 | Supplementary Table 7. General characteristics of the included prospective studies. |
| Page 70 | Supplementary Table 8. Incidence and amount/depth of Mucosal Level (ML) changes, peri-implant soft tissue dehiscence (PSTD), and mucosal recession (MREC). |
| Page 98 | Supplementary Table 9. Clinical, radiographic, esthetic, and patient-reported outcomes of the included prospective studies. |
| Page 128 | Supplementary Table 10. Risk indicators for PSTD and MREC reported in the included studies. |
| Page 129 | Supplementary Table 11. Summary of the parameters correlated with PSTD/MREC/ML apical changes in the included prospective studies. |
| Page 130 | Supplementary Table 12. General characteristics of the included studies reporting on soft tissue augmentation and treatment of soft tissue dehiscence. |
| Page 132 | Supplementary Table 13. Clinical, esthetic, and patient-reported outcomes of soft tissue augmentation and treatment of soft tissue dehiscence at implant sites. |
| Page 134 | Supplementary Table 14. Risk of bias assessment of the included cross sectional studies using The Joanna Briggs Institute (JBI) Critical Appraisal checklist for analytical cross sectional studies. |
| Page 136 | Supplementary Table 15. Risk of bias assessment of the included randomized clinical trials using the risk-of-bias 2 (ROB2) tool. |
| Page 141 | Supplementary Table 16. Risk of bias assessment of the included non-randomized interventional studies using the ROBINS-I tool. |
| Page 146 | Supplementary Table 17. Outcomes of the studies reporting on PSTD/MREC treatment and peri-implant soft tissue augmentation. |
| Page 147 | References |

**Additional information on eligible studies**

To specifically address the focused questions, only cross sectional and prospective studies were included in this systematic review's qualitative and quantitative assessment. Studies were considered eligible for inclusion if they met the following criteria in at least one study arm: i) Reporting information on soft tissue “recession” / stability of the soft tissue margin around osseointegrated dental implants; ii) implants being functionally loaded for at least 6 months; iii) having at least one arm with ≥ 10 patients; and iv) clear definition of soft tissue “recession”/dehiscence. Reasons for article exclusion included: i) Retrospective studies and case reports; ii) Case series with < 10 patients; and iii) unclear assessment of soft tissue “recession”/dehiscence. RCTs and non-RCTs with at least one treatment arm meeting the above-mentioned eligibility criteria were included in the present review. Data from the excluded treatment arm(s) were not considered.

**Additional study outcomes retrieved from the eligible studies**

The general study characteristics retrieved from the eligible studies included year of publication, study design (single- vs multi-center, cross-sectional study, RCT, and prospective non-RCT), setting (university and/or private practice), geographic location, patient’s age at baseline, inclusion of smokers (current and/or former smokers), number of patients and implant sites, percentage of females included, study funding (sponsored by companies, foundation or none), type of implant, single and/or multiple sites, maxilla and/or mandibular sites, anterior and/or posterior sites, type of intervention, timing of implant placement (in healed ridge, immediate or early), flap vs flapless, use of surgical guide, use of cover screw/healing abutment/immediate provisionalization, type of loading (immediate, early, delayed), use of bone graft, type of bone graft, use of soft tissue graft, type of soft tissue graft, characteristics of the final restoration, follow-up time points from implant loading, and definition of soft tissue dehiscence (“recession”). The mean and standard deviation (SD) of the following outcomes were collected from the included studies at the different time points: i) prevalence and incidence of soft tissue dehiscence (for cross-sectional studies and prospective studies, respectively); ii) depth of soft tissue dehiscence; iii) keratinized mucosa (KM) width, mucosal thickness (MT), and probing depth (PD); iv) buccal bone thickness (BBT) and marginal bone loss (MBL); v) patient-reported esthetics assessed using a 0-10 or 0-100 visual analogue scale (VAS); and vi) professional esthetic assessment using the pink esthetic score (PES) ^17^ or the modified pink esthetic score (mPES)^30^. In addition to these outcomes, parameters associated with soft tissue dehiscence (“recession”) were collected, together with the respective odds ratio (OR), 95% confidence interval, and p-value, when possible.

For studies reporting on the outcomes of soft tissue augmentation at implant sites, or treatment of soft tissue dehiscence/implant esthetic complications, the type of flap reflected, pre-surgical prosthetic treatment, and professional esthetic outcomes using the implant soft tissue dehiscence coverage esthetic score (IDES)^18^, the PES^17^, the mPES^30^, and 0-10 or 0-100 VAS were also assessed.

**Information sources and search strategy**

To identify eligible articles, detailed search strategies were modelled for MEDLINE (via PubMed), EMBASE, and Cochrane Central Register of Controlled Trials (CENTRAL) databases. Searches were conducted to identify papers published up to October 31^th^, 2023, based on the following comprehensive search strategy: “("dental implant") AND ("recession" OR "dehiscence" OR "deficiency" OR "exposure" OR "gingival margin" OR "facial margin" OR "crown height changes" OR "crown length changes" OR "apical migration" OR "apical displacement" OR "gum loss" OR "esthetic complication")”. The search strategy was primarily designed for the MEDLINE database with a string of medical subject headings and free-text terms and then modified appropriately for other databases. No restrictions were set for language. The search results were downloaded to a bibliographic database to facilitate duplicate removal and cross-reference checks.

The reference lists of the retrieved studies for full-text screening and previous reviews in periodontal regeneration were screened. A manual search was also performed in the *Journal of Periodontology,* *Journal of Clinical Periodontology, Journal of Periodontal* *Research,* and *International Journal of Periodontics and Restorative Dentistry.* Previous systematic reviews assessing peri-implant soft tissue dehiscences and implant esthetic complications were also assessed.^4, 8, 9, 14, 19, 31-36^

**Article selection process and data extraction**

Two independent reviewers (L.T. and S.B.) screened the titles and abstracts (if available) of the entries identified in the literature search in duplicate and independently. Next, the full-text version of all studies that potentially met the eligibility criteria or for which there was insufficient information in the title and abstract to make a decision were obtained. Any article considered potentially relevant by at least one of the reviewers was included in the next screening phase. Subsequently, the full-text publications were also evaluated in duplicate and independently by the same review examiners. Disagreements between the review authors were resolved by open discussion. All articles that did not meet the eligibility criteria were excluded, and the reasons for exclusion were noted. Inter-examiner agreement following full-text assessment was calculated via kappa statistics. Any missing information that could contribute to this systematic review was requested to the corresponding author(s) via email communication. In the case of multiple publications reporting on the same study or investigating the same cohort at different follow-up intervals (or secondary analysis of the same data), it was decided to pool together all relevant details as a single report with the most comprehensive data for inclusion in the qualitative and quantitative analyses.

Two examiners (L.T. and S.B.) independently retrieved all information described above (paragraph 2.4) from the included articles using a data extraction sheet specifically designed for this review. All values were extracted from the selected publications as mean ± standard deviation (SD).

**Methodological quality and risk of bias assessment**

The risk of bias of the included cross sectional studies was assessed using **The Joanna Briggs Institute (JBI) Critical Appraisal** checklist for analytical cross sectional studies (Moola et al., 2017). This tool includes the evaluation of the following 8 components: i) Were the criteria for inclusion in the sample clearly defined?; ii) Were the study subjects and the setting described in detail?; iii) Was the exposure measured in a valid and reliable way?; iv) Were objective, standard criteria used for measurement of the condition?; v) Were confounding factors identified?; vi) Were strategies to deal with confounding factors stated?; vii) Were the outcomes measured in a valid and reliable way?; viii) Was appropriate statistical analysis used? The response options for each domain are “yes”, “no”, “unclear” and “not applicable”. The overall risk of bias was considered low if a study provided detail information in all domains. The risk of bias was judged to be unclear if at least one domain was judged unclear, while the risk of bias was considered high if at least one domain was considered at high risk of bias (“no” in at least one domain).

The recommendation of the Cochrane collaboration group for randomized trials **(risk-of-bias 2 [ROB2])** (J. A. C. Sterne et al., 2019) was utilized to assess the risk of bias of the included studies. This method includes the evaluation of the following 5 domains: i) Bias arising from the randomization process; ii) Bias due to deviations from intended intervention; iii) Bias due to missing outcome data; iv) Bias in measurement of the outcome; and v) Bias in selection of the reported result. The risk of bias in each domain was be rated as low, unclear/ “some concerns” or high. The overall risk of bias was categorized as follows: i) Low (plausible bias unlikely to seriously affect the results) if a study provided detailed information and low risk of bias for all 5 domains; ii) Unclear/ “some concerns” (plausible bias that raises some doubts about the results) if at least one domain was judged unclear/ or having some concerns; and High (plausible bias that seriously weakens confidence in the results) if at least one domain was considered at high risk of bias.

The risk of bias of non-randomized prospective interventional studies was assessed using the **ROBINS-I** tool (J. A. Sterne et al., 2016). This tool involved the evaluation of the following 7 domains: i) Bias due to confounding; ii) Bias in selection of participants; iii) Bias in classification of intervention; iv) Bias due to deviations from intended interventions; v) Bias due to missing data; vi) Bias in measurement of outcomes; and vii) Bias in selection of the reported result. The overall risk of bias was considered low if a study provided detail information in all domains. The risk of bias was judged to be unclear if at least one domain was judged unclear, while the risk of bias was considered high if at least one domain was considered at high risk of bias.

**Supplementary Table 1.** Reference and the reasons for the excluded articles.

| **Reason for exclusion** | **Reference** |
| --- | --- |
| Data on PSTD/MREC/ML changes not reported or unclear  (N=118) | (Abd-Elrahman, Shaheen, Askar, & Atef, 2020; Acham et al., 2017; Amato et al., 2022; Amato & Cracknell, 2022; Amorfini, Storelli, Mosca, Scanferla, & Romeo, 2018; Arnhart et al., 2012; Assaf, Assaf, Antoniazzi, Osório, & França, 2017; Bartols, Kasprzyk, Walther, & Korsch, 2018; Bienz et al., 2023; Bogaerde, Rangert, & Wendelhag, 2005; Boven, Meijer, Slot, Vissink, & Raghoebar, 2015; Brägger, Hugel-Pisoni, Bürgin, Buser, & Lang, 1996; Bruno, O'Sullivan, Badino, & Catapano, 2014; Cannizzaro, Felice, Leone, Checchi, & Esposito, 2011; Canullo et al., 2019; Chang & Wennstrom, 2010; Chappuis et al., 2018; Cho, Moon, Chung, & Kim, 2011; Chu et al., 2021; Chu et al., 2020; Clauser, Sforza, Menini, Kalemaj, & Buti, 2020; Covani et al., 2007; de Carvalho, de Carvalho, & Consani, 2013; de Oliveira Silva et al., 2020; Degidi, Nardi, Daprile, & Piattelli, 2014; Degidi, Novaes, Nardi, & Piattelli, 2008; Doornewaard et al., 2020; Doornewaard et al., 2019; Eeckhout, Bouckaert, Verleyen, De Bruyckere, & Cosyn, 2020; Eeckhout et al., 2023; Eghbali, De Bruyn, Cosyn, Kerckaert, & Van Hoof, 2016; Eladl, Sholkamy, & Emara, 2021; Elaskary et al., 2023; Esposito et al., 2018; Galindo-Moreno et al., 2017; Ghanaati et al., 2014; Ghazal et al., 2019; Girbes-Ballester, Vina-Almunia, Penarrocha-Oltra, & Penarrocha-Diago, 2016; Groenendijk, Staas, Bronkhorst, Raghoebar, & Meijer, 2023; Hammerle et al., 2023; Hassan, 2009; Hernández et al., 2022; M. Hosseini, Worsaae, & Gotfredsen, 2020; Hutton, Johnson, Barwacz, Allareddy, & Avila-Ortiz, 2018; Janyaphadungpong, Serichetaphongse, & Pimkhaokham, 2019; Jeong et al., 2011; Jonker, Strauss, et al., 2021; Jovanovic, Spiekermann, & Richter, 1992; Jung, Hälg, Thoma, & Hämmerle, 2009; Jung et al., 2017; Karkar, Metwally, & Mohsen, 2023; King et al., 2016; Kniha et al., 2019; Konstantinidis, Siormpas, Kontsiotou-Siormpa, Mitsias, & Kotsakis, 2016; Lambert, Morris, & Ochi, 2000; Lazzari et al., 2022; H. Lee et al., 2020; Lertwongpaisan, Amornsettachai, Panyayong, & Suphangul, 2023; Levin & Clark-Perry, 2022; Li, Gao, Zhou, & Zhu, 2021; Liaje, Ozkan, Ozkan, & Vanlioğlu, 2012; Magdy et al., 2021; Maiorana et al., 2018; Manfredini, Poli, Guerrieri, Beretta, & Maiorana, 2023; Mayfield, Nobréus, Attström, & Linde, 1997; Mazor, Peleg, Garg, & Chaushu, 2000; Meloni et al., 2019; Mendoza-Azpur et al., 2016; R. Mengel & L. Flores-de-Jacoby, 2005; R. Mengel & L. F. Flores-de-Jacoby, 2005; Merli et al., 2018; Nahid, Bansal, & Pandey, 2022; Naishlos et al., 2021; Nissan, Mardinger, Strauss, et al., 2011; Noelken, Moergel, Kunkel, & Wagner, 2018; Norton, 2004; Omara, Abdelwahed, Ahmed, & Hindy, 2016; Östman, Chu, Drago, Saito, & Nevins, 2020; Parel & Schow, 2005; Patel, Mardas, & Donos, 2013; Pellicer-Chover et al., 2019; Petsos, Trimpou, Eickholz, Lauer, & Weigl, 2017; Pirc et al., 2021; Pohl, Cede, Pokorny, Haas, & Pohl, 2022; Pohl et al., 2017; Raj et al., 2022; Restoy-Lozano et al., 2015; Rompen, Raepsaet, Domken, Touati, & Van Dooren, 2007; Rosano et al., 2023; Saito et al., 2018; Salman & Bede, 2022; Sarnachiaro, Chu, Sarnachiaro, Gotta, & Tarnow, 2016; Schepke, Meijer, Kerdijk, Raghoebar, & Cune, 2017; Schmitt et al., 2021; Schmitt et al., 2016; Schropp, Wenzel, Spin-Neto, & Stavropoulos, 2015; Shadid, 2022; Shibly, Kutkut, Patel, & Albandar, 2012; Simion, Fontana, Rasperini, & Maiorana, 2007; Sisti et al., 2012; Song et al., 2023; Spies, Balmer, Patzelt, Vach, & Kohal, 2015; Spies, Sperlich, Fleiner, Stampf, & Kohal, 2016; Stefanini, Rendon, & Zucchelli, 2020; Tabrizi, Pourdanesh, Zare, Daneste, & Zeini, 2013; Telleman, Meijer, Vissink, & Raghoebar, 2013; Telleman, Raghoebar, Vissink, & Meijer, 2017; Thoma, Gasser, Hammerle, Strauss, & Jung, 2022; Thoma, Gasser, Jung, & Hammerle, 2020; Thoma et al., 2018; Vellis, Kutkut, & Al-Sabbagh, 2019; Verdugo, Laksmana, D'Addona, & Uribarri, 2020; Vervaeke et al., 2018; Waller et al., 2020; F. Wang et al., 2017; I. C. Wang, Chan, Kinney, & Wang, 2020; Weerapong, Sirimongkolwattana, Sastraruji, & Khongkhunthian, 2019; Zuercher et al., 2023) |
| Retrospective design  (N=21) | (Artzi, Carmeli, & Kozlovsky, 2006; Bienz et al., 2017; Bonnet, Karouni, & Antoun, 2018; Cairo et al., 2020; Chen, Darby, Reynolds, & Clement, 2009; Evans & Chen, 2008; Frisch & Ratka-Kruger, 2020; Fürhauser et al., 2022; Jensen, Cullum, & Baer, 2009; Khzam, Mattheos, Roberts, Bruce, & Ivanovski, 2014; B. S. Kim et al., 2009; Y. K. Kim & Yun, 2014; Kniha et al., 2020; Kolerman et al., 2018; Kuchler, Chappuis, Gruber, Lang, & Salvi, 2016; Y. M. Lee et al., 2012; Lin, Kuo, Chiu, Chen, & Wang, 2023; Papaspyridakos, Bordin, Natto, El-Rafie, et al., 2019; Papaspyridakos, Bordin, Natto, Kim, et al., 2019; Siormpas, Mitsias, Kontsiotou-Siormpa, Garber, & Kotsakis, 2014; Zigdon & Machtei, 2008) |
| Follow-up after crown delivery < 6 months (N=19) | (Abdelsamie, Elarab, Ibrahim, & Rahman, 2022; Abrahamsson, Andersson, Wälivaara, & Isaksson, 2011; Cairo et al., 2017; Cosyn et al., 2021; Hinze, Janousch, Goldhahn, & Schlee, 2018; Hu et al., 2018; Huang, Ogata, Hanley, Finkelman, & Hur, 2016; Huynh-Ba et al., 2016; Jiang, Di, Ren, Zhang, & Lin, 2020; Jonker, Gil, et al., 2021; Jonker, Strauss, et al., 2021; D. H. Lee, Choi, Jeong, Xuan, & Kim, 2011; Nissan, Mardinger, Calderon, Romanos, & Chaushu, 2011; Patil et al., 2013; Schneider et al., 2014; Thoma et al., 2016; van Kesteren, Schoolfield, West, & Oates, 2010a; Xu et al., 2023; Zang et al., 2022) |
| Cohorts with < 10 subjects (N=12) | (Jeng & Chiang, 2020; D.-W. Lee et al., 2015; Mazor, Steigmann, Leshem, & Peleg, 2004; Menini et al., 2022; Moreno Rodríguez, Guerrero Gironés, Pecci Lloret, & Ortiz Ruiz, 2021; Palattella, Torsello, & Cordaro, 2008; Pelekanos & Pozidi, 2017; Pirker & Kocher, 2009; Poli, Manfredini, Maiorana, Salina, & Beretta, 2023; Rodriguez, Girones, Lloret, & Ruiz, 2021; Saito, Chu, & Tarnow, 2022; H. L. Wang, Misch, & Neiva, 2004) |

**Supplementary Table 2.** References of the included studies.

| **References of the 221 included study** |
| --- |
| (Able, Sartori, Younes, & Bombarda, 2021; Adibrad, Shahabuei, & Sahabi, 2009; Anderson, Inglehart, El-Kholy, Eber, & Wang, 2014; Andersson, Odman, Lindvall, & Branemark, 1998; Apaza-Bedoya et al., 2023; Arora & Ivanovski, 2017, 2018; Arora, Khzam, Roberts, Bruce, & Ivanovski, 2017; Atef, El Barbary, Dahrous, & Zahran, 2021; Barone et al., 2016; Barone, Toti, Quaranta, Derchi, & Covani, 2015; Bengazi, Wennstrom, & Lekholm, 1996; Benic et al., 2017; Benic et al., 2012; Benitez Silva et al., 2022; Bianchi & Sanfilippo, 2004; Bittner, Planzos, Volchonok, Tarnow, & Schulze-Spate, 2020; Bittner, Schulze-Spate, et al., 2020; Bittner et al., 2019; Blanes, Bernard, Blanes, & Belser, 2007; Block et al., 2009; Bonino et al., 2018; Brägger, Hämmerle, & Lang, 1996; Bressan et al., 2017; Brunello et al., 2022; Burkhardt, Joss, & Lang, 2008; Buser, Chappuis, Bornstein, et al., 2013; Buser, Chappuis, Kuchler, et al., 2013; Buser et al., 2009; Buser et al., 2011; Bushahri et al., 2021; Cabello, Rioboo, & Fábrega, 2013; Canullo, Caneva, & Tallarico, 2017; Canullo, Iurlaro, & Iannello, 2009; Cardaropoli, Gaveglio, Gherlone, & Cardaropoli, 2014; Cecchinato, Lops, Salvi, & Sanz, 2015; Chan et al., 2019; Chang, Wennstrom, Odman, & Andersson, 1999; Chen, Darby, & Reynolds, 2007; Chung, Rungcharassaeng, Kan, Roe, & Lozada, 2011; Clem et al., 2023; Cooper et al., 2010; Cooper et al., 2015; Cooper et al., 2019; Cooper et al., 2014; Cordaro, Torsello, & Roccuzzo, 2009; Cosyn, De Bruyn, & Cleymaet, 2013; Cosyn & De Rouck, 2009; Cosyn et al., 2022; Cosyn et al., 2011; Cosyn et al., 2016; Cosyn, Pollaris, Van der Linden, & De Bruyn, 2015; Cosyn, Sabzevar, & De Bruyn, 2012; Covani, Canullo, Toti, Alfonsi, & Barone, 2014; Crespi et al., 2019; Crespi, Capparè, & Gherlone, 2010a, 2010b; Crespi, Capparè, Gherlone, & Romanos, 2012; Crespi, Capparè, Polizzi, & Gherlone, 2015; D'Elia et al., 2017; da Rosa, Rosa, Francischone, & Sotto-Maior, 2014; de Albornoz et al., 2014; De Bruyckere et al., 2020; De Bruyn et al., 2013; De Rouck, Collys, & Cosyn, 2008; De Rouck, Collys, Wyn, & Cosyn, 2009; de Siqueira et al., 2017; de Siqueira et al., 2020; den Hartog, Raghoebar, Stellingsma, Vissink, & Meijer, 2011; Duque, Aristizabal, Londono, Castro, & Alvarez, 2016; Eghbali et al., 2018; Ekfeldt, Eriksson, & Johansson, 2003; Esposito et al., 2017; Farrag & Khamis, 2023; Farronato et al., 2021; Farronato et al., 2020; Fenner, Hammerle, Sailer, & Jung, 2016; Fernandes, Marques, Borges, & Montero, 2023; Ferrari, Cagidiaco, Garcia-Godoy, Goracci, & Cairo, 2015; Finelle, Popelut, Knafo, & Martin, 2021; Frizzera et al., 2019; Fürhauser et al., 2017; Gallucci, Grutter, Nedir, Bischof, & Belser, 2011; Garaicoa-Pazmino et al., 2021; Ghallab et al., 2023; Girlanda et al., 2019; Givens et al., 2015; Gómez-Meda et al., 2022; Grandi, Guazzi, Samarani, & Grandi, 2013; Grassi et al., 2015; Groenendijk, Bronkhorst, & Meijer, 2021; Guarnieri, Ceccarelli, Ricci, & Testori, 2018; Guarnieri, Ceccherini, & Grande, 2015; Guarnieri, Di Nardo, Di Giorgio, Miccoli, & Testarelli, 2019; Guarnieri, Di Nardo, Gaimari, Miccoli, & Testarelli, 2019; Guarnieri et al., 2022; Hall et al., 2007; Hattingh, De Bruyn, Van Weehaeghe, Hommez, & Vandeweghe, 2020; Hof et al., 2015; Hof et al., 2014; Hollander et al., 2016; B. Hosseini et al., 2015; Humm et al., 2023; Huynh-Ba et al., 2019; Iglhaut et al., 2021; Iorio-Siciliano et al., 2016; Jacobs, Zadeh, De Kok, & Cooper, 2020; Jeffcoat, McGlumphy, Reddy, Geurs, & Proskin, 2003; Jemt, Ahlberg, Henriksson, & Bondevik, 2006; Jung et al., 2016; J. Y. Kan, Rungcharassaeng, Lozada, & Zimmerman, 2011; J. Y. K. Kan, Rungcharassaeng, & Lozada, 2003; J. Y. K. Kan, Rungcharassaeng, Sclar, & Lozada, 2007; Karoussis et al., 2004; Kobayashi et al., 2020; Koh et al., 2011; Kungsadalpipob et al., 2020; Lago, da Silva, Gude, & Rilo, 2017; C. T. Lee et al., 2020; C. T. Lee et al., 2023; Lilet et al., 2022; Lindeboom, Tjiook, & Kroon, 2006; Liu et al., 2019; Lops et al., 2015; Lops, Romeo, Chiapasco, Procopio, & Oteri, 2013; Lorenz et al., 2019; Lorenz et al., 2022; Lorenzo, Garcia, Orsini, Martin, & Sanz, 2012; Lowy et al., 2019; Mailoa et al., 2018; Malchiodi, Cucchi, Ghensi, & Nocini, 2013; Manopattanasoontorn et al., 2021; Marconcini et al., 2018; Mau et al., 2019; C. M. Meijndert et al., 2017; C. M. Meijndert, Raghoebar, Vissink, & Meijer, 2022; L. Meijndert, Raghoebar, Meijer, & Vissink, 2008; Migliorati, Amorfini, Signori, Biavati, & Benedicenti, 2015; Mizuno, Nakano, Shimomoto, Fujita, & Ishigaki, 2022; Molina, Sanz-Sánchez, Martín, Blanco, & Sanz, 2017; Muñoz-Cámara, Gilbel-Del Águila, Pardo-Zamora, & Camacho-Alonso, 2020; Nettemu et al., 2021; Nisapakultorn, Suphanantachat, Silkosessak, & Rattanamongkolgul, 2010; Oates, West, Jones, Kaiser, & Cochran, 2002; Obreja et al., 2022; Obreja et al., 2021; Oh, Ji, & Azad, 2020; Parvini, Muller, Cafferata, Schwarz, & Obreja, 2022; Parvini et al., 2023; Perez et al., 2020; Pieri, Aldini, Marchetti, & Corinaldesi, 2011, 2013; Pohl, Fürhauser, Haas, & Pohl, 2020; Proussaefs, Kan, Lozada, Kleinman, & Farnos, 2002; Puisys et al., 2022; Qian et al., 2023; F. Raes, Cosyn, Crommelinck, Coessens, & De Bruyn, 2011; F. Raes, Cosyn, & De Bruyn, 2013; S. Raes et al., 2018; S. Raes et al., 2015; Ramanauskaite et al., 2020; Ribeiro dos Reis et al., 2023; Rivara et al., 2020; Roccuzzo, Dalmasso, Pittoni, & Roccuzzo, 2019; Roccuzzo, Gaudioso, Bunino, & Dalmasso, 2014; Roccuzzo, Grasso, & Dalmasso, 2016; Romandini et al., 2021; Romanos, Malmstrom, Feng, Ercoli, & Caton, 2014; Salvi et al., 2020; Santing, Raghoebar, Vissink, den Hartog, & Meijer, 2013; Sanz Martin, Benic, Hämmerle, & Thoma, 2016; Sanz-Martín, Encalada, Sanz-Sánchez, Aracil, & Sanz, 2019; Sanz-Martin et al., 2020; Sapata et al., 2018; Schallhorn, McClain, Charles, Clem, & Newman, 2015; Schropp & Isidor, 2008; Schrott, Jimenez, Hwang, Fiorellini, & Weber, 2009; Schwarz, Sahm, & Becker, 2012; Schwarz, Schmucker, & Becker, 2017; Seyssens, Eghbali, & Cosyn, 2020; Siegenthaler et al., 2022; Slagter, Meijer, Bakker, Vissink, & Raghoebar, 2016; Slagter, Meijer, Bakker, Vissink, & Raghoebar, 2015; Slagter, Meijer, Hentenaar, Vissink, & Raghoebar, 2021; Slagter, Raghoebar, Hentenaar, Vissink, & Meijer, 2021; Small & Tarnow, 2000; Stefanini et al., 2016; Stoupel et al., 2016; Strasding et al., 2023; Sun et al., 2020; Suphanantachat, Thovanich, & Nisapakultorn, 2012; Takuma, Oishi, Manabe, Yoneda, & Nagata, 2014; Tavelli et al., 2022; Tavelli, Majzoub, et al., 2023; Tavelli, Zucchelli, et al., 2023; Thoma, Maggetti, Waller, Hammerle, & Jung, 2019; Tian et al., 2019; Todescan et al., 2023; Tsuda et al., 2011; Tur & Sarıbaş, 2023; Ueno et al., 2016; van Kesteren, Schoolfield, West, & Oates, 2010b; van Nimwegen et al., 2018; Vandeweghe, Cosyn, Thevissen, Van den Berghe, & De Bruyn, 2012; Wanis, Hosny, & ElNahass, 2022; Weber, Kim, Ng, Hwang, & Fiorellini, 2006; Wohrle, 1998; Yang, Zhou, Zhou, & Man, 2019; Yoshino, Kan, Rungcharassaeng, Roe, & Lozada, 2014; Yuenyongorarn et al., 2020; Zembic, Philipp, Hammerle, Wohlwend, & Sailer, 2015; Zhang et al., 2017; Ziebolz et al., 2017; Zitzmann, Scharer, & Marinello, 2001; Zucchelli et al., 2018; Zucchelli et al., 2013; Elise G. Zuiderveld, den Hartog, Vissink, Raghoebar, & Meijer, 2014; E. G. Zuiderveld, Meijer, den Hartog, Vissink, & Raghoebar, 2018; E. G. Zuiderveld, Meijer, Vissink, & Raghoebar, 2018; E. G. Zuiderveld et al., 2021). |

**Supplementary Table 3.** Study characteristics at baseline.

| **Study** | **Center(s), Setting, Geographic location** | **Study design** | **Patients (N), age (years), females (%), smokers, implants (N)** | **Funding (company)** |
| --- | --- | --- | --- | --- |
| Able, Sartori, Younes, & Bombarda, 2021 | 1, University, South America | Cross sectional | 52, 57.4, 76.9, NR, 355 | No |
| Adibrad, Shahabuei, & Sahabi, 2009 | 1, University, Asia | Cross sectional | 27, 63.1, 56, Yes, 66 | No |
| Anderson, Inglehart, El-Kholy, Eber, & Wang, 2014 | 1, University, North America | RCT | 6, NR, NR, No, 6 | No |
| Anderson, Inglehart, El-Kholy, Eber, & Wang, 2014 | 1, University, North America | RCT | 7, NR, NR, No, 7 | No |
| Andersson, Odman, Lindvall, & Branemark, 1998 | 1, University, Europe | nonRCT | 57, 31, 42.1, NR, 65 | No |
| Apaza-Bedoya et al., 2023 | 3, University, South America | Cross sectional | 99, 55.2, 55.6, Yes, 266 | No |
| Arora & Ivanovski, 2017 | 1, Private practice, Oceania | nonRCT | 18, NR, 83.3, Yes (if < 10 cig/day), 18 | No |
| Arora & Ivanovski, 2018 | 1, Private practice, Oceania | nonRCT | 15, 55.7, 53.3, Yes (if < 10 cig/day), 15 | No |
|  |  |  | 15, 49.2, 60, Yes (if < 10 cig/day), 15 |  |
| Arora, Khzam, Roberts, Bruce, & Ivanovski, 2017 | 1, Private practice, Oceania | nonRCT | 30, NR, 70, Yes (if < 10 cig/day), 30 | No |
| Atef, El Barbary, Dahrous, & Zahran, 2021 | 1, University, Africa | RCT | 21, 36, 76.2, No, 21 | No |
|  |  |  | 21, 36, 76.2, No, 21 |  |
| Barone et al., 2016 | 1, University, Europe | RCT | 58, 51.5, 67.2, Yes (if < 10 cig/day), 58 | No |
|  |  |  | 58, 51.3, 65.6, Yes (if < 10 cig/day), 58 |  |
| Barone, Toti, Quaranta, Derchi, & Covani, 2015 | 1, University, Europe | nonRCT | 15, 43, 53.3, Yes, 15 | No |
|  |  | nonRCT | 15, 51, 53.3, Yes, 15 |  |
| Bengazi, Wennströnm & Lekholm, 1996 | 2, University and Private Practice, Europe | nonRCT | 41, 55, 43.9, NR, 40 | NR |
|  |  |  | 41, 55, 43.9, NR, 202 |  |
| Benic et al., 2012 | 1, University, Europe | nonRCT | 14, 67.1, 42.9, NR, 14 | No |
| Benic et al., 2017 | 1, University, Europe | nonRCT | 10, 49.5, 60, NR, 10 | NR |
|  |  |  | 18, 52.2, 50, NR, 18 |  |
| Benitez Silva et al., 2022 | 1, University, South America | RCT | 27, 46.9, 40.7, Yes, 27 | Yes |
|  |  |  | 27, 45.6, 55.6, Yes, 27 |  |
| Bianchi & Sanfilippo, 2004 | 1, NR, Europe | RCT | 96, 45.4, 50, Yes, 96 | NR |
|  |  |  | 20, 45.4, 50, Yes, 20 |  |
| Bittner et al., 2020a | 1, University, North America | RCT | 16, 52.3, 43.8, No, 16 | Yes |
|  |  |  | 15, 52.3, 43.8, No, 15 |  |
| Bittner et al., 2019 and Bittner et al., 2020b | 2, University, North America | RCT | 14, 46.9, NR, NR, 14 | Yes |
|  |  |  | 11, 46.9, NR, NR, 11 |  |
| Blanes, Bernard, Blanes, & Belser, 2007 | 1, University, Europe | nonRCT | 83, 60.6, NR, Yes, 192 | NR |
| Block et al., 2009 | 1, University, North America | RCT | 29, NR, NR, NR, 29 | No |
|  |  |  | 26, NR, NR, NR, 26 |  |
| Bonino et al., 2018 | 1, University, North America | nonRCT | 24, 60.6, NR, NR, 28 | No |
| Bragger, Hammerle, & Lang, 1996 | 1, University, Europe | nonRCT | 15, NR, NR, NR, 20 | Foundation |
|  |  |  | 6, NR, NR, NR, 8 |  |
|  |  |  | 20, NR, NR, NR, 20 |  |
| Bressan et al., 2017 | 4, Private practice, Europe | RCT | 33, 55.6, 58, Yes, 33 | Yes |
|  |  |  | 39, 57.6, 60, Yes, 39 |  |
| Brunello et al., 2022 | 1, University, Europe | nonRCT | 48, 47.6, 64.6, NR, 48 | No |
| Burkhardt, Joss, & Lang, 2008 | 1, Private practice, Europe | nonRCT | 10, NR, 60, No, 10 | No |
| Buser et al., 2009 | 1, University, Europe | nonRCT | 20, 41.7, 75, Yes (if < 10 cig/day), 20 | Yes |
| Buser et al., 2011 | 1, University, Europe | nonRCT | 20, 41.7, 75, Yes (if < 10 cig/day), 20 | Yes |
| Buser, Chappuis, Bornstein, et al., 2013 | 1, University, Europe | nonRCT | 41, 38.8, 39, Yes, 41 | No |
| Buser, Chappuis, Kuchler, et al., 2013 | 1, University, Europe | nonRCT | 20, 41.7, 75, Yes (if < 10 cig/day), 20 | Yes |
| Bushahri et al., 2021 | 1, University, North America | RCT | 16, 60.4, NR, No, 16 | Yes |
|  |  |  | 12, 57.9, NR, No, 12 |  |
| Cabello, Rioboo, & Fabrega, 2013 | 2, Private practice, Europe | nonRCT | 14, 52.3, 50, Yes, 14 | No |
| Canullo, Caneva, & Tallarico, 2017 | 2, Private practice, Europe | RCT | 10, NR, NR, Yes, 10 | NR |
|  |  |  | 9, NR, NR, Yes, 9 |  |
| Canullo, Iurlaro, & Iannello, 2009 | 2, Private practice, Europe | RCT | 11, 50, 36.4, Yes, 11 | No |
|  |  |  | 11, 50, 54.5, Yes, 11 |  |
| Cardaropoli, Gaveglio, Gherlone, & Cardaropoli, 2014 | 1, Private practice, Europe | RCT | 26, 42, NR, Yes, 26 | NR |
|  |  |  | 26, 44, NR, Yes, 26 |  |
| Cecchinato, Lops, Salvi, & Sanz, 2015 | 3, University, Europe | RCT | 43, 51, NR, Yes, 43 | NR |
|  |  |  | 45, 51, NR, Yes, 45 |  |
| Chan et al., 2019 | 1, University, North America | RCT | 18, 60.4, NR, No, 18 | Yes |
|  |  |  | 20, 57.9, NR, No, 20 |  |
| Chang, Wennstrom, Odman, & Andersson, 1999 | 1, University, Europe | Cross sectional | 20, NR, NR, NR, 20 | No |
| Chen, Darby, & Reynolds, 2007 | 1, University, Oceania | nonRCT | 10, 50.4, 60, Yes, 10 | NR |
|  |  |  | 10, 43.2, 70, Yes, 10 |  |
|  |  |  | 10, 42.1, 70, Yes, 10 |  |
| Chung, Rungcharassaeng, Kan, Roe, & Lozada, 2011 | 1, University, North America | nonRCT | 10, 52.1, 40, No, 10 | Yes |
| Clem et al., 2023 | 5, Private practice, North America | RCT | 29, 57.76, 60.6, Yes, 29 | Yes |
|  |  |  | 28, 53.16, 58.1, Yes, 28 |  |
| Cooper et al., 2010 | 4, University, Europe and North America | nonRCT | 55, 45.1, 60, No, 55 | Yes |
|  |  |  | 60, 42.1, 58.3, No, 60 |  |
| Cooper et al., 2015 | 4, University and Private Practice, North America | RCT | 48, 43, 48, No, 53 | Yes |
|  |  |  | 49, 36, 71, No, 53 |  |
|  |  |  | 44, 46, 50, No, 50 |  |
| Cordaro, Torsello, & Roccuzzo, 2009 | 2, Private practice, Europe | RCT | 14, NR, NR, NR, 14 | Foundation |
|  |  |  | 14, NR, NR, NR, 14 |  |
| Cosyn & De Rouck, 2009 | 1, University, Europe | Cross sectional | 27, 50, 66.7, No, 27 | No |
| Cosyn et al., 2011 | 1, University, Europe | nonRCT | 28, 54, NR, No, 28 | No |
| Cosyn, Sabzevar, & De Bruyn, 2012 | 1, University, Europe | Cross sectional | 97, 51, 61.9, NR, 97 | No |
| Cosyn, De Bruyn, & Cleymaet, 2013 | 1, Private practice, Europe | nonRCT | 22, 50, 45.5, No, 22 | No |
| Cosyn, Pollaris, Van der Linden, & De Bruyn, 2015 | 1, University, Europe | nonRCT | 39, NR, NR, NR, 39 | Yes |
|  |  |  | 8, NR, NR, NR, 8 |  |
| Cosyn et al., 2016 | 1, Private practice, Europe | nonRCT | 15, 50, NR, No, 15 | No |
|  |  |  | 7, 50, NR, No, 7 |  |
| Cosyn et al., 2022 | 6, NR, Europe | RCT | 30, 50.1, 50, No, 30 | Foundation |
|  |  |  | 30, 48.2, 53, No, 30 |  |
| Covani, Canullo, Toti, Alfonsi, & Barone, 2014 | 1, University, Europe | nonRCT | 45, 42.8, 57.8, Yes, 45 | No |
| Crespi, Cappare, & Gherlone, 2010a | 1, University, Europe | nonRCT | 29, 49.5, NR, Yes, 164 | No |
| Crespi, Cappare, & Gherlone, 2010b | 1, University, Europe | RCT | 15, 51.2, NR, Yes, 15 | NR |
|  |  |  | 15, 51.2, NR, Yes, 15 |  |
| Crespi, Cappare, Gherlone, & Romanos, 2012 | 1, University, Europe | nonRCT | 15, NR, 66.7, Yes (if < 10 cig/day), 20 | No |
| Crespi, Cappare, Polizzi, & Gherlone, 2015 | 1, University, Europe | nonRCT | NR, NR, NR, Yes (if < 10 cig/day), 47 | NR |
|  |  |  | NR, NR, NR, Yes (if < 10 cig/day), 47 |  |
| Crespi et al., 2019 | 1, University, Europe | RCT | 21, 52.2, NR, Yes, 61 | NR |
|  |  |  | 21, 52.2, NR, Yes, 62 |  |
| D'Elia et al., 2017 | 1, University, Europe | RCT | 15, 47.5, 50, Yes (if ≤ 10 cig/day), 15 | Yes |
|  |  |  | 15, 47.5, 50, Yes (if ≤ 10 cig/day), 15 |  |
| da Rosa, Rosa, Francischone, & Sotto-Maior, 2014 | 1, Private practice, South America | nonRCT | 18, NR, 44.4, No, 18 | No |
| de Albornoz et al., 2014 | 1, University, Europe | RCT | 11, 51.6, 81.2, NR, 11 | Yes |
|  |  |  | 14, 51.8, 42.9, NR, 14 |  |
| De Bruyn et al., 2013 | 4, University, Europe and North America | nonRCT | 46, NR, NR, No, 46 | Yes |
|  |  |  | 51, NR, NR, No, 51 |  |
| De Bruyckere et al., 2020 | 2, Private practice, Europe | RCT | 21, 48, 42.9, No, 21 | No |
|  |  |  | 21, 51, 52.4, No, 21 |  |
| De Rouck, Collys, & Cosyn, 2008 | 1, University, Europe | nonRCT | 32, 54, NR, Yes (if < 10 cig/day), 32 | No |
| De Rouck, Collys, Wyn, & Cosyn, 2009 | 1, University, Europe | RCT | 24, 55, 54.1, Yes, 24 | NR |
|  |  |  | 25, 52, 52, Yes, 25 |  |
| de Siqueira et al., 2017 | 1, University, South America | RCT | 11, NR, NR, No, 28 | No |
|  |  |  | 11, NR, NR, No, 27 |  |
| de Siqueira et al., 2020 | 1, University, South America | RCT | 11, NR, NR, No, 28 | No |
|  |  |  | 11, NR, NR, No, 27 |  |
| Degidi, Nardi, Daprile, & Piattelli, 2014 | 1, Private practice, Europe | RCT | 29, 37.7, NR, Yes, 29 | NR |
|  |  |  | 24, 40.1, NR, Yes, 24 |  |
| den Hartog, Raghoebar, Stellingsma, Vissink, & Meijer, 2011 | 1, University, Europe | RCT | 31, 38.4, 71, No, 31 | Yes |
|  |  |  | 31, 40.1, 45.2, No, 31 |  |
| Duque, Aristizabal, Londono, Castro, & Alvarez, 2016 | 1, University, South America | Cross sectional | 25, 54, 72, No, 64 | No |
| Eghbali et al., 2018 | 1, University, Europe | nonRCT | 37, 38, 48.6, No, 37 | No |
| Ekfeldt, Eriksson, & Johansson, 2003 | 1, University, Europe | nonRCT | 10, 66, 30, NR, 58 | NR |
|  |  |  | 10, 67, 40, NR, 49 |  |
| Esposito et al., 2017 | 4, Private practice, Europe | RCT | 40, 57.6, 60, Yes, 40 | Yes |
|  |  |  | 40, 55.6, 58, Yes, 40 |  |
| Farrag & Khamis, 2023 | 1, University, Africa | nonRCT | 28, 38.5, 64.3, Yes (if < 10 cig/day), 28 | No |
|  |  |  | 28, 38.5, 64.3, Yes (if < 10 cig/day), 28 |  |
| Farronato et al., 2020 | 1, University, Europe | nonRCT | NR, NR, NR, Yes, 14 | No |
|  |  |  | NR, NR, NR, Yes, 64 |  |
| Farronato et al., 2021 | 1, University, Europe | RCT | 38, NR, 65.8, Yes, 38 | No |
|  |  |  | 39, NR, 66.7, Yes, 39 |  |
| Fenner, Hammerle, Sailer, & Jung, 2016 | 1, University, Europe | nonRCT | 13, NR, NR, Yes, 13 | No |
|  |  |  | 15, NR, NR, Yes, 15 |  |
| Fernandes, Marques, Borges, & Montero, 2023 | 1, University, Europe | RCT | 16, 51.3, 50, No, 16 | No |
|  |  |  | 16, 44.1, 81, No, 16 |  |
| Ferrari, Cagidiaco, Garcia-Godoy, Goracci, & Cairo, 2015 | 1, University, Europe | RCT | 15, NR, NR, Yes (if < 10 cig/day), 27 | NR |
|  |  |  | 18, NR, NR, Yes (if < 10 cig/day), 42 |  |
|  |  |  | 14, NR, NR, Yes (if < 10 cig/day), 28 |  |
| Finelle, Popelut, Knafo, & Sanz Martin, 2021 | NR, NR, Europe | nonRCT | 17, 58.7, 41.2, Yes (if < 10 cig/day), 17 | No |
| Frizzera et al., 2019 | 1, University, South America | RCT | 8, NR, NR, No, 8 | NR |
|  |  |  | 8, NR, NR, No, 8 |  |
|  |  |  | 8, NR, NR, No, 8 |  |
| Furhauser et al., 2017 | 1, University, Europe | nonRCT | 77, 48.8, 59.7, NR, 77 | NR |
| Gallucci, Grutter, Nedir, Bischof, & Belser, 2011 | 1, University, Europe | RCT | 10, NR, NR, Yes (if < 10 cig/day), 10 | Foundation |
|  |  |  | 10, NR, NR, Yes (if < 10 cig/day), 10 |  |
| Garaicoa-Pazmino et al., 2021 | 1, University, North America | nonRCT | 13, 56.54, 61.5, No, 13 | Foundation |
|  |  |  | 13, 56.54, 61.5, No, 13 |  |
| Ghallab et al., 2023 | 1, Private practice, Africa | RCT | 15, 37.4, 66, No, 15 | No |
|  |  |  | 15, 43.07, 80, No, 15 |  |
| Girlanda et al., 2019 | 1, University, South America | RCT | 11, NR, 63.6, No, 11 | NR |
|  |  |  | 11, NR, 63.6, No, 11 |  |
| Gomez-Meda et al., 2022 | NR, NR, Europe | nonRCT | 15, 56.8, 66.7, Yes, 20 | No |
|  |  |  | 15, 56.2, 66.7, Yes, 6 |  |
| Grandi, Guazzi, Samarani, & Grandi, 2013 | 2, Private practice, Europe | nonRCT | 23, 54.7, 64, Yes, 23 | No |
|  |  |  | 24, 59, 52, Yes, 24 |  |
| Grassi et al., 2015 | 2, University and Private Practice, Europe | nonRCT | 17, 52.3, 52.9, Yes, 32 | No |
| Groenendijk, Bronkhorst, & Meijer, 2021 | NR, NR, Europe | nonRCT | 97, 46, 57.7, Yes (if < 10 cig/day), 97 | Yes |
| Guarnieri, Ceccherini, & Grande, 2015 | 1, Private practice, Europe | nonRCT | 20, 34, 35, No, 20 | NR |
| Guarnieri, Ceccarelli, Ricci, & Testori, 2018 | 1, Private practice, Europe | nonRCT | 16, NR, NR, NR, 16 | NR |
|  |  |  | 16, NR, NR, NR, 16 |  |
| Guarnieri, Di Nardo, Di Giorgio, Miccoli, & Testarelli, 2019 | 1, University, Europe | RCT | 20, 49.7, 40, No, 20 | Yes |
|  |  |  | 20, 49.7, 40, No, 20 |  |
| Guarnieri, Di Nardo, Gaimari, Miccoli, & Testarelli, 2019 | NR, NR, Europe | nonRCT | 28, 51, 50, Yes (if < 10 cig/day), 28 | Yes |
|  |  |  | 28, 51, 50, Yes (if < 10 cig/day), 28 |  |
| Guarnieri et al., 2022 | 1, University, Europe | RCT | 20, 49.7, 40, No, 20 | Yes |
|  |  |  | 20, 49.7, 40, No, 20 |  |
| Hall et al., 2007 | 1, University, Oceania | RCT | 14, NR, NR, Yes, 14 | Yes |
|  |  |  | 14, NR, NR, Yes, 14 |  |
| Hattingh, De Bruyn, Van Weehaeghe, Hommez, & Vandeweghe, 2020 | 1, University, Europe | nonRCT | 27, 61, 25.9, NR, 27 | No |
| Hof et al., 2014 | 1, University, Europe | Cross sectional | 43, 41, 34.9, NR, 43 | No |
| Hof et al., 2015 | 1, University, Europe | nonRCT | 15, NR, NR, Yes, 15 | No |
|  |  |  | 13, NR, NR, Yes, 13 |  |
|  |  |  | 26, NR, NR, Yes, 26 |  |
|  |  |  | 35, NR, NR, Yes, 35 |  |
|  |  |  | 64, NR, NR, Yes, 64 |  |
| Hollander et al., 2016 | NR, NR, Europe | nonRCT | 38, 56.2, 52.6, NR, 106 | No |
| Hosseini et al., 2015 | 1, University, North America | RCT | 10, NR, NR, Yes, 10 | Yes |
|  |  |  | 10, NR, NR, Yes, 10 |  |
| Humm et al., 2023 | 1, University, Europe | RCT | 15, NR, NR, NR, 13 | No |
|  |  |  | 15, NR, NR, NR, 8 |  |
| Huynh-Ba et al., 2019 | 1, University, North America | RCT | 20, 52.6, 45, Yes, 20 | Foundation |
|  |  |  | 15, 51.8, 53.3, Yes, 15 |  |
| Iglhaut et al., 2021 | 2, Private practice, Europe | Cross sectional | 40, 61.5, 50, Yes, 40 | No |
| Iorio-Siciliano et al., 2016 | 1, University, Europe | nonRCT | 20, 46.5, 65, Yes (if < 10 cig/day), 20 | No |
| Jacobs, Zadeh, De Kok, & Cooper, 2020 | 2, University, North America | RCT | 19, 53, 57.9, No, 19 | Yes |
|  |  |  | 14, 63, 57.1, No, 14 |  |
| Jeffcoat, McGlumphy, Reddy, Geurs, & Proskin, 2003 | 2, University, North America | RCT | 40, NR, NR, No, 40 | Yes |
|  |  |  | 40, NR, NR, No, 40 |  |
|  |  |  | 40, NR, NR, No, 40 |  |
| Jemt, Ahlberg, Henriksson, & Bondevik, 2006 | 1, University, Europe | nonRCT | 23, 26.1, 26.1, NR, 47 | NR |
| Jung et al., 2016 | 2, University, Europe | nonRCT | 60, NR, 50, Yes (if < 10 cig/day), 71 | Yes |
| Kan, Rungcharassaeng, & Lozada, 2003 | 1, University, North America | nonRCT | 35, 36.5, NR, No, 35 | Yes |
| Kan, Rungcharassaeng, Sclar, & Lozada, 2007 | 1, University, North America | nonRCT | 23, 39.5, NR, NR, 23 | NR |
| Kan, Rungcharassaeng, Lozada, & Zimmerman, 2011 | 1, University, North America | nonRCT | 35, 36.8, NR, No, 35 | Yes |
| Karoussis et al., 2004 | 1, University, Europe | nonRCT | 89, 49.3, 61.8, Yes, 179 | Foundation |
| Kobayashi et al., 2020 | 1, University, Asia | nonRCT | 14, 50, 35.7, No, 14 | No |
|  |  |  | 12, 47.1, 58.3, No, 12 | No |
| Koh et al., 2011 | 1, University, North America | RCT | 10, 54.1, 20, No, 10 | Yes |
|  |  |  | 10, 56.8, 60, No, 10 |  |
| Kungsadalpipob et al., 2020 | 1, University, Asia | Cross sectional | 200, 57.3, 58.5, Yes, 412 | No |
| Lago, da Silva, Gude, & Rilo, 2017 | 1, University, Europe | nonRCT | 35, , 42.9, Yes (if < 10 cig/day), 67 | No |
| Lee et al., 2020 | 1, University, North America | RCT | 18, 49.22, NR, NR, 18 | Yes |
|  |  |  | 21, 49.22, NR, NR, 21 |  |
| Lee et al., 2023 | 1, University, North America | RCT | 15, 61.5, 47, Yes (if < 10 cig/day), 15 | Foundation |
|  |  |  | 15, 63.5, 53, Yes (if < 10 cig/day), 15 |  |
|  |  |  | 16, 58.9, 56, Yes (if < 10 cig/day), 16 |  |
| Lilet et al., 2022 | 1, University, Europe | nonRCT | 20, 51.4, 51.4, No, 20 | Yes |
| Lindeboom, Tjiook, & Kroon, 2006 | 1, University, Europe | RCT | 23, 39.9, NR, No, 23 | No |
|  |  |  | 25, 39.5, NR, No, 25 |  |
| Liu et al., 2019 | 1, University, Asia | nonRCT | 45, 36.7, 53.3, No, 45 | No |
| Lops, Romeo, Chiapasco, Procopio, & Oteri, 2013 | 2, University, Europe | nonRCT | 21, 42, 47.6, Yes (if < 10 cig/day), 21 | NR |
| Lops et al., 2015 | 2, University, Europe | nonRCT | 13, NR, NR, Yes (if < 15 cig/day), 13 | NR |
|  |  |  | 23, NR, NR, Yes (if < 15 cig/day), 23 |  |
|  |  |  | 20, NR, NR, Yes (if < 15 cig/day), 20 |  |
|  |  |  | 16, 63.5, NR, Yes (if < 15 cig/day), 16 |  |
| Lorenz et al., 2019 | 2, University and Private Practice, Europe | nonRCT | 28, 54, 53.6, NR, 83 | No |
| Lorenz et al., 2022 | 3, University and Private Practice, Europe | nonRCT | 19, NR, 68.4, Yes, 24 | Yes |
| Lorenzo, Garcia, Orsini, Martin, & Sanz, 2012 | 1, University, Europe | RCT | 12, 63, 66.7, Yes (if < 10 cig/day), 12 | Yes |
|  |  |  | 12, 62, 83.3, Yes (if < 10 cig/day), 12 |  |
| Lowy et al., 2019 | NR, Private practice, North America | RCT | 12, NR, NR, No, 12 | Yes |
|  |  |  | 12, NR, NR, No, 12 |  |
| Mailoa et al., 2018 | 1, University, North America | Cross sectional | 14, 67.5, 50, No, 14 | No |
| Malchiodi, Cucchi, Ghensi, & Nocini, 2013 | 1, University, Europe | nonRCT | 58, 39.9, 44.8, Yes (if < 20 cig/day), 64 | No |
| Manopattanasoontorn et al., 2021 | 1, University, Asia | Cross sectional | 165, 58.9, 57, Yes, 331 | No |
| Marconcini et al., 2018 | 1, University, Europe | RCT | 58, 51.5, 67.2, Yes (if < 10 cig/day), 58 | No |
|  |  |  | 58, 51.3, 65.6, Yes (if < 10 cig/day), 58 |  |
| Mau et al., 2019 | 2, University, Asia and North America | RCT | 24, 40.9, 70.8, Yes, 24 | Foundation |
|  |  |  | 24, 42.8, 54.2, Yes, 24 |  |
| Meijndert, Raghoebar, Meijer, & Vissink, 2008 | 1, University, Europe | RCT | 31, 33.3, 58.1, No, 31 | Yes |
|  |  |  | 31, 34.6, 48.4, No, 31 |  |
|  |  |  | 31, 32.2, 51.6, No, 31 |  |
| Meijndert et al., 2017 | 1, University, Europe | RCT | 26, NR, NR, No, 26 | Yes |
|  |  |  | 18, NR, NR, No, 18 |  |
|  |  |  | 17, NR, NR, No, 17 |  |
| Meijndert, Raghoebar, Vissink, & Meijer, 2022 | 1, University, Europe | nonRCT | 30, 38, 50, No, 30 | Yes |
| Migliorati, Amorfini, Signori, Biavati, & Benedicenti, 2015 | 1, University, Europe | RCT | 24, NR, NR, Yes (if < 10 cig/day), 24 | NR |
|  |  |  | 23, NR, NR, Yes (if < 10 cig/day), 23 |  |
| Mizuno, Nakano, Shimomoto, Fujita, & Ishigaki, 2022 | 1, University, Asia | nonRCT | 20, 60, 65, No, 20 | No |
| Molina, Sanz-Sanchez, Martin, Blanco, & Sanz, 2017 | 1, University, Europe | RCT | 20, 51.62, 58.8, Yes (if < 10 cig/day), 30 | Yes |
|  |  |  | 16, 52.61, 41.2, Yes (if < 10 cig/day), 26 |  |
| Munoz-Camara, Gilbel-Del Aguila, Pardo-Zamora, & Camacho-Alonso, 2020 | 2, University and Private Practice, Europe | nonRCT | 50, 48.8, 52, Yes, 50 | No |
|  |  |  | 50, 47.6, 58, Yes, 50 |  |
| Nettemu et al., 2021 | 1, University, Asia | Cross sectional | 80, 56.4, 52.5, Yes, 714 | No |
| Nisapakultorn, Suphanantachat, Silkosessak, & Rattanamongkolgul, 2010 | 1, University, Asia | Cross sectional | 40, 45.2, 55, NR, 40 | No |
| Oates, West, Jones, Kaiser, & Cochran, 2002 | 1, University, North America | nonRCT | 39, NR, 55, Yes (if < 10 cig/day), 106 | Yes |
| Obreja et al., 2021 | 1, University, Europe | Cross sectional | 19, 46.2, 55.6, No, 29 | No |
|  |  |  | 36, 62.2, 68.4, No, 55 |  |
| Obreja et al., 2022 | 1, University, Europe | Cross sectional | 44, 55.6, 63.6, NR, 57 | No |
| Oh, Ji, & Azad, 2020 | 1, University, North America | RCT | 11, 65.3, 81.8, No, 18 | No |
|  |  |  | 7, 66, 57.1, No, 8 |  |
| Parvini et al., 2023 | 1, University, Europe | Cross sectional | 27, 47.8, 26.9, Yes, 27 | Foundation |
|  |  |  | 25, 58.6, 9.6, Yes, 25 |  |
| Parvini, Muller, Cafferata, Schwarz, & Obreja, 2022 | 1, University, Europe | nonRCT | 14, 58.7, NR, Yes, 16 | No |
|  |  |  | 11, 58.7, NR, Yes, 16 |  |
| Perez et al., 2020 | 1, University, Europe | RCT | 18, 50.8, 38.9, Yes, 18 | Yes |
|  |  |  | 18, 59, 55.6, Yes, 18 |  |
| Pieri, Aldini, Marchetti, & Corinaldesi, 2011 | 1, University, Europe | RCT | 19, 45.8, 63.2, Yes, 19 | NR |
|  |  |  | 19, 46.6, 57.9, Yes, 19 |  |
| Pieri, Aldini, Marchetti, & Corinaldesi, 2013 | NR, NR, Europe | nonRCT | 29, 45.2, 62.1, Yes (if < 10 cig/day), 29 | NR |
| Pohl, Furhauser, Haas, & Pohl, 2020 | 1, University, Europe | nonRCT | 12, NR, 66.7, NR, 12 | No |
|  |  |  | 12, NR, NR, NR, 12 |  |
| Proussaefs, Kan, Lozada, Kleinman, & Farnos, 2002 | 1, University, North America | nonRCT | 10, 45, NR, No, 10 | Yes |
| Puisys et al., 2022 | 1, Private practice, Europe | RCT | 25, 45.8, 60, Yes (if < 10 cig/day), 25 | Yes |
|  |  |  | 25, 41.2, 52, Yes (if < 10 cig/day), 25 |  |
| Qian et al., 2023 | 1, University, Asia | nonRCT | 12, 34.8, 25, Yes (if < 10 cig/day), 12 | No |
| Raes, Cosyn, Crommelinck, Coessens, & De Bruyn, 2011 | 1, University, Europe | nonRCT | 23, 45, 47.8, No, 23 | Yes |
|  |  |  | 16, 40, 37.5, No, 16 |  |
| Raes, Cosyn, & De Bruyn, 2013 | 1, University, Europe | nonRCT | 23, 32, 48, No, 23 | Yes |
|  |  |  | 16, 49, 38, No, 16 |  |
|  |  |  | 9, 45, 44, No, 9 |  |
| Raes et al., 2015 | 3, University and Private Practice, Europe and North America | nonRCT | 46, 42, 47.8, Yes (≥ 10 cig/day), 46 | Yes |
|  |  |  | 39, 44, 53.8, No, 39 |  |
| Raes et al., 2018 | 1, University, Europe | nonRCT | 11, NR, 54.5, No, 11 | Yes |
|  |  |  | 18, NR, 61.1, No, 18 |  |
| Ramanauskaite et al., 2020 | 1, University, Europe | Cross sectional | 121, 65.2, 57.9, Yes, 283 | No |
|  |  |  | 111, 64.8, 57.7, Yes, 291 |  |
| Ribeiro dos Reis et al., 2023 | 1, University, South America | nonRCT | 7, 32.3, 71.4, NR, 7 | Yes |
|  |  |  | 8, 32.1, 62.5, NR, 8 |  |
| Rivara et al., 2020 | 1, University, Europe | RCT | 15, NR, NR, Yes (if < 10 cig/day), 30 | Yes |
|  |  |  | 15, NR, NR, Yes (if < 10 cig/day), 30 |  |
| Roccuzzo, Gaudioso, Bunino, & Dalmasso, 2014 | 1, Private practice, Europe | nonRCT | 16, 53.1, 81.3, Yes, 16 | No |
| Roccuzzo, Grasso, & Dalmasso, 2016 | 1, Private practice, Europe | nonRCT | 63, 52.2, 58.7, Yes, 63 | No |
|  |  |  | 24, 52.8, 65.7, Yes, 24 |  |
|  |  |  | 11, 52.8, 65.7, Yes, 11 |  |
| Roccuzzo, Dalmasso, Pittoni, & Roccuzzo, 2019 | 1, Private practice, Europe | nonRCT | 13, NR, NR, Yes, 13 | No |
| Romandini et al., 2021 | 1, University, Europe | Cross sectional | 92, 64.2, 60.9, Yes, 272 | No |
| Romanos, Malmstrom, Feng, Ercoli, & Caton, 2014 | 1, University, North America | RCT | 17, NR, NR, NR, 52 | NR |
|  |  |  | 17, NR, NR, NR, 52 |  |
| Salvi et al., 2020 | 1, University, Europe | RCT | 19, 59, 50, Yes (if < 10 cig/day), 19 | Yes |
|  |  |  | 19, 56, 61.9, Yes (if < 10 cig/day), 19 |  |
| Santing, Raghoebar, Vissink, den Hartog, & Meijer, 2013 | 1, University, Europe | nonRCT | 60, 36.9, 51.7, No, 60 | Yes |
| Sanz Martin, Benic, Hammerle, & Thoma, 2016 | 1, University, Europe | RCT | 15, NR, NR, NR, 15 | No |
|  |  |  | 18, NR, NR, NR, 18 |  |
| Sanz-Martin et al., 2020 | 1, Private practice, Europe | Cross sectional | 104, 67.3, NR, Yes, NR | No |
| Sanz-Martin, Encalada, Sanz-Sanchez, Aracil, & Sanz, 2019 | 1, University, Europe | nonRCT | 12, 58, 75, Yes (if < 10 cig/day), 12 | Yes |
| Sapata et al., 2018 | 1, University, Europe | RCT | 17, NR, NR, NR, 17 | No |
|  |  |  | 16, NR, NR, NR, 16 |  |
| Schallhorn, McClain, Charles, Clem, & Newman, 2015 | 3, Private practice, North America | nonRCT | 30, NR, NR, No, 35 | Yes |
| Schropp & Isidor, 2008 | 1, University, Europe | RCT | 18, 47, NR, NR, 18 | NR |
|  |  |  | 16, 47, NR, NR, 16 |  |
| Schrott, Jimenez, Hwang, Fiorellini, & Weber, 2009 | 5, University and Private Practice, Europe and North America | nonRCT | 58, 58, 52, Yes, 346 | Yes |
|  |  |  | 58, 58, 52, Yes, 40 |  |
| Schwarz, Sahm, & Becker, 2012 | 1, University, Europe | RCT | 8, 44, 25, No, 8 | Yes |
|  |  |  | 8, 59.4, 25, No, 8 |  |
|  |  |  | 8, 50.5, 25, No, 8 |  |
| Schwarz, Schmucker, & Becker, 2017 | 1, University, Europe | RCT | 10, 51.5, 80, No, 10 | Yes |
|  |  |  | 9, 44, 77.8, No, 9 |  |
| Seyssens, Eghbali, & Cosyn, 2020 | 1, Private practice, Europe | nonRCT | 12, 50, NR, No, 12 | No |
|  |  |  | 6, 50, NR, No, 6 |  |
| Siegenthaler et al., 2022 | 1, University, Europe | RCT | 15, 59.2, 27, Yes (if < 15 cig/day), 15 | Yes |
|  |  |  | 16, 60.8, 38, Yes (if < 15 cig/day), 16 |  |
|  |  |  | 16, 60.9, 63, Yes (if < 15 cig/day), 16 |  |
| Slagter, Meijer, Bakker, Vissink, & Raghoebar, 2015 | 1, University, Europe | RCT | 20, 39.4, 75, NR, 20 | Yes |
|  |  |  | 20, 42.3, 60, NR, 20 |  |
| Slagter, Meijer, Bakker, Vissink, & Raghoebar, 2016 | 1, University, Europe | RCT | 20, 43.7, 10, No, 20 | Yes |
|  |  |  | 20, 48.6, 10, No, 20 |  |
| Slagter, Meijer, Hentenaar, Vissink, & Raghoebar, 2021 | 1, University, Europe | RCT | 17, 48.6, NR, NR, 17 | Yes |
|  |  |  | 18, 43.7, NR, NR, 18 |  |
|  |  |  | 18, 39.4, 72.2, NR, 18 |  |
|  |  |  | 17, 42.3, 52.9, NR, 17 |  |
| Small & Tarnow, 2000 | 1, University, North America | nonRCT | 11, , NR, Yes (if < 10 cig/day), 65 | No |
| Stefanini et al., 2016 | 1, University, Europe | nonRCT | 20, NR, 60, No, 20 | NR |
| Stoupel et al., 2016 | 1, University, North America | RCT | 18, 54, 55.6, Yes, 18 | Yes |
|  |  |  | 20, 46, 70, Yes, 20 |  |
| Strasding et al., 2023 | 5, University and Private Practice, Europe | RCT | 30, NR, NR, Yes (if < 10 cig/day), 30 | Yes |
|  |  |  | 30, 47.3, NR, Yes (if < 10 cig/day), 30 |  |
| Sun et al., 2020 | 1, University, Asia | RCT | 15, 6, 26.7, Yes, 15 | NR |
|  |  |  | 15, 6, 20, Yes, 15 |  |
| Suphanantachat, Thovanich, & Nisapakultorn, 2012 | 1, University, Asia | Cross sectional | 40, 45.2, 55, NR, 40 | No |
| Takuma, Oishi, Manabe, Yoneda, & Nagata, 2014 | 2, University, Asia | nonRCT | 30, 34.6, 86.7, No, 66 | NR |
| Tavelli et al., 2022 | 1, University, North America | Cross sectional | 158, 59.5, 46.2, Yes, 176 | No |
| Tavelli, Majzoub, et al., 2023 | 1, University, North America | RCT | 14, 46.9, 57.1, No, 14 | Foundation |
| Tavelli, Majzoub, et al., 2023 | 1, University, North America | RCT | 14, 47.1, 57.1, No, 14 | Foundation |
| Tavelli, Zucchelli, et al., 2023 | 1, University, North America | nonRCT | 10, 52.8, 70, No, 10 | No |
| Thoma, Maggetti, Waller, Hammerle, & Jung, 2019 | 1, University, Europe | Cross sectional | 19, 51.9, 31.6, Yes, 27 | No |
|  |  |  | 19, 71.1, 73.7, No, 39 |  |
| Tian et al., 2019 | 1, University, Asia | nonRCT | 27, 35, NR, No, 27 | No |
| Todescan et al., 2023 | 1, University, South America | nonRCT | 20, 35, 57.7, Yes (if < 10 cig/day), 20 | Yes |
|  |  |  | 6, 48, 57.7, Yes (if < 10 cig/day), 6 |  |
| Tsuda et al., 2011 | 1, University, North America | nonRCT | 10, 55, 60, No, 10 | Yes |
| Tur & Saribas, 2023 | 1, University, Asia | Cross sectional | 60, NR, 52.87, Yes, 202 | NR |
| Ueno et al., 2016 | 1, University, Asia | Cross sectional | 60, 60.7, 61.2, Yes, 90 | NR |
| van Kesteren, Schoolfield, West, & Oates, 2010 | NR, NR, North America | RCT | 13, NR, NR, Yes, 13 | Yes (Foundation and company) |
|  |  |  | 13, NR, NR, Yes, 13 |  |
| van Nimwegen et al., 2018 | 1, University, Europe | RCT | 30, 47.8, 50, No, 30 | Yes |
|  |  |  | 30, 45.5, 56.7, No, 30 |  |
| Vandeweghe, Cosyn, Thevissen, Van den Berghe, & De Bruyn, 2012 | 1, University, Europe | nonRCT | 14, NR, 57.1, NR, 15 | Yes |
| Wanis, Hosny, & ElNahass, 2022 | 1, University, Africa | RCT | 12, 34.3, 63.4, No, 12 | No |
|  |  |  | 12, 30.3, 70, No, 12 |  |
| Weber, Kim, Ng, Hwang, & Fiorellini, 2006 | 5, University and Private Practice, North America | nonRCT | 80 (in total), NR, NR, NR, 93 | NR |
|  |  |  | 80 (in total), NR, NR, NR, 59 |  |
| Wohrle, 1998 | 1, Private practice, North America | nonRCT | 14, NR, NR, NR, 14 | NR |
| Yang, Zhou, Zhou, & Man, 2019 | 1, University, Asia | nonRCT | 16, NR, NR, Yes (if < 10 cig/day), 16 | No |
|  |  |  | 14, NR, NR, Yes (if < 10 cig/day), 20 |  |
|  |  |  | 14, NR, NR, Yes (if < 10 cig/day), 14 |  |
| Yoshino, Kan, Rungcharassaeng, Roe, & Lozada, 2014 | 1, University, North America | RCT | 10, 52.6, NR, No, 10 | Yes |
| Yuenyongorarn et al., 2020 | 1, University, North America | RCT | 10, NR, NR, No, 10 | No |
|  |  |  | 10, NR, NR, No, 10 |  |
| Zembic, Philipp, Hammerle, Wohlwend, & Sailer, 2015 | NR, University, Europe | nonRCT | 16, 36, 56.2, NR, 31 | NR |
| Zhang et al., 2017 | 1, University, Asia | nonRCT | 16, 36, 56.2, Yes (if < 10 cig/day), 16 | No |
| Ziebolz et al., 2017 | 1, University, Europe | RCT | 17, 51.9, 52.9, No, 24 | No |
|  |  |  | 15, 57.1, 46.7, No, 26 |  |
|  |  |  | 16, 56, 37.5, No, 30 |  |
|  |  |  | 14, 56.4, 35.7, No, 21 |  |
| Zitzmann, Scharer, & Marinello, 2001 | 1, University, Europe | RCT | 75, 56.1, 74.6, NR, 112 | NR |
|  |  |  | 75, 56.1, 74.6, NR, 41 |  |
|  |  |  | 75, 56.1, 74.6, NR, 112 |  |
| Zucchelli et al., 2013 | 1, University, Europe | nonRCT | 20, NR, 70, Yes (if < 10 cig/day), 20 | No |
| Zucchelli et al., 2018 | 1, University, Europe | nonRCT | 19, NR, 70, Yes (if < 10 cig/day), 19 | No |
| Zuiderveld, den Hartog, Vissink, Raghoebar, & Meijer, 2014 | 1, University, Europe | Cross sectional | 90, 36.5, 46.7, NR, 90 | NR |
| Zuiderveld, Meijer, den Hartog, Vissink, & Raghoebar, 2018 | 1, University, Europe | RCT | 30, 47.8, 50, No, 30 | Yes |
|  |  |  | 30, 45.5, 56.7, No, 30 |  |
| Zuiderveld, Meijer, Vissink, & Raghoebar, 2018 | 1, University, Europe | RCT | 20, 38.2, 45, No, 20 | No |
|  |  |  | 20, 42, 65, No, 20 |  |
|  |  |  | 20, 45.4, 65, No, 20 |  |
| Zuiderveld et al., 2021 | 1, University, Europe | RCT | 28, 45.3, 57.1, NR, 28 | Yes |
|  |  |  | 27, 47.2, 55.5, NR, 27 |  |

**Legend**. G1: treatment group 1; G2: treatment group 2; G3: treatment group 3; NR: not reported. Priv. Practice: private practice; Univ.: university; *: treatment arm not included in the quantitative analysis; †: number of patients included in the statistical analysis at the first follow-up time point (≥ 6 months).

**Supplementary Table 4.** General characteristics of the included cross-sectional studies.

| **Study** | **Patients (N), implants (n), Single/multiple implants** | **Location** | **Implant type** | **Bone graft, soft tissue graft** | **Loading** | **Restoration** | **Mean follow-up from loading (months)** |
| --- | --- | --- | --- | --- | --- | --- | --- |
| Able, Sartori, Younes, & Bombarda, 2021 | 52, 355, Multiple | Maxilla, Anterior and posterior | Internal conical connection (Neodent) | NR, NR | Mixed (immediate, early, delayed) | Screw-retained | 56.3 |
| Adibrad, Shahabuei, & Sahabi, 2009 | 27, 66, Multiple | Maxilla and mandible, NR | NR | NR, NR | NR | NR | 25.4 |
| Apaza-Bedoya et al., 2023 | 99, 266, Single and Multiple | Maxilla and mandible, Anterior and posterior | Internal conical connection (Implacil) | NR, NR | NR | NR | 30.2 |
| Chang, Wennstrom, Odman, & Andersson, 1999 | 20, 20, Single | Maxilla, Anterior and premolar | Branemark (Nobel Biocare) | NR, NR | NR | NR | 48 |
| Cosyn & De Rouck, 2009 | 27, 27, Single | Maxilla, Anterior | NobelReplace (Nobel Biocare) | DBBM (when needed), NR | Delayed | Cement-retained | 21 |
| Cosyn, Sabzevar, & De Bruyn, 2012 | 97, 97, Single | Maxilla, Anterior and premolar | NobelReplace (Nobel Biocare) | Yes (when needed), No | Delayed | Cement-retained | 31 |
| Duque, Aristizabal, Londono, Castro, & Alvarez, 2016 | 25, 64, Single and Multiple | NR, NR | 3i Biomet | NR, NR | NR | Screw-retained and cement-retained | 12.0 |
| Hof et al., 2014 | 43, 43, Single | Mandible, Anterior | Nobel Biocare | Yes (when needed), No | Delayed | NR | 37 |
| Iglhaut et al., 2021 | 40, 40, Single | Maxilla and mandible, Posterior | Simple Solution BioHorizons and Laser-Lok Tapered BioHorizons | NR, NR | NR | NR | 37.2 |
| Kungsadalpipob et al., 2020 | 200, 412 | Maxilla and mandible, Anterior and posterior | Straumann, Astra Tech, Zimmer, Replace | NR, NR | NR | Screw-retained and cement-retained | 52.8 |
| Mailoa et al., 2018 | 14, 14, Single | Maxilla and mandible, NR | NR | NR, NR | Delayed | NR | 92.4 |
| Manopattanasoontorn et al., 2021 | 165, 331 | Maxilla and mandible, Premolar and molar | Straumann, Astra Tech, Zimmer, Nobel replace, Intra-lock, and others | NR, NR | NR | Screw-retained and cement-retained | 54.0 |
| Nettemu et al., 2021 | 80, 714 | Maxilla and mandible, Anterior and posterior | NR | NR, NR | NR | NR | 20.4 |
| Nisapakultorn, Suphanantachat, Silkosessak, & Rattanamongkolgul, 2010 | 40, 40, Single | Maxilla, Anterior | Paragon (Zimmer dental), Astra Tech (Astra Tech), SteriOss Replace (Nobel Biocare), Frialit-2 (Friadent) and Tissue Level (Straumann) | NR, NR | NR | NR | NR |
| Obreja et al., 2021 | 19, 29 | Maxilla, Anterior and premolar | Ankylos (Dentsply Sirona) + CTG | No, CTG | Delayed | NR | 86.3 |
| Obreja et al., 2021 | 36, 55 | Maxilla, Anterior and premolar | Ankylos (Dentsply Sirona) without soft tissue graft | No, No | Delayed | NR | 49.9 |
| Obreja et al., 2022 | 44, 57 | NR, Anterior and premolar | morse taper implant (Ankylos, Dentsply Sirona) | DBBM (when needed), CTG (when needed) | Immediate | NR | 47.6 |
| Parvini et al., 2023 | 27, 27, Single | Maxilla, Anterior and premolar | Ankylos Dentsply Sirona | DBBM (when needed), CTG (when needed) | Immediate | Screw-retained and cement-retained | 71.3 |
| Parvini et al., 2023 | 25, 25, Single | Maxilla, Anterior and premolar | Straumann BLX | DBBM (when needed), CTG (when needed) | Immediate | Screw-retained and cement-retained | 26.6 |
| Ramanauskaite et al., 2020 | 121, 283, Single and Multiple | Maxilla and mandible, Anterior and posterior | Ankylos Dentsply Sirona | No, No | Delayed | Screw-retained and cement-retained | 141.1 |
| Ramanauskaite et al., 2020 | 111, 291, Single and Multiple | Maxilla and mandible, Anterior and posterior | Ankylos Dentsply Sirona | DBBM, No | Delayed | Screw-retained and cement-retained | 97.4 |
| Roccuzzo, Grasso, & Dalmasso, 2016* | 63, 63, Single and Multiple | Mandible, Posterior | Tissue level SLA implant (Straumann) | NR, No | Delayed | NR | 120 |
| Roccuzzo, Grasso, & Dalmasso, 2016* | 24, 24, Single and Multiple | Mandible, Posterior | Tissue level SLA implant (Straumann) | NR, No | Delayed | NR | 120 |
| Roccuzzo, Grasso, & Dalmasso, 2016* | 11, 11, Single and Multiple | Mandible, Posterior | Tissue level SLA implant (Straumann) | NR, FGG | Delayed | NR | 120 |
| Romandini et al., 2021 | 92, 272, Single and Multiple | Maxilla and mandible, Anterior and first premolar | Straumann, Nobel Biocare, AstraTech | NR, NR | NR | Screw-retained and cement-retained | NR |
| Sanz-Martin et al., 2020 | 104, 104, Single and Multiple | Maxilla and mandible, Anterior and first premolar | Straumann tissue level and Straumann bone level | NR, NR | NR | Screw-retained and cement-retained | 133.2 |
| Suphanantachat, Thovanich, & Nisapakultorn, 2012 | 40, 40, Single | Maxilla, Anterior | NR | NR, NR | NR | NR | NR |
| Tavelli et al., 2022 | 158, 176, Single and Multiple | Maxilla, Anterior and first premolar | Bone level implants | NR, No | NR | NR | 88.8 |
| Thoma, Maggetti, Waller, Hammerle, & Jung, 2019 | 19, 27, Single | Maxilla and mandible, Anterior and posterior | Straumann implant and Branemark system | Autogenous bone block, No | NR | NR | 122.4 |
| Thoma, Maggetti, Waller, Hammerle, & Jung, 2019 | 19, 39, Single and Multiple | Maxilla and mandible, Anterior and posterior | Straumann implant and Branemark system | No, No | NR | NR | 99.6 |
| Tur & Saribas, 2023 | 60, 202, Single and Multiple | Maxilla and mandible, Anterior and posterior | NR | NR, No | NR | NR | NR |
| Ueno et al., 2016 | 60, 90, Single and Multiple | Maxilla and mandible, Premolar and molar | Nobel and Straumann | NR, NR | NR | Screw-retained and cement-retained | 56.4 |
| Ziebolz et al., 2017 * | 17, 24, Single and Multiple | Maxilla and mandible, Anterior and posterior | NR | NR, NR | NR | NR | NR |
| Ziebolz et al., 2017 * | 15, 26, Single and Multiple | Maxilla and mandible, Anterior and posterior | NR | NR, NR | NR | NR | NR |
| Ziebolz et al., 2017 * | 16, 30, Single and Multiple | Maxilla and mandible, Anterior and posterior | NR | NR, NR | NR | NR | NR |
| Ziebolz et al., 2017* | 14, 21, Single and Multiple | Maxilla and mandible, Anterior and posterior | NR | NR, NR | NR | NR | NR |
| Zuiderveld, den Hartog, Vissink, Raghoebar, & Meijer, 2014 | 90, 90, Single | NR, Anterior | Internal conical connection (Nobel replace, Straumann) | Yes, No | Delayed | NR | 12.0 |

**Legend:** NR: not reported; * baseline data on MREC available at one time point only, and therefore the study was used as a cross sectional study

**Supplementary Table 5.** Prevalence and amount/depth of PSTD and MREC reported in the included cross sectional studies.

| **Study** | **Definition of PSTD/MREC** | **Prevalence PSTD (%)** | **PSTD depth**  **(mean ± SD) (mm)** | **Prevalence MREC (%)** | **MREC depth (mean ± SD) (mm)** | **OR PSTD/MREC (impl level) - Risk indicators**  **(implant level)** | **Prevalence PSTD/MREC**  **(patient level) (%)** |
| --- | --- | --- | --- | --- | --- | --- | --- |
| Able, Sartori, Younes, & Bombarda, 2021 | MREC | NR | NR | 20.85 | NR | NR | 61.54 |
| Adibrad, Shahabuei, & Sahabi, 2009 | MREC | NR | NR | NR | 0.79 ± 0.85 | SSD correlation KM < 2 mm and MREC, compared to implant with KM ≥ 2 mm (p=0.03) | NR |
| Apaza-Bedoya et al., 2023 | MREC | NR | NR | 37.6 | 0.45 ± 0.80 | NR | NR |
| Chang, Wennstrom, Odman, & Andersson, 1999 | PSTD | NR | 1 ± 1.3 | NR | NR | NR | NR |
| Cosyn & De Rouck, 2009 | PSTD | NR | 0.3 ± 1.1 | NR | NR | NR | NR |
| Cosyn, Sabzevar, & De Bruyn, 2012 | PSTD | 35.1 | NR | NR | NR | Buccal shoulder position significantly associated with PSTD (OR 17.2, 95% CI 3.1-100.00, p=0.001). | NR |
| Duque, Aristizabal, Londono, Castro, & Alvarez, 2016 | MREC | NR | NR | NR | 0.3 ± 0.7 | NR | NR |
| Hof et al., 2014 | PSTD | 49 | NR | NR | NR | SSD association of PSTD with papilla index (p=0.001), and subject esthetic index (p=0.021) | NR |
| Iglhaut et al., 2021 | MREC | NR | NR | NR | 0.33 ± 0.7 | NR | NR |
| Kungsadalpipob et al., 2020 | MREC | NR | NR | NR | 0.03 /- 0.2 | MREC ≥ 1 mm SSD associated with absence of KM (OR 3.20 95% CI 1.03-9.90, p<0.05) | NR |
| Mailoa et al., 2018 | MREC | 50 | NR | NR | 0.57 ± 0.65 | SSD association of MREC with thin MT (p=0.021) | NR |
| Manopattanasoontorn et al., 2021 | MREC | NR | NR | NR | 0.1 ± 0.45 | SSD association between MREC and KMW < 2 mm (B=0.12, CI 0.01, 0.23, p=0.038) | NR |
| Nettemu et al., 2021 | MREC | NR | NR | 22.69 | NR | BOP SSD associated to presence of MREC (OR 2.601, 95% CI 1.817-3.722, p<0.001) | NR |
| Nisapakultorn, Suphanantachat, Silkosessak, & Rattanamongkolgul, 2010 | PSTD | 35 | 0.5 ± 0.9 | NR | NR | Periodontal phenotype (OR 18.8 (95% CI 2-180) p=0.01), facial bone crest level (OR 1.3 (95% CI 1-1.7) p=0.03), implant fixture angle (OR 0.9 (95% CI 0.8-1) p=0.02), contact point to bone crest (OR 3.4 (95% CI 1.3-8.8) p=0.01), contact point to platform (OR 2.3 (95% CI 1.3-4.2) p=0.005), and contact point to implant bone (OR 2.4 (95% CI 1.2-4.7) p=0.01) SSD associated with PSTD | NR |
| Obreja et al., 2021 | MREC | NR | NR | NR | 0.09 ± 0.21 | NR | NR |
| Obreja et al., 2021 | MREC | NR | NR | NR | 0.07 ± 0.25 | SSD correlation between KM and MREC in the control group only (p=0.003) | NR |
| Obreja et al., 2022 | MREC | NR | NR | NR | 0.03 ± 0.13 | NR | NR |
| Parvini et al., 2023 | MREC | NR | NR | NR | 0.01 ± 0.64 | NR | NR |
| Parvini et al., 2023 | MREC | NR | NR | NR | 0.05 ± 0.18 | NR | NR |
| Ramanauskaite et al., 2020 | MREC | NR | NR | NR | 0.16 ± 0.36 | Increased MREC when KM < 2 mm (p=0.001) | NR |
| Ramanauskaite et al., 2020 | MREC | NR | NR | NR | 0.19 ± 0.36 | Increased MREC when KM < 2 mm (p=0.001) | NR |
| Roccuzzo, Grasso, & Dalmasso, 2016* | MREC | NR | NR | NR | 0.16 ± 0.39 | NR | NR |
| Roccuzzo, Grasso, & Dalmasso, 2016* | MREC | NR | NR | NR | 2.08 ± 0.71 | NR | NR |
| Roccuzzo, Grasso, & Dalmasso, 2016* | MREC | NR | NR | NR | 1.27 ± 1.17 | NR | NR |
| Romandini et al., 2021 | MREC | NR | NR | 12 | 0.15 ± 0.47 | too buccal OR 14.67 (p=0.006), thin phenotype OR 8.31 (p=0.008), lack of adjacent tooth OR 0.08 (p=0.001), lack of abutment OR 0.12 (p=0.02) | 15.8 |
| Sanz-Martin et al., 2020 | MREC | NR | NR | NR | 2.09 ± 0.79 | KM ≤ 2 OR 0.06 (p=0.001), buccal position mm out bone > 1 mm OR 34.65 (p<0.001), one piece implant OR 11.89 (p=0.022) associated with MREC. KM, mm outside bone, and buccal bone dehiscence SSD associated with the depth of MREC | 50 |
| Suphanantachat, Thovanich, & Nisapakultorn, 2012 | PSTD | 35 | 0.5 ± 0.9 | NR | NR | NR | NR |
| Tavelli et al., 2022 | PSTD | 56.8 | 1.58 ± 1.06 | NR | NR | Presence Adjacent implants (OR 10.9 p<0.001), Years in function (OR 1.4, p=0.001), KM (OR 0.73, p=0.03), MT at 1 mm (OR 0.11, p<0.001), MT at 3 mm (OR 0.34, p=0.01), BBD (OR 1.41, p=0.02) | 54.2 |
| Thoma, Maggetti, Waller, Hammerle, & Jung, 2019 | MREC | NR | NR | NR | 0.38 ± 0.57 | NR | NR |
| Thoma, Maggetti, Waller, Hammerle, & Jung, 2019 | MREC | NR | NR | NR | 0.17 ± 0.46 | NR | NR |
| Tur & Saribas, 2023 | MREC | NR | NR | NR | 1.01 ± 1.2 | MREC significantly higher when KM < 2 mm (p=0.001) and in presence of thin mucosa phenotype (p=0.001) | NR |
| Ueno et al., 2016 | MREC | NR | NR | NR | 0.42 ± 0.74 | NR | NR |
| Ziebolz et al., 2017 * | MREC | NR | NR | NR | 1.5 ± 0.89 | NR | NR |
| Ziebolz et al., 2017 * | MREC | NR | NR | NR | 0.88 ± 1.14 | NR | NR |
| Ziebolz et al., 2017 * | MREC | NR | NR | NR | 1.23 ± 1 | NR | NR |
| Ziebolz et al., 2017 * | MREC | NR | NR | NR | 1.67 ± 0.66 | NR | NR |
| Zuiderveld, den Hartog, Vissink, Raghoebar, & Meijer, 2014 | PSTD | NR | 0.55 ± 0.72 | NR | NR | Factors associated with the condition: pre-implant bone augmentation (p=0.005), thick biotype (p=0.005), and buccal implant position (p=0.015) | NR |

**Legend:** BOP: bleeding on probing; CI: confidence interval; KM: keratinized mucosa; MREC: mucosal recession; MT: mucosal thickness; NR: not reported; OR: odds ratio; PSTD: peri-implant soft tissue dehiscence; SSD: statistically significant difference; * baseline data on MREC available at one time point only, and therefore the study was used as a cross sectional study.

**Supplementary Table 6.** Clinical, radiographic, esthetic, and patient-reported outcomes of the included cross sectional studies.

| **Study** | **KM**  **(mean ± SD) (mm)** | **MT**  **(mean ± SD) (mm)** | **PD**  **(mean ± SD) (mm)** | **Rx marginal bone levels**  **(mean ± SD) (mm)** | **Patient-reported esthetics (0-10 VAS)**  **(mean ± SD) (points)** | **PES (total) (0-14)**  **(mean ± SD) (points)** |
| --- | --- | --- | --- | --- | --- | --- |
| Able, Sartori, Younes, & Bombarda, 2021 | NR | NR | NR | NR | NR | NR |
| Adibrad, Shahabuei, & Sahabi, 2009 | 2.5 ± 1.5 | NR | 3.12 ± 0.71 | 1.25 ± 0.64 | NR | NR |
| Apaza-Bedoya et al., 2023 | 3.06 ± 1.89 | NR | 2.77 ± 1.19 | 0.2 ± 1.65 | NR | NR |
| Chang, Wennstrom, Odman, & Andersson, 1999 | 3.9 ± 1.4 | 2 ± 0.7 | NR | NR | NR | NR |
| Cosyn & De Rouck, 2009 | 5.3 ± 1.8 | NR | 3.2 ± 0.6 | NR | NR | NR |
| Cosyn, Sabzevar, & De Bruyn, 2012 | NR | NR | NR | 1.2 ± NR | NR | NR |
| Duque, Aristizabal, Londono, Castro, & Alvarez, 2016 | NR | NR | 2.7 ± 1.1 | 1.73 ± 0.88 | NR | NR |
| Hof et al., 2014 | 3.6 ± 1.9 | NR | 2.3 ± 1 | 1 | 8.7 | 9.12 |
| Iglhaut et al., 2021 | 2.08 ± 1.1 | NR | 3.48 ± 1.0 | 0.41 ± 0.5 | NR | NR |
| Kungsadalpipob et al., 2020 | NR | NR | NR | NR | NR | NR |
| Mailoa et al., 2018 | NR | 2.43 ± 0.68 | 2.43 ± 0.76 | NR | NR | NR |
| Manopattanasoontorn et al., 2021 | 2.32 ± 1.38 | NR | 2.86 ± 0.78 | 0.98 ± 1.34 | NR | NR |
| Nettemu et al., 2021 | NR | NR | NR | NR | NR | NR |
| Nisapakultorn, Suphanantachat, Silkosessak, & Rattanamongkolgul, 2010 | NR | NR | 2.6 ± 1 | NR | NR | NR |
| Obreja et al., 2021 | NR | NR | 2.40 ± 0.54 | NR | NR | NR |
| Obreja et al., 2021 | NR | NR | 2.99 ± 0.64 | NR | NR | NR |
| Obreja et al., 2022 | 4.29 ± 1.60 | NR | 2.52 ± 0.88 | 0.13 ± 0.5 | NR | 12.06 |
| Parvini et al., 2023 | 5.00 ± 1.08 | NR | 2.31 ± 0.56 | NR | NR | 12.1 ± 1.1 |
| Parvini et al., 2023 | 5.51 ± 1.09 | NR | 2.23 ± 0.35 | NR | NR | 11.2 ± 1.86 |
| Ramanauskaite et al., 2020 | 2.84 ± 1.61 | NR | 3 ± 0.85 | NR | NR | NR |
| Ramanauskaite et al., 2020 | 2.47 ± 1.52 | NR | 2.78 ± 0.86 | NR | NR | NR |
| Roccuzzo, Grasso, & Dalmasso, 2016* | NR | NR | 3.13 ± 0.59 | 0.34 ± 0.38 | NR | NR |
| Roccuzzo, Grasso, & Dalmasso, 2016* | 0 | NR | 2.77 ± 0.7 | 0.50 ± 0.38 | NR | NR |
| Roccuzzo, Grasso, & Dalmasso, 2016* | NR | NR | 2.95 ± 0.8 | 0.56 ± 0.39 | NR | NR |
| Romandini et al., 2021 | 2.12 ± 1.45 | NR | NR | NR | 7.81 | NR |
| Sanz-Martin et al., 2020 | 1.65 ± 1.31 with MREC, and 3.27 ± 1.28 without MREC | NR | 2.88 ± 0.94 | 1.71 ± 1.04 | NR | NR |
| Suphanantachat, Thovanich, & Nisapakultorn, 2012 | NR | NR | NR | NR | 8.45 ± 1.36 | NR |
| Tavelli et al., 2022 | 2.2 ± 1.7 implants with PSTD and 4.5 ± 1.7 without PSTD | MT1= 0.65 ± 0.36, MT3 =1.35 ± 0.56 | 2.6 ± 0.6 | NR | NR | NR |
| Thoma, Maggetti, Waller, Hammerle, & Jung, 2019 | 2.91 ± 1.09 | NR | 3.13 ± 0.7 | 1.75 ± 1.11 | NR | NR |
| Thoma, Maggetti, Waller, Hammerle, & Jung, 2019 | 2.69 ± 1.51 | NR | 3.01 ± 0.63 | 1.21 ± 0.93 | NR | NR |
| Tur & Saribas, 2023 | NR | NR | 4.21 ± 1.42 | NR | NR | NR |
| Ueno et al., 2016 | 2.35 ± 2.01 | NR | NR | NR | NR | NR |
| Ziebolz et al., 2017 * | NR | NR | 1.75 ± 1.23 | NR | NR | NR |
| Ziebolz et al., 2017 * | NR | NR | 1.77 ± 1.58 | NR | NR | NR |
| Ziebolz et al., 2017 * | NR | NR | 2.67 ± 1.63 | NR | NR | NR |
| Ziebolz et al., 2017 * | NR | NR | 2 ± 1.38 | NR | NR | NR |
| Zuiderveld, den Hartog, Vissink, Raghoebar, & Meijer, 2014 | NR | NR | NR | NR | NR | NR |

**Legend.** KM: keratinized mucosa; MT: mucosal thickness; NR: not reported; PD: probing depth; PES: pink esthetic score; SD: standard deviation; VAS: visual analogue scale; * baseline on MREC available at one time point only, and therefore the study was used as a cross sectional study

**Supplementary Table 7.** General characteristics of the included prospective studies

| **Article** | **Study design, Patients (N), Implants (n)** | **Intervention** | **Implant type** | **Location** | **Flap, Guided surgery** | **Bone graft, soft tissue graft** | **Provisional/ healing abutment/ cover screw** | **Final Restoration** |
| --- | --- | --- | --- | --- | --- | --- | --- | --- |
| Andersson, Odman, Lindvall, & Branemark, 1998 | nonRCT, 57, 65 | Implant placement | CeraOne (Nobel Biocare) | Maxilla and mandible, Ant and premolar | NR, NR | NR, NR | NR | All-ceramic and metal-ceramic crown |
| Arora & Ivanovski, 2017 | nonRCT, 18, 18 | Immediate implant placement + immediate provisionalization | Astra Tech | Maxilla, Anterior | No, NR | DBBM, No | Immediate provisionalization | NR |
| Arora & Ivanovski, 2017 | nonRCT, 18, 18 | Immediate implant placement + immediate provisionalization | Astra Tech | Maxilla, Anterior | No, NR | DBBM, No | Immediate provisionalization | NR |
| Arora & Ivanovski, 2018 | nonRCT, 15, 15 | Immediate implant placement | Straumann | Maxilla, Anterior | No, NR | DBBM, No | Healing abutment | NR |
| Arora & Ivanovski, 2018 | nonRCT, 15, 15 | Early implant placement | Straumann | Maxilla, Anterior | Yes, NR | DBBM, No | Healing abutment | NR |
| Arora, Khzam, Roberts, Bruce, & Ivanovski, 2017 | nonRCT, 30, 30 | Immediate implant placement + immediate provisionalization | Astra Tech | Maxilla, Anterior | No, NR | DBBM, No | Immediate provisionalization | NR |
| Arora, Khzam, Roberts, Bruce, & Ivanovski, 2017 | nonRCT, 30, 30 | Immediate implant placement + immediate provisionalization | Astra Tech | Maxilla, Anterior | No, NR | DBBM, No | Immediate provisionalization | NR |
| Atef, El Barbary, Dahrous, & Zahran, 2021 | RCT, 21, 21 | Immediate implant placement + socket shield | IS-II (NeoBiotech) | Maxilla, Ant and premolar | No, NR | No, No | Cover screw | NR |
| Atef, El Barbary, Dahrous, & Zahran, 2021 | RCT, 21, 21 | Immediate implant placement | IS-II (NeoBiotech) | Maxilla, Ant and premolar | No, NR | Xenograft, No | Cover screw | NR |
| Atef, El Barbary, Dahrous, & Zahran, 2021 | RCT, 21, 21 | Immediate implant placement + socket shield | IS-II (NeoBiotech) | Maxilla, Ant and premolar | No, NR | No, No | Cover screw | NR |
| Atef, El Barbary, Dahrous, & Zahran, 2021 | RCT, 21, 21 | Immediate implant placement | IS-II (NeoBiotech) | Maxilla, Ant and premolar | No, NR | Xenograft, No | Cover screw | NR |
| Barone et al., 2016 | RCT, 58, 58 | Implant placement (regular insertion torque) | Blossom CT implant (Intralock International) | Maxilla and mandible, Ant and posterior | Yes, Yes | No, No | Cover screw | Metal-ceramic crown |
| Barone et al., 2016 | RCT, 58, 58 | Implant placement (high insertion torque) | CT implant (Intralock International) | Maxilla and mandible, Ant and posterior | Yes, Yes | No, No | Cover screw | Metal-ceramic crown |
| Barone, Toti, Quaranta, Derchi, & Covani, 2015 | nonRCT, 15, 15 | Immediate implant placement + immediate provisionalization | Blossom implant (Intralock International) | Maxilla and mandible, Canine and premolar | No, NR | DBBM, No | Immediate provisionalization | Metal-ceramic crown |
| Barone, Toti, Quaranta, Derchi, & Covani, 2015 | nonRCT, 15, 15 | Immediate implant placement (delayed restoration) | Blossom implant (Intralock International) | Maxilla and mandible, Canine and premolar | No, NR | DBBM, No | Cover screw | Metal-ceramic crown |
| Bengazi, Wennströnm & Lekholm, 1996 | nonRCT, 41, 40 | Implant placement (in presence of alveolar mucosa) | Branemark (Nobel Biocare) | Maxilla and mandible, Full arch | Yes, NR | No, No | NR | NR |
| Bengazi, Wennströnm & Lekholm, 1996 | nonRCT, 41, 202 | Implant placement (in presence of KM) | Branemark (Nobel Biocare) | Maxilla and mandible, Full arch | Yes, NR | No, No | NR | NR |
| Benic et al., 2012 | nonRCT, 14, 14 | Immediate implant placement + GBR | Tissue Level (Straumann) | Maxilla and mandible, Anterior and premolar | Yes, NR | DBBM, No | Healing abutment | Porcelain fused to metal crown |
| Benic et al., 2017 | nonRCT, 10, 10 | Implant placement + GBR | Bone Level (Straumann) | Maxilla and mandible, Anterior and premolar | Yes, NR | DBBM, No | Cover screw | Metal-ceramic crown |
| Benic et al., 2017 | nonRCT, 18, 18 | Implant placement (without bone augmentation) | Bone Level (Straumann) | Maxilla and mandible, Anterior and premolar | Yes, NR | No, No | Cover screw | Metal-ceramic crown |
| Benic et al., 2017 | nonRCT, 10, 10 | Implant placement + GBR | Bone Level (Straumann) | Maxilla and mandible, Anterior and premolar | Yes, NR | DBBM, No | Cover screw | Metal-ceramic crown |
| Benic et al., 2017 | nonRCT, 18, 18 | Implant placement (without bone augmentation) | Bone Level (Straumann) | Maxilla and mandible, Anterior and premolar | Yes, NR | No, No | Cover screw | Metal-ceramic crown |
| Benitez Silva et al., 2022 | RCT, 27, 27 | ARP with DBBM, and implant placement after 4 months | BLT (Straumann) | Maxilla, Anterior | Yes, Yes | DBBM + CM, No | NR | NR |
| Benitez Silva et al., 2022 | RCT, 27, 27 | ARP with DBBM-C, and implant placement after 4 months | BLT (Straumann) | Maxilla, Anterior | Yes, Yes | DBBM-C + CM, No | NR | NR |
| Bianchi & Sanfilippo, 2004 | RCT, 96, 96 | Immediate implant placement + CTG | Tissue level (Straumann) | Maxilla and mandible, NR | No, NR | No, CTG (palate or tuberosity) | Cover screw | Fused to gold-allow crown with a ceramic occlusal surface |
| Bianchi & Sanfilippo, 2004 | RCT, 20, 20 | Immediate implant placement | Tissue level (Straumann) | Maxilla and mandible, NR | No, NR | No, No | NR | Fused to gold-allow crown with a ceramic occlusal surface |
| Bianchi & Sanfilippo, 2004 | RCT, 96, 96 | Immediate implant placement + CTG | Tissue level (Straumann) | Maxilla and mandible, NR | No, NR | No, CTG (palate or tuberosity) | Cover screw | Fused to gold-allow crown with a ceramic occlusal surface |
| Bianchi & Sanfilippo, 2004 | RCT, 20, 20 | Immediate implant placement | Tissue level (Straumann) | Maxilla and mandible, NR | No, NR | No, No | NR | Fused to gold-allow crown with a ceramic occlusal surface |
| Bianchi & Sanfilippo, 2004 | RCT, 96, 96 | Immediate implant placement + CTG | Tissue level (Straumann) | Maxilla and mandible, NR | No, NR | No, CTG (palate or tuberosity) | Cover screw | Fused to gold-allow crown with a ceramic occlusal surface |
| Bianchi & Sanfilippo, 2004 | RCT, 20, 20 | Immediate implant placement | Tissue level (Straumann) | Maxilla and mandible, NR | No, NR | No, No | NR | Fused to gold-allow crown with a ceramic occlusal surface |
| Bittner et al., 2019 and Bittner et al., 2020b | RCT, 14, 14 | Immediate implant placement in patients with thin phenotype | PrimaConnex (Keystone Dental) | Maxilla, Ant and premolar | No, NR | No, No | Customized healing abutment or screw-retained temporary restoration | Zirconia cement-retained crown |
| Bittner et al., 2019 and Bittner et al., 2020b | RCT, 11, 11 | Immediate implant placement (implant with anodised pink neck) in patients with thin phenotype | Genesis (Keystone Dental) | Maxilla, Ant and premolar | No, NR | No, No | Customized healing abutment or screw-retained temporary restoration | Zirconia cement-retained crown |
| Bittner et al., 2019 and Bittner et al., 2020b | RCT, 14, 14 | Immediate implant placement in patients with thick phenotype | PrimaConnex (Keystone Dental) | Maxilla, Ant and premolar | No, NR | No, No | Customized healing abutment or screw-retained temporary restoration | Zirconia cement-retained crown |
| Bittner et al., 2019 and Bittner et al., 2020b | RCT, 11, 11 | Immediate implant placement (implant with anodised pink neck) in patients with thick phenotype | Genesis (Keystone Dental) | Maxilla, Ant and premolar | No, NR | No, No | Customized healing abutment or screw-retained temporary restoration | Zirconia cement-retained crown |
| Bittner et al., 2020a | RCT, 16, 16 | Immediate implant placement + immediate provisionalization with bone graft | Certain (Zimmer Biomet) | Maxilla, Ant and premolar | No, NR | DBBM-C, No | Customized healing abutment or screw-retained temporary restoration | Screw-retained porcelain-fused-to-metal crown |
| Bittner et al., 2020a | RCT, 16, 16 | Immediate implant placement + immediate provisionalization with bone graft | Certain (Zimmer Biomet) | Maxilla, Ant and premolar | No, NR | DBBM-C, No | Customized healing abutment or screw-retained temporary restoration | Screw-retained porcelain-fused-to-metal crown |
| Bittner et al., 2020a | RCT, 16, 16 | Immediate implant placement + immediate provisionalization | Certain (Zimmer Biomet) | Maxilla, Ant and premolar | No, NR | No, No | Customized healing abutment or screw-retained temporary restoration | Screw-retained porcelain-fused-to-metal crown |
| Bittner et al., 2020a | RCT, 15, 15 | Immediate implant placement + immediate provisionalization | Certain (Zimmer Biomet) | Maxilla, Ant and premolar | No, NR | No, No | Customized healing abutment or screw-retained temporary restoration | Screw-retained porcelain-fused-to-metal crown |
| Blanes, Bernard, Blanes, & Belser, 2007 | nonRCT, 83, 192 | Implant placement | Tissue Level (Straumann) | NR, NR | Yes, NR | NR, NR | NR | NR |
| Block et al., 2009 | RCT, 29, 29 | ARP, delayed implant placement, and immediate provisionalization | Certain Implant, Biology of Metals 3i) | NR, NR | Yes, NR | NR, NR | NR | NR |
| Block et al., 2009 | RCT, 29, 29 | ARP, delayed implant placement, and immediate provisionalization | Certain Implant, Biology of Metals 3i) | Maxilla, Ant and premolar | Yes, NR | No, No | Immediate provisionalization | Porcelain-fused-to-metal crown |
| Block et al., 2009 | RCT, 29, 29 | ARP, delayed implant placement, and immediate provisionalization | Certain Implant, Biology of Metals 3i) | Maxilla, Ant and premolar | Yes, NR | No, No | Immediate provisionalization | Porcelain-fused-to-metal crown |
| Block et al., 2009 | RCT, 29, 29 | ARP, delayed implant placement, and immediate provisionalization | Certain Implant, Biology of Metals 3i) | Maxilla, Ant and premolar | Yes, NR | No, No | Immediate provisionalization | Porcelain-fused-to-metal crown |
| Block et al., 2009 | RCT, 26, 26 | Immediate implant placement + immediate provisionalization | Certain Implant, Biology of Metals 3i) | Maxilla, Ant and premolar | No, NR | No, No | Immediate provisionalization | Porcelain-fused-to-metal crown |
| Block et al., 2009 | RCT, 26, 26 | Immediate implant placement + immediate provisionalization | Certain Implant, Biology of Metals 3i) | Maxilla, Ant and premolar | No, NR | No, No | Immediate provisionalization | Porcelain-fused-to-metal crown |
| Block et al., 2009 | RCT, 26, 26 | Immediate implant placement + immediate provisionalization | Certain Implant, Biology of Metals 3i) | Maxilla, Ant and premolar | No, NR | No, No | Immediate provisionalization | Porcelain-fused-to-metal crown |
| Block et al., 2009 | RCT, 26, 26 | Immediate implant placement + immediate provisionalization | Certain Implant, Biology of Metals 3i) | Maxilla, Ant and premolar | No, NR | No, No | Immediate provisionalization | Porcelain-fused-to-metal crown |
| Bonino et al., 2018 | nonRCT, 24, 28 | Implant placement | NR | Maxilla and mandible, Anterior and posterior | NR, NR | NR, NR | Cover screw | NR |
| Bragger, Hammerle, & Lang, 1996 | nonRCT, 15, 20 | Immediate implant placement (with barrier membrane) | ITI dental implant (Straumann) | NR, NR | NR, NR | No, No | Cover screw | NR |
| Bragger, Hammerle, & Lang, 1996 | nonRCT, 6, 8 | Immediate implant placement (without barrier membrane) | ITI dental implant (Straumann) | NR, NR | NR, NR | No, No | Cover screw | NR |
| Bragger, Hammerle, & Lang, 1996 | nonRCT, 20, 20 | Implant placement | ITI dental implant (Straumann) | NR, NR | NR, NR | No, No | Cover screw | NR |
| Bressan et al., 2017 | RCT, 30, 30 | Implant placement + definitive abutment | Ankylos C/X (Dentsply Sirona) | Maxilla and mandible, Ant and posterior | Yes and no (depending on the case), NR | Yes, if needed, No | NR | Metal-ceramic crown |
| Bressan et al., 2017 | RCT, 33, 33 | Implant placement + definitive abutment | Ankylos C/X (Dentsply Sirona) | Maxilla and mandible, Ant and posterior | Yes and no (depending on the case), NR | Yes, if needed, No | NR | Metal-ceramic crown |
| Bressan et al., 2017 | RCT, 37, 37 | Implant placement + repeated abutment changes | Ankylos C/X (Dentsply Sirona) | Maxilla and mandible, Ant and posterior | Yes and no (depending on the case), NR | Yes, if needed, No | NR | Metal-ceramic crown |
| Bressan et al., 2017 | RCT, 39, 39 | Implant placement + repeated abutment changes | Ankylos C/X (Dentsply Sirona) | Maxilla and mandible, Ant and posterior | Yes and no (depending on the case), NR | Yes, if needed, No | NR | Metal-ceramic crown |
| Brunello et al., 2022 | nonRCT, 48, 48 | Implant placement (zirconia implant) | Patent (Zircon Medical) | Maxilla and mandible, Posterior | Yes, NR | DBBM, No | Healing abutment | All-ceramic crown |
| Brunello et al., 2022 | nonRCT, 30, 30 | Implant placement (zirconia implant) | Patent (Zircon Medical) | Maxilla and mandible, Posterior | Yes, NR | DBBM, No | Healing abutment | All-ceramic crown |
| Buser et al., 2009 | nonRCT, 20, 20 | Early implant placement + GBR | Bone Level (Straumann) | Maxilla, Anterior | Yes, NR | Autogenous and DBBM, No | Cover screw | All-ceramic crown |
| Buser et al., 2011 | nonRCT, 20, 20 | Early implant placement + GBR | Bone Level (Straumann) | Maxilla, Anterior | Yes, NR | Autogenous and DBBM, No | Cover screw | All-ceramic crown |
| Buser, Chappuis, Bornstein, et al., 2013 | nonRCT, 41, 41 | Early implant placement + GBR | Tissue Level (Straumann) | Maxilla, Anterior and premolar | Yes, NR | Autogenous and DBBM, No | Cover screw | Metal ceramic crown |
| Buser, Chappuis, Kuchler, et al., 2013 | nonRCT, 20, 20 | Early implant placement + GBR | Bone Level (Straumann) | Maxilla, Anterior | Yes, NR | Autogenous and DBBM, No | Cover screw | All-ceramic crown |
| Bushahri et al., 2021 | RCT, 16, 16 | Immediate implant placement + immediate provisionalization | IS II Active (Neobiotech) | Maxilla, Ant and premolar | No, Yes | Allograft, No | Immediate provisionalization | Ceramic crown |
| Bushahri et al., 2021 | RCT, 12, 12 | Immediate implant placement + delayed restoration | IS II Active (Neobiotech) | Maxilla, Ant and premolar | No, Yes | Allograft, No | Healing abutment | Ceramic crown |
| Cabello, Rioboo, & Fabrega, 2013 | nonRCT, 14, 14 | Immediate implant + immediate provisionalization | Tissue Level (Straumann) | Maxilla, Anterior and premolar | No, NR | No, No | Immediate provisionalization | Metal-ceramic crown or Zirconia-based crown |
| Canullo, Caneva, & Tallarico, 2017 | RCT, 10, 10 | Immediate implant placement (platform switching) | Global Implant (Sweden and Martina) | Maxilla, Ant and premolar | No, NR | DBBM-C (if gap > 1 mm), No | Immediate provisionalization | Gold-ceramic crown |
| Canullo, Caneva, & Tallarico, 2017 | RCT, 9, 9 | Immediate implant placement (non platform switching) | Global Implant (Sweden and Martina) | Maxilla, Ant and premolar | No, NR | DBBM-C (if gap > 1 mm), No | Immediate provisionalization | Gold-ceramic crown |
| Canullo, Iurlaro, & Iannello, 2009 | RCT, 11, 11 | Immediate implant placement (platform switching) | Global Implant (Sweden and Martina) | Maxilla, Ant and premolar | No, NR | DBBM-C (if gap > 1 mm), No | Immediate provisionalization | Gold-ceramic crown |
| Canullo, Iurlaro, & Iannello, 2009 | RCT, 11, 11 | Immediate implant placement (non platform switching) | Global Implant (Sweden and Martina) | Maxilla, Ant and premolar | No, NR | DBBM-C (if gap > 1 mm), No | Immediate provisionalization | Gold-ceramic crown |
| Cardaropoli, Gaveglio, Gherlone, & Cardaropoli, 2014 | RCT, 26, 26 | Immediate implant placement + DBBM-C + CM | Osseotite Tapered Certain (Biomet 3i) | Maxilla and mandible, Ant and premolar | No, NR | DBBM-C + CM, No | Healing abutment | NR |
| Cardaropoli, Gaveglio, Gherlone, & Cardaropoli, 2014 | RCT, 26, 26 | Immediate implant placement | Osseotite Tapered Certain (Biomet 3i) | Maxilla and mandible, Ant and premolar | No, NR | No, No | Healing abutment | NR |
| Cecchinato, Lops, Salvi, & Sanz, 2015 | RCT, 43, 43 | Immediate implant placement (cylindrical implant) | Osseospeed (Dentsply) | Maxilla, Ant and premolar | No, NR | No, No | Healing abutment | NR |
| Cecchinato, Lops, Salvi, & Sanz, 2015 | RCT, 40, 40 | Immediate implant placement (cylindrical implant) | Osseospeed (Dentsply) | Maxilla, Ant and premolar | No, NR | No, No | Healing abutment | NR |
| Cecchinato, Lops, Salvi, & Sanz, 2015 | RCT, 43, 43 | Immediate implant placement (cylindrical implant) | Osseospeed (Dentsply) | Maxilla, Ant and premolar | No, NR | No, No | Healing abutment | NR |
| Cecchinato, Lops, Salvi, & Sanz, 2015 | RCT, 45, 45 | Immediate implant placement (conical/cylindrical implant) | Osseospeed (Dentsply) | Maxilla, Ant and premolar | No, NR | No, No | Healing abutment | NR |
| Cecchinato, Lops, Salvi, & Sanz, 2015 | RCT, 43, 43 | Immediate implant placement (conical/cylindrical implant) | Osseospeed (Dentsply) | Maxilla, Ant and premolar | No, NR | No, No | Healing abutment | NR |
| Cecchinato, Lops, Salvi, & Sanz, 2015 | RCT, 39, 39 | Immediate implant placement (conical/cylindrical implant) | Osseospeed (Dentsply) | Maxilla, Ant and premolar | No, NR | No, No | Healing abutment | NR |
| Chan et al., 2019 | RCT, 18, 18 | Immediate implant placement + immediate provisionalization | IS II Active (Neobiotech) | Maxilla, Ant and premolar | No, Yes | Allograft, No | Immediate provisionalization | Ceramic crown |
| Chan et al., 2019 | RCT, 20, 20 | Immediate implant placement + delayed restoration | IS II Active (Neobiotech) | Maxilla, Ant and premolar | No, Yes | Allograft, No | Healing abutment | Ceramic crown |
| Chen, Darby, & Reynolds, 2007 | nonRCT, 10, 10 | Immediate implant placement (+ DBBM) | Tissue Level (Straumann) | Maxilla, Anterior | Yes, NR | DBBM, No | Cover screw | NR |
| Chen, Darby, & Reynolds, 2007 | nonRCT, 10, 10 | Immediate implant placement (+ DBBM and CM) | Tissue Level (Straumann) | Maxilla, Anterior | Yes, NR | DBBM, No | Cover screw | NR |
| Chen, Darby, & Reynolds, 2007 | nonRCT, 10, 10 | Immediate implant placement (without graft) | Tissue Level (Straumann) | Maxilla, Anterior | Yes, NR | No, No | Cover screw | NR |
| Chung, Rungcharassaeng, Kan, Roe, & Lozada, 2011 | nonRCT, 10, 10 | Immediate implant (+ CTG) + immediate provisionalization | Osseotite Prevail (Biomet 3i) | Maxilla and mandible, Anterior and premolar | No, NR | DBBM, CTG | Immediate provisionalization | Metal-ceramic crown |
| Cooper et al., 2010 | nonRCT, 55, 55 | Immediate implant placement + immediate provisionalization | OsseoSpeed (AstraTech) | Maxilla, Ant and premolar | No, NR | No, No | Immediate provisionalization | Ceramic crown |
| Cooper et al., 2010 | nonRCT, 60, 60 | Delayed implant placement | OsseoSpeed (AstraTech) | Maxilla, Ant and premolar | Yes, NR | No, No | Immediate provisionalization | Ceramic crown |
| Cooper et al., 2014 | nonRCT, 45, 45 | Immediate implant placement + immediate provisionalization | OsseoSpeed (AstraTech) | Maxilla, Ant and premolar | No, NR | No, No | Immediate provisionalization | Ceramic crown |
| Cooper et al., 2014 | nonRCT, 49, 49 | Delayed implant placement | OsseoSpeed (AstraTech) | Maxilla, Ant and premolar | Yes, NR | No, No | Immediate provisionalization | Ceramic crown |
| Cooper et al., 2015 | RCT, 48, 53 | implant placement with conus interface between abutment and implant | OsseoSpeed (Dentsply Sirona) | Maxilla, Ant and premolar | No, NR | Yes, if needed, If needed | Immediate provisionalization | Lithium disilicate crown |
| Cooper et al., 2015 | RCT, 49, 53 | implant placement with flat-to-flat interface between abutment and implant | Speedy Replace (Nobel Biocare) | Maxilla, Ant and premolar | No, NR | Yes, if needed, If needed | Immediate provisionalization | Lithium disilicate crown |
| Cooper et al., 2015 | RCT, 44, 50 | implant placement with platform switched interface between abutment and implant | NanoTite Certain Prevail (Biomet 3i) | Maxilla, Ant and premolar | No, NR | Yes, if needed, If needed | Immediate provisionalization | Lithium disilicate crown |
| Cooper et al., 2019 | RCT, 45, 45 | implant placement with conus interface between abutment and implant | OsseoSpeed (Dentsply Sirona) | Maxilla, Ant and premolar | No, NR | Yes, if needed, If needed | Immediate provisionalization | Lithium disilicate crown |
| Cooper et al., 2019 | RCT, 34, 34 | implant placement with flat-to-flat interface between abutment and implant | Speedy Replace (Nobel Biocare) | Maxilla, Ant and premolar | No, NR | Yes, if needed, If needed | Immediate provisionalization | Lithium disilicate crown |
| Cooper et al., 2019 | RCT, 32, 32 | implant placement with platform switched interface between abutment and implant | NanoTite Certain Prevail (Biomet 3i) | Maxilla, Ant and premolar | No, NR | Yes, if needed, If needed | Immediate provisionalization | Lithium disilicate crown |
| Cordaro, Torsello, & Roccuzzo, 2009 | RCT, 14, 14 | Immediate implant placement (submerged) + delayed provisionalization | Tapered TE Implants (Straumann Dental Implant System) | Maxilla, Ant and premolar | Yes, NR | No, No | Cover screw | NR |
| Cordaro, Torsello, & Roccuzzo, 2009 | RCT, 14, 14 | Immediate implant placement (submerged) + delayed provisionalization | Tapered TE Implants (Straumann Dental Implant System) | Maxilla, Ant and premolar | Yes, NR | No, No | Cover screw | NR |
| Cordaro, Torsello, & Roccuzzo, 2009 | RCT, 14, 14 | Immediate implant placement (non submerged) + delayed provisionalization | Tapered TE Implants (Straumann Dental Implant System) | Maxilla, Ant and premolar | Yes, NR | No, No | Healing abutment | NR |
| Cordaro, Torsello, & Roccuzzo, 2009 | RCT, 14, 14 | Immediate implant placement (non submerged) + delayed provisionalization | Tapered TE Implants (Straumann Dental Implant System) | Maxilla, Ant and premolar | Yes, NR | No, No | Healing abutment | NR |
| Cosyn et al., 2011 | nonRCT, 28, 28 | Immediate implant placement | NobelReplace (Nobel Biocare) | Maxilla, Ant and premolar | Yes, NR | DBBM, No | Healing abutment | Metal-ceramic crown |
| Cosyn et al., 2011 | nonRCT, 25, 25 | Immediate implant placement | NobelReplace (Nobel Biocare) | Maxilla, Ant and premolar | Yes, NR | DBBM, No | Healing abutment | Metal-ceramic crown |
| Cosyn et al., 2016 | nonRCT, 15, 15 | Immediate implant placement + immediate provisionalization | NobelActive (Nobel Biocare) | Maxilla, Ant and premolar | No, NR | DBBM, No | Immediate provisionalization | Full-ceramic crown or metal-ceramic crown |
| Cosyn et al., 2016 | nonRCT, 12, 12 | Immediate implant placement + immediate provisionalization | NobelActive (Nobel Biocare) | Maxilla, Ant and premolar | No, NR | DBBM, No | Immediate provisionalization | Full-ceramic crown or metal-ceramic crown |
| Cosyn et al., 2016 | nonRCT, 7, 7 | Immediate implant placement + immediate provisionalization + CTG after 3 months | NobelActive (Nobel Biocare) | Maxilla, Ant and premolar | No, NR | DBBM, CTG (3 months after implant placement) | Immediate provisionalization | Full-ceramic crown or metal-ceramic crown |
| Cosyn et al., 2016 | nonRCT, 5, 5 | Immediate implant placement + immediate provisionalization + CTG after 3 months | NobelActive (Nobel Biocare) | Maxilla, Ant and premolar | No, NR | DBBM, CTG (3 months after implant placement) | Immediate provisionalization | Full-ceramic crown or metal-ceramic crown |
| Cosyn et al., 2022 | RCT, 30, 30 | Implant placement + CTG | Nobel Replace CC (Nobel Biocare) | Maxilla and mandible, Ant and premolar | Yes, NR | No, CTG | Immediate provisionalization | NR |
| Cosyn et al., 2022 | RCT, 30, 30 | Implant placement + CMX | Nobel Replace CC (Nobel Biocare) | Maxilla and mandible, Ant and premolar | Yes, NR | No, CMX | Immediate provisionalization | NR |
| Cosyn, De Bruyn, & Cleymaet, 2013 | nonRCT, 22, 22 | Immediate implant placement + immediate provisionalization | NobelActive (Nobel Biocare) | Maxilla, Ant and premolar | No, NR | DBBM, CTG (3 months after implant placement) if needed | Immediate provisionalization | Full-ceramic crown or metal-ceramic crown |
| Cosyn, De Bruyn, & Cleymaet, 2013 | nonRCT, 22, 22 | Immediate implant placement + immediate provisionalization | NobelActive (Nobel Biocare) | Maxilla, Ant and premolar | No, NR | DBBM, CTG (3 months after implant placement) if needed | Immediate provisionalization | Full-ceramic crown or metal-ceramic crown |
| Cosyn, Pollaris, Van der Linden, & De Bruyn, 2015 | nonRCT, 39, 39 | ARP + delayed implant placement + CTG (at sites without midfacial recession) | Nobel Active (Nobel Biocare) | Maxilla, Anterior and premolar | No, NR | No, CTG | Cover screw or healing abutment | Full-ceramic crown |
| Cosyn, Pollaris, Van der Linden, & De Bruyn, 2015 | nonRCT, 8, 8 | ARP + CTG + delayed implant placement (at sites with midfacial recession) | Nobel Active (Nobel Biocare) | Maxilla, Anterior and premolar | No, NR | No, CTG | Cover screw or healing abutment | Full-ceramic crown |
| Covani, Canullo, Toti, Alfonsi, & Barone, 2014 | nonRCT, 45, 45 | Immediate implant placement | Khono (Sweden & Martina) | Maxilla and mandible, Anterior and premolar | No, NR | Xenograft, No | Cover screw | NR |
| Crespi et al., 2019 | RCT, 21, 61 | Immediate implant placement (≥ 2 mm KT) + immediate provisionalization | external hex Outlink (Sweden & Martina) | Maxilla and mandible, Ant and premolar | No, NR | No, No | Immediate provisionalization | Screwed Ceramic crown |
| Crespi et al., 2019 | RCT, 21, 61 | Immediate implant placement (≥ 2 mm KT) + immediate provisionalization | external hex Outlink (Sweden & Martina) | Maxilla and mandible, Ant and premolar | No, NR | No, No | Immediate provisionalization | Cemented Ceramic crown |
| Crespi et al., 2019 | RCT, 21, 61 | Immediate implant placement (≥ 2 mm KT) + immediate provisionalization | external hex Outlink (Sweden & Martina) | Maxilla and mandible, Ant and premolar | No, NR | No, No | Immediate provisionalization | Screwed Ceramic crown |
| Crespi et al., 2019 | RCT, 21, 61 | Immediate implant placement (≥ 2 mm KT) + immediate provisionalization | external hex Outlink (Sweden & Martina) | Maxilla and mandible, Ant and premolar | No, NR | No, No | Immediate provisionalization | Cemented Ceramic crown |
| Crespi et al., 2019 | RCT, 21, 61 | Immediate implant placement (≥ 2 mm KT) + immediate provisionalization | external hex Outlink (Sweden & Martina) | Maxilla and mandible, Ant and premolar | No, NR | No, No | Immediate provisionalization | Screwed Ceramic crown |
| Crespi et al., 2019 | RCT, 21, 61 | Immediate implant placement (≥ 2 mm KT) + immediate provisionalization | external hex Outlink (Sweden & Martina) | Maxilla and mandible, Ant and premolar | No, NR | No, No | Immediate provisionalization | Cemented Ceramic crown |
| Crespi et al., 2019 | RCT, 21, 62 | Immediate implant placement (<2 mm KT) + immediate provisionalization | external hex Outlink (Sweden & Martina) | Maxilla and mandible, Ant and premolar | No, NR | No, No | Immediate provisionalization | Screwed Ceramic crown |
| Crespi et al., 2019 | RCT, 21, 62 | Immediate implant placement (<2 mm KT) + immediate provisionalization | external hex Outlink (Sweden & Martina) | Maxilla and mandible, Ant and premolar | No, NR | No, No | Immediate provisionalization | Cemented Ceramic crown |
| Crespi et al., 2019 | RCT, 21, 62 | Immediate implant placement (<2 mm KT) + immediate provisionalization | external hex Outlink (Sweden & Martina) | Maxilla and mandible, Ant and premolar | No, NR | No, No | Immediate provisionalization | Screwed Ceramic crown |
| Crespi et al., 2019 | RCT, 21, 62 | Immediate implant placement (<2 mm KT) + immediate provisionalization | external hex Outlink (Sweden & Martina) | Maxilla and mandible, Ant and premolar | No, NR | No, No | Immediate provisionalization | Cemented Ceramic crown |
| Crespi et al., 2019 | RCT, 21, 62 | Immediate implant placement (<2 mm KT) + immediate provisionalization | external hex Outlink (Sweden & Martina) | Maxilla and mandible, Ant and premolar | No, NR | No, No | Immediate provisionalization | Screwed Ceramic crown |
| Crespi et al., 2019 | RCT, 21, 62 | Immediate implant placement (<2 mm KT) + immediate provisionalization | external hex Outlink (Sweden & Martina) | Maxilla and mandible, Ant and premolar | No, NR | No, No | Immediate provisionalization | Cemented Ceramic crown |
| Crespi, Cappare, & Gherlone, 2010a | nonRCT, NR, 125 | Immediate implant placement + immediate provisionalization (in presence of KM > 2 mm) | Seven (Sweden & Martina) | Maxilla and mandible, Anterior and premolar | No, NR | No, No | Immediate provisionalization | NR |
| Crespi, Cappare, & Gherlone, 2010a | nonRCT, NR, 125 | Immediate implant placement + immediate provisionalization (in presence of KM > 2 mm) | Seven (Sweden & Martina) | Maxilla and mandible, Anterior and premolar | No, NR | No, No | Immediate provisionalization | NR |
| Crespi, Cappare, & Gherlone, 2010a | nonRCT, NR, 39 | Immediate implant placement + immediate provisionalization (in presence of KM < 2 mm) | Seven (Sweden & Martina) | Maxilla and mandible, Anterior and premolar | No, NR | No, No | Immediate provisionalization | NR |
| Crespi, Cappare, & Gherlone, 2010a | nonRCT, NR, 39 | Immediate implant placement + immediate provisionalization (in presence of KM < 2 mm) | Seven (Sweden & Martina) | Maxilla and mandible, Anterior and premolar | No, NR | No, No | Immediate provisionalization | NR |
| Crespi, Cappare, & Gherlone, 2010b | RCT, 15, 15 | Immediate implant placement (in sites without peri-apical lesion) | Seven (Sweden Martina) | Maxilla and mandible, Ant and premolar | Yes, NR | No, No | Cover screw | Metal ceramic crown |
| Crespi, Cappare, & Gherlone, 2010b | RCT, 15, 15 | Immediate implant placement (in sites without peri-apical lesion) | Seven (Sweden Martina) | Maxilla and mandible, Ant and premolar | Yes, NR | No, No | Cover screw | Metal ceramic crown |
| Crespi, Cappare, & Gherlone, 2010b | RCT, 15, 15 | Immediate implant placement (in sites with peri-apical lesion) | Seven (Sweden Martina) | Maxilla and mandible, Ant and premolar | Yes, NR | No, No | Cover screw | Metal ceramic crown |
| Crespi, Cappare, & Gherlone, 2010b | RCT, 15, 15 | Immediate implant placement (in sites with peri-apical lesion) | Seven (Sweden Martina) | Maxilla and mandible, Ant and premolar | Yes, NR | No, No | Cover screw | Metal ceramic crown |
| Crespi, Cappare, Gherlone, & Romanos, 2012 | nonRCT, 15, 20 | Immediate implant placement + immediate provisionalization | Seven (Sweden & Martina) | Maxilla and mandible, Anterior and premolar | No, NR | No, No | Immediate provisionalization | NR |
| Crespi, Cappare, Gherlone, & Romanos, 2012 | nonRCT, 15, 20 | Immediate implant placement + immediate provisionalization | Seven (Sweden & Martina) | Maxilla and mandible, Anterior and premolar | No, NR | No, No | Immediate provisionalization | NR |
| Crespi, Cappare, Polizzi, & Gherlone, 2015 | nonRCT, NR, 47 | Immediate implant placement (implant with short collar) + immediate provisionalization | Outlink (Sweden & Martina) | Maxilla, Anterior | No, NR | No, No | Immediate provisionalization | Ceramic-fused-to-metal |
| Crespi, Cappare, Polizzi, & Gherlone, 2015 | nonRCT, NR, 47 | Immediate implant placement (implant with short collar) + immediate provisionalization | Outlink (Sweden & Martina) | Maxilla, Anterior | No, NR | No, No | Immediate provisionalization | Ceramic-fused-to-metal |
| Crespi, Cappare, Polizzi, & Gherlone, 2015 | nonRCT, NR, 47 | Immediate implant placement (implant with long collar) + immediate provisionalization | Advanced (Ticino Forniture Dentali) | Maxilla, Anterior | No, NR | No, No | Immediate provisionalization | Ceramic-fused-to-metal |
| Crespi, Cappare, Polizzi, & Gherlone, 2015 | nonRCT, NR, 47 | Immediate implant placement (implant with long collar) + immediate provisionalization | Advanced (Ticino Forniture Dentali) | Maxilla, Anterior | No, NR | No, No | Immediate provisionalization | Ceramic-fused-to-metal |
| D'Elia et al., 2017 | RCT, 15, 15 | Implant placement + GBR | SPI Element (Thommen Medical) | Maxilla, Ant and premolar | Yes, NR | DBBM, No | Healing abutment | Porcelain-fused-to-ceramic crown |
| D'Elia et al., 2017 | RCT, 15, 15 | Implant placement + CTG | SPI Element (Thommen Medical) | Maxilla, Ant and premolar | Yes, NR | No, CTG | Healing abutment | Porcelain-fused-to-ceramic crown |
| da Rosa, Rosa, Francischone, & Sotto-Maior, 2014 | nonRCT, 18, 18 | Immediate implant placement (+ CTG) + immediate provisionalization | Nobel Replace (Nobel Biocare) | Maxilla, Anterior and premolar | No, NR | Autogenous (from tuberosity), No | Immediate provisionalization | Metal-ceramic crown or Zirconia-based crown |
| de Albornoz et al., 2014 | RCT, 11, 11 | Restoration with zirconia abutment | SPI Element (Thommen Medical) | Maxilla, Anterior | Yes, NR | No, No | NR | NR |
| de Albornoz et al., 2014 | RCT, 14, 14 | Restoration with titanium abutment | SPI Element (Thommen Medical) | Maxilla, Anterior | Yes, NR | No, No | NR | NR |
| De Bruyckere et al., 2020 | RCT, 21, 21 | Implant placement + CTG | NobelActive TiUnite (Nobel Biocare) | Maxilla, Anterior | Yes, NR | No, CTG | Healing abutment | NR |
| De Bruyckere et al., 2020 | RCT, 21, 21 | Implant placement + GBR | NobelActive TiUnite (Nobel Biocare) | Maxilla, Anterior | Yes, NR | DBBM, No | Cover screw | NR |
| De Bruyn et al., 2013 | nonRCT, 46, 46 | Immediate implant placement + immediate provisionalization | OsseoSpeed (AstraTech) | Maxilla, Ant and premolar | No, NR | No, No | Immediate provisionalization | Ceramic crown |
| De Bruyn et al., 2013 | nonRCT, 51, 51 | Delayed implant placement | OsseoSpeed (AstraTech) | Maxilla, Ant and premolar | Yes, NR | No, No | Immediate provisionalization | Ceramic crown |
| De Rouck, Collys, & Cosyn, 2008 | nonRCT, 32, 32 | Immediate implant placement + immediate provisionalization | Nobelreplace tapered TiUnite (Nobel Biocare) | Maxilla, Ant and premolar | Yes, NR | DBBM, No | Immediate provisionalization | Metal ceramic crown |
| De Rouck, Collys, Wyn, & Cosyn, 2009 | RCT, 24, 24 | Immediate implant placement + immediate provisionalization | Nobelreplace tapered TiUnite (Nobel Biocare) | Maxilla, Ant and premolar | Yes, NR | DBBM, No | Immediate provisionalization | Metal ceramic crown |
| De Rouck, Collys, Wyn, & Cosyn, 2009 | RCT, 24, 24 | Immediate implant placement + immediate provisionalization | Nobelreplace tapered TiUnite (Nobel Biocare) | Maxilla, Ant and premolar | Yes, NR | DBBM, No | Immediate provisionalization | Metal ceramic crown |
| De Rouck, Collys, Wyn, & Cosyn, 2009 | RCT, 25, 25 | Immediate implant placement with submerged healing and delayed restoration | Nobelreplace tapered TiUnite (Nobel Biocare) | Maxilla, Ant and premolar | Yes, NR | DBBM, No | Cover screw | Metal ceramic crown |
| De Rouck, Collys, Wyn, & Cosyn, 2009 | RCT, 25, 25 | Immediate implant placement with submerged healing and delayed restoration | Nobelreplace tapered TiUnite (Nobel Biocare) | Maxilla, Ant and premolar | Yes, NR | DBBM, No | Cover screw | Metal ceramic crown |
| de Siqueira et al., 2017 | RCT, 11, 28 | Implant placement (equicrestal) | Titamax CM (Neodent) | Maxilla and mandible, Full arch | Yes, NR | No, No | Immediate provisionalization | NR |
| de Siqueira et al., 2017 | RCT, 11, 27 | Implant placement (subcrestal) | Titamax CM (Neodent) | Maxilla and mandible, Full arch | Yes, NR | No, No | Immediate provisionalization | NR |
| de Siqueira et al., 2020 | RCT, 11, 28 | Implant placement (equicrestal) | Titamax CM (Neodent) | Maxilla and mandible, Full arch | Yes, NR | No, No | Immediate provisionalization | NR |
| de Siqueira et al., 2020 | RCT, 11, 27 | Implant placement (subcrestal) | Titamax CM (Neodent) | Maxilla and mandible, Full arch | Yes, NR | No, No | Immediate provisionalization | NR |
| Degidi, Nardi, Daprile, & Piattelli, 2014 | RCT, 29, 29 | Immediate implant placement + immediate provisionalization (and conventional final impression) | Ankylos (Dentsply) | Maxilla, Incisor and canine | No, NR | No, No | Immediate provisionalization | Metal ceramic crown |
| Degidi, Nardi, Daprile, & Piattelli, 2014 | RCT, 29, 29 | Immediate implant placement + immediate provisionalization (and conventional final impression) | Ankylos (Dentsply) | Maxilla, Incisor and canine | No, NR | No, No | Immediate provisionalization | Metal ceramic crown |
| Degidi, Nardi, Daprile, & Piattelli, 2014 | RCT, 29, 29 | Immediate implant placement + immediate provisionalization (and conventional final impression) | Ankylos (Dentsply) | Maxilla, Incisor and canine | No, NR | No, No | Immediate provisionalization | Metal ceramic crown |
| Degidi, Nardi, Daprile, & Piattelli, 2014 | RCT, 24, 24 | Immediate implant placement + immediate provisionalization (and final impression without abutment removal) | Ankylos (Dentsply) | Maxilla, Incisor and canine | No, NR | No, No | Immediate provisionalization | Metal ceramic crown |
| Degidi, Nardi, Daprile, & Piattelli, 2014 | RCT, 24, 24 | Immediate implant placement + immediate provisionalization (and final impression without abutment removal) | Ankylos (Dentsply) | Maxilla, Incisor and canine | No, NR | No, No | Immediate provisionalization | Metal ceramic crown |
| Degidi, Nardi, Daprile, & Piattelli, 2014 | RCT, 24, 24 | Immediate implant placement + immediate provisionalization (and final impression without abutment removal) | Ankylos (Dentsply) | Maxilla, Incisor and canine | No, NR | No, No | Immediate provisionalization | Metal ceramic crown |
| den Hartog, Raghoebar, Stellingsma, Vissink, & Meijer, 2011 | RCT, 31, 31 | Immediate implant placement + immediate provisionalization | Nobel Replace (Nobel Biocare) | Maxilla, Anterior | NR, Yes | Autogenous bone mixed with DBBM, No | Immediate provisionalization | Zirconia crown |
| den Hartog, Raghoebar, Stellingsma, Vissink, & Meijer, 2011 | RCT, 31, 31 | Conventional Implant placement | Nobel Replace (Nobel Biocare) | Maxilla, Anterior | NR, Yes | Autogenous bone mixed with DBBM, No | Cover screw | Zirconia crown |
| Eghbali et al., 2018 | nonRCT, 37, 37 | ARP + implant placement + CTG (3 months after implant placement) | NobelActive (Nobel Biocare) | Maxilla, Ant and premolar | Yes and no (depending on the case), NR | No, CTG (3 months after implant placement) | Healing abutment | Full-ceramic crown |
| Eghbali et al., 2018 | nonRCT, 37, 37 | ARP + implant placement + CTG (3 months after implant placement) | NobelActive (Nobel Biocare) | Maxilla, Ant and premolar | Yes and no (depending on the case), NR | No, CTG (3 months after implant placement) | Healing abutment | Full-ceramic crown |
| Ekfeldt, Eriksson, & Johansson, 2003 | nonRCT, 10, 58 | Implant placement (maxilla only) | Branemark system (Nobel Biocare) | Maxilla, Full arch | Yes, NR | No, No | NR | Full arch with framework in gold or titanium |
| Ekfeldt, Eriksson, & Johansson, 2003 | nonRCT, 10, 49 | Implant placement (mandible only) | Branemark system (Nobel Biocare) | Mandible, Full arch | Yes, NR | No, No | NR | Full arch with framework in gold or titanium |
| Esposito et al., 2017 | RCT, 40, 40 | Implant placement + transmucosal abutment | Ankylos (Dentsply) | Maxilla and mandible, Ant and posterior | Flap and flapless, NR | NR, No | Cover screw or healing abutment | Metal ceramic crown |
| Esposito et al., 2017 | RCT, 40, 40 | Implant placement + definitive abutment + immediate provisionalization | Ankylos (Dentsply) | Maxilla and mandible, Ant and posterior | Flap and flapless, NR | NR, No | Immediate provisionalization | Metal ceramic crown |
| Farrag & Khamis, 2023 | nonRCT, 28, 28 | Implant placement + anodized titanium abutment collar | Dentium Superline (Dentium) | Maxilla and mandible, Anterior and posterior | NR, NR | NR, NR | Healing abutment | Lithium disilicate crown |
| Farrag & Khamis, 2023 | nonRCT, 28, 28 | Implant placement + unanodized titanium abutment collar | Dentium Superline (Dentium) | Maxilla and mandible, Anterior and posterior | NR, NR | NR, NR | Healing abutment | Lithium disilicate crown |
| Farrag & Khamis, 2023 | nonRCT, 28, 28 | Implant placement + anodized titanium abutment collar | Dentium Superline (Dentium) | Maxilla and mandible, Anterior and posterior | NR, NR | NR, NR | Healing abutment | Lithium disilicate crown |
| Farrag & Khamis, 2023 | nonRCT, 28, 28 | Implant placement + unanodized titanium abutment collar | Dentium Superline (Dentium) | Maxilla and mandible, Anterior and posterior | NR, NR | NR, NR | Healing abutment | Lithium disilicate crown |
| Farronato et al., 2020 | nonRCT, NR, 14 | Implant placement | MegaGen AnyRidge (MegaGen Implant Co.) | Maxilla and mandible, Anterior | Yes, NR | No, No | Cover screw | Metal ceramic crown |
| Farronato et al., 2020 | nonRCT, NR, 14 | Implant placement | MegaGen AnyRidge (MegaGen Implant Co.) | Maxilla and mandible, Anterior | Yes, NR | No, No | Cover screw | Metal ceramic crown |
| Farronato et al., 2020 | nonRCT, NR, 14 | Implant placement | MegaGen AnyRidge (MegaGen Implant Co.) | Maxilla and mandible, Anterior | Yes, NR | No, No | Cover screw | Metal ceramic crown |
| Farronato et al., 2020 | nonRCT, NR, 64 | Implant placement | MegaGen AnyRidge (MegaGen Implant Co.) | Maxilla and mandible, Posterior | Yes, NR | No, No | Cover screw | Metal ceramic crown |
| Farronato et al., 2020 | nonRCT, NR, 64 | Implant placement | MegaGen AnyRidge (MegaGen Implant Co.) | Maxilla and mandible, Posterior | Yes, NR | No, No | Cover screw | Metal ceramic crown |
| Farronato et al., 2020 | nonRCT, NR, 64 | Implant placement | MegaGen AnyRidge (MegaGen Implant Co.) | Maxilla and mandible, Posterior | Yes, NR | No, No | Cover screw | Metal ceramic crown |
| Farronato et al., 2021 | RCT, 38, 38 | Implant placement (platform-switching) | MegaGen AnyRidge (MegaGen Implant Co.) | Maxilla and mandible, Anterior and posterior | Yes, NR | No, No | Cover screw | Metal ceramic crown |
| Farronato et al., 2021 | RCT, 39, 39 | Implant placement (non-platform-switching) | MegaGen AnyRidge (MegaGen Implant Co.) | Maxilla and mandible, Anterior and posterior | Yes, NR | No, No | Cover screw | Metal ceramic crown |
| Farronato et al., 2021 | RCT, 38, 38 | Implant placement (platform-switching) | MegaGen AnyRidge (MegaGen Implant Co.) | Maxilla and mandible, Anterior and posterior | Yes, NR | No, No | Cover screw | Metal ceramic crown |
| Farronato et al., 2021 | RCT, 39, 39 | Implant placement (non-platform-switching) | MegaGen AnyRidge (MegaGen Implant Co.) | Maxilla and mandible, Anterior and posterior | Yes, NR | No, No | Cover screw | Metal ceramic crown |
| Farronato et al., 2021 | RCT, 38, 38 | Implant placement (platform-switching) | MegaGen AnyRidge (MegaGen Implant Co.) | Maxilla and mandible, Anterior and posterior | Yes, NR | No, No | Cover screw | Metal ceramic crown |
| Farronato et al., 2021 | RCT, 39, 39 | Implant placement (non-platform-switching) | MegaGen AnyRidge (MegaGen Implant Co.) | Maxilla and mandible, Anterior and posterior | Yes, NR | No, No | Cover screw | Metal ceramic crown |
| Fenner, Hammerle, Sailer, & Jung, 2016 | nonRCT, 13, 13 | Implant placement (and all-ceramic crowns on aluminium oxide-based abutments) | (Straumann) | NR, NR | Yes, NR | DBBM (when needed), CTG (when needed) | NR | All-ceramic crowns on aluminium oxide-based abutments |
| Fenner, Hammerle, Sailer, & Jung, 2016 | nonRCT, 15, 15 | Implant placement (and metal abutments on porcelain-fused-to-metal crowns) | (Straumann) | NR, NR | Yes, NR | DBBM (when needed), CTG (when needed) | NR | Metal abutments on porcelain-fused-to-metal crowns |
| Fernandes, Marques, Borges, & Montero, 2023 | RCT, 16, 16 | Immediate implant placement | OsseoSpeed (AstraTech, Dentsply) | Maxilla, Ant and premolar | No, NR | DBBM, No | Healing abutment | All-ceramic crown |
| Fernandes, Marques, Borges, & Montero, 2023 | RCT, 16, 16 | Immediate implant placement + CTG | OsseoSpeed (AstraTech, Dentsply) | Maxilla, Ant and premolar | No, NR | DBMM, CTG | Healing abutment | All-ceramic crown |
| Ferrari, Cagidiaco, Garcia-Godoy, Goracci, & Cairo, 2015 | RCT, 15, 27 | Implant placement and restoration with titanium abutment | OsseoSpeed (AstraTech, Dentsply) | Maxilla and mandible, NR | NR, NR | NR, NR | Healing abutment | NR |
| Ferrari, Cagidiaco, Garcia-Godoy, Goracci, & Cairo, 2015 | RCT, 18, 42 | Implant placement and restoration with titanium nitride abutment | OsseoSpeed (AstraTech, Dentsply) | Maxilla and mandible, NR | NR, NR | NR, NR | Healing abutment | NR |
| Ferrari, Cagidiaco, Garcia-Godoy, Goracci, & Cairo, 2015 | RCT, 14, 28 | Implant placement and restoration with zirconia abutment | OsseoSpeed (AstraTech, Dentsply) | Maxilla and mandible, NR | NR, NR | NR, NR | Healing abutment | NR |
| Finelle, Popelut, Knafo, & Sanz Martin, 2021 | nonRCT, 17, 17 | Immediate implant placement + socket seal abutment | Tissue Level or Bone Level (Straumann) | Maxilla and mandible, Posterior | No, NR | DBBM, No | Healing abutment (socket seal abutment) | Lithium disilicate crown and zirconia crown |
| Frizzera et al., 2019 | RCT, 8, 8 | Immediate implant placement and immediate provisionalization | Flash (Conexao Sistemas de Protese) | Maxilla, Incisor | No, NR | DBBM-C, No | Immediate provisionalization | NR |
| Frizzera et al., 2019 | RCT, 8, 8 | Immediate implant placement (+ CMX) and immediate provisionalization | Flash (Conexao Sistemas de Protese) | Maxilla, Incisor | No, NR | DBBM-C, CMX | Immediate provisionalization | NR |
| Frizzera et al., 2019 | RCT, 8, 8 | Immediate implant placement (+ CTG) and immediate provisionalization | Flash (Conexao Sistemas de Protese) | Maxilla, Incisor | No, NR | DBBM-C, CTG | Immediate provisionalization | NR |
| Frizzera et al., 2019 | RCT, 8, 8 | Immediate implant placement and immediate provisionalization | Flash (Conexao Sistemas de Protese) | Maxilla, Incisor | No, NR | DBBM-C, No | Immediate provisionalization | NR |
| Frizzera et al., 2019 | RCT, 8, 8 | Immediate implant placement (+ CTG) and immediate provisionalization | Flash (Conexao Sistemas de Protese) | Maxilla, Incisor | No, NR | DBBM-C, CTG | Immediate provisionalization | NR |
| Frizzera et al., 2019 | RCT, 8, 8 | Immediate implant placement (+ CMX) and immediate provisionalization | Flash (Conexao Sistemas de Protese) | Maxilla, Incisor | No, NR | DBBM-C, CMX | Immediate provisionalization | NR |
| Furhauser et al., 2017 | nonRCT, 77, 77 | Immediate implant + immediate provisionalization | Nobel Replace (Nobel Biocare) and Osseospeed (Astra Tech) | Maxilla, Anterior | NR, NR | No, No | Immediate provisionalization | Ceramic crown |
| Furhauser et al., 2017 | nonRCT, 77, 77 | Immediate implant + immediate provisionalization | Nobel Replace (Nobel Biocare) and Osseospeed (Astra Tech) | Maxilla, Anterior | NR, NR | No, No | Immediate provisionalization | Ceramic crown |
| Furhauser et al., 2017 | nonRCT, 77, 77 | Immediate implant + immediate provisionalization | Nobel Replace (Nobel Biocare) and Osseospeed (Astra Tech) | Maxilla, Anterior | NR, NR | No, No | Immediate provisionalization | Ceramic crown |
| Furhauser et al., 2017 | nonRCT, 77, 77 | Immediate implant + immediate provisionalization | Nobel Replace (Nobel Biocare) and Osseospeed (Astra Tech) | Maxilla, Anterior | NR, NR | No, No | Immediate provisionalization | Ceramic crown |
| Furhauser et al., 2017 | nonRCT, 77, 77 | Immediate implant + immediate provisionalization | Nobel Replace (Nobel Biocare) and Osseospeed (Astra Tech) | Maxilla, Anterior | NR, NR | No, No | Immediate provisionalization | Ceramic crown |
| Gallucci, Grutter, Nedir, Bischof, & Belser, 2011 | RCT, 10, 10 | Implant placement and all-ceramic restoration | Standard Plus (Straumann) | Maxilla, Anterior | NR, NR | No, No | Healing abutment or cover screw | All-ceramic crown |
| Gallucci, Grutter, Nedir, Bischof, & Belser, 2011 | RCT, 10, 10 | Implant placement and porcelain-fused-to-ceramic restoration | Standard Plus (Straumann) | Maxilla, Anterior | NR, NR | No, No | Healing abutment or cover screw | Porcelain-fused-to-ceramic crown |
| Gallucci, Grutter, Nedir, Bischof, & Belser, 2011 | RCT, 10, 10 | Implant placement and all-ceramic restoration | Standard Plus (Straumann) | Maxilla, Anterior | NR, NR | No, No | Healing abutment or cover screw | All-ceramic crown |
| Gallucci, Grutter, Nedir, Bischof, & Belser, 2011 | RCT, 10, 10 | Implant placement and porcelain-fused-to-ceramic restoration | Standard Plus (Straumann) | Maxilla, Anterior | NR, NR | No, No | Healing abutment or cover screw | Porcelain-fused-to-ceramic crown |
| Garaicoa-Pazmino et al., 2021 | nonRCT, 13, 13 | Implant placement (STH ≤ 2 mm) | Tapered tissue level (BioHorizons) | Maxilla and mandible, Premolar and molar | Yes, Yes | No, No | Healing abutment | NR |
| Garaicoa-Pazmino et al., 2021 | nonRCT, 13, 13 | Implant placement (STH > 2 mm) | Tapered tissue level (BioHorizons) | Maxilla and mandible, Premolar and molar | Yes, Yes | No, No | Healing abutment | NR |
| Garaicoa-Pazmino et al., 2021 | nonRCT, 13, 13 | Implant placement (STH ≤ 2 mm) | Tapered tissue level (BioHorizons) | Maxilla and mandible, Premolar and molar | Yes, Yes | No, No | Healing abutment | NR |
| Garaicoa-Pazmino et al., 2021 | nonRCT, 13, 13 | Implant placement (STH > 2 mm) | Tapered tissue level (BioHorizons) | Maxilla and mandible, Premolar and molar | Yes, Yes | No, No | Healing abutment | NR |
| Ghallab et al., 2023 | RCT, 15, 15 | Immediate implant placement (vestibular extraction technique) | Biohorizons | Maxilla, Anterior | No, Yes | Autogenous bone mixed with allograft, No | Healing abutment | NR |
| Ghallab et al., 2023 | RCT, 15, 15 | Immediate implant placement (conventional extraction technique) | Biohorizons | Maxilla, Anterior | No, Yes | Autogenous bone mixed with allograft, No | Healing abutment | NR |
| Girlanda et al., 2019 | RCT, 11, 11 | Immediate implant placement (with DBBM-C) and immediate provisionalization | Full Osseotite Tapered Certain (Biomet 3i ) | Maxilla, Incisor | No, NR | DBBM-C, No | Immediate provisionalization | NR |
| Girlanda et al., 2019 | RCT, 11, 11 | Immediate implant placement (without bone graft) and immediate provisionalization | Full Osseotite Tapered Certain (Biomet 3i ) | Maxilla, Incisor | No, NR | No, No | Immediate provisionalization | NR |
| Gomez-Meda et al., 2022 | nonRCT, 15, 20 | Immediate implant placement (+ CTG) | Tri-vent SBA surface (BioHorizons) and Mozo Grau Inhex STD | Maxilla, Ant and premolar | No, NR | No, CTG | Immediate provisionalization or healing abutment | Zirconia-ceramic crown |
| Gomez-Meda et al., 2022 | nonRCT, 15, 6 | Immediate implant placement (+ socket shield) | Tri-vent SBA surface (BioHorizons) and Mozo Grau Inhex STD | Maxilla, Ant and premolar | No, NR | No, No | Immediate provisionalization or healing abutment | Zirconia-ceramic crown |
| Grandi, Guazzi, Samarani, & Grandi, 2013 | nonRCT, 23, 23 | Immediate implant placement + immediate provisionalization | JDentalCare | Maxilla, Ant and premolar | No, NR | DBBM, No | Immediate provisionalization | NR |
| Grandi, Guazzi, Samarani, & Grandi, 2013 | nonRCT, 24, 24 | ARP + delayed implant placement (flapless) | JDentalCare | Maxilla, Ant and premolar | No, NR | No, No | Immediate provisionalization | NR |
| Grassi et al., 2015 | nonRCT, 17, 32 | Implant placement (zirconia implant) + immediate provisionalization | WhiteSKY zirconia implant (WhiteSKY, Bredent Medical) | Maxilla and mandible, Anterior and posterior | Yes, NR | Synthetic (when needed), No | Immediate provisionalization | Zirconia-ceramic crown |
| Groenendijk, Bronkhorst, & Meijer, 2021 | nonRCT, 97, 97 | Immediate implant + immediate provisionalization | NR | Maxilla, Incisor | No, NR | No, No | Immediate provisionalization | NR |
| Guarnieri et al., 2022 | RCT, 20, 20 | Implant placement (submerged) | Tapered Internal Laser-Lok (BioHorizons) | Maxilla and mandible, Premolar and molar | Yes, NR | No, No | Cover screw | NR |
| Guarnieri et al., 2022 | RCT, 20, 20 | Implant placement (nonsubmerged) | Tapered Tissue Level Laser-Lok (BioHorizons) | Maxilla and mandible, Premolar and molar | Yes, NR | No, No | Healing abutment | NR |
| Guarnieri, Ceccarelli, Ricci, & Testori, 2018 | nonRCT, 16, 16 | Implant placement (implants with laser-microtextured collar surface) | BioLok SilhouetteTM (BioHorizons) | Maxilla and mandible, NR | No, NR | No, No | NR | NR |
| Guarnieri, Ceccarelli, Ricci, & Testori, 2018 | nonRCT, 16, 16 | Implant placement (implants with machined collar surface) | BioLok SilhouetteTM (BioHorizons) | Maxilla and mandible, NR | No, NR | No, No | NR | NR |
| Guarnieri, Ceccherini, & Grande, 2015 | nonRCT, 20, 20 | Immediate implant placement and early loading | BioLok SilhouetteTM (BioHorizons) | Maxilla, Anterior | No, NR | No, No | Immediate provisionalization | All-ceramic crown |
| Guarnieri, Di Nardo, Di Giorgio, Miccoli, & Testarelli, 2019 | RCT, 20, 20 | Implant placement (submerged) | Tapered Internal Laser-Lok (BioHorizons) | Maxilla and mandible, Premolar and molar | Yes, NR | No, No | Cover screw | NR |
| Guarnieri, Di Nardo, Di Giorgio, Miccoli, & Testarelli, 2019 | RCT, 20, 20 | Implant placement (nonsubmerged) | Tapered Tissue Level Laser-Lok (BioHorizons) | Maxilla and mandible, Premolar and molar | Yes, NR | No, No | Healing abutment | NR |
| Guarnieri, Di Nardo, Gaimari, Miccoli, & Testarelli, 2019 | nonRCT, 28, 28 | Implant placement (short implant) | Tapered Short Laser-Lok and Tapered Internal Laser-Lok (BioHorizons) | Maxilla and mandible, Premolar and molar | Yes, NR | No, No | Cover screw | NR |
| Guarnieri, Di Nardo, Gaimari, Miccoli, & Testarelli, 2019 | nonRCT, 28, 28 | Implant placement (standard implant) | Tapered Internal Laser-Lok (BioHorizons) | Maxilla and mandible, Premolar and molar | Yes, NR | No, No | Cover screw | NR |
| Hall et al., 2007 | RCT, 14, 14 | Implant placement with delayed restoration | Southern Implants | Maxilla, Ant and premolar | Yes, No | Autograft if needed, No | Cover screw | Metal-ceramic crown |
| Hall et al., 2007 | RCT, 14, 14 | Implant placement with immediate provisionalization | Southern Implants | Maxilla, Ant and premolar | Yes, No | Autograft if needed, No | Immediate provisionalization | Metal-ceramic crown |
| Hattingh, De Bruyn, Van Weehaeghe, Hommez, & Vandeweghe, 2020 | nonRCT, 27, 27 | Immediate implant placement (ultra-wide implants in molar sites) | MAX (Southern Implants) | Maxilla and mandible, Molar | No, NR | No, No | Healing abutment | NR |
| Hof et al., 2015 | nonRCT, 15, 15 | Implant placement + GBR | Branemark system (Nobel Biocare) | Maxilla, Anterior and premolar | Yes, NR | Autogenous and DBBM, No | Cover screw | NR |
| Hof et al., 2015 | nonRCT, 13, 13 | Delayed implant placement | Branemark system (Nobel Biocare) | Maxilla, Anterior and premolar | Yes, NR | No, No | Cover screw | NR |
| Hof et al., 2015 | nonRCT, 26, 26 | Immediate implant placement | Branemark system (Nobel Biocare) | Maxilla, Anterior and premolar | Yes, NR | No, No | Cover screw | NR |
| Hof et al., 2015 | nonRCT, 35, 35 | Early implant placement | Branemark system (Nobel Biocare) | Maxilla, Anterior and premolar | Yes, NR | No, No | Cover screw | NR |
| Hof et al., 2015 | nonRCT, 64, 64 | Autogenous bone grafting + delayed implant placement | Branemark system (Nobel Biocare) | Maxilla, Anterior and premolar | Yes, NR | No, No | NR | NR |
| Hollander et al., 2016 | nonRCT, 38, 106 | Implant placement (zirconia implant) | Z-Look 3 implant system (Z-Systems) | Maxilla and mandible, Anterior and posterior | NR, NR | No, No | Healing abutment | All-ceramic crown |
| Hosseini et al., 2015 | RCT, 10, 10 | Immediate implant placement + immediate provisionalization (with antibiotic therapy) | Tapered-Screw Vent (Zimmer) | Maxilla and mandible, Ant and premolar | No, NR | No, No | Immediate provisionalization | Porcelain crown |
| Hosseini et al., 2015 | RCT, 10, 10 | Immediate implant placement + immediate provisionalization (without antibiotic therapy) | Tapered-Screw Vent (Zimmer) | Maxilla and mandible, Ant and premolar | No, NR | No, No | Immediate provisionalization | Porcelain crown |
| Humm et al., 2023 | RCT, 15, 13 | Ceramic restoration with zirconia abutment | Branemark (Nobel Biocare) | Maxilla and mandible, Ant and posterior | NR, NR | NR, No | NR | All-ceramic crown |
| Humm et al., 2023 | RCT, 15, 8 | Metal ceramic crown with titanium abutment | Branemark (Nobel Biocare) | Maxilla and mandible, Ant and posterior | NR, NR | NR, No | NR | Metal ceramic crown |
| Huynh-Ba et al., 2019 | RCT, 20, 20 | Immediate implant placement | Straumann Bone Level Implants, SLActive (Straumann) | Maxilla and mandible, Ant and premolar | Yes, NR | Allograft (FDBA), No | Cover screw or healing abutment | NR |
| Huynh-Ba et al., 2019 | RCT, 15, 15 | Early implant placement with GBR | Straumann Bone Level Implants, SLActive (Straumann) | Maxilla and mandible, Ant and premolar | Yes, NR | Allograft (FDBA), No | Cover screw or healing abutment | NR |
| Iorio-Siciliano et al., 2016 | nonRCT, 20, 20 | Implant placement | Laser-Lok Tapered (BioHorizons) | NR, NR | Yes, NR | No, No | Cover screw | Porcelain fused to metal crown |
| Jacobs, Zadeh, De Kok, & Cooper, 2020 | RCT, 19, 19 | Immediate implant placement (+ DBBM) | OsseoSpeed TX Profile (Dentsply Sirona) | Maxilla, Ant and premolar | No, NR | DBBM, No | Healing abutment | Ceramic crown |
| Jacobs, Zadeh, De Kok, & Cooper, 2020 | RCT, 14, 14 | Immediate implant placement (without bone graft) | OsseoSpeed TX Profile (Dentsply Sirona) | Maxilla, Ant and premolar | No, NR | No, No | Healing abutment | Ceramic crown |
| Jeffcoat, McGlumphy, Reddy, Geurs, & Proskin, 2003 | RCT, 40, 40 | Implant placement | Machined-surface Hydroxyapatite-coated Ti threaded implant (Nobel Biocare) | Mandible, Full arch | Yes, NR | No, No | Cover screw | Full arch |
| Jeffcoat, McGlumphy, Reddy, Geurs, & Proskin, 2003 | RCT, 40, 40 | Implant placement | Machined-surface Hydroxyapatite-coated cylindric (Nobel Biocare) | Mandible, Full arch | Yes, NR | No, No | Cover screw | Full arch |
| Jeffcoat, McGlumphy, Reddy, Geurs, & Proskin, 2003 | RCT, 40, 40 | Implant placement | Machined titanium threaded implant (Nobel Biocare) | Mandible, Full arch | Yes, NR | No, No | Cover screw | Full arch |
| Jemt, Ahlberg, Henriksson, & Bondevik, 2006 | nonRCT, 23, 47 | Implant placement | Branemark system (Nobel Biocare) | Maxilla, Anterior | NR, NR | No, No | Cover screw | Porcelain fused to metal crown |
| Jung et al., 2016 | nonRCT, 60, 71 | Implant placement (zirconia implant) + immediate provisionalization | Ceramic.implant (Vita-clinical, VITA Zahnfabrik) | Maxilla and mandible, Anterior and posterior | Yes, NR | DBBM (when needed), No | Immediate provisionalization | Zirconia-ceramic crown |
| Kan, Rungcharassaeng, & Lozada, 2003 | nonRCT, 35, 35 | Immediate implant placement + immediate provisionalization | Replace (Nobel Biocare) | Maxilla, Incisor and canine | No, NR | No, No | Immediate provisionalization | Metal-ceramic crown |
| Kan, Rungcharassaeng, Lozada, & Zimmerman, 2011 | nonRCT, 35, 35 | Immediate implant placement + immediate provisionalization | Replace (Nobel Biocare) | Maxilla, Incisor and canine | No, NR | No, No | Immediate provisionalization | Metal-ceramic crown |
| Kan, Rungcharassaeng, Sclar, & Lozada, 2007 | nonRCT, 23, 23 | Immediate implant placement + immediate provisionalization | Nobel Replace Select or NobelPerfect (Nobel Biocare) | Maxilla, Incisor and canine | Yes and no (depending on the case), NR | Autogenous bone or DBBM, CTG if needed (thin phenotype) | Immediate provisionalization | Metal-ceramic crown |
| Karoussis et al., 2004 | nonRCT, 89, 179 | Implant placement | ITI dental implant (Straumann) | Maxilla and mandible, Anterior and posterior | NR, NR | No, No | NR | NR |
| Kobayashi et al., 2020 | nonRCT, 14, 14 | Implant placement + GBR | NR | Maxilla, Anterior | Yes, NR | DBBM, No | Cover screw | Zirconia-ceramic crown |
| Kobayashi et al., 2020 | nonRCT, 12, 12 | Implant placement + GBR + CTG (at second stage) | NR | Maxilla, Anterior | Yes, NR | DBBM, CTG (at second stage) | Cover screw | Zirconia-ceramic crown |
| Koh et al., 2011 | RCT, 10, 10 | Immediate implant placement (at the level of the crest) | Tubingen Implant (FRIALIT-1) | Maxilla, Ant and premolar | No, Yes | Allograft, No | Cover screw | NR |
| Koh et al., 2011 | RCT, 10, 10 | Immediate implant placement (subcrestal) | Tubingen Implant (FRIALIT-1) | Maxilla, Ant and premolar | No, Yes | Allograft, No | Cover screw | NR |
| Lago, da Silva, Gude, & Rilo, 2017 | nonRCT, 35, 67 | Implant placement | Bone Level (Straumann) | Maxilla and mandible, Premolar | Yes, NR | No, No | Healing abutment | NR |
| Lee et al., 2020 | RCT, 18, 18 | Immediate implant placement (flapless) and immediate provisionalization | Full OSSEOTITE Certain Tapered Implant (Biomet 3i) | Maxilla, Ant and premolar | No, NR | No, No | Immediate provisionalization | NR |
| Lee et al., 2020 | RCT, 21, 21 | Immediate implant placement (with a flap) and immediate provisionalization | Full OSSEOTITE Certain Tapered Implant (Biomet 3i) | Maxilla, Ant and premolar | Yes, NR | No, No | Immediate provisionalization | NR |
| Lee et al., 2023 | RCT, 15, 15 | Immediate implant placement + CTG | NobelReplace (Nobel Biocare) | Maxilla, Ant and premolar | No, Yes | DBBM, CTG | Healing abutment | NR |
| Lee et al., 2023 | RCT, 15, 15 | Immediate implant placement + ADM | NobelReplace (Nobel Biocare) | Maxilla, Ant and premolar | No, Yes | DBBM, ADM | Healing abutment | NR |
| Lee et al., 2023 | RCT, 16, 16 | Immediate implant placement | NobelReplace (Nobel Biocare) | Maxilla, Ant and premolar | No, Yes | DBBM, No | Healing abutment | NR |
| Lilet et al., 2022 | nonRCT, 20, 20 | Immediate implant placement + socket seal abutment | BLX (Straumann) | Maxilla and mandible, Posterior | No, NR | DBBM, No | Healing abutment (socket seal abutment) | Ceramic crown |
| Lindeboom, Tjiook, & Kroon, 2006 | RCT, 23, 23 | Immediate implant placement | Frialit-2 Synchro | Maxilla, Ant and premolar | Yes, NR | Autograft, No | Cover screw | NR |
| Lindeboom, Tjiook, & Kroon, 2006 | RCT, 25, 25 | Delayed implant placement | Frialit-2 Synchro | Maxilla, Ant and premolar | Yes, NR | Autograft, No | Cover screw | NR |
| Liu et al., 2019 | nonRCT, 45, 45 | Immediate implant placement + GBR | Superline (Dentium) | Maxilla, Anterior | Yes, NR | Autogenous mixed with DBBM, No | Healing abutment | NR |
| Lops et al., 2015 | nonRCT, 13, 13 | Implant placement and restoration with zirconia stock abutment | Osseospeed (Astra Tech) | Maxilla, Anterior and premolar | Yes, NR | No, No | Cover screw | Zirconia-ceramic crown |
| Lops et al., 2015 | nonRCT, 23, 23 | Implant placement and restoration with titanium stock abutment | Osseospeed (Astra Tech) | Maxilla, Anterior and premolar | Yes, NR | No, No | Cover screw | Metal-ceramic crown |
| Lops et al., 2015 | nonRCT, 20, 20 | Implant placement and restoration with zirconia cad-cam abutment | Osseospeed (Astra Tech) | Maxilla, Anterior and premolar | Yes, NR | No, No | Cover screw | Zirconia-ceramic crown |
| Lops et al., 2015 | nonRCT, 16, 16 | Implant placement and restoration with titanium cad-dam abutment | Osseospeed (Astra Tech) | Maxilla, Anterior and premolar | Yes, NR | No, No | Cover screw | Metal-ceramic crown |
| Lops, Romeo, Chiapasco, Procopio, & Oteri, 2013 | nonRCT, 21, 21 | Immediate implant placement | SLActive Bone level implants (Straumann) | Maxilla, Premolar | Yes, NR | No, No | Healing abutment | NR |
| Lorenz et al., 2019 | nonRCT, 28, 83 | Implant placement (zirconia implant) | Zirconia implant (NR) | Maxilla and mandible, Anterior and posterior | NR, NR | NR, NR | NR | All-ceramic crown |
| Lorenz et al., 2022 | nonRCT, 19, 24 | Implant placement (zirconia implant) | PURE Ceramic Implant (Straumann) | Maxilla and mandible, Anterior and posterior | NR, NR | Yes (if needed), No | NR | NR |
| Lowy et al., 2019 | RCT, 12, 12 | Implant placement (platform-matched implant) | Laser-grooved platform-matched implant (BioHorizons) | Maxilla, Ant and premolar | Yes, NR | No, No | Cover screw | NR |
| Lowy et al., 2019 | RCT, 12, 12 | Implant placement (platform-switched implant) | Laser-grooved platform-switched implant (BioHorizons) | Maxilla, Ant and premolar | Yes, NR | No, No | Cover screw | NR |
| Malchiodi, Cucchi, Ghensi, & Nocini, 2013 | nonRCT, 58, 64 | Immediate implant placement + immediate provisionalization | NR | Maxilla, Incisor and canine | No, NR | No, No | Immediate provisionalization | Metal-ceramic crown or Zirconia-ceramic crown |
| Marconcini et al., 2018 | RCT, 58, 58 | Implant placement (regular insertion torque) | Blossom CT implant (Intralock International) | Maxilla and mandible, Ant and posterior | Yes, Yes | No, No | Cover screw | Metal-ceramic crown |
| Marconcini et al., 2018 | RCT, 58, 58 | Implant placement (high insertion torque) | CT implant (Intralock International) | Maxilla and mandible, Ant and posterior | Yes, Yes | No, No | Cover screw | Metal-ceramic crown |
| Marconcini et al., 2018 | RCT, 58, 58 | Implant placement (regular insertion torque) | Blossom CT implant (Intralock International) | Maxilla and mandible, Ant and posterior | Yes, Yes | No, No | Cover screw | Metal-ceramic crown |
| Marconcini et al., 2018 | RCT, 58, 58 | Implant placement (high insertion torque) | CT implant (Intralock International) | Maxilla and mandible, Ant and posterior | Yes, Yes | No, No | Cover screw | Metal-ceramic crown |
| Mau et al., 2019 | RCT, 24, 24 | Early implant placement with FDBA | SLActive Bone level implants (Straumann) | Maxilla, Incisor and premolar | Yes, NR | FDBA, No | Cover screw | NR |
| Mau et al., 2019 | RCT, 24, 24 | Early implant placement with autogenous graft + DBBM | SLActive Bone level implants (Straumann) | Maxilla, Incisor and premolar | Yes, NR | Autogenous bone + DBBM, No | Cover screw | NR |
| Meijndert et al., 2017 | RCT, 26, 26 | Bone augmentation with autogenous bone graft and implant placement (after 3 months) | ITI-Esthetic Plus (Straumann) | Maxilla, Anterior | Yes, Yes | Autogenous, No | Cover screw | Porcelain crown |
| Meijndert et al., 2017 | RCT, 18, 18 | Bone augmentation with autogenous bone graft + collagen membrane and implant placement (after 3 months) | ITI-Esthetic Plus (Straumann) | Maxilla, Anterior | Yes, Yes | Autogenous, No | Cover screw | Porcelain crown |
| Meijndert et al., 2017 | RCT, 17, 17 | Bone augmentation with DBBM + collagen membrane and implant placement (after 6 months) | ITI-Esthetic Plus (Straumann) | Maxilla, Anterior | Yes, Yes | DBBM, No | Cover screw | Porcelain crown |
| Meijndert, Raghoebar, Meijer, & Vissink, 2008 | RCT, 31, 31 | Bone augmentation with autogenous bone graft and implant placement (after 3 months) | ITI-Esthetic Plus (Straumann) | Maxilla, Anterior | Yes, Yes | Autogenous, No | Cover screw | Porcelain crown |
| Meijndert, Raghoebar, Meijer, & Vissink, 2008 | RCT, 31, 31 | Bone augmentation with autogenous bone graft + collagen membrane and implant placement (after 3 months) | ITI-Esthetic Plus (Straumann) | Maxilla, Anterior | Yes, Yes | Autogenous, No | Cover screw | Porcelain crown |
| Meijndert, Raghoebar, Meijer, & Vissink, 2008 | RCT, 31, 31 | Bone augmentation with DBBM + collagen membrane and implant placement (after 6 months) | ITI-Esthetic Plus (Straumann) | Maxilla, Anterior | Yes, Yes | DBBM, No | Cover screw | Porcelain crown |
| Meijndert, Raghoebar, Vissink, & Meijer, 2022 | nonRCT, 30, 30 | Implant placement + GBR | Bone Level Tapered (Straumann) | Maxilla, Anterior | Yes, Yes | Autogenous mixed with DBBM (when needed), No | Cover screw | Porcelain-fused-to-zirconia crown |
| Migliorati, Amorfini, Signori, Biavati, & Benedicenti, 2015 | RCT, 24, 24 | Immediate implant placement + CTG | Tapered effect or bone level SLActive (Straumann) | Maxilla, Ant and premolar | No, NR | DBBM, CTG | Immediate provisionalization | NR |
| Migliorati, Amorfini, Signori, Biavati, & Benedicenti, 2015 | RCT, 23, 23 | Immediate implant placement | Tapered effect or bone level SLActive (Straumann) | Maxilla, Ant and premolar | No, NR | DBBM, No | Immediate provisionalization | NR |
| Migliorati, Amorfini, Signori, Biavati, & Benedicenti, 2015 | RCT, 24, 24 | Immediate implant placement + CTG | Tapered effect or bone level SLActive (Straumann) | Maxilla, Ant and premolar | No, NR | DBBM, CTG | Immediate provisionalization | NR |
| Migliorati, Amorfini, Signori, Biavati, & Benedicenti, 2015 | RCT, 23, 23 | Immediate implant placement | Tapered effect or bone level SLActive (Straumann) | Maxilla, Ant and premolar | No, NR | DBBM, No | Immediate provisionalization | NR |
| Mizuno, Nakano, Shimomoto, Fujita, & Ishigaki, 2022 | nonRCT, 20, 20 | Immediate implant + GBR | Tapered implant (Nobel Biocare or Straumann) | Maxilla, Anterior | Yes, Yes | DBBM, No | Cover screw | NR |
| Molina, Sanz-Sanchez, Martin, Blanco, & Sanz, 2017 | RCT, 20, 30 | Implant placement (with healing cap) | Camlog Screw-Line Implant (Camlog) | Maxilla and mandible, Premolar and molar | Yes, NR | NR, No | Healing abutment | NR |
| Molina, Sanz-Sanchez, Martin, Blanco, & Sanz, 2017 | RCT, 16, 26 | Implant placement (with definitive abutment) | Camlog Screw-Line Implant (Camlog) | Maxilla and mandible, Premolar and molar | Yes, NR | NR, No | Definitive abutment | NR |
| Molina, Sanz-Sanchez, Martin, Blanco, & Sanz, 2017 | RCT, 19, 29 | Implant placement (with healing cap) | Camlog Screw-Line Implant (Camlog) | Maxilla and mandible, Premolar and molar | Yes, NR | NR, No | Healing abutment | NR |
| Molina, Sanz-Sanchez, Martin, Blanco, & Sanz, 2017 | RCT, 16, 26 | Implant placement (with definitive abutment) | Camlog Screw-Line Implant (Camlog) | Maxilla and mandible, Premolar and molar | Yes, NR | NR, No | Definitive abutment | NR |
| Munoz-Camara, Gilbel-Del Aguila, Pardo-Zamora, & Camacho-Alonso, 2020 | nonRCT, 50, 50 | Immediate implant placement + immediate provisionalization (at sites with peri-apical pathology) | Biomet 3i (Zimmer Biomet) | Maxilla and mandible, Anterior and posterior | No, NR | DBBM, No | Immediate provisionalization | NR |
| Munoz-Camara, Gilbel-Del Aguila, Pardo-Zamora, & Camacho-Alonso, 2020 | nonRCT, 50, 50 | Immediate implant placement + immediate provisionalization (at sites without peri-apical pathology) | Biomet 3i (Zimmer Biomet) | Maxilla and mandible, Anterior and posterior | No, NR | DBBM, No | Immediate provisionalization | NR |
| Oates, West, Jones, Kaiser, & Cochran, 2002 | nonRCT, 39, 106 | Implant placement | Tissue Level (Straumann) | Maxilla and mandible, Anterior | Yes, NR | No, No | NR | NR |
| Parvini, Muller, Cafferata, Schwarz, & Obreja, 2022 | nonRCT, 14, 16 | Immediate implant placement + immediate provisionalization | BLX (Straumann) | Maxilla, Ant and premolar | No, No | DBBM, No | Immediate provisionalization | NR |
| Parvini, Muller, Cafferata, Schwarz, & Obreja, 2022 | nonRCT, 11, 16 | Delayed implant placement + immediate provisionalization | BLX (Straumann) | Maxilla, Ant and premolar | Yes, No | Autogenous bone mixed with DBBM, No | Immediate provisionalization | NR |
| Perez et al., 2020 | RCT, 18, 18 | Immediate implant placement + customized healing abutment | BLT implants, Straumann (Switzerland) | Maxilla and mandible, Ant and premolar | No, NR | Alloplast, No | Healing abutment | NR |
| Perez et al., 2020 | RCT, 18, 18 | Immediate implant placement + standard healing abutment | BLT implants, Straumann (Switzerland) | Maxilla and mandible, Ant and premolar | No, NR | Alloplast, No | Healing abutment | NR |
| Pieri, Aldini, Marchetti, & Corinaldesi, 2011 | RCT, 19, 19 | Immediate implant placement + immediate provisionalization (abutment with morse connection and platform switch) | Samo Smiler Implants (Biospark) | Maxilla, Premolar | Yes, if facial socket walls not intact, NR | Autogenous bone mixed with DBBM, No | Immediate provisionalization | All-ceramic or meta-ceramic crown |
| Pieri, Aldini, Marchetti, & Corinaldesi, 2011 | RCT, 19, 19 | Immediate implant placement + immediate provisionalization (abutment with internal connection and matching diameter) | Samo Smiler Implants (Biospark) | Maxilla, Premolar | Yes, if facial socket walls not intact, NR | Autogenous bone mixed with DBBM, No | Immediate provisionalization | All-ceramic or meta-ceramic crown |
| Pieri, Aldini, Marchetti, & Corinaldesi, 2013 | nonRCT, 29, 29 | Staged bone augmentation and delayed implant placement | Osseospeed (Astra Tech) | Maxilla, Anterior | Yes, NR | No, CTG | Cover screw | Zirconia-ceramic crown |
| Pohl, Furhauser, Haas, & Pohl, 2020 | nonRCT, 12, 12 | Immediate implant + immediate provisionalization (at sites with buccal bone dehiscence) | Nobel Active (Nobel Biocare) | Maxilla, Incisor and canine | No, NR | No, No | Immediate provisionalization | NR |
| Pohl, Furhauser, Haas, & Pohl, 2020 | nonRCT, 12, 12 | Immediate implant + immediate provisionalization (at sites with intact buccal plate) | Nobel Active (Nobel Biocare) | Maxilla, Incisor and canine | No, NR | No, No | Immediate provisionalization | NR |
| Proussaefs, Kan, Lozada, Kleinman, & Farnos, 2002 | nonRCT, 10, 10 | Implant placement + immediate provisionalization | Nobel Replace (Nobel Biocare) | Maxilla, Premolar | Yes, NR | No, No | Immediate provisionalization | Metal ceramic crown |
| Puisys et al., 2022 | RCT, 25, 25 | Immediate implant placement + CTG + immediate provisionalization | Bone Level Tapered SLActive (Straumann) | Maxilla, Incisor and canine | No, NR | Allograft, No | Immediate provisionalization | Zirconia crown |
| Puisys et al., 2022 | RCT, 25, 25 | Extraction + CTG. Early implant placement + GBR + delayed loading | Bone Level Tapered SLActive (Straumann) | Maxilla, Incisor and canine | Yes, NR | Autogenous bone graft mixed with allograft, No | Healing abutment | Zirconia crown |
| Qian et al., 2023 | nonRCT, 12, 12 | Immediate implant placement + GBR + CTG | NobelActive or NobelReplace Conical Connection (Nobel Biocare) | Maxilla, Anterior | Yes, NR | DBBM, CTG | Immediate provisionalization or healing abutment | NR |
| Raes et al., 2015 | nonRCT, 46, 46 | Implant placement + immediate provisionalization (in smokers) | Osseospeed (Astra Tech) | Maxilla, Ant and premolar | Yes and no (depending on the case), NR | No, No | Immediate provisionalization | Metal fused to porcelain restoration or full-ceramic restoration |
| Raes et al., 2015 | nonRCT, 39, 39 | Implant placement + immediate provisionalization (in non-smokers) | Osseospeed (Astra Tech) | Maxilla, Ant and premolar | Yes and no (depending on the case), NR | No, No | Immediate provisionalization | Metal fused to porcelain restoration or full-ceramic restoration |
| Raes et al., 2018 | nonRCT, 11, 11 | Immediate implant placement + immediate provisionalization | Osseospeed (Astra Tech) | Maxilla, Ant and premolar | Yes, NR | No, No | Immediate provisionalization | Ceramic crown |
| Raes et al., 2018 | nonRCT, 18, 18 | Conventional implant placement + immediate provisionalization | Osseospeed (Astra Tech) | Maxilla, Ant and premolar | Yes, NR | No, No | Immediate provisionalization | Ceramic crown |
| Raes, Cosyn, & De Bruyn, 2013 | nonRCT, 23, 23 | Conventional implant placement + immediate provisionalization | Osseospeed (Astra Tech) | Maxilla, Ant and premolar | Yes, NR | No, No | Immediate provisionalization | Ceramic crown |
| Raes, Cosyn, & De Bruyn, 2013 | nonRCT, 16, 16 | Immediate implant placement + immediate provisionalization | Osseospeed (Astra Tech) | Maxilla, Ant and premolar | Yes, NR | No, No | Immediate provisionalization | Ceramic crown |
| Raes, Cosyn, & De Bruyn, 2013 | nonRCT, 9, 9 | Staged GBR and Implant placement (after 4-5 months) | Osseospeed (Astra Tech) | Maxilla, Ant and premolar | Yes, NR | No, No | NR | Ceramic crown |
| Raes, Cosyn, Crommelinck, Coessens, & De Bruyn, 2011 | nonRCT, 23, 23 | Conventional implant placement + immediate provisionalization | Osseospeed (Astra Tech) | Maxilla, Ant and premolar | Yes, NR | No, No | Immediate provisionalization | Ceramic crown |
| Raes, Cosyn, Crommelinck, Coessens, & De Bruyn, 2011 | nonRCT, 16, 16 | Immediate implant placement + immediate provisionalization | Osseospeed (Astra Tech) | Maxilla, Ant and premolar | Yes, NR | No, No | Immediate provisionalization | Ceramic crown |
| Ribeiro dos Reis et al., 2023 | nonRCT, 7, 7 | Implant placement (in presence of STH < 3 mm) + immediate provisionalization | Nobel Active (Nobel Biocare) | Maxilla and mandible, NR | Yes, NR | No, No | Immediate provisionalization | NR |
| Ribeiro dos Reis et al., 2023 | nonRCT, 8, 8 | Implant placement (in presence of STH ≥ 3 mm) + immediate provisionalization | Nobel Active (Nobel Biocare) | Maxilla and mandible, NR | Yes, NR | No, No | Immediate provisionalization | NR |
| Rivara et al., 2020 | RCT, 15, 30 | Implant placement (2 mm of distance between the two implants) | Nobel Replace CC (Nobel Biocare) | Maxilla and mandible, Premolar | Yes, Yes | No, No | Healing abutment after the surgery, with provisional crown delivered after 24h | Zirconia crown |
| Rivara et al., 2020 | RCT, 15, 30 | Implant placement (3 mm of distance between the two implants) | Nobel Replace CC (Nobel Biocare) | Maxilla and mandible, Premolar | Yes, Yes | No, No | Healing abutment after the surgery, with provisional crown delivered after 24h | Zirconia crown |
| Rivara et al., 2020 | RCT, 15, 30 | Implant placement (2 mm of distance between the two implants) | Nobel Replace CC (Nobel Biocare) | Maxilla and mandible, Premolar | Yes, Yes | No, No | Healing abutment after the surgery, with provisional crown delivered after 24h | Zirconia crown |
| Rivara et al., 2020 | RCT, 15, 30 | Implant placement (3 mm of distance between the two implants) | Nobel Replace CC (Nobel Biocare) | Maxilla and mandible, Premolar | Yes, Yes | No, No | Healing abutment after the surgery, with provisional crown delivered after 24h | Zirconia crown |
| Romanos, Malmstrom, Feng, Ercoli, & Caton, 2014 | RCT, 17, 52 | Implant placement | Ankylos plus (Dentsply) | Mandible, Anterior | Yes, Yes | No, No | Immediate provisionalization | NR |
| Romanos, Malmstrom, Feng, Ercoli, & Caton, 2014 | RCT, 17, 52 | Implant placement | Certain Prevail (Biomet 3i) | Mandible, Anterior | Yes, Yes | No, No | Immediate provisionalization | NR |
| Romanos, Malmstrom, Feng, Ercoli, & Caton, 2014 | RCT, 15, 40 | Implant placement | Ankylos plus (Dentsply) | Mandible, Anterior | Yes, Yes | No, No | Immediate provisionalization | NR |
| Romanos, Malmstrom, Feng, Ercoli, & Caton, 2014 | RCT, 15, 40 | Implant placement | Certain Prevail (Biomet 3i) | Mandible, Anterior | Yes, Yes | No, No | Immediate provisionalization | NR |
| Salvi et al., 2020 | RCT, 19, 19 | Implant placement (implant with modified SLA transmucosal neck surface) | Tissue level standard plus SLActive with modified surface of the transmucosal neck (Straumann) | Maxilla and mandible, Posterior | Yes, NR | No, No | Healing abutment | NR |
| Salvi et al., 2020 | RCT, 19, 19 | Implant placement (implant with machined transmucosal neck surface) | Tissue level standard plus SLActive with machined surface of the transmucosal neck (Straumann) | Maxilla and mandible, Posterior | Yes, NR | No, No | Healing abutment | NR |
| Salvi et al., 2020 | RCT, 19, 19 | Implant placement (implant with modified SLA transmucosal neck surface) | Tissue level standard plus SLActive with modified surface of the transmucosal neck (Straumann) | Maxilla and mandible, Posterior | Yes, NR | No, No | Healing abutment | NR |
| Salvi et al., 2020 | RCT, 19, 19 | Implant placement (implant with machined transmucosal neck surface) | Tissue level standard plus SLActive with machined surface of the transmucosal neck (Straumann) | Maxilla and mandible, Posterior | Yes, NR | No, No | Healing abutment | NR |
| Santing, Raghoebar, Vissink, den Hartog, & Meijer, 2013 | nonRCT, 60, 60 | Implant placement | Bone Level (Straumann) | Maxilla, Anterior | Yes, NR | Autogenous and DBBM (when needed), No | Cover screw | Porcelain fused to metal crown |
| Sanz Martin, Benic, Hammerle, & Thoma, 2016 | RCT, 15, 15 | Implant placement (one-piece implant) | Straumann | NR, NR | Yes, NR | DBBM, No | NR | NR |
| Sanz Martin, Benic, Hammerle, & Thoma, 2016 | RCT, 18, 18 | implant placement (two-piece implant) | Branemark (Nobel Biocare) | NR, NR | Yes, NR | DBBM, No | NR | NR |
| Sanz-Martin, Encalada, Sanz-Sanchez, Aracil, & Sanz, 2019 | nonRCT, 12, 12 | Immediate implant placement (+ CMX) + immediate provisionalization | Nobel Active (Nobel Biocare) | Maxilla, Ant and premolar | No, NR | DBBM-C, CMX | Immediate provisionalization | NR |
| Sapata et al., 2018 | RCT, 17, 17 | Implant placement (one-piece implant) | Straumann | NR, NR | Yes, NR | DBBM, No | NR | NR |
| Sapata et al., 2018 | RCT, 16, 16 | implant placement (two-piece implant) | Branemark (Nobel Biocare) | NR, NR | Yes, NR | DBBM, No | NR | NR |
| Schropp & Isidor, 2008 | RCT, 34, 34 | Early or delayed implant placement | Osseotite implant (Biomet/3i) | Maxilla and mandible, Ant and premolar | Yes, NR | No, No | Cover screw | NR |
| Schropp & Isidor, 2008 | RCT, 18, 18 | Early implant placement | Osseotite implant (Biomet/3i) | Maxilla and mandible, Ant and premolar | Yes, NR | No, No | Cover screw | NR |
| Schropp & Isidor, 2008 | RCT, 16, 16 | Delayed implant placement | Osseotite implant (Biomet/3i) | Maxilla and mandible, Ant and premolar | Yes, NR | No, No | Cover screw | NR |
| Schropp & Isidor, 2008 | RCT, 18, 18 | Early implant placement | Osseotite implant (Biomet/3i) | Maxilla and mandible, Ant and premolar | Yes, NR | No, No | Cover screw | NR |
| Schropp & Isidor, 2008 | RCT, 16, 16 | Delayed implant placement | Osseotite implant (Biomet/3i) | Maxilla and mandible, Ant and premolar | Yes, NR | No, No | Cover screw | NR |
| Schrott, Jimenez, Hwang, Fiorellini, & Weber, 2009 | nonRCT, 58, 346 | Implant placement (KM ≥ 2 mm) | ITI Solid screw TPS surface (Straumann) | Mandible, Full arch | NR, NR | No, No | NR | Full arch screw-retained hybrid-type prostheses |
| Schrott, Jimenez, Hwang, Fiorellini, & Weber, 2009 | nonRCT, 58, 346 | Implant placement (KM ≥ 2 mm) | ITI Solid screw TPS surface (Straumann) | Mandible, Full arch | NR, NR | No, No | NR | Full arch screw-retained hybrid-type prostheses |
| Schrott, Jimenez, Hwang, Fiorellini, & Weber, 2009 | nonRCT, 58, 346 | Implant placement (KM ≥ 2 mm) | ITI Solid screw TPS surface (Straumann) | Mandible, Full arch | NR, NR | No, No | NR | Full arch screw-retained hybrid-type prostheses |
| Schrott, Jimenez, Hwang, Fiorellini, & Weber, 2009 | nonRCT, 58, 346 | Implant placement (KM ≥ 2 mm) | ITI Solid screw TPS surface (Straumann) | Mandible, Full arch | NR, NR | No, No | NR | Full arch screw-retained hybrid-type prostheses |
| Schrott, Jimenez, Hwang, Fiorellini, & Weber, 2009 | nonRCT, 58, 346 | Implant placement (KM ≥ 2 mm) | ITI Solid screw TPS surface (Straumann) | Mandible, Full arch | NR, NR | No, No | NR | Full arch screw-retained hybrid-type prostheses |
| Schrott, Jimenez, Hwang, Fiorellini, & Weber, 2009 | nonRCT, 58, 346 | Implant placement (KM ≥ 2 mm) | ITI Solid screw TPS surface (Straumann) | Mandible, Full arch | NR, NR | No, No | NR | Full arch screw-retained hybrid-type prostheses |
| Schrott, Jimenez, Hwang, Fiorellini, & Weber, 2009 | nonRCT, 58, 40 | Implant placement (KM < 2 mm) | ITI Solid screw TPS surface (Straumann) | Mandible, Full arch | NR, NR | No, No | NR | Full arch screw-retained hybrid-type prostheses |
| Schrott, Jimenez, Hwang, Fiorellini, & Weber, 2009 | nonRCT, 58, 40 | Implant placement (KM < 2 mm) | ITI Solid screw TPS surface (Straumann) | Mandible, Full arch | NR, NR | No, No | NR | Full arch screw-retained hybrid-type prostheses |
| Schrott, Jimenez, Hwang, Fiorellini, & Weber, 2009 | nonRCT, 58, 40 | Implant placement (KM < 2 mm) | ITI Solid screw TPS surface (Straumann) | Mandible, Full arch | NR, NR | No, No | NR | Full arch screw-retained hybrid-type prostheses |
| Schrott, Jimenez, Hwang, Fiorellini, & Weber, 2009 | nonRCT, 58, 40 | Implant placement (KM < 2 mm) | ITI Solid screw TPS surface (Straumann) | Mandible, Full arch | NR, NR | No, No | NR | Full arch screw-retained hybrid-type prostheses |
| Schrott, Jimenez, Hwang, Fiorellini, & Weber, 2009 | nonRCT, 58, 40 | Implant placement (KM < 2 mm) | ITI Solid screw TPS surface (Straumann) | Mandible, Full arch | NR, NR | No, No | NR | Full arch screw-retained hybrid-type prostheses |
| Schrott, Jimenez, Hwang, Fiorellini, & Weber, 2009 | nonRCT, 58, 40 | Implant placement (KM < 2 mm) | ITI Solid screw TPS surface (Straumann) | Mandible, Full arch | NR, NR | No, No | NR | Full arch screw-retained hybrid-type prostheses |
| Schwarz, Sahm, & Becker, 2012 | RCT, 8, 8 | Implant placement + GBR resulting in 0 mm of residual bone defect height after 4 months | Camlog Screw-Line Implant, Promote plus (Camlog) | Maxilla and mandible, NR | Yes, NR | DBBM, No | Cover screw | NR |
| Schwarz, Sahm, & Becker, 2012 | RCT, 8, 8 | Implant placement + GBR resulting in 1 mm of residual bone defect height after 4 months | Camlog Screw-Line Implant, Promote plus (Camlog) | Maxilla and mandible, NR | Yes, NR | DBBM, No | Cover screw | NR |
| Schwarz, Sahm, & Becker, 2012 | RCT, 8, 8 | Implant placement + GBR resulting in > 1 mm of residual bone defect height after 4 months | Camlog Screw-Line Implant, Promote plus (Camlog) | Maxilla and mandible, NR | Yes, NR | DBBM, No | Cover screw | NR |
| Schwarz, Schmucker, & Becker, 2017 | RCT, 10, 10 | Implant placement + GBR (with native collagen membrane) | Camlog Screw-Line Implant, Promote plus (Camlog) | Maxilla and mandible, NR | Yes, NR | DBBM, No | Cover screw | NR |
| Schwarz, Schmucker, & Becker, 2017 | RCT, 9, 9 | Implant placement + GBR (with cross-linked collagen membrane) | Camlog Screw-Line Implant, Promote plus (Camlog) | Maxilla and mandible, NR | Yes, NR | DBBM, No | Cover screw | NR |
| Seyssens, Eghbali, & Cosyn, 2020 | nonRCT, 12, 12 | Immediate implant placement + immediate provisionalization | NobelActive (Nobel Biocare) | Maxilla, Ant and premolar | No, NR | DBBM, No | Immediate provisionalization | Full-ceramic crown or metal-ceramic crown |
| Seyssens, Eghbali, & Cosyn, 2020 | nonRCT, 6, 6 | Immediate implant placement + immediate provisionalization + CTG after 3 months | NobelActive (Nobel Biocare) | Maxilla, Ant and premolar | No, NR | DBBM, CTG | Immediate provisionalization | Full-ceramic crown or metal-ceramic crown |
| Siegenthaler et al., 2022 | RCT, 16, 16 | Provisional crown with a concave contour | OsseoSpeed EV, Astra Tech, Dentsply Sirona | Maxilla and mandible, Ant and premolar | NR, NR | No, No | Healing abutment/cover screw | Zirconia crown |
| Siegenthaler et al., 2022 | RCT, 15, 15 | Provisional crown with a convex contour | OsseoSpeed EV, Astra Tech, Dentsply Sirona | Maxilla and mandible, Ant and premolar | NR, NR | No, No | Healing abutment/cover screw | Zirconia crown |
| Siegenthaler et al., 2022 | RCT, 16, 16 | Healing abutment, no provisional crown | OsseoSpeed EV, Astra Tech, Dentsply Sirona | Maxilla and mandible, Ant and premolar | NR, NR | No, No | Healing abutment/cover screw | Zirconia crown |
| Siegenthaler et al., 2022 | RCT, 15, 15 | Provisional crown with a convex contour | OsseoSpeed EV, Astra Tech, Dentsply Sirona | Maxilla and mandible, Ant and premolar | NR, NR | No, No | Healing abutment/cover screw | Zirconia crown |
| Siegenthaler et al., 2022 | RCT, 16, 16 | Provisional crown with a concave contour | OsseoSpeed EV, Astra Tech, Dentsply Sirona | Maxilla and mandible, Ant and premolar | NR, NR | No, No | Healing abutment/cover screw | Zirconia crown |
| Siegenthaler et al., 2022 | RCT, 16, 16 | Healing abutment, no provisional crown | OsseoSpeed EV, Astra Tech, Dentsply Sirona | Maxilla and mandible, Ant and premolar | NR, NR | No, No | Healing abutment/cover screw | Zirconia crown |
| Slagter, Meijer, Bakker, Vissink, & Raghoebar, 2015 | RCT, 20, 20 | Immediate implant placement + immediate provisionalization | NobelActive (Nobel Biocare) | Maxilla, Ant and premolar | No, NR | Autogenous and DBBM, No | Immediate provisionalization | Zirconia crown |
| Slagter, Meijer, Bakker, Vissink, & Raghoebar, 2015 | RCT, 20, 20 | Immediate implant placement + delayed provisionalization | NobelActive (Nobel Biocare) | Maxilla, Ant and premolar | No, NR | Autogenous and DBBM, Yes (de-epithelialized punch obtained at second stage) | Cover screw | Zirconia crown |
| Slagter, Meijer, Bakker, Vissink, & Raghoebar, 2016 | RCT, 20, 20 | Immediate implant placement | NobelActive (Nobel Biocare) | Maxilla, Ant and premolar | No, NR | Autogenous and DBBM, CTG (tuberosity) | Cover screw | NR |
| Slagter, Meijer, Bakker, Vissink, & Raghoebar, 2016 | RCT, 20, 20 | ARP + delayed implant placement | NobelActive (Nobel Biocare) | Maxilla, Ant and premolar | Yes, NR | No, No | Cover screw | NR |
| Slagter, Meijer, Hentenaar, Vissink, & Raghoebar, 2021 | RCT, 17, 17 | ARP + delayed implant placement | NobelActive (Nobel Biocare) | Maxilla, Ant and premolar | No, Yes | No, No | Cover screw | NR |
| Slagter, Meijer, Hentenaar, Vissink, & Raghoebar, 2021 | RCT, 18, 18 | Immediate implant placement | NobelActive (Nobel Biocare) | Maxilla, Ant and premolar | No, Yes | Autogenous and DBBM, CTG (tuberosity) | Cover screw | NR |
| Slagter, Raghoebar, Hentenaar, Vissink, & Meijer, 2021 | RCT, 18, 18 | Immediate implant placement + immediate provisionalization | NobelActive (Nobel Biocare) | Maxilla, Ant and premolar | No, Yes | Autogenous and DBBM, No | Immediate provisionalization | Zirconia crown |
| Slagter, Raghoebar, Hentenaar, Vissink, & Meijer, 2021 | RCT, 17, 17 | Immediate implant placement + delayed provisionalization | NobelActive (Nobel Biocare) | Maxilla, Ant and premolar | No, Yes | Autogenous and DBBM, Yes (de-epithelialized punch obtained at second stage) | Cover screw | Zirconia crown |
| Small & Tarnow, 2000 | nonRCT, 11, 65 | Implant placement | Nobel Biocare, Interpore International, Stryker, and ITI Straumann | Maxilla and mandible, Anterior and posterior | Yes, NR | No, No | NR | NR |
| Stefanini et al., 2016 | nonRCT, 20, 20 | Implant placement + CTG | Standard Plus (Straumann) | Maxilla and mandible, Premolar and molar | Yes, NR | No, CTG | Healing abutment | Metal-ceramic crown |
| Stefanini et al., 2016 | nonRCT, 20, 20 | Implant placement + CTG | Standard Plus (Straumann) | Maxilla and mandible, Premolar and molar | Yes, NR | No, CTG | Healing abutment | Metal-ceramic crown |
| Stoupel et al., 2016 | RCT, 18, 18 | Immediate implant placement and provisionalization (flapless) | Biomet 3i | Maxilla, Ant and premolar | No, NR | No, No | Immediate provisionalization | NR |
| Stoupel et al., 2016 | RCT, 20, 20 | Immediate implant placement and provisionalization (with a flap) | Biomet 3i | Maxilla, Ant and premolar | Yes, NR | No, No | Immediate provisionalization | NR |
| Stoupel et al., 2016 | RCT, 16, 16 | Immediate implant placement and provisionalization (flapless) | Biomet 3i | Maxilla, Ant and premolar | No, NR | No, No | Immediate provisionalization | NR |
| Stoupel et al., 2016 | RCT, 20, 20 | Immediate implant placement and provisionalization (with a flap) | Biomet 3i | Maxilla, Ant and premolar | Yes, NR | No, No | Immediate provisionalization | NR |
| Strasding et al., 2023 | RCT, 30, 30 | Immediate implant + immediate provisionalization | Bone Level Tapered (Straumann) | Maxilla and mandible, Ant and premolar | No, NR | NR, No | NR | All-ceramic crown |
| Strasding et al., 2023 | RCT, 30, 30 | Early implant placement + GBR | Bone Level Tapered (Straumann) | Maxilla and mandible, Ant and premolar | Yes, NR | Autogenous and DBBM, No | NR | All-ceramic crown |
| Sun et al., 2020 | RCT, 15, 15 | Immediate implant placement with Socket-shield approach | NobelReplace (Nobel Biocare) | Maxilla, Incisor and canine | No, Yes | DBBM (if gap > 1 mm), No | Immediate provisionalization | Zirconia crown |
| Sun et al., 2020 | RCT, 15, 15 | Immediate implant placement with Socket-shield approach | NobelReplace (Nobel Biocare) | Maxilla, Incisor and canine | No, Yes | DBBM (if gap > 1 mm), No | Immediate provisionalization | Zirconia crown |
| Sun et al., 2020 | RCT, 15, 15 | Immediate implant placement | NobelReplace (Nobel Biocare) | Maxilla, Incisor and canine | No, Yes | DBBM (if gap > 1 mm), No | Immediate provisionalization | Zirconia crown |
| Sun et al., 2020 | RCT, 15, 15 | Immediate implant placement | NobelReplace (Nobel Biocare) | Maxilla, Incisor and canine | No, Yes | DBBM (if gap > 1 mm), No | Immediate provisionalization | Zirconia crown |
| Takuma, Oishi, Manabe, Yoneda, & Nagata, 2014 | nonRCT, 30, 66 | Implant placement | Osseotite NT (Biomet 3i) | Maxilla and mandible, Posterior | Yes, NR | No, No | Healing abutment | NR |
| Tian et al., 2019 | nonRCT, 27, 27 | Immediate implant placement + immediate provisionalization | Nobel Active (Nobel Biocare) and Camlog Screw-line (Camlog) | Maxilla, Anterior | No, NR | DBBM and DBBM-C, No | Immediate provisionalization | NR |
| Todescan et al., 2023 | nonRCT, 20, 20 | Immediate implant placement + immediate provisionalization (in patients with thin soft tissue phenotype) | Nobel Active (Nobel Biocare) | Maxilla, Anterior | No, Yes | DBBM, No | Immediate provisionalization | NR |
| Todescan et al., 2023 | nonRCT, 6, 6 | Immediate implant placement + immediate provisionalization (in patients with thick soft tissue phenotype) | Nobel Active (Nobel Biocare) | Maxilla, Anterior | No, Yes | DBBM, No | Immediate provisionalization | NR |
| Tsuda et al., 2011 | nonRCT, 10, 10 | Immediate implant placement (+ CTG) + immediate provisionalization | Osseospeed (Astra Tech) | Maxilla, Anterior | No, NR | DBBM, CTG | Immediate provisionalization | All-ceramic crown |
| van Kesteren, Schoolfield, West, & Oates, 2010 | RCT, 13, 13 | Immediate implant placement | Sandblasted acid etched SLA (Straumann) | Maxilla and mandible, Ant and premolar | Yes, Yes | FDBA (if gap ≥ 2 mm), No | Healing abutment | NR |
| van Kesteren, Schoolfield, West, & Oates, 2010 | RCT, 13, 13 | ARP + delayed implant placement | Sandblasted acid etched SLA (Straumann) | Maxilla and mandible, Ant and premolar | Yes, Yes | No, No | Healing abutment | NR |
| van Nimwegen et al., 2018 | RCT, 30, 30 | Immediate implant placement and provisionalization | NobelActive (Nobel Biocare) | Maxilla, Ant and premolar | No, NR | Autogenous and DBBM, No | Immediate provisionalization | Zirconia crown |
| van Nimwegen et al., 2018 | RCT, 30, 30 | Immediate implant placement and provisionalization + CTG | NobelActive (Nobel Biocare) | Maxilla, Ant and premolar | No, NR | Autogenous and DBBM, CTG (tuberosity) | Immediate provisionalization | Zirconia crown |
| Vandeweghe, Cosyn, Thevissen, Van den Berghe, & De Bruyn, 2012 | nonRCT, 14, 15 | Implant placement + immediate provisionalization | Co-Axis (Southern Implants) | Maxilla, Anterior and premolar | Yes, NR | No, No | Immediate provisionalization | All-ceramic crown |
| Wanis, Hosny, & ElNahass, 2022 | RCT, 12, 12 | Immediate implant (dual zone approach) + immediate provisionalization | IS-II (NeoBiotech) | Maxilla, Ant and premolar | No, NR | Cortico-cancellous collagenated xenograft, No | Immediate provisionalization | Zirconia crown |
| Wanis, Hosny, & ElNahass, 2022 | RCT, 12, 12 | Immediate implant (dual zone approach) + immediate provisionalization | IS-II (NeoBiotech) | Maxilla, Ant and premolar | No, NR | Cortico-cancellous collagenated xenograft, No | Immediate provisionalization | Zirconia crown |
| Wanis, Hosny, & ElNahass, 2022 | RCT, 12, 12 | Immediate implant + immediate provisionalization | IS-II (NeoBiotech) | Maxilla, Ant and premolar | No, NR | Cortico-cancellous collagenated xenograft, No | Immediate provisionalization | Zirconia crown |
| Wanis, Hosny, & ElNahass, 2022 | RCT, 12, 12 | Immediate implant + immediate provisionalization | IS-II (NeoBiotech) | Maxilla, Ant and premolar | No, NR | Cortico-cancellous collagenated xenograft, No | Immediate provisionalization | Zirconia crown |
| Weber, Kim, Ng, Hwang, & Fiorellini, 2006 | nonRCT, 80 (in total), 93 | Implant placement and screw-retained restoration | ITI cylinder implant (Straumann) | Maxilla, Anterior | Yes, NR | No, No | NR | Porcelain-fused-to-ceramic crown |
| Weber, Kim, Ng, Hwang, & Fiorellini, 2006 | nonRCT, 80 (in total), 59 | Implant placement and cement-retained restoration | ITI cylinder implant (Straumann) | Maxilla, Anterior | Yes, NR | No, No | NR | Porcelain-fused-to-ceramic crown |
| Weber, Kim, Ng, Hwang, & Fiorellini, 2006 | nonRCT, 80 (in total), 93 | Implant placement and screw-retained restoration | ITI cylinder implant (Straumann) | Maxilla, Anterior | Yes, NR | No, No | NR | Porcelain-fused-to-ceramic crown |
| Weber, Kim, Ng, Hwang, & Fiorellini, 2006 | nonRCT, 80 (in total), 59 | Implant placement and cement-retained restoration | ITI cylinder implant (Straumann) | Maxilla, Anterior | Yes, NR | No, No | NR | Porcelain-fused-to-ceramic crown |
| Wohrle, 1998 | nonRCT, 14, 14 | Immediate implant placement + immediate provisionalization | Replace (Steri-Oss) | Maxilla, Incisor and lateral | No, NR | No, No | Immediate provisionalization | NR |
| Yang, Zhou, Zhou, & Man, 2019 | nonRCT, 16, 16 | Immediate implant + immediate provisionalization (in presence of buccal bone thickness < 0.5mm) | Nobel Active, Osstem, Apollo, and Dentium | Maxilla, Anterior | No, NR | Xenograft or synthetic graft, No | Immediate provisionalization | Porcelain crown |
| Yang, Zhou, Zhou, & Man, 2019 | nonRCT, 14, 20 | Immediate implant + immediate provisionalization (in presence of buccal bone thickness of 0.5-1mm) | Nobel Active, Osstem, Apollo, and Dentium | Maxilla, Anterior | No, NR | Xenograft or synthetic graft, No | Immediate provisionalization | Porcelain crown |
| Yang, Zhou, Zhou, & Man, 2019 | nonRCT, 14, 14 | Immediate implant + immediate provisionalization (in presence of buccal bone thickness ≥ 1mm) | Nobel Active, Osstem, Apollo, and Dentium | Maxilla, Anterior | No, NR | Xenograft or synthetic graft, No | Immediate provisionalization | Porcelain crown |
| Yoshino, Kan, Rungcharassaeng, Roe, & Lozada, 2014 | RCT, 10, 10 | Immediate implant placement (with CTG) + immediate provisionalization | Bone Level (Straumann) | Maxilla, Ant and premolar | No, NR | DBBM, CTG | Immediate provisionalization | Ceramic crown |
| Yoshino, Kan, Rungcharassaeng, Roe, & Lozada, 2014 | RCT, 10, 10 | Immediate implant placement + immediate provisionalization | Bone Level (Straumann) | Maxilla, Ant and premolar | No, NR | DBBM, No | Immediate provisionalization | Ceramic crown |
| Yoshino, Kan, Rungcharassaeng, Roe, & Lozada, 2014 | RCT, 10, 10 | Immediate implant placement (with CTG) + immediate provisionalization | Bone Level (Straumann) | Maxilla, Ant and premolar | No, NR | DBBM, CTG | Immediate provisionalization | Ceramic crown |
| Yoshino, Kan, Rungcharassaeng, Roe, & Lozada, 2014 | RCT, 10, 10 | Immediate implant placement + immediate provisionalization | Bone Level (Straumann) | Maxilla, Ant and premolar | No, NR | DBBM, No | Immediate provisionalization | Ceramic crown |
| Yuenyongorarn et al., 2020 | RCT, 10, 10 | Immediate implant placement (without socket grafting) + immediate provisionalization | SuperLine fixture (Dentium) | Maxilla, Anterior | No, NR | No, No | Immediate provisionalization | Metal ceramic crown |
| Yuenyongorarn et al., 2020 | RCT, 10, 10 | Immediate implant placement (with socket grafting) + immediate provisionalization | SuperLine fixture (Dentium) | Maxilla, Anterior | No, NR | DBBM, No | Immediate provisionalization | Metal ceramic crown |
| Zembic, Philipp, Hammerle, Wohlwend, & Sailer, 2015 | nonRCT, 16, 31 | Implant placement and restoration with zirconia abutment and all-ceramic crown | Branemark system (Nobel Biocare) | Maxilla and mandible, Anterior and premolar | NR, NR | NR, NR | Cover screw | All-ceramic crown |
| Zhang et al., 2017 | nonRCT, 16, 16 | Immediate implant (+ bone graft + PRF) + immediate provisionalization | NR | Maxilla, Anterior | No, NR | Yes, but type of bone graft not specified, No | Immediate provisionalization | Zirconia-ceramic crown |
| Zhang et al., 2017 | nonRCT, 16, 16 | Immediate implant (+ bone graft + PRF) + immediate provisionalization | NR | Maxilla, Anterior | No, NR | Yes, but type of bone graft not specified, No | Immediate provisionalization | Zirconia-ceramic crown |
| Zhang et al., 2017 | nonRCT, 16, 16 | Immediate implant (+ bone graft + PRF) + immediate provisionalization | NR | Maxilla, Anterior | No, NR | Yes, but type of bone graft not specified, No | Immediate provisionalization | Zirconia-ceramic crown |
| Zitzmann, Scharer, & Marinello, 2001 | RCT, 75, 112 | Implant placement + GBR (with DBBM and CM) | Branemark system (Nobel Biocare) | Maxilla and mandible, Anterior and posterior | Yes, NR | DBBM, No | Cover screw | NR |
| Zitzmann, Scharer, & Marinello, 2001 | RCT, 75, 41 | Implant placement + GBR (with DBBM and PTFE membrane) | Branemark system (Nobel Biocare) | Maxilla and mandible, Anterior and posterior | Yes, NR | DBBM, No | Cover screw | NR |
| Zitzmann, Scharer, & Marinello, 2001 | RCT, 75, 112 | Implant placement (without bone augmentation) | Branemark system (Nobel Biocare) | Maxilla and mandible, Anterior and posterior | Yes, NR | No, No | Cover screw | NR |
| Zuiderveld et al., 2021 | RCT, 28, 28 | Immediate implant placement (with CTG) + immediate provisionalization | NobelActive (Nobel Biocare) | Maxilla, Ant and premolar | Yes, Yes | Autogenous and DBBM, CTG (tuberosity) | Immediate provisionalization | NR |
| Zuiderveld et al., 2021 | RCT, 27, 27 | Immediate implant placement + immediate provisionalization | NobelActive (Nobel Biocare) | Maxilla, Ant and premolar | No, Yes | Autogenous and DBBM, No | Immediate provisionalization | NR |
| Zuiderveld, Meijer, den Hartog, Vissink, & Raghoebar, 2018 | RCT, 30, 30 | Immediate implant placement + immediate provisionalization | NobelActive (Nobel Biocare) | Maxilla, Ant and premolar | No, Yes | Autogenous and DBBM, No | Immediate provisionalization | NR |
| Zuiderveld, Meijer, den Hartog, Vissink, & Raghoebar, 2018 | RCT, 30, 30 | Immediate implant placement (with CTG) + immediate provisionalization | NobelActive (Nobel Biocare) | Maxilla, Ant and premolar | No, Yes | Autogenous and DBBM, CTG (tuberosity) | Immediate provisionalization | NR |
| Zuiderveld, Meijer, Vissink, & Raghoebar, 2018 | RCT, 20, 20 | ARP + Implant placement + CTG | Nobel Replace CC (Nobel Biocare) | Maxilla, Ant and premolar | Yes, Yes | No, CTG | Cover screw | NR |
| Zuiderveld, Meijer, Vissink, & Raghoebar, 2018 | RCT, 20, 20 | ARP + Implant placement | Nobel Replace CC (Nobel Biocare) | Maxilla, Ant and premolar | Yes, Yes | No, No | Cover screw | NR |
| Zuiderveld, Meijer, Vissink, & Raghoebar, 2018 | RCT, 20, 20 | ARP + Implant placement + CMX | Nobel Replace CC (Nobel Biocare) | Maxilla, Ant and premolar | Yes, Yes | No, CMX | Cover screw | NR |

**Legend**: CMX: collagen matrix; CTG: connective tissue graft; DBBM: deproteinized bovine bone mineral; DBBM-C: deproteinized bovine bone mineral particles embedded in collagen; FDBA: freeze-dried bone allograft; NR: not reported; RCT: randomized clinical trial

**Supplementary Table 8.** Incidence and amount/depth of Mucosal Level (ML) changes, peri-implant soft tissue dehiscence (PSTD), and mucosal recession (MREC).

| **Article** | **Intervention** | **Follow-up (months)** | **Mucosal level (ML) changes** | | **PSTD** | | **MREC** | |
| --- | --- | --- | --- | --- | --- | --- | --- | --- |
|  |  |  | **Incidence (%)** | **ML changes depth (mean ± SD) (mm)** | **Incidence (%)** | **PSTD depth (mean ± SD) (mm)** | **Incidence (%)** | **MREC depth (mean ± SD) (mm)** |
| Andersson, Odman, Lindvall, & Branemark, 1998 | Implant placement | 60 | 13.6 | NR | NR | NR | NR | NR |
| Arora & Ivanovski, 2017 | Immediate implant placement + immediate provisionalization | 24 | NR | 0.22 ± 0.83 | NR | NR | NR | NR |
| Arora & Ivanovski, 2017 | Immediate implant placement + immediate provisionalization | 12 | NR | 0.33 ± 0.84 | NR | NR | NR | NR |
| Arora & Ivanovski, 2018 | Immediate implant placement | 12 | 27 | 0.26 ± 1 | NR | NR | NR | NR |
| Arora & Ivanovski, 2018 | Early implant placement | 12 | 27 | 0.39 ± 1.01 | NR | NR | NR | NR |
| Arora, Khzam, Roberts, Bruce, & Ivanovski, 2017 | Immediate implant placement + immediate provisionalization | 60 | 13 | 0.23 ± 0.69 | NR | NR | NR | NR |
| Arora, Khzam, Roberts, Bruce, & Ivanovski, 2017 | Immediate implant placement + immediate provisionalization | 12 | NR | 0.2 ± 0.47 | NR | NR | NR | NR |
| Atef, El Barbary, Dahrous, & Zahran, 2021 | Immediate implant placement + socket shield | 6 | NR | NR | NR | NR | NR | NR |
| Atef, El Barbary, Dahrous, & Zahran, 2021 | Immediate implant placement | 6 | NR | NR | NR | NR | NR | NR |
| Atef, El Barbary, Dahrous, & Zahran, 2021 | Immediate implant placement + socket shield | 12 | NR | -0.45 | NR | NR | NR | NR |
| Atef, El Barbary, Dahrous, & Zahran, 2021 | Immediate implant placement | 12 | NR | 0.466 | NR | NR | NR | NR |
| Barone et al., 2016 | Implant placement (regular insertion torque) | 12 | NR | 0.2 ± NR | NR | NR | NR | NR |
| Barone et al., 2016 | Implant placement (high insertion torque) | 12 | NR | 0.88 ± NR | NR | NR | NR | NR |
| Barone, Toti, Quaranta, Derchi, & Covani, 2015 | Immediate implant placement + immediate provisionalization | 24 | NR | 0.5 ± 0.7 | NR | NR | NR | NR |
| Barone, Toti, Quaranta, Derchi, & Covani, 2015 | Immediate implant placement (delayed restoration) | 24 | NR | 0.3 ± 0.8 | NR | NR | NR | NR |
| Bengazi, Wennströnm & Lekholm, 1996 | Implant placement (in presence of alveolar mucosa) | 24 | NR | NR | NR | NR | 57 | 0.6 |
| Bengazi, Wennströnm & Lekholm, 1996 | Implant placement (in presence of KM) | 24 | NR | NR | NR | NR | 38 | 0.37 |
| Benic et al., 2012 | Immediate implant placement + GBR | 84 | NR | NR | NR | NR | 14.3 | 0.8 ± 0.9 |
| Benic et al., 2017 | Implant placement + GBR | 12 | NR | NR | NR | NR | NR | 1.17 ± 0.53 |
| Benic et al., 2017 | Implant placement (without bone augmentation) | 12 | NR | NR | NR | NR | NR | 0.64 ± 0.54 |
| Benic et al., 2017 | Implant placement + GBR | 36 | NR | 0.19 ± 0.31 | NR | NR | NR | 1 ± 0.62 |
| Benic et al., 2017 | Implant placement (without bone augmentation) | 36 | NR | 0.03 ± 0.31 | NR | NR | NR | 0.60 ± 0.67 |
| Benitez Silva et al., 2022 | ARP with DBBM, and implant placement after 4 months | 12 | NR | 0.08 ± 0.42 | NR | NR | NR | NR |
| Benitez Silva et al., 2022 | ARP with DBBM-C, and implant placement after 4 months | 12 | NR | 0.13 ± 0.54 | NR | NR | NR | NR |
| Bianchi & Sanfilippo, 2004 | Immediate implant placement + CTG | 24 | 0 | NR | NR | NR | NR | NR |
| Bianchi & Sanfilippo, 2004 | Immediate implant placement | 24 | 20 | NR | NR | NR | NR | NR |
| Bianchi & Sanfilippo, 2004 | Immediate implant placement + CTG | 54 | 2 | NR | NR | NR | NR | NR |
| Bianchi & Sanfilippo, 2004 | Immediate implant placement | 54 | 22.7 | NR | NR | NR | NR | NR |
| Bianchi & Sanfilippo, 2004 | Immediate implant placement + CTG | 90 | 4.5 | NR | NR | NR | NR | NR |
| Bianchi & Sanfilippo, 2004 | Immediate implant placement | 90 | 25.7 | NR | NR | NR | NR | NR |
| Bittner et al., 2019 and Bittner et al., 2020b | Immediate implant placement in patients with thin phenotype | 6 | NR | 1.99 ± 0.42 | NR | NR | NR | NR |
| Bittner et al., 2019 and Bittner et al., 2020b | Immediate implant placement (implant with anodized pink neck) in patients with thin phenotype | 6 | NR | 1.37 ± 1.05 | NR | NR | NR | NR |
| Bittner et al., 2019 and Bittner et al., 2020b | Immediate implant placement in patients with thick phenotype | 6 | NR | 1.13 ± 0.88 | NR | NR | NR | NR |
| Bittner et al., 2019 and Bittner et al., 2020b | Immediate implant placement (implant with anodized pink neck) in patients with thick phenotype | 6 | NR | 1.28 ± 0.79 | NR | NR | NR | NR |
| Bittner et al., 2020a | Immediate implant placement + immediate provisionalization with bone graft | 6 | NR | 0.8 ± 1.4 | NR | NR | NR | NR |
| Bittner et al., 2020a | Immediate implant placement + immediate provisionalization with bone graft | 12 | NR | 0.9 ± 1.2 | NR | NR | NR | NR |
| Bittner et al., 2020a | Immediate implant placement + immediate provisionalization | 6 | NR | 1.0 ± 1.1 | NR | NR | NR | NR |
| Bittner et al., 2020a | Immediate implant placement + immediate provisionalization | 12 | NR | 1.3 ± 1.5 | NR | NR | NR | NR |
| Blanes, Bernard, Blanes, & Belser, 2007 | Implant placement | 72 | NR | NR | NR | NR | NR | 0.33 ± 0.7 |
| Block et al., 2009 | ARP, delayed implant placement, and immediate provisionalization | 6 | NR | -0.052 | NR | NR | NR | NR |
| Block et al., 2009 | ARP, delayed implant placement, and immediate provisionalization | 12 | NR | 0.205 | NR | NR | NR | NR |
| Block et al., 2009 | ARP, delayed implant placement, and immediate provisionalization | 18 | NR | -0.84 | NR | NR | NR | NR |
| Block et al., 2009 | ARP, delayed implant placement, and immediate provisionalization | 24 | NR | -0.04 | NR | NR | NR | NR |
| Block et al., 2009 | Immediate implant placement + immediate provisionalization | 6 | NR | -0.033 | NR | NR | NR | NR |
| Block et al., 2009 | Immediate implant placement + immediate provisionalization | 12 | NR | 0.24 | NR | NR | NR | NR |
| Block et al., 2009 | Immediate implant placement + immediate provisionalization | 18 | NR | -0.13 | NR | NR | NR | NR |
| Block et al., 2009 | Immediate implant placement + immediate provisionalization | 24 | NR | -0.41 | NR | NR | NR | NR |
| Bonino et al., 2018 | Implant placement | 6 | NR | NR | NR | NR | 14.3 | 0.33 |
| Bragger, Hammerle, & Lang, 1996 | Immediate implant placement (with barrier membrane) | 12 | NR | NR | NR | NR | NR | 0.26 |
| Bragger, Hammerle, & Lang, 1996 | Immediate implant placement (without barrier membrane) | 12 | NR | NR | NR | NR | NR | 0.25 |
| Bragger, Hammerle, & Lang, 1996 | Implant placement | 12 | NR | NR | NR | NR | NR | 0.19 |
| Bressan et al., 2017 | Implant placement + definitive abutment | 36 | NR | 0.13 ± 0.76 | NR | NR | NR | NR |
| Bressan et al., 2017 | Implant placement + definitive abutment | 12 | NR | 0.07 ± 0.35 | NR | NR | NR | NR |
| Bressan et al., 2017 | Implant placement + repeated abutment changes | 36 | NR | 0.12 ± 1.15 | NR | NR | NR | NR |
| Bressan et al., 2017 | Implant placement + repeated abutment changes | 12 | NR | 0.12 ± 0.65 | NR | NR | NR | NR |
| Brunello et al., 2022 | Implant placement (zirconia implant) | 24 | NR | NR | NR | NR | NR | 0.1 ± 0.1 |
| Brunello et al., 2022 | Implant placement (zirconia implant) | 108 | NR | NR | NR | NR | 34.5 | 0.1 ± 0.2 |
| Buser et al., 2009 | Early implant placement + GBR | 12 | NR | NR | NR | 0.18 ± 0.58 | NR | NR |
| Buser et al., 2011 | Early implant placement + GBR | 36 | NR | NR | 5 | 0.09 ± 0.33 | NR | NR |
| Buser, Chappuis, Bornstein, et al., 2013 | Early implant placement + GBR | 84 | NR | 0.32 | NR | NR | NR | NR |
| Buser, Chappuis, Kuchler, et al., 2013 | Early implant placement + GBR | 72 | NR | 0.27 | NR | NR | NR | NR |
| Bushahri et al., 2021 | Immediate implant placement + immediate provisionalization | 30 | NR | 0.06 ± 1.18 | NR | NR | NR | NR |
| Bushahri et al., 2021 | Immediate implant placement + delayed restoration | 30 | NR | 0.38 ± 0.64 | NR | NR | NR | NR |
| Cabello, Rioboo, & Fabrega, 2013 | Immediate implant + immediate provisionalization | 12 | NR | 0.45 ± 0.3 | NR | NR | NR | NR |
| Canullo, Caneva, & Tallarico, 2017 | Immediate implant placement (platform switching) | 120 | NR | -0.23 | NR | NR | NR | NR |
| Canullo, Caneva, & Tallarico, 2017 | Immediate implant placement (non-platform switching) | 120 | NR | 0.59 | NR | NR | NR | NR |
| Canullo, Iurlaro, & Iannello, 2009 | Immediate implant placement (platform switching) | 25 | NR | -0.18 | NR | NR | NR | NR |
| Canullo, Iurlaro, & Iannello, 2009 | Immediate implant placement (non-platform switching) | 25 | NR | 0.45 | NR | NR | NR | NR |
| Cardaropoli, Gaveglio, Gherlone, & Cardaropoli, 2014 | Immediate implant placement + DBBM-C + CM | 12 | NR | 0.58 | NR | NR | NR | NR |
| Cardaropoli, Gaveglio, Gherlone, & Cardaropoli, 2014 | Immediate implant placement | 12 | NR | 1.69 | NR | NR | NR | NR |
| Cecchinato, Lops, Salvi, & Sanz, 2015 | Immediate implant placement (cylindrical implant) | 12 | NR | 0 ± 1 | NR | NR | NR | NR |
| Cecchinato, Lops, Salvi, & Sanz, 2015 | Immediate implant placement (cylindrical implant) | 24 | NR | 0.1 ± 0.9 | NR | NR | NR | NR |
| Cecchinato, Lops, Salvi, & Sanz, 2015 | Immediate implant placement (cylindrical implant) | 36 | 28 | 0 ± 1.1 | NR | NR | NR | NR |
| Cecchinato, Lops, Salvi, & Sanz, 2015 | Immediate implant placement (conical/cylindrical implant) | 12 | NR | 0.3 ± 1.6 | NR | NR | NR | NR |
| Cecchinato, Lops, Salvi, & Sanz, 2015 | Immediate implant placement (conical/cylindrical implant) | 24 | NR | 0.8 ± 1.4 | NR | NR | NR | NR |
| Cecchinato, Lops, Salvi, & Sanz, 2015 | Immediate implant placement (conical/cylindrical implant) | 36 | 11 | 0.5 ± 1.2 | NR | NR | NR | NR |
| Chan et al., 2019 | Immediate implant placement + immediate provisionalization | 12 | NR | 0.1 ± 0.9 | NR | NR | NR | NR |
| Chan et al., 2019 | Immediate implant placement + delayed restoration | 12 | NR | 0.1 ± 0.7 | NR | NR | NR | NR |
| Chen, Darby, & Reynolds, 2007 | Immediate implant placement (+ DBBM) | 12 | NR | NR | 30 | NR | NR | NR |
| Chen, Darby, & Reynolds, 2007 | Immediate implant placement (+ DBBM and CM) | 12 | NR | NR | 40 | NR | NR | NR |
| Chen, Darby, & Reynolds, 2007 | Immediate implant placement (without graft) | 12 | NR | NR | 30 | NR | NR | NR |
| Chung, Rungcharassaeng, Kan, Roe, & Lozada, 2011 | Immediate implant (+ CTG) + immediate provisionalization | 12 | 11.1 | 0.05 | NR | NR | NR | NR |
| Cooper et al., 2010 | Immediate implant placement + immediate provisionalization | 12 | 16.4 | 0.35 ± 0.89 | NR | NR | NR | NR |
| Cooper et al., 2010 | Delayed implant placement | 12 | 13 | 0.30 ± 0.76 | NR | NR | NR | NR |
| Cooper et al., 2014 | Immediate implant placement + immediate provisionalization | 60 | 34.9 | 0.06 ± 0.98 | NR | NR | NR | NR |
| Cooper et al., 2014 | Delayed implant placement | 60 | 17.4 | 0.42 ± 1.07 | NR | NR | NR | NR |
| Cooper et al., 2015 | implant placement with conus interface between abutment and implant | 9 | NR | 0.1 ± 0.7 | NR | NR | NR | NR |
| Cooper et al., 2015 | implant placement with flat-to-flat interface between abutment and implant | 9 | NR | 0.1 ± 0.7 | NR | NR | NR | NR |
| Cooper et al., 2015 | implant placement with platform switched interface between abutment and implant | 9 | NR | 0.3 ± 0.8 | NR | NR | NR | NR |
| Cooper et al., 2019 | implant placement with conus interface between abutment and implant | 36 | 20 | 0.2 ± 0.7 | NR | NR | NR | NR |
| Cooper et al., 2019 | implant placement with flat-to-flat interface between abutment and implant | 36 | 39 | 0 ± 1 | NR | NR | NR | NR |
| Cooper et al., 2019 | implant placement with platform switched interface between abutment and implant | 36 | 16 | 0.3 ± 0.7 | NR | NR | NR | NR |
| Cordaro, Torsello, & Roccuzzo, 2009 | Immediate implant placement (submerged) + delayed provisionalization | 6 | NR | 0.93 ± 0.62 | NR | NR | NR | NR |
| Cordaro, Torsello, & Roccuzzo, 2009 | Immediate implant placement (submerged) + delayed provisionalization | 12 | NR | 0.82 ± 0.67 | NR | NR | NR | NR |
| Cordaro, Torsello, & Roccuzzo, 2009 | Immediate implant placement (non-submerged) + delayed provisionalization | 6 | NR | 0.73 ± 0.7 | NR | NR | NR | NR |
| Cordaro, Torsello, & Roccuzzo, 2009 | Immediate implant placement (non-submerged) + delayed provisionalization | 12 | NR | 0.73 ± 0.7 | NR | NR | NR | NR |
| Cosyn et al., 2011 | Immediate implant placement | 12 | NR | 0.53 ± 0.76 | NR | NR | NR | NR |
| Cosyn et al., 2011 | Immediate implant placement | 36 | 8 | 0.34 ± 0.8 | NR | NR | NR | NR |
| Cosyn et al., 2016 | Immediate implant placement + immediate provisionalization | 12 | 31.8 | 0.23 ± NR | NR | NR | NR | NR |
| Cosyn et al., 2016 | Immediate implant placement + immediate provisionalization | 60 | 25 | 0.63 ± NR | NR | NR | NR | NR |
| Cosyn et al., 2016 | Immediate implant placement + immediate provisionalization + CTG after 3 months | 12 | NR | 0.21 ± NR | NR | NR | NR | NR |
| Cosyn et al., 2016 | Immediate implant placement + immediate provisionalization + CTG after 3 months | 60 | NR | 0.5 ± NR | NR | NR | NR | NR |
| Cosyn et al., 2022 | Implant placement + CTG | 12 | NR | NR | NR | NR | NR | 0.38 ± NR |
| Cosyn et al., 2022 | Implant placement + CMX | 12 | NR | NR | NR | NR | NR | 0.37 ± NR |
| Cosyn, De Bruyn, & Cleymaet, 2013 | Immediate implant placement + immediate provisionalization | 12 | NR | 0.2 ± 0.4 | NR | NR | NR | NR |
| Cosyn, De Bruyn, & Cleymaet, 2013 | Immediate implant placement + immediate provisionalization | 6 | NR | 0.3 ± 0.5 | NR | NR | NR | NR |
| Cosyn, Pollaris, Van der Linden, & De Bruyn, 2015 | ARP + delayed implant placement + CTG (at sites without midfacial recession) | 12 | NR | 0.1 ± 0.3 | NR | NR | NR | NR |
| Cosyn, Pollaris, Van der Linden, & De Bruyn, 2015 | ARP + CTG + delayed implant placement (at sites with midfacial recession) | 12 | NR | 0.9 ± 1 | NR | NR | NR | NR |
| Covani, Canullo, Toti, Alfonsi, & Barone, 2014 | Immediate implant placement | 60 | NR | 0.56 ± 0.69 | NR | NR | NR | NR |
| Crespi et al., 2019 | Immediate implant placement (≥ 2 mm KT) + immediate provisionalization | 24 | NR | NR | NR | NR | NR | -0.13 ± 0.07 |
| Crespi et al., 2019 | Immediate implant placement (≥ 2 mm KT) + immediate provisionalization | 24 | NR | NR | NR | NR | NR | -0.15 ± 0.08 |
| Crespi et al., 2019 | Immediate implant placement (≥ 2 mm KT) + immediate provisionalization | 60 | NR | NR | NR | NR | NR | -0.14 ±0.12 |
| Crespi et al., 2019 | Immediate implant placement (≥ 2 mm KT) + immediate provisionalization | 60 | NR | NR | NR | NR | NR | -0.15 ± 0.13 |
| Crespi et al., 2019 | Immediate implant placement (≥ 2 mm KT) + immediate provisionalization | 96 | NR | NR | NR | NR | NR | -0.14 ± 0.13 |
| Crespi et al., 2019 | Immediate implant placement (≥ 2 mm KT) + immediate provisionalization | 96 | NR | NR | NR | NR | NR | -0.16 ± 0.09 |
| Crespi et al., 2019 | Immediate implant placement (<2 mm KT) + immediate provisionalization | 24 | NR | NR | NR | NR | NR | 0.17 ± 0.11 |
| Crespi et al., 2019 | Immediate implant placement (<2 mm KT) + immediate provisionalization | 24 | NR | NR | NR | NR | NR | 0.13 ± 0.09 |
| Crespi et al., 2019 | Immediate implant placement (<2 mm KT) + immediate provisionalization | 60 | NR | NR | NR | NR | NR | 0.14 ± 0.1 |
| Crespi et al., 2019 | Immediate implant placement (<2 mm KT) + immediate provisionalization | 60 | NR | NR | NR | NR | NR | 0.16 ± 0.08 |
| Crespi et al., 2019 | Immediate implant placement (<2 mm KT) + immediate provisionalization | 96 | NR | NR | NR | NR | NR | 0.15 ± 0.09 |
| Crespi et al., 2019 | Immediate implant placement (<2 mm KT) + immediate provisionalization | 96 | NR | NR | NR | NR | NR | 0.17 ± 0.12 |
| Crespi, Cappare, & Gherlone, 2010a | Immediate implant placement + immediate provisionalization (in presence of KM > 2 mm) | 24 | NR | NR | NR | NR | NR | 0.23 ± 0.15 |
| Crespi, Cappare, & Gherlone, 2010a | Immediate implant placement + immediate provisionalization (in presence of KM > 2 mm) | 48 | NR | NR | NR | NR | NR | 0.24 ± 0.16 |
| Crespi, Cappare, & Gherlone, 2010a | Immediate implant placement + immediate provisionalization (in presence of KM < 2 mm) | 24 | NR | NR | NR | NR | NR | 1.03 ± 0.7 |
| Crespi, Cappare, & Gherlone, 2010a | Immediate implant placement + immediate provisionalization (in presence of KM < 2 mm) | 48 | NR | NR | NR | NR | NR | 1.30 ± 0.8 |
| Crespi, Cappare, & Gherlone, 2010b | Immediate implant placement (in sites without peri-apical lesion) | 12 | NR | NR | NR | NR | NR | 0.21 ± 0.13 |
| Crespi, Cappare, & Gherlone, 2010b | Immediate implant placement (in sites without peri-apical lesion) | 24 | NR | NR | NR | NR | NR | 0.74 ± 0.29 |
| Crespi, Cappare, & Gherlone, 2010b | Immediate implant placement (in sites with peri-apical lesion) | 12 | NR | NR | NR | NR | NR | 0.66 ± 0.28 |
| Crespi, Cappare, & Gherlone, 2010b | Immediate implant placement (in sites with peri-apical lesion) | 24 | NR | NR | NR | NR | NR | 0.69 ± 0.29 |
| Crespi, Cappare, Gherlone, & Romanos, 2012 | Immediate implant placement + immediate provisionalization | 12 | NR | NR | NR | NR | NR | 0.18 ± 0.11 |
| Crespi, Cappare, Gherlone, & Romanos, 2012 | Immediate implant placement + immediate provisionalization | 24 | NR | NR | NR | NR | NR | 0.22 ± 0.15 |
| Crespi, Cappare, Polizzi, & Gherlone, 2015 | Immediate implant placement (implant with short collar) + immediate provisionalization | 24 | NR | NR | NR | NR | 60 | 0.21 ± 0.17 |
| Crespi, Cappare, Polizzi, & Gherlone, 2015 | Immediate implant placement (implant with short collar) + immediate provisionalization | 36 | NR | NR | NR | NR | 60 | 0.20 ± 0.16 |
| Crespi, Cappare, Polizzi, & Gherlone, 2015 | Immediate implant placement (implant with long collar) + immediate provisionalization | 24 | NR | NR | NR | NR | 60 | 0.22 ± 0.1 |
| Crespi, Cappare, Polizzi, & Gherlone, 2015 | Immediate implant placement (implant with long collar) + immediate provisionalization | 36 | NR | NR | NR | NR | 60 | 0.23 ± 0.12 |
| D'Elia et al., 2017 | Implant placement + GBR | 12 | NR | NR | NR | NR | NR | 0.23 ± 0.34 |
| D'Elia et al., 2017 | Implant placement + CTG | 12 | NR | NR | NR | NR | NR | 0.35 ± 0.56 |
| da Rosa, Rosa, Francischone, & Sotto-Maior, 2014 | Immediate implant placement (+ CTG) + immediate provisionalization | 58 | NR | -0.06 | NR | NR | NR | NR |
| de Albornoz et al., 2014 | Restoration with zirconia abutment | 12 | NR | NR | NR | NR | NR | 0 ± 0 |
| de Albornoz et al., 2014 | Restoration with titanium abutment | 12 | NR | NR | NR | NR | NR | 0.04 ± 0.1 |
| De Bruyckere et al., 2020 | Implant placement + CTG | 12 | 26.3 | 0.22 ± 0.43 | NR | NR | NR | NR |
| De Bruyckere et al., 2020 | Implant placement + GBR | 12 | 47.3 | 0.27 ± 0.42 | NR | NR | NR | NR |
| De Bruyn et al., 2013 | Immediate implant placement + immediate provisionalization | 60 | NR | 0.23 ± 1.08 | NR | NR | NR | NR |
| De Bruyn et al., 2013 | Delayed implant placement | 60 | NR | 0.27 ± 1.03 | NR | NR | NR | NR |
| De Rouck, Collys, & Cosyn, 2008 | Immediate implant placement + immediate provisionalization | 12 | NR | 0.53 ± 0.76 | NR | NR | NR | NR |
| De Rouck, Collys, Wyn, & Cosyn, 2009 | Immediate implant placement + immediate provisionalization | 6 | NR | 0.47 ± 0.72 | NR | NR | NR | NR |
| De Rouck, Collys, Wyn, & Cosyn, 2009 | Immediate implant placement + immediate provisionalization | 12 | NR | 0.41 ± 0.75 | NR | NR | NR | NR |
| De Rouck, Collys, Wyn, & Cosyn, 2009 | Immediate implant placement with submerged healing and delayed restoration | 6 | NR | 1.16 ± 0.64 | NR | NR | NR | NR |
| De Rouck, Collys, Wyn, & Cosyn, 2009 | Immediate implant placement with submerged healing and delayed restoration | 12 | NR | 1.16 ± 0.66 | NR | NR | NR | NR |
| de Siqueira et al., 2017 | Implant placement (equicrestal) | 8 | NR | NR | NR | NR | NR | 0.30 ± 0.46 |
| de Siqueira et al., 2017 | Implant placement (subcrestal) | 8 | NR | NR | NR | NR | NR | 0.60 ± 0.52 |
| de Siqueira et al., 2020 | Implant placement (equicrestal) | 60 | NR | NR | NR | NR | NR | 1.14 ± 0.54 |
| de Siqueira et al., 2020 | Implant placement (subcrestal) | 60 | NR | NR | NR | NR | NR | 1.06 ± 0.35 |
| Degidi, Nardi, Daprile, & Piattelli, 2014 | Immediate implant placement + immediate provisionalization (and conventional final impression) | 6 | NR | 0.32 ± 0.16 | NR | NR | NR | NR |
| Degidi, Nardi, Daprile, & Piattelli, 2014 | Immediate implant placement + immediate provisionalization (and conventional final impression) | 12 | NR | 0.57 ± 0.17 | NR | NR | NR | NR |
| Degidi, Nardi, Daprile, & Piattelli, 2014 | Immediate implant placement + immediate provisionalization (and conventional final impression) | 24 | NR | 0.59 ± 0.21 | NR | NR | NR | NR |
| Degidi, Nardi, Daprile, & Piattelli, 2014 | Immediate implant placement + immediate provisionalization (and final impression without abutment removal) | 6 | NR | 0.32 ± 0.13 | NR | NR | NR | NR |
| Degidi, Nardi, Daprile, & Piattelli, 2014 | Immediate implant placement + immediate provisionalization (and final impression without abutment removal) | 12 | NR | 0.33 ± 0.12 | NR | NR | NR | NR |
| Degidi, Nardi, Daprile, & Piattelli, 2014 | Immediate implant placement + immediate provisionalization (and final impression without abutment removal) | 24 | NR | 0.35 ± 0.12 | NR | NR | NR | NR |
| den Hartog, Raghoebar, Stellingsma, Vissink, & Meijer, 2011 | Immediate implant placement + immediate provisionalization | 18 | NR | 0.06 ± 0.42 | NR | NR | NR | NR |
| den Hartog, Raghoebar, Stellingsma, Vissink, & Meijer, 2011 | Conventional Implant placement | 18 | NR | 0.09 ± 0.34 | NR | NR | NR | NR |
| Eghbali et al., 2018 | ARP + implant placement + CTG (3 months after implant placement) | 60 | NR | 0.12 ± 0.36 | NR | NR | NR | NR |
| Eghbali et al., 2018 | ARP + implant placement + CTG (3 months after implant placement) | 12 | NR | 0.05 ± 0.27 | NR | NR | NR | NR |
| Ekfeldt, Eriksson, & Johansson, 2003 | Implant placement (maxilla only) | 12 | 81 | 0.9 ± 0.1 | NR | NR | NR | NR |
| Ekfeldt, Eriksson, & Johansson, 2003 | Implant placement (mandible only) | 12 | 94 | 1.5 ± 0.7 | NR | NR | NR | NR |
| Esposito et al., 2017 | Implant placement + transmucosal abutment | 12 | NR | 0.12 ± 0.65 | NR | NR | NR | NR |
| Esposito et al., 2017 | Implant placement + definitive abutment + immediate provisionalization | 12 | NR | 0.07 ± 0.35 | NR | NR | NR | NR |
| Farrag & Khamis, 2023 | Implant placement + anodized titanium abutment collar | 12 | NR | 0.25 ± 0.4 | NR | NR | NR | NR |
| Farrag & Khamis, 2023 | Implant placement + unanodized titanium abutment collar | 12 | NR | 0.25 ± 0.44 | NR | NR | NR | NR |
| Farrag & Khamis, 2023 | Implant placement + anodized titanium abutment collar | 18 | NR | 0.27 ± 0.52 | NR | NR | NR | NR |
| Farrag & Khamis, 2023 | Implant placement + unanodized titanium abutment collar | 18 | NR | 0.27 ± 0.44 | NR | NR | NR | NR |
| Farronato et al., 2020 | Implant placement | 12 | NR | 0.61 | NR | NR | NR | NR |
| Farronato et al., 2020 | Implant placement | 24 | NR | 1.11 | NR | NR | NR | NR |
| Farronato et al., 2020 | Implant placement | 36 | NR | 1.43 | NR | NR | NR | NR |
| Farronato et al., 2020 | Implant placement | 12 | NR | 0.33 | NR | NR | NR | NR |
| Farronato et al., 2020 | Implant placement | 24 | NR | 0.33 | NR | NR | NR | NR |
| Farronato et al., 2020 | Implant placement | 36 | NR | 0.1 | NR | NR | NR | NR |
| Farronato et al., 2021 | Implant placement (platform-switching) | 12 | NR | 0.11 ± 0.5 | NR | NR | NR | NR |
| Farronato et al., 2021 | Implant placement (non-platform-switching) | 12 | NR | 0.32 ± 0.6 | NR | NR | NR | NR |
| Farronato et al., 2021 | Implant placement (platform-switching) | 24 | NR | 0.11 ± 0.8 | NR | NR | NR | NR |
| Farronato et al., 2021 | Implant placement (non-platform-switching) | 24 | NR | 0.62 ± 1.4 | NR | NR | NR | NR |
| Farronato et al., 2021 | Implant placement (platform-switching) | 36 | NR | 0.17 ± 0.96 | NR | NR | NR | NR |
| Farronato et al., 2021 | Implant placement (non-platform-switching) | 36 | NR | 0.54 ± 1.39 | NR | NR | NR | NR |
| Fenner, Hammerle, Sailer, & Jung, 2016 | Implant placement (and all-ceramic crowns on aluminium oxide-based abutments) | 86.4 | NR | NR | NR | NR | NR | 0.31 ± 0.47 |
| Fenner, Hammerle, Sailer, & Jung, 2016 | Implant placement (and metal abutments on porcelain-fused-to-metal crowns) | 86.4 | NR | NR | NR | NR | NR | 0.29 ± 0.47 |
| Fernandes, Marques, Borges, & Montero, 2023 | Immediate implant placement | 12 | NR | 0.60 ± 0.72 | NR | NR | NR | NR |
| Fernandes, Marques, Borges, & Montero, 2023 | Immediate implant placement + CTG | 12 | NR | 0.38 ± 0.45 | NR | NR | NR | NR |
| Ferrari, Cagidiaco, Garcia-Godoy, Goracci, & Cairo, 2015 | Implant placement and restoration with titanium abutment | 24 | 13.4 | NR | NR | NR | NR | NR |
| Ferrari, Cagidiaco, Garcia-Godoy, Goracci, & Cairo, 2015 | Implant placement and restoration with titanium nitride abutment | 24 | 13.4 | NR | NR | NR | NR | NR |
| Ferrari, Cagidiaco, Garcia-Godoy, Goracci, & Cairo, 2015 | Implant placement and restoration with zirconia abutment | 24 | 13.4 | NR | NR | NR | NR | NR |
| Finelle, Popelut, Knafo, & Sanz Martin, 2021 | Immediate implant placement + socket seal abutment | 24 | NR | 0.53 ± 0.35 | NR | NR | NR | NR |
| Frizzera et al., 2019 | Immediate implant placement and immediate provisionalization | 6 | NR | NR | NR | 0.41 ± 0.4 | NR | NR |
| Frizzera et al., 2019 | Immediate implant placement (+ CMX) and immediate provisionalization | 6 | NR | NR | NR | 0.14 ± 0.37 | NR | NR |
| Frizzera et al., 2019 | Immediate implant placement (+ CTG) and immediate provisionalization | 6 | NR | NR | NR | -0.41 ± 0.75 | NR | NR |
| Frizzera et al., 2019 | Immediate implant placement and immediate provisionalization | 12 | NR | NR | NR | 0.72 ± 0.57 | NR | NR |
| Frizzera et al., 2019 | Immediate implant placement (+ CTG) and immediate provisionalization | 12 | NR | NR | NR | -0.04 ± 0.3 | NR | NR |
| Frizzera et al., 2019 | Immediate implant placement (+ CMX) and immediate provisionalization | 12 | NR | NR | NR | 0.42 ± 0.6 | NR | NR |
| Furhauser et al., 2017 | Immediate implant + immediate provisionalization | 24 | NR | NR | 40 | NR | NR | NR |
| Furhauser et al., 2017 | Immediate implant + immediate provisionalization | 36 | NR | NR | 41 | NR | NR | NR |
| Furhauser et al., 2017 | Immediate implant + immediate provisionalization | 48 | NR | NR | 40 | NR | NR | NR |
| Furhauser et al., 2017 | Immediate implant + immediate provisionalization | 60 | NR | 0.3 ± 1 | 40 | NR | NR | NR |
| Furhauser et al., 2017 | Immediate implant + immediate provisionalization | 12 | NR | 0.2 ± 0.8 | 40 | NR | NR | NR |
| Gallucci, Grutter, Nedir, Bischof, & Belser, 2011 | Implant placement and all-ceramic restoration | 12 | NR | 0.44 ± NR | NR | NR | NR | NR |
| Gallucci, Grutter, Nedir, Bischof, & Belser, 2011 | Implant placement and porcelain-fused-to-ceramic restoration | 12 | NR | 0.18 ± NR | NR | NR | NR | NR |
| Gallucci, Grutter, Nedir, Bischof, & Belser, 2011 | Implant placement and all-ceramic restoration | 24 | NR | 0.22 ± NR | NR | NR | NR | NR |
| Gallucci, Grutter, Nedir, Bischof, & Belser, 2011 | Implant placement and porcelain-fused-to-ceramic restoration | 24 | NR | -0.48 ± NR | NR | NR | NR | NR |
| Garaicoa-Pazmino et al., 2021 | Implant placement (STH ≤ 2 mm) | 6 | NR | NR | NR | NR | NR | 0.03 ± 0.06 |
| Garaicoa-Pazmino et al., 2021 | Implant placement (STH > 2 mm) | 6 | NR | NR | NR | NR | NR | 0 ± 0 |
| Garaicoa-Pazmino et al., 2021 | Implant placement (STH ≤ 2 mm) | 12 | NR | NR | NR | NR | NR | 0.03 ± 0.16 |
| Garaicoa-Pazmino et al., 2021 | Implant placement (STH > 2 mm) | 12 | NR | NR | NR | NR | NR | 0 ± 0 |
| Ghallab et al., 2023 | Immediate implant placement (vestibular extraction technique) | 12 | NR | 0.39 ± 0.64 | NR | NR | NR | NR |
| Ghallab et al., 2023 | Immediate implant placement (conventional extraction technique) | 12 | NR | 0.32 ± 0.68 | NR | NR | NR | NR |
| Girlanda et al., 2019 | Immediate implant placement (with DBBM-C) and immediate provisionalization | 6 | NR | 0 | NR | NR | NR | NR |
| Girlanda et al., 2019 | Immediate implant placement (without bone graft) and immediate provisionalization | 6 | NR | 1 | NR | NR | NR | NR |
| Gomez-Meda et al., 2022 | Immediate implant placement (+ CTG) | 12 | NR | NR | NR | -0.08 ± 1.69 | NR | NR |
| Gomez-Meda et al., 2022 | Immediate implant placement (+ socket shield) | 12 | NR | NR | NR | -0.01 ± 1.18 | NR | NR |
| Grandi, Guazzi, Samarani, & Grandi, 2013 | Immediate implant placement + immediate provisionalization | 12 | 47.8 | NR | NR | NR | NR | NR |
| Grandi, Guazzi, Samarani, & Grandi, 2013 | ARP + delayed implant placement (flapless) | 12 | 16.7 | NR | NR | NR | NR | NR |
| Grassi et al., 2015 | Implant placement (zirconia implant) + immediate provisionalization | 60 | NR | 0.3 | NR | 0.31 | NR | NR |
| Groenendijk, Bronkhorst, & Meijer, 2021 | Immediate implant + immediate provisionalization | 12 | NR | NR | 26 | NR | NR | NR |
| Guarnieri et al., 2022 | Implant placement (submerged) | 60 | NR | NR | NR | NR | NR | 0.7 ± 0.3 |
| Guarnieri et al., 2022 | Implant placement (nonsubmerged) | 60 | NR | NR | NR | NR | NR | 0.6 ± 0.2 |
| Guarnieri, Ceccarelli, Ricci, & Testori, 2018 | Implant placement (implants with laser-microtextured collar surface) | 120 | NR | NR | NR | NR | NR | 1.08 ± 0.4 |
| Guarnieri, Ceccarelli, Ricci, & Testori, 2018 | Implant placement (implants with machined collar surface) | 120 | NR | NR | NR | NR | NR | 2.46 ± 0.3 |
| Guarnieri, Ceccherini, & Grande, 2015 | Immediate implant placement and early loading | 60 | NR | NR | 30 | NR | 5 | 0.10 ± 0.61 |
| Guarnieri, Di Nardo, Di Giorgio, Miccoli, & Testarelli, 2019 | Implant placement (submerged) | 36 | NR | NR | NR | NR | NR | 0.5 ± 0.2 |
| Guarnieri, Di Nardo, Di Giorgio, Miccoli, & Testarelli, 2019 | Implant placement (nonsubmerged) | 36 | NR | NR | NR | NR | NR | 0.6 ± 0.3 |
| Guarnieri, Di Nardo, Gaimari, Miccoli, & Testarelli, 2019 | Implant placement (short implant) | 36 | NR | NR | NR | NR | NR | 0.15 ± 0.2 |
| Guarnieri, Di Nardo, Gaimari, Miccoli, & Testarelli, 2019 | Implant placement (standard implant) | 36 | NR | NR | NR | NR | NR | 0.18 ± 0.9 |
| Hall et al., 2007 | Implant placement with delayed restoration | 12 | NR | 0.33 ± 0.78 | NR | NR | NR | NR |
| Hall et al., 2007 | Implant placement with immediate provisionalization | 12 | NR | 0.67 ± 0.49 | NR | NR | NR | NR |
| Hattingh, De Bruyn, Van Weehaeghe, Hommez, & Vandeweghe, 2020 | Immediate implant placement (ultra-wide implants in molar sites) | 12 | 44.4 | 0.59 ± 1.37 | NR | NR | NR | NR |
| Hof et al., 2015 | Implant placement + GBR | 46 | NR | NR | 47 | NR | NR | NR |
| Hof et al., 2015 | Delayed implant placement | 58 | NR | NR | 23 | NR | NR | NR |
| Hof et al., 2015 | Immediate implant placement | 56 | NR | NR | 35 | NR | NR | NR |
| Hof et al., 2015 | Early implant placement | 54 | NR | NR | 34 | NR | NR | NR |
| Hof et al., 2015 | Autogenous bone grafting + delayed implant placement | 42 | NR | NR | 40 | NR | NR | NR |
| Hollander et al., 2016 | Implant placement (zirconia implant) | 14 | NR | NR | NR | NR | NR | 0.14 ± 0.55 |
| Hosseini et al., 2015 | Immediate implant placement + immediate provisionalization (with antibiotic therapy) | 6 | NR | 0.43 ± 0.76 | NR | NR | NR | NR |
| Hosseini et al., 2015 | Immediate implant placement + immediate provisionalization (without antibiotic therapy) | 6 | NR | 1.70 ± 1.06 | NR | NR | NR | NR |
| Humm et al., 2023 | Ceramic restoration with zirconia abutment | 156 | NR | NR | NR | NR | NR | 1.0 ± 1.78 |
| Humm et al., 2023 | Metal ceramic crown with titanium abutment | 156 | NR | NR | NR | NR | NR | 0.85 ± 1.99 |
| Huynh-Ba et al., 2019 | Immediate implant placement | 12 | 65 | 1.03 ± 0.24 | NR | NR | NR | NR |
| Huynh-Ba et al., 2019 | Early implant placement with GBR | 12 | 80 | 1.37 ± 0.28 | NR | NR | NR | NR |
| Iorio-Siciliano et al., 2016 | Implant placement | 24 | NR | NR | NR | NR | 0 | 0 |
| Jacobs, Zadeh, De Kok, & Cooper, 2020 | Immediate implant placement (+ DBBM) | 9.7 | NR | 0.94 ± 1.13 | NR | NR | NR | NR |
| Jacobs, Zadeh, De Kok, & Cooper, 2020 | Immediate implant placement (without bone graft) | 9.7 | NR | 0.92 ± 0.67 | NR | NR | NR | NR |
| Jeffcoat, McGlumphy, Reddy, Geurs, & Proskin, 2003 | Implant placement | 60 | NR | NR | NR | NR | NR | 2.11 |
| Jeffcoat, McGlumphy, Reddy, Geurs, & Proskin, 2003 | Implant placement | 60 | NR | NR | NR | NR | NR | 1.97 |
| Jeffcoat, McGlumphy, Reddy, Geurs, & Proskin, 2003 | Implant placement | 60 | NR | NR | NR | NR | NR | 2.03 |
| Jemt, Ahlberg, Henriksson, & Bondevik, 2006 | Implant placement | 180 | 80.9 | NR | 80.9 | 0.6 ± 1.04 | NR | NR |
| Jung et al., 2016 | Implant placement (zirconia implant) + immediate provisionalization | 12 | NR | 0 | NR | NR | NR | 0.7 ± 2 |
| Kan, Rungcharassaeng, & Lozada, 2003 | Immediate implant placement + immediate provisionalization | 12 | NR | 0.55 ± 0.53 | NR | NR | NR | NR |
| Kan, Rungcharassaeng, Lozada, & Zimmerman, 2011 | Immediate implant placement + immediate provisionalization | 48 | NR | 1.13 ± 0.87 | NR | NR | NR | NR |
| Kan, Rungcharassaeng, Sclar, & Lozada, 2007 | Immediate implant placement + immediate provisionalization | 12 | 34.8 | NR | NR | NR | NR | NR |
| Karoussis et al., 2004 | Implant placement | 120 | NR | NR | NR | NR | NR | 0.55 |
| Kobayashi et al., 2020 | Implant placement + GBR | 12 | NR | 0.64 ± 0.42 | NR | NR | NR | NR |
| Kobayashi et al., 2020 | Implant placement + GBR + CTG (at second stage) | 12 | NR | 0.09 ± 0.3 | NR | NR | NR | NR |
| Koh et al., 2011 | Immediate implant placement (at the level of the crest) | 6 | NR | 0.3 ± 0.4 | NR | NR | NR | NR |
| Koh et al., 2011 | Immediate implant placement (subcrestal) | 6 | NR | 0.4 ± 0.4 | NR | NR | NR | NR |
| Lago, da Silva, Gude, & Rilo, 2017 | Implant placement | 48 | NR | 0.53 | NR | NR | NR | NR |
| Lee et al., 2020 | Immediate implant placement (flapless) and immediate provisionalization | 12 | NR | 0.08 ± 0.15 | NR | NR | NR | NR |
| Lee et al., 2020 | Immediate implant placement (with a flap) and immediate provisionalization | 12 | NR | 0.25 ± 0.36 | NR | NR | NR | NR |
| Lee et al., 2023 | Immediate implant placement + CTG | 12 | 7.14 | 0.07 ± 1 | NR | NR | NR | NR |
| Lee et al., 2023 | Immediate implant placement + ADM | 12 | 20 | 0.67 ± 0.96 | NR | NR | NR | NR |
| Lee et al., 2023 | Immediate implant placement | 12 | 7.14 | 0.18 ± 1.08 | NR | NR | NR | NR |
| Lilet et al., 2022 | Immediate implant placement + socket seal abutment | 12 | NR | 0.07 ± 0.55 | NR | NR | NR | NR |
| Lindeboom, Tjiook, & Kroon, 2006 | Immediate implant placement | 12 | NR | NR | 39 | NR | NR | NR |
| Lindeboom, Tjiook, & Kroon, 2006 | Delayed implant placement | 12 | NR | NR | 16 | NR | NR | NR |
| Liu et al., 2019 | Immediate implant placement + GBR | 12 | 44.4 | 0.59 ± 0.71 | 44.4 | NR | NR | NR |
| Lops et al., 2015 | Implant placement and restoration with zirconia stock abutment | 24 | NR | 0.3 ± 0.3 | NR | NR | NR | NR |
| Lops et al., 2015 | Implant placement and restoration with titanium stock abutment | 24 | NR | 0.3 ± 0.4 | NR | NR | NR | NR |
| Lops et al., 2015 | Implant placement and restoration with zirconia cad-cam abutment | 24 | NR | 0.1 ± 0.3 | NR | NR | NR | NR |
| Lops et al., 2015 | Implant placement and restoration with titanium cad-dam abutment | 24 | NR | -0.3 ± 0.4 | NR | NR | NR | NR |
| Lops, Romeo, Chiapasco, Procopio, & Oteri, 2013 | Immediate implant placement | 12 | 47.6 | 0.18 ± 0.57 | NR | NR | NR | NR |
| Lorenz et al., 2019 | Implant placement (zirconia implant) | 93.6 | NR | NR | NR | NR | NR | 0.43 ± 0.8 |
| Lorenz et al., 2022 | Implant placement (zirconia implant) | 15 | NR | NR | NR | NR | 0 | 0 |
| Lowy et al., 2019 | Implant placement (platform-matched implant) | 12 | NR | NR | NR | 0.9 ± 0.8 | NR | NR |
| Lowy et al., 2019 | Implant placement (platform-switched implant) | 12 | NR | NR | NR | 0.1 ± 0.3 | NR | NR |
| Malchiodi, Cucchi, Ghensi, & Nocini, 2013 | Immediate implant placement + immediate provisionalization | 36 | NR | NR | 53.1 | 0.5 ± 0.6 | NR | NR |
| Marconcini et al., 2018 | Implant placement (regular insertion torque) | 12 | NR | 0.19 | NR | NR | NR | NR |
| Marconcini et al., 2018 | Implant placement (high insertion torque) | 12 | NR | 1.04 | NR | NR | NR | NR |
| Marconcini et al., 2018 | Implant placement (regular insertion torque) | 36 | NR | 0.2 | NR | NR | NR | NR |
| Marconcini et al., 2018 | Implant placement (high insertion torque) | 36 | NR | 1.1 | NR | NR | NR | NR |
| Mau et al., 2019 | Early implant placement with FDBA | 12 | NR | 0 ± 0.21 | NR | NR | NR | NR |
| Mau et al., 2019 | Early implant placement with autogenous graft + DBBM | 12 | NR | 0.02 ± 0.22 | NR | NR | NR | NR |
| Meijndert et al., 2017 | Bone augmentation with autogenous bone graft and implant placement (after 3 months) | 120 | NR | 0.10 ± 0.68 | NR | NR | NR | NR |
| Meijndert et al., 2017 | Bone augmentation with autogenous bone graft + collagen membrane and implant placement (after 3 months) | 120 | NR | 0.46 ± 1 | NR | NR | NR | NR |
| Meijndert et al., 2017 | Bone augmentation with DBBM + collagen membrane and implant placement (after 6 months) | 120 | NR | 0.47 ± 0.79 | NR | NR | NR | NR |
| Meijndert, Raghoebar, Meijer, & Vissink, 2008 | Bone augmentation with autogenous bone graft and implant placement (after 3 months) | 12 | NR | 0.02 ± 0.32 | NR | NR | NR | NR |
| Meijndert, Raghoebar, Meijer, & Vissink, 2008 | Bone augmentation with autogenous bone graft + collagen membrane and implant placement (after 3 months) | 12 | NR | 0.06 ± 0.39 | NR | NR | NR | NR |
| Meijndert, Raghoebar, Meijer, & Vissink, 2008 | Bone augmentation with DBBM + collagen membrane and implant placement (after 6 months) | 12 | NR | 0.27 ± 0.94 | NR | NR | NR | NR |
| Meijndert, Raghoebar, Vissink, & Meijer, 2022 | Implant placement + GBR | 6 | NR | 0.14 ± 0.4 | NR | NR | NR | NR |
| Migliorati, Amorfini, Signori, Biavati, & Benedicenti, 2015 | Immediate implant placement + CTG | 12 | NR | NR | NR | 0.13 | NR | NR |
| Migliorati, Amorfini, Signori, Biavati, & Benedicenti, 2015 | Immediate implant placement | 12 | NR | NR | NR | 0.5 | NR | NR |
| Migliorati, Amorfini, Signori, Biavati, & Benedicenti, 2015 | Immediate implant placement + CTG | 24 | NR | NR | 26.6 | 0.33 | NR | NR |
| Migliorati, Amorfini, Signori, Biavati, & Benedicenti, 2015 | Immediate implant placement | 24 | NR | NR | 52.2 | 0.65 | NR | NR |
| Mizuno, Nakano, Shimomoto, Fujita, & Ishigaki, 2022 | Immediate implant + GBR | 12 | NR | 0.5 ± 0.5 | NR | NR | NR | NR |
| Molina, Sanz-Sanchez, Martin, Blanco, & Sanz, 2017 | Implant placement (with healing cap) | 6 | NR | 0.18 ± 1.03 | NR | NR | NR | NR |
| Molina, Sanz-Sanchez, Martin, Blanco, & Sanz, 2017 | Implant placement (with definitive abutment) | 6 | NR | 0.31 ± 0.65 | NR | NR | NR | NR |
| Molina, Sanz-Sanchez, Martin, Blanco, & Sanz, 2017 | Implant placement (with healing cap) | 12 | NR | 0.24 ± 1 | NR | NR | NR | NR |
| Molina, Sanz-Sanchez, Martin, Blanco, & Sanz, 2017 | Implant placement (with definitive abutment) | 12 | NR | 0.55 ± 0.85 | NR | NR | NR | NR |
| Munoz-Camara, Gilbel-Del Aguila, Pardo-Zamora, & Camacho-Alonso, 2020 | Immediate implant placement + immediate provisionalization (at sites with peri-apical pathology) | 12 | NR | 0.35 ± 0.41 | NR | NR | NR | NR |
| Munoz-Camara, Gilbel-Del Aguila, Pardo-Zamora, & Camacho-Alonso, 2020 | Immediate implant placement + immediate provisionalization (at sites without peri-apical pathology) | 12 | NR | 0.22 ± 0.32 | NR | NR | NR | NR |
| Oates, West, Jones, Kaiser, & Cochran, 2002 | Implant placement | 24 | 61 | NR | NR | NR | 61 | 0.6 |
| Parvini, Muller, Cafferata, Schwarz, & Obreja, 2022 | Immediate implant placement + immediate provisionalization | 12 | NR | NR | NR | NR | NR | 0 |
| Parvini, Muller, Cafferata, Schwarz, & Obreja, 2022 | Delayed implant placement + immediate provisionalization | 12 | NR | NR | NR | NR | NR | 0 ± 0.06 |
| Perez et al., 2020 | Immediate implant placement + customized healing abutment | 12 | NR | 0.2 ± 0.4 | NR | NR | NR | NR |
| Perez et al., 2020 | Immediate implant placement + standard healing abutment | 12 | NR | 0.1 ± 0.5 | NR | NR | NR | NR |
| Pieri, Aldini, Marchetti, & Corinaldesi, 2011 | Immediate implant placement + immediate provisionalization (abutment with morse connection and platform switch) | 12 | NR | 0.61 ± 0.54 | NR | NR | NR | NR |
| Pieri, Aldini, Marchetti, & Corinaldesi, 2011 | Immediate implant placement + immediate provisionalization (abutment with internal connection and matching diameter) | 12 | NR | 0.73 ± 0.52 | NR | NR | NR | NR |
| Pieri, Aldini, Marchetti, & Corinaldesi, 2013 | Staged bone augmentation and delayed implant placement | 60 | NR | 1.12 ± 0.4 | NR | NR | NR | NR |
| Pohl, Furhauser, Haas, & Pohl, 2020 | Immediate implant + immediate provisionalization (at sites with buccal bone dehiscence) | 12 | NR | 0.35 | NR | 0.61 | NR | NR |
| Pohl, Furhauser, Haas, & Pohl, 2020 | Immediate implant + immediate provisionalization (at sites with intact buccal plate) | 12 | NR | 0.58 | NR | 0.74 | NR | NR |
| Proussaefs, Kan, Lozada, Kleinman, & Farnos, 2002 | Implant placement + immediate provisionalization | 12 | NR | 0.43 | NR | NR | NR | NR |
| Puisys et al., 2022 | Immediate implant placement + CTG + immediate provisionalization | 12 | NR | NR | NR | 0 ± 0.2 | NR | NR |
| Puisys et al., 2022 | Extraction + CTG. Early implant placement + GBR + delayed loading | 12 | NR | NR | NR | 0 ± 0.38 | NR | NR |
| Qian et al., 2023 | Immediate implant placement + GBR + CTG | 12 | NR | NR | NR | 0.03 ± 0.17 | NR | NR |
| Raes et al., 2015 | Implant placement + immediate provisionalization (in smokers) | 24 | NR | 0.09 ± 0.82 | NR | NR | NR | NR |
| Raes et al., 2015 | Implant placement + immediate provisionalization (in non-smokers) | 24 | NR | -0.53 ± 0.89 | NR | NR | NR | NR |
| Raes et al., 2018 | Immediate implant placement + immediate provisionalization | 96 | 9.1 | 0.21 | 54.5 | NR | NR | NR |
| Raes et al., 2018 | Conventional implant placement + immediate provisionalization | 96 | 0 | 1.01 | 27.8 | NR | NR | NR |
| Raes, Cosyn, & De Bruyn, 2013 | Conventional implant placement + immediate provisionalization | 13 | 43 | 1 ± 1.15 | NR | NR | NR | NR |
| Raes, Cosyn, & De Bruyn, 2013 | Immediate implant placement + immediate provisionalization | 13 | 7 | 0.12 ± 0.78 | NR | NR | NR | NR |
| Raes, Cosyn, & De Bruyn, 2013 | Staged GBR and Implant placement (after 4-5 months) | 13 | 22 | 0.49 ± 0.82 | NR | NR | NR | NR |
| Raes, Cosyn, Crommelinck, Coessens, & De Bruyn, 2011 | Conventional implant placement + immediate provisionalization | 13 | 43 | 1 ± 1.15 | NR | NR | NR | NR |
| Raes, Cosyn, Crommelinck, Coessens, & De Bruyn, 2011 | Immediate implant placement + immediate provisionalization | 13 | 7 | 0.12 ± 0.78 | NR | NR | NR | NR |
| Ribeiro dos Reis et al., 2023 | Implant placement (in presence of STH < 3 mm) + immediate provisionalization | 12 | NR | 0.47 ± 0.57 | NR | NR | NR | NR |
| Ribeiro dos Reis et al., 2023 | Implant placement (in presence of STH ≥ 3 mm) + immediate provisionalization | 12 | NR | 0.19 ± 0.41 | NR | NR | NR | NR |
| Rivara et al., 2020 | Implant placement (2 mm of distance between the two implants) | 6 | NR | NR | NR | NR | NR | 0.14 ± NR |
| Rivara et al., 2020 | Implant placement (3 mm of distance between the two implants) | 6 | NR | NR | NR | NR | NR | 0.39 ± NR |
| Rivara et al., 2020 | Implant placement (2 mm of distance between the two implants) | 12 | NR | NR | NR | NR | NR | 0.66 ± NR |
| Rivara et al., 2020 | Implant placement (3 mm of distance between the two implants) | 12 | NR | NR | NR | NR | NR | 0.38 ± NR |
| Romanos, Malmstrom, Feng, Ercoli, & Caton, 2014 | Implant placement (Ankylos plus, Dentsply) | 12 | NR | NR | NR | NR | NR | 0.39 ± 0.73 |
| Romanos, Malmstrom, Feng, Ercoli, & Caton, 2014 | Implant placement (Certain Prevail, Biomet 3i) | 12 | NR | NR | NR | NR | NR | 0.55 ± 0.67 |
| Romanos, Malmstrom, Feng, Ercoli, & Caton, 2014 | Implant placement (Ankylos plus, Dentsply) | 24 | NR | NR | NR | NR | NR | 0.25 ± 0.66 |
| Romanos, Malmstrom, Feng, Ercoli, & Caton, 2014 | Implant placement (Certain Prevail, Biomet 3i) | 24 | NR | NR | NR | NR | NR | 0.38 ± 0.57 |
| Salvi et al., 2020 | Implant placement (implant with modified SLA transmucosal neck surface) | 12 | NR | NR | NR | NR | NR | 1.1 |
| Salvi et al., 2020 | Implant placement (implant with machined transmucosal neck surface) | 12 | NR | NR | NR | NR | NR | 0.8 |
| Salvi et al., 2020 | Implant placement (implant with modified SLA transmucosal neck surface) | 36 | NR | NR | NR | NR | NR | 1.3 |
| Salvi et al., 2020 | Implant placement (implant with machined transmucosal neck surface) | 36 | NR | NR | NR | NR | NR | 0.9 |
| Santing, Raghoebar, Vissink, den Hartog, & Meijer, 2013 | Implant placement | 18 | NR | 0.04 ± 0.29 | NR | NR | NR | NR |
| Sanz Martin, Benic, Hammerle, & Thoma, 2016 | Implant placement (one-piece implant) | 12 | NR | 0.17 ± 0.58 | NR | NR | NR | NR |
| Sanz Martin, Benic, Hammerle, & Thoma, 2016 | implant placement (two-piece implant) | 12 | NR | 0.02 ± 0.32 | NR | NR | NR | NR |
| Sanz-Martin, Encalada, Sanz-Sanchez, Aracil, & Sanz, 2019 | Immediate implant placement (+ CMX) + immediate provisionalization | 6 | 50 | 0.29 ± 0.37 | NR | NR | NR | NR |
| Sapata et al., 2018 | Implant placement (one-piece implant) | 60 | NR | 0.22 | NR | NR | NR | NR |
| Sapata et al., 2018 | implant placement (two-piece implant) | 60 | NR | 0.09 | NR | NR | NR | NR |
| Schropp & Isidor, 2008 | Early or delayed implant placement | 54 | NR | NR | NR | NR | 14.7 | NR |
| Schropp & Isidor, 2008 | Early implant placement | 24 | NR | NR | 11.6 | NR | NR | NR |
| Schropp & Isidor, 2008 | Delayed implant placement | 24 | NR | NR | 12.1 | NR | NR | NR |
| Schropp & Isidor, 2008 | Early implant placement | 60 | NR | NR | 17.2 | NR | NR | NR |
| Schropp & Isidor, 2008 | Delayed implant placement | 60 | NR | NR | 12.1 | NR | NR | NR |
| Schrott, Jimenez, Hwang, Fiorellini, & Weber, 2009 | Implant placement (KM ≥ 2 mm) | 12 | NR | NR | NR | NR | NR | 0.07 |
| Schrott, Jimenez, Hwang, Fiorellini, & Weber, 2009 | Implant placement (KM ≥ 2 mm) | 18 | NR | NR | NR | NR | NR | 0.07 |
| Schrott, Jimenez, Hwang, Fiorellini, & Weber, 2009 | Implant placement (KM ≥ 2 mm) | 24 | NR | NR | NR | NR | NR | 0.13 |
| Schrott, Jimenez, Hwang, Fiorellini, & Weber, 2009 | Implant placement (KM ≥ 2 mm) | 36 | NR | NR | NR | NR | NR | 0.03 |
| Schrott, Jimenez, Hwang, Fiorellini, & Weber, 2009 | Implant placement (KM ≥ 2 mm) | 48 | NR | NR | NR | NR | NR | 0.09 |
| Schrott, Jimenez, Hwang, Fiorellini, & Weber, 2009 | Implant placement (KM ≥ 2 mm) | 60 | NR | NR | NR | NR | NR | 0.15 |
| Schrott, Jimenez, Hwang, Fiorellini, & Weber, 2009 | Implant placement (KM < 2 mm) | 12 | NR | NR | NR | NR | NR | 0.8 |
| Schrott, Jimenez, Hwang, Fiorellini, & Weber, 2009 | Implant placement (KM < 2 mm) | 18 | NR | NR | NR | NR | NR | 0.87 |
| Schrott, Jimenez, Hwang, Fiorellini, & Weber, 2009 | Implant placement (KM < 2 mm) | 24 | NR | NR | NR | NR | NR | 0.88 |
| Schrott, Jimenez, Hwang, Fiorellini, & Weber, 2009 | Implant placement (KM < 2 mm) | 36 | NR | NR | NR | NR | NR | 0.88 |
| Schrott, Jimenez, Hwang, Fiorellini, & Weber, 2009 | Implant placement (KM < 2 mm) | 48 | NR | NR | NR | NR | NR | 0.97 |
| Schrott, Jimenez, Hwang, Fiorellini, & Weber, 2009 | Implant placement (KM < 2 mm) | 60 | NR | NR | NR | NR | NR | 0.78 |
| Schwarz, Sahm, & Becker, 2012 | Implant placement + GBR resulting in 0 mm of residual bone defect height after 4 months | 48 | NR | NR | NR | NR | NR | 0.2 ± 0.3 |
| Schwarz, Sahm, & Becker, 2012 | Implant placement + GBR resulting in 1 mm of residual bone defect height after 4 months | 48 | NR | NR | NR | NR | NR | 0.5 ± 0.7 |
| Schwarz, Sahm, & Becker, 2012 | Implant placement + GBR resulting in > 1 mm of residual bone defect height after 4 months | 48 | NR | NR | NR | NR | NR | 0.4 ± 0.6 |
| Schwarz, Schmucker, & Becker, 2017 | Implant placement + GBR (with native collagen membrane) | 96 | NR | NR | NR | NR | NR | 0.3 ± 0.1 |
| Schwarz, Schmucker, & Becker, 2017 | Implant placement + GBR (with cross-linked collagen membrane) | 96 | NR | NR | NR | NR | NR | 0.1 ± 0.2 |
| Seyssens, Eghbali, & Cosyn, 2020 | Immediate implant placement + immediate provisionalization | 120 | 50 | NR | NR | NR | NR | NR |
| Seyssens, Eghbali, & Cosyn, 2020 | Immediate implant placement + immediate provisionalization + CTG after 3 months | 120 | 0 | NR | NR | NR | NR | NR |
| Siegenthaler et al., 2022 | Provisional crown with a concave contour | 12 | 14.3 | 1.00 ± 0 | NR | NR | NR | NR |
| Siegenthaler et al., 2022 | Provisional crown with a convex contour | 6 | 53.8 | 0.83 ± 0.4 | NR | NR | NR | NR |
| Siegenthaler et al., 2022 | Healing abutment, no provisional crown | 6 | 21.4 | 0.83 ± 0.28 | NR | NR | NR | NR |
| Siegenthaler et al., 2022 | Provisional crown with a convex contour | 12 | 64.3 | 0.72 ± 0.60 | NR | NR | NR | NR |
| Siegenthaler et al., 2022 | Provisional crown with a concave contour | 6 | 7.7 | 1.00 ± 0 | NR | NR | NR | NR |
| Siegenthaler et al., 2022 | Healing abutment, no provisional crown | 12 | 31.4 | 0.90 ± 0.65 | NR | NR | NR | NR |
| Slagter, Meijer, Bakker, Vissink, & Raghoebar, 2015 | Immediate implant placement + immediate provisionalization | 12 | NR | 0.95 ± 0.62 | NR | NR | NR | NR |
| Slagter, Meijer, Bakker, Vissink, & Raghoebar, 2015 | Immediate implant placement + delayed provisionalization | 12 | NR | 0.85 ± 0.86 | NR | NR | NR | NR |
| Slagter, Meijer, Bakker, Vissink, & Raghoebar, 2016 | Immediate implant placement | 12 | NR | 0.15 ± 0.28 | NR | NR | NR | NR |
| Slagter, Meijer, Bakker, Vissink, & Raghoebar, 2016 | ARP + delayed implant placement | 12 | NR | 0.34 ± 0.55 | NR | NR | NR | NR |
| Slagter, Meijer, Hentenaar, Vissink, & Raghoebar, 2021 | ARP + delayed implant placement | 60 | NR | 0.45 ± 0.59 | NR | NR | NR | NR |
| Slagter, Meijer, Hentenaar, Vissink, & Raghoebar, 2021 | Immediate implant placement | 60 | NR | 0.25 ± 0.57 | NR | NR | NR | NR |
| Slagter, Raghoebar, Hentenaar, Vissink, & Meijer, 2021 | Immediate implant placement + immediate provisionalization | 60 | NR | 1.44 ± 0.98 | NR | NR | NR | NR |
| Slagter, Raghoebar, Hentenaar, Vissink, & Meijer, 2021 | Immediate implant placement + delayed provisionalization | 60 | NR | 0.81 ± 1.01 | NR | NR | NR | NR |
| Small & Tarnow, 2000 | Implant placement | 12 | 82 | 0.88 ± 0.75 | NR | NR | NR | NR |
| Stefanini et al., 2016 | Implant placement + CTG | 12 | 0 | -0.3 ± NR | NR | NR | NR | NR |
| Stefanini et al., 2016 | Implant placement + CTG | 36 | 0 | -0.75 ± NR | NR | NR | NR | NR |
| Stoupel et al., 2016 | Immediate implant placement and provisionalization (flapless) | 6 | NR | 0.19 ± 0.30 | NR | NR | NR | NR |
| Stoupel et al., 2016 | Immediate implant placement and provisionalization (with a flap) | 6 | NR | 0.40 ± 0.60 | NR | NR | NR | NR |
| Stoupel et al., 2016 | Immediate implant placement and provisionalization (flapless) | 12 | NR | 0.22 ± 0.31 | NR | NR | NR | NR |
| Stoupel et al., 2016 | Immediate implant placement and provisionalization (with a flap) | 12 | NR | 0.42 ± 0.52 | NR | NR | NR | NR |
| Strasding et al., 2023 | Immediate implant + immediate provisionalization | 12 | NR | 0.51 ± 0.43 | NR | NR | NR | NR |
| Strasding et al., 2023 | Early implant placement + GBR | 12 | NR | 0.54 ± 36 | NR | NR | NR | NR |
| Sun et al., 2020 | Immediate implant placement with Socket-shield approach | 12 | NR | 1.09 ± 0.22 | NR | NR | NR | NR |
| Sun et al., 2020 | Immediate implant placement with Socket-shield approach | 6 | NR | 0.87 ± 0.19 | NR | NR | NR | NR |
| Sun et al., 2020 | Immediate implant placement | 6 | NR | 0.30 ± 0.07 | NR | NR | NR | NR |
| Sun et al., 2020 | Immediate implant placement | 12 | NR | 0.59 ± 0.09 | NR | NR | NR | NR |
| Takuma, Oishi, Manabe, Yoneda, & Nagata, 2014 | Implant placement | 12 | NR | NR | NR | NR | NR | 0.39 ± 0.14 |
| Tian et al., 2019 | Immediate implant placement + immediate provisionalization | 12 | NR | 0.51 ± 0.45 | NR | 0.51 ± 0.45 | NR | NR |
| Todescan et al., 2023 | Immediate implant placement + immediate provisionalization (in patients with thin soft tissue phenotype) | 6 | 0 | 0.01 ± 0.4 | NR | NR | NR | NR |
| Todescan et al., 2023 | Immediate implant placement + immediate provisionalization (in patients with thick soft tissue phenotype) | 6 | 0 | 0.03 ± 0.22 | NR | NR | NR | NR |
| Tsuda et al., 2011 | Immediate implant placement (+ CTG) + immediate provisionalization | 12 | NR | 0.05 | NR | NR | NR | NR |
| van Kesteren, Schoolfield, West, & Oates, 2010 | Immediate implant placement | 6 | NR | 0.05 ± NR | NR | NR | NR | NR |
| van Kesteren, Schoolfield, West, & Oates, 2010 | ARP + delayed implant placement | 6 | NR | 0.28 ± NR | NR | NR | NR | NR |
| van Nimwegen et al., 2018 | Immediate implant placement and provisionalization | 12 | 32 | 0.48 ± 1.13 | NR | NR | NR | NR |
| van Nimwegen et al., 2018 | Immediate implant placement and provisionalization + CTG | 12 | 8 | -0.20 ± 0.70 | NR | NR | NR | NR |
| Vandeweghe, Cosyn, Thevissen, Van den Berghe, & De Bruyn, 2012 | Implant placement + immediate provisionalization | 12 | NR | 0.37 ± 0.39 | NR | NR | NR | NR |
| Wanis, Hosny, & ElNahass, 2022 | Immediate implant (dual zone approach) + immediate provisionalization | 12 | NR | 0.27 ± 0.34 | NR | NR | NR | NR |
| Wanis, Hosny, & ElNahass, 2022 | Immediate implant (dual zone approach) + immediate provisionalization | 6 | NR | 0.18 ± 0.34 | NR | NR | NR | NR |
| Wanis, Hosny, & ElNahass, 2022 | Immediate implant + immediate provisionalization | 12 | NR | 0.45 ± 0.44 | NR | NR | NR | NR |
| Wanis, Hosny, & ElNahass, 2022 | Immediate implant + immediate provisionalization | 6 | NR | 0.30 ± 0.26 | NR | NR | NR | NR |
| Weber, Kim, Ng, Hwang, & Fiorellini, 2006 | Implant placement and screw-retained restoration | 12 | NR | NR | NR | NR | NR | 1.07 ± 0.96 |
| Weber, Kim, Ng, Hwang, & Fiorellini, 2006 | Implant placement and cement-retained restoration | 12 | NR | NR | NR | NR | NR | 1.22 ± 1.15 |
| Weber, Kim, Ng, Hwang, & Fiorellini, 2006 | Implant placement and screw-retained restoration | 36 | NR | NR | NR | NR | NR | 1.05 ± 0.93 |
| Weber, Kim, Ng, Hwang, & Fiorellini, 2006 | Implant placement and cement-retained restoration | 36 | NR | NR | NR | NR | NR | 1.24 ± 1.31 |
| Wohrle, 1998 | Immediate implant placement + immediate provisionalization | 21.7 | 14.3 | NR | NR | NR | NR | NR |
| Yang, Zhou, Zhou, & Man, 2019 | Immediate implant + immediate provisionalization (in presence of buccal bone thickness < 0.5mm) | 12 | NR | 0.61 ± 1.02 | NR | NR | NR | NR |
| Yang, Zhou, Zhou, & Man, 2019 | Immediate implant + immediate provisionalization (in presence of buccal bone thickness of 0.5-1mm) | 12 | NR | -0.06 ± 0.37 | NR | NR | NR | NR |
| Yang, Zhou, Zhou, & Man, 2019 | Immediate implant + immediate provisionalization (in presence of buccal bone thickness ≥ 1mm) | 12 | NR | -0.30 ± 0.88 | NR | NR | NR | NR |
| Yoshino, Kan, Rungcharassaeng, Roe, & Lozada, 2014 | Immediate implant placement (with CTG) + immediate provisionalization | 6 | NR | 0.4 ± 0.52 | NR | NR | NR | NR |
| Yoshino, Kan, Rungcharassaeng, Roe, & Lozada, 2014 | Immediate implant placement + immediate provisionalization | 6 | NR | 0.75 ± 0.54 | NR | NR | NR | NR |
| Yoshino, Kan, Rungcharassaeng, Roe, & Lozada, 2014 | Immediate implant placement (with CTG) + immediate provisionalization | 12 | NR | 0.25 ± 0.35 | NR | NR | NR | NR |
| Yoshino, Kan, Rungcharassaeng, Roe, & Lozada, 2014 | Immediate implant placement + immediate provisionalization | 12 | NR | 0.7 ± 0.48 | NR | NR | NR | NR |
| Yuenyongorarn et al., 2020 | Immediate implant placement (without socket grafting) + immediate provisionalization | 12 | NR | 1.35 | NR | NR | NR | NR |
| Yuenyongorarn et al., 2020 | Immediate implant placement (with socket grafting) + immediate provisionalization | 12 | NR | 0.77 | NR | NR | NR | NR |
| Zembic, Philipp, Hammerle, Wohlwend, & Sailer, 2015 | Implant placement and restoration with zirconia abutment and all-ceramic crown | 132 | NR | NR | NR | NR | NR | 0.2 ± 1.1 |
| Zhang et al., 2017 | Immediate implant (+ bone graft + PRF) + immediate provisionalization | 12 | NR | 0.31 ± 0.13 | NR | NR | NR | NR |
| Zhang et al., 2017 | Immediate implant (+ bone graft + PRF) + immediate provisionalization | 24 | NR | 0.31 ± 0.13 | NR | NR | NR | NR |
| Zhang et al., 2017 | Immediate implant (+ bone graft + PRF) + immediate provisionalization | 36 | NR | 0.39 ± 0.15 | NR | NR | NR | NR |
| Zitzmann, Scharer, & Marinello, 2001 | Implant placement + GBR (with DBBM and CM) | 60 | NR | NR | NR | NR | 3.72 | 0.05 ± 0.93 |
| Zitzmann, Scharer, & Marinello, 2001 | Implant placement + GBR (with DBBM and PTFE membrane) | 60 | NR | NR | NR | NR | 7.32 | 0.27 ± 1.39 |
| Zitzmann, Scharer, & Marinello, 2001 | Implant placement (without bone augmentation) | 60 | NR | NR | NR | NR | 3.15 | 0.15 ± 0.94 |
| Zuiderveld et al., 2021 | Immediate implant placement (with CTG) + immediate provisionalization | 12 | NR | -0.07 ± 0.85 | NR | NR | NR | NR |
| Zuiderveld et al., 2021 | Immediate implant placement + immediate provisionalization | 12 | NR | 0.52 ± 1.16 | NR | NR | NR | NR |
| Zuiderveld, Meijer, den Hartog, Vissink, & Raghoebar, 2018 | Immediate implant placement + immediate provisionalization | 12 | NR | 0.5 ± 1.1 | NR | NR | NR | NR |
| Zuiderveld, Meijer, den Hartog, Vissink, & Raghoebar, 2018 | Immediate implant placement (with CTG) + immediate provisionalization | 12 | NR | -0.1 ± 0.8 | NR | NR | NR | NR |
| Zuiderveld, Meijer, Vissink, & Raghoebar, 2018 | ARP + Implant placement + CTG | 12 | NR | 0.04 ± 1.1 | NR | NR | NR | NR |
| Zuiderveld, Meijer, Vissink, & Raghoebar, 2018 | ARP + Implant placement | 12 | NR | 0.48 ± 1.5 | NR | NR | NR | NR |
| Zuiderveld, Meijer, Vissink, & Raghoebar, 2018 | ARP + Implant placement + CMX | 12 | NR | 0.17 ± 1.3 | NR | NR | NR | NR |

**Legend**. ADM: acellular dermal matrix; ARP: alveolar ridge preservation; CMX: collagen matrix; CTG: connective tissue graft; DBBM: deproteinized bovine bone mineral; DBBM-C: deproteinized bovine bone mineral particles embedded in collagen; FDBA: freeze-dried bone allograft; GBR: guided bone regeneration; KM: keratinized mucosa; ML: mucosal level; MREC: mucosal recession; NR: not reported; PRF: platelet-rich fibrin; PSTD: peri-implant soft tissue dehiscence; PTFE: polytetrafluoroethylene; SD: standard deviation; STH: supracrestal tissue height.

**Supplementary Table 9**. Clinical, radiographic, esthetic, and patient-reported outcomes of the included prospective studies.

| **Article** | **Intervention** | **Follow-up (months)** | **KM, MT, PD (mean SD)** | **BBT (at BL)** | **mean Rx MBL** | **Parameters associated with ML changes/ PSTD/ MREC (implant level)** | **Patient EST**  **(0-10 VAS)** | **Final PES (0-14)** | **Final mPES (0-10)** |
| --- | --- | --- | --- | --- | --- | --- | --- | --- | --- |
| Andersson, Odman, Lindvall, & Branemark, 1998 | Implant placement | 60 | NR, NR, NR | NR | NR | NR | NR | NR | NR |
| Arora & Ivanovski, 2017 | Immediate implant placement + immediate provisionalization | 24 | NR, NR, NR | 0.74 ± 0.26 | NR | No correlations pre-operative buccal bone thickness and soft tissue parameters | NR | 10.78 ± 1.93 | NR |
| Arora & Ivanovski, 2017 | Immediate implant placement + immediate provisionalization | 12 | NR, NR, NR | 0.74 ± 0.26 | NR |  | NR | 10.61 ± 1.5 | NR |
| Arora & Ivanovski, 2018 | Immediate implant placement | 12 | NR, NR, 2.46 ± 0.54 | NR | 0.07 | NR | NR | 9.40 ± 1.76 | NR |
| Arora & Ivanovski, 2018 | Early implant placement | 12 | NR, NR, 2.67 ± 0.44 | NR | 0.16 | NR | NR | 9.27 ± 1.62 | NR |
| Arora, Khzam, Roberts, Bruce, & Ivanovski, 2017 | Immediate implant placement + immediate provisionalization | 60 | NR, NR, NR | NR | NR | NR | NR | 11.25 ± 1.36 | NR |
| Arora, Khzam, Roberts, Bruce, & Ivanovski, 2017 | Immediate implant placement + immediate provisionalization | 12 | NR, NR, NR | NR | NR | NR | NR | 11.17 ± 1.64 | NR |
| Atef, El Barbary, Dahrous, & Zahran, 2021 | Immediate implant placement + socket shield | 6 | NR, NR, NR | 1.19 ± 0.82 | NR | NR | NR | NR | NR |
| Atef, El Barbary, Dahrous, & Zahran, 2021 | Immediate implant placement | 6 | NR, NR, NR | 1.48 ± 1.12 | NR | NR | NR | NR | NR |
| Atef, El Barbary, Dahrous, & Zahran, 2021 | Immediate implant placement + socket shield | 12 | NR, NR, NR | 1.19 ± 0.82 | NR | NR | 9.62 ± 0.51 | 12.12 ± 0.64 | NR |
| Atef, El Barbary, Dahrous, & Zahran, 2021 | Immediate implant placement | 12 | NR, NR, NR | 1.48 ± 1.12 | NR | NR | 9.25 ± 0.7 | 11.86 ± 0.35 | NR |
| Barone et al., 2016 | Implant placement (regular insertion torque) | 12 | NR, NR, NR | NR | NR | SSD higher recession in the group with high insertion torque (p<0.001) | NR | NR | NR |
| Barone et al., 2016 | Implant placement (high insertion torque) | 12 | NR, NR, NR | NR | NR |  | NR | NR | NR |
| Barone, Toti, Quaranta, Derchi, & Covani, 2015 | Immediate implant placement + immediate provisionalization | 24 | 3 ±1, NR, NR | 0.7 ± 0.2 | -1 | No correlations between pre-operative buccal bone thickness and soft tissue parameters | NR | NR | NR |
| Barone, Toti, Quaranta, Derchi, & Covani, 2015 | Immediate implant placement (delayed restoration) | 24 | 3 ±0.5, NR, NR | 0.6 ± 0.2 | -1 |  | NR | NR | NR |
| Bengazi, Wennströnm & Lekholm, 1996 | Implant placement (in presence of alveolar mucosa) | 24 | NR, NR, NR | NR | NR | NR | NR | NR | NR |
| Bengazi, Wennströnm & Lekholm, 1996 | Implant placement (in presence of KM) | 24 | NR, NR, NR | NR | NR | NR | NR | NR | NR |
| Benic et al., 2012 | Immediate implant placement + GBR | 84 | 2.4 ± 0.8, 1.5 ± 0.5, 2.7 ± 0.5 | NR | NR | NR | NR | NR | NR |
| Benic et al., 2017 | Implant placement + GBR | 12 | NR, NR, NR | NR | NR | NR | NR | NR | NR |
| Benic et al., 2017 | Implant placement (without bone augmentation) | 12 | NR, NR, NR | NR | NR | NR | NR | NR | NR |
| Benic et al., 2017 | Implant placement + GBR | 36 | NR, NR, NR | NR | NR | NR | NR | NR | NR |
| Benic et al., 2017 | Implant placement (without bone augmentation) | 36 | NR, NR, NR | NR | NR | NR | NR | NR | NR |
| Benitez Silva et al., 2022 | ARP with DBBM, and implant placement after 4 months | 12 | NR, NR, 2.73 ± NR | NR | 0.30 ± 0.9 | NR | 9.9 ± 1.3 | NR | NR |
| Benitez Silva et al., 2022 | ARP with DBBM-C, and implant placement after 4 months | 12 | NR, NR, 3.01 ± NR | NR | 0.25 ± 0.83 | NR | 9.5 ± 8.9 | NR | NR |
| Bianchi & Sanfilippo, 2004 | Immediate implant placement + CTG | 24 | NR, NR, NR | NR | NR | NR | NR | NR | NR |
| Bianchi & Sanfilippo, 2004 | Immediate implant placement | 24 | NR, NR, NR | NR | NR | NR | NR | NR | NR |
| Bianchi & Sanfilippo, 2004 | Immediate implant placement + CTG | 54 | NR, NR, NR | NR | NR | NR | NR | NR | NR |
| Bianchi & Sanfilippo, 2004 | Immediate implant placement | 54 | NR, NR, NR | NR | NR | NR | NR | NR | NR |
| Bianchi & Sanfilippo, 2004 | Immediate implant placement + CTG | 90 | NR, NR, NR | NR | NR | NR | NR | NR | NR |
| Bianchi & Sanfilippo, 2004 | Immediate implant placement | 90 | NR, NR, NR | NR | NR | NR | NR | NR | NR |
| Bittner et al., 2019 and Bittner et al., 2020b | Immediate implant placement in patients with thin phenotype | 6 | NR, NR, NR | NR | NR | NR | NR | NR | NR |
| Bittner et al., 2019 and Bittner et al., 2020b | Immediate implant placement (implant with anodised pink neck) in patients with thin phenotype | 6 | NR, NR, NR | NR | NR | NR | NR | NR | NR |
| Bittner et al., 2019 and Bittner et al., 2020b | Immediate implant placement in patients with thick phenotype | 6 | NR, NR, NR | NR | NR | NR | NR | NR | NR |
| Bittner et al., 2019 and Bittner et al., 2020b | Immediate implant placement (implant with anodised pink neck) in patients with thick phenotype | 6 | NR, NR, NR | NR | NR | NR | NR | NR | NR |
| Bittner et al., 2020a | Immediate implant placement + immediate provisionalization with bone graft | 6 | NR, NR, NR | 1.1 ± 0.7 | NR | NR | NR | NR | NR |
| Bittner et al., 2020a | Immediate implant placement + immediate provisionalization with bone graft | 12 | NR, NR, NR | 1.1 ± 0.7 | NR | NR | NR | NR | NR |
| Bittner et al., 2020a | Immediate implant placement + immediate provisionalization | 6 | NR, NR, NR | 0.8 ± 0.5 | NR | NR | NR | NR | NR |
| Bittner et al., 2020a | Immediate implant placement + immediate provisionalization | 12 | NR, NR, NR | 0.8 ± 0.5 | NR | NR | NR | NR | NR |
| Blanes, Bernard, Blanes, & Belser, 2007 | Implant placement | 72 | NR, NR, 2.54 ± 0.46 | NR | 4.24 ± 1.25 | NR | NR | NR | NR |
| Block et al., 2009 | ARP, delayed implant placement, and immediate provisionalization | 6 | NR, NR, NR | NR | NR | NR | NR | NR | NR |
| Block et al., 2009 | ARP, delayed implant placement, and immediate provisionalization | 12 | NR, NR, NR | NR | NR | NR | NR | NR | NR |
| Block et al., 2009 | ARP, delayed implant placement, and immediate provisionalization | 18 | NR, NR, NR | NR | NR | NR | NR | NR | NR |
| Block et al., 2009 | ARP, delayed implant placement, and immediate provisionalization | 24 | NR, NR, NR | NR | NR | NR | NR | NR | NR |
| Block et al., 2009 | Immediate implant placement + immediate provisionalization | 6 | NR, NR, NR | NR | NR | NR | NR | NR | NR |
| Block et al., 2009 | Immediate implant placement + immediate provisionalization | 12 | NR, NR, NR | NR | NR | NR | NR | NR | NR |
| Block et al., 2009 | Immediate implant placement + immediate provisionalization | 18 | NR, NR, NR | NR | NR | NR | NR | NR | NR |
| Block et al., 2009 | Immediate implant placement + immediate provisionalization | 24 | NR, NR, NR | NR | NR | NR | NR | NR | NR |
| Bonino et al., 2018 | Implant placement | 6 | NR, NR, NR | NR | NR | Association between MREC and lower patients' reported esthetic score (p<0.05) and between MREC at implants without KM (p<0.05) | NR | NR | NR |
| Bragger, Hammerle, & Lang, 1996 | Immediate implant placement (with barrier membrane) | 12 | NR, NR, 2.54 | NR | NR | NR | NR | NR | NR |
| Bragger, Hammerle, & Lang, 1996 | Immediate implant placement (without barrier membrane) | 12 | NR, NR, 2.47 | NR | NR | NR | NR | NR | NR |
| Bragger, Hammerle, & Lang, 1996 | Implant placement | 12 | NR, NR, 2.7 | NR | NR | NR | NR | NR | NR |
| Bressan et al., 2017 | Implant placement + definitive abutment | 36 | NR, NR, NR | NR | 0.11 ± 0.2 | NR | NR | 11.74 ± 1.85 | NR |
| Bressan et al., 2017 | Implant placement + definitive abutment | 12 | NR, NR, NR | NR | 0.09 ± 0.20 | NR | NR | 11.4 ± 1.5 | NR |
| Bressan et al., 2017 | Implant placement + repeated abutment changes | 36 | NR, NR, NR | NR | 0.61 ± 1 | NR | NR | 11.33 ± 1.55 | NR |
| Bressan et al., 2017 | Implant placement + repeated abutment changes | 12 | NR, NR, NR | NR | 0.33 ± 0.53 | NR | NR | 11 ± 2 | NR |
| Brunello et al., 2022 | Implant placement (zirconia implant) | 24 | NR, NR, 3.2 ± 0.5 | NR | NR | NR | NR | NR | NR |
| Brunello et al., 2022 | Implant placement (zirconia implant) | 108 | NR, NR, 3 ± 0.6 | NR | NR | NR | NR | NR | NR |
| Buser et al., 2009 | Early implant placement + GBR | 12 | 4.50 ± 1.54, NR, 4.43 ± 0.57 | NR | 0.18 ± 0.2 | NR | NR | NR | 8.1 |
| Buser et al., 2011 | Early implant placement + GBR | 36 | 4.10 ± 1.17, NR, 4 ± 0.56 | NR | 0.18 ± 0.23 | NR | NR | NR | 8.1 |
| Buser, Chappuis, Bornstein, et al., 2013 | Early implant placement + GBR | 84 | NR, NR, 4.26 ± 1.13 | NR | 2.18 ± 0.72 | NR | NR | NR | 7.78 |
| Buser, Chappuis, Kuchler, et al., 2013 | Early implant placement + GBR | 72 | 4, NR, 4.24 | NR | 0.44 ± 0.24 | NR | NR | NR | 7.49 |
| Bushahri et al., 2021 | Immediate implant placement + immediate provisionalization | 30 | 4.8 ± 1.2, 1.36 ± 0.7, 2.9 ± 0.4 | NR | 1.26 ± 0.61 | Abutment crown-angle SSD related to the mucosal margin change (p=0.02) | NR | NR | NR |
| Bushahri et al., 2021 | Immediate implant placement + delayed restoration | 30 | 4.9 ± 1, 1.39 ± 0.38, 3.3 ± 0.7 | NR | 1.09 ± 0.5 |  | NR | NR | NR |
| Cabello, Rioboo, & Fabrega, 2013 | Immediate implant + immediate provisionalization | 12 | 7.14 ± 1.51, 1.65 ± 0.52, 2.64 ± 0.93 | NR | NR | NR | NR | NR | NR |
| Canullo, Caneva, & Tallarico, 2017 | Immediate implant placement (platform switching) | 120 | NR, NR, NR | NR | NR | NR | NR | NR | NR |
| Canullo, Caneva, & Tallarico, 2017 | Immediate implant placement (non platform switching) | 120 | NR, NR, NR | NR | NR | NR | NR | NR | NR |
| Canullo, Iurlaro, & Iannello, 2009 | Immediate implant placement (platform switching) | 25 | NR, NR, NR | NR | NR | NR | NR | NR | NR |
| Canullo, Iurlaro, & Iannello, 2009 | Immediate implant placement (non platform switching) | 25 | NR, NR, NR | NR | NR | NR | NR | NR | NR |
| Cardaropoli, Gaveglio, Gherlone, & Cardaropoli, 2014 | Immediate implant placement + DBBM-C + CM | 12 | NR, NR, NR | NR | NR | NR | NR | NR | NR |
| Cardaropoli, Gaveglio, Gherlone, & Cardaropoli, 2014 | Immediate implant placement | 12 | NR, NR, NR | NR | NR | NR | NR | NR | NR |
| Cecchinato, Lops, Salvi, & Sanz, 2015 | Immediate implant placement (cylindrical implant) | 12 | NR, NR, NR | NR | NR | NR | NR | NR | NR |
| Cecchinato, Lops, Salvi, & Sanz, 2015 | Immediate implant placement (cylindrical implant) | 24 | NR, NR, NR | NR | NR | NR | NR | NR | NR |
| Cecchinato, Lops, Salvi, & Sanz, 2015 | Immediate implant placement (cylindrical implant) | 36 | NR, NR, NR | NR | NR | NR | NR | NR | NR |
| Cecchinato, Lops, Salvi, & Sanz, 2015 | Immediate implant placement (conical/cylindrical implant) | 12 | NR, NR, NR | NR | NR | NR | NR | NR | NR |
| Cecchinato, Lops, Salvi, & Sanz, 2015 | Immediate implant placement (conical/cylindrical implant) | 24 | NR, NR, NR | NR | NR | NR | NR | NR | NR |
| Cecchinato, Lops, Salvi, & Sanz, 2015 | Immediate implant placement (conical/cylindrical implant) | 36 | NR, NR, NR | NR | NR | NR | NR | NR | NR |
| Chan et al., 2019 | Immediate implant placement + immediate provisionalization | 12 | 5.3 ± 1.7, 0.6 ± 0.2, NR | NR | NR | NR | NR | NR | NR |
| Chan et al., 2019 | Immediate implant placement + delayed restoration | 12 | 6.1 ± 1.8, 0.7 ± 0.4, NR | NR | NR | NR | NR | NR | NR |
| Chen, Darby, & Reynolds, 2007 | Immediate implant placement (+ DBBM) | 12 | NR, NR, NR | 2.4 ± 0.3 | NR | Recession SSD associated with buccally positioned implants (p=0.032) | NR | NR | NR |
| Chen, Darby, & Reynolds, 2007 | Immediate implant placement (+ DBBM and CM) | 12 | NR, NR, NR | 2.2 ± 0.2 | NR |  | NR | NR | NR |
| Chen, Darby, & Reynolds, 2007 | Immediate implant placement (without graft) | 12 | NR, NR, NR | 2.3 ± 0.7 | NR |  | NR | NR | NR |
| Chung, Rungcharassaeng, Kan, Roe, & Lozada, 2011 | Immediate implant (+ CTG) + immediate provisionalization | 12 | NR, NR, NR | NR | NR | NR | NR | NR | NR |
| Cooper et al., 2010 | Immediate implant placement + immediate provisionalization | 12 | NR, NR, NR | NR | NR | NR | NR | NR | NR |
| Cooper et al., 2010 | Delayed implant placement | 12 | NR, NR, NR | NR | NR | NR | NR | NR | NR |
| Cooper et al., 2014 | Immediate implant placement + immediate provisionalization | 60 | NR, NR, NR | NR | NR | NR | NR | NR | NR |
| Cooper et al., 2014 | Delayed implant placement | 60 | NR, NR, NR | NR | NR | NR | NR | NR | NR |
| Cooper et al., 2015 | implant placement with conus interface between abutment and implant | 9 | NR, NR, NR | NR | 0.22 | NR | NR | NR | NR |
| Cooper et al., 2015 | implant placement with flat-to-flat interface between abutment and implant | 9 | NR, NR, NR | NR | 1.2 | NR | NR | NR | NR |
| Cooper et al., 2015 | implant placement with platform switched interface between abutment and implant | 9 | NR, NR, NR | NR | 1.32 | NR | NR | NR | NR |
| Cooper et al., 2019 | implant placement with conus interface between abutment and implant | 36 | NR, NR, 2.2 | NR | 0.12 | NR | NR | NR | NR |
| Cooper et al., 2019 | implant placement with flat-to-flat interface between abutment and implant | 36 | NR, NR, 2.7 | NR | 1.02 | NR | NR | NR | NR |
| Cooper et al., 2019 | implant placement with platform switched interface between abutment and implant | 36 | NR, NR, 2.2 | NR | 1.04 | NR | NR | NR | NR |
| Cordaro, Torsello, & Roccuzzo, 2009 | Immediate implant placement (submerged) + delayed provisionalization | 6 | 3.27 ± 1.03, NR, NR | NR | NR | NR | NR | NR | NR |
| Cordaro, Torsello, & Roccuzzo, 2009 | Immediate implant placement (submerged) + delayed provisionalization | 12 | 3.47 ± 0.99, NR, NR | NR | NR | NR | NR | NR | NR |
| Cordaro, Torsello, & Roccuzzo, 2009 | Immediate implant placement (non submerged) + delayed provisionalization | 6 | 2.07 ± 0.73, NR, NR | NR | NR | NR | NR | NR | NR |
| Cordaro, Torsello, & Roccuzzo, 2009 | Immediate implant placement (non submerged) + delayed provisionalization | 12 | 2.57 ± 0.93, NR, NR | NR | NR | NR | NR | NR | NR |
| Cosyn et al., 2011 | Immediate implant placement | 12 | NR, NR, 3.46 ± 0.69 | NR | 0.88 | NR | NR | NR | NR |
| Cosyn et al., 2011 | Immediate implant placement | 36 | NR, NR, 3.17 ± 0.63 | NR | 0.99 | NR | NR | 10.48 ± 2.47 | NR |
| Cosyn et al., 2016 | Immediate implant placement + immediate provisionalization | 12 | NR, NR, NR | NR | NR | NR | NR | NR | NR |
| Cosyn et al., 2016 | Immediate implant placement + immediate provisionalization | 60 | NR, NR, NR | NR | NR | NR | NR | NR | NR |
| Cosyn et al., 2016 | Immediate implant placement + immediate provisionalization + CTG after 3 months | 12 | NR, NR, NR | NR | NR | NR | NR | NR | NR |
| Cosyn et al., 2016 | Immediate implant placement + immediate provisionalization + CTG after 3 months | 60 | NR, NR, NR | NR | NR | NR | NR | NR | NR |
| Cosyn et al., 2022 | Implant placement + CTG | 12 | NR, NR, 3.12 ± NR | NR | 0.66 ± NR | NR | 8.96 | 11.99 | NR |
| Cosyn et al., 2022 | Implant placement + CMX | 12 | NR, NR, 3.14 ± NR | NR | 1.05 ± NR | NR | 8.94 | 11.23 | NR |
| Cosyn, De Bruyn, & Cleymaet, 2013 | Immediate implant placement + immediate provisionalization | 12 | NR, NR, NR | NR | 0.1 ± 0.5 | NR | NR | 12.15 ± 0.99 | NR |
| Cosyn, De Bruyn, & Cleymaet, 2013 | Immediate implant placement + immediate provisionalization | 6 | NR, NR, NR | NR | 0.1 ± 0.5 | NR | NR | 11.67 ± 1.07 | NR |
| Cosyn, Pollaris, Van der Linden, & De Bruyn, 2015 | ARP + delayed implant placement + CTG (at sites without midfacial recession) | 12 | NR, NR, NR | NR | NR | NR | NR | NR | NR |
| Cosyn, Pollaris, Van der Linden, & De Bruyn, 2015 | ARP + CTG + delayed implant placement (at sites with midfacial recession) | 12 | NR, NR, NR | NR | NR | NR | NR | NR | NR |
| Covani, Canullo, Toti, Alfonsi, & Barone, 2014 | Immediate implant placement | 60 | 0.67 ± 0.74, NR, NR | 0.76 ± 0.24 | 0.90 ± 0.2 | NR | 7.4 | NR | NR |
| Crespi et al., 2019 | Immediate implant placement (<2 mm KT) + immediate provisionalization | 96 | NR, NR, 2.80 ± 0.48 | NR | NR | Sites with ≥ 2 mm KT width showed higher soft tissue stability than sites with <2 mm KT width (p<0.01) | NR | NR | NR |
| Crespi et al., 2019 | Immediate implant placement (<2 mm KT) + immediate provisionalization | 96 | NR, NR, 2.75 ± 0.38 | NR | NR |  | NR | NR | NR |
| Crespi, Cappare, & Gherlone, 2010a | Immediate implant placement + immediate provisionalization (in presence of KM > 2 mm) | 24 | NR, NR, NR | NR | NR | NR | NR | NR | NR |
| Crespi, Cappare, & Gherlone, 2010a | Immediate implant placement + immediate provisionalization (in presence of KM > 2 mm) | 48 | NR, NR, 2.73 ± 0.34 | NR | NR | NR | NR | NR | NR |
| Crespi, Cappare, & Gherlone, 2010a | Immediate implant placement + immediate provisionalization (in presence of KM < 2 mm) | 24 | NR, NR, NR | NR | NR | NR | NR | NR | NR |
| Crespi, Cappare, & Gherlone, 2010a | Immediate implant placement + immediate provisionalization (in presence of KM < 2 mm) | 48 | NR, NR, 2.81 ± 0.41 | NR | NR | NR | NR | NR | NR |
| Crespi, Cappare, & Gherlone, 2010b | Immediate implant placement (in sites without peri-apical lesion) | 12 | 3.68 ± 0.72, NR, 1.85 ± 0.68 | NR | 0.80 ± 0.47 | NR | NR | NR | NR |
| Crespi, Cappare, & Gherlone, 2010b | Immediate implant placement (in sites without peri-apical lesion) | 24 | 3.67 ± 0.61, NR, 2.05 ± 0.66 | NR | 0.82 ± 0.52 | NR | NR | NR | NR |
| Crespi, Cappare, & Gherlone, 2010b | Immediate implant placement (in sites with peri-apical lesion) | 12 | 3.64 ± 0.68, NR, 1.80 ± 0.64 | NR | 0.83 ± 0.51 | NR | NR | NR | NR |
| Crespi, Cappare, & Gherlone, 2010b | Immediate implant placement (in sites with peri-apical lesion) | 24 | 3.62 ± 0.65, NR, 1.99 ± 0.57 | NR | 0.86 ± 0.54 | NR | NR | NR | NR |
| Crespi, Cappare, Gherlone, & Romanos, 2012 | Immediate implant placement + immediate provisionalization | 12 | 3.66 ± 0.7, NR, 1.82 ± 0.66 | NR | 0.81 ± 0.49 | NR | NR | NR | NR |
| Crespi, Cappare, Gherlone, & Romanos, 2012 | Immediate implant placement + immediate provisionalization | 24 | 3.64 ± 0.62, NR, 2.01 ± 0.61 | NR | 0.83 ± 0.52 | NR | NR | NR | NR |
| Crespi, Cappare, Polizzi, & Gherlone, 2015 | Immediate implant placement (implant with short collar) + immediate provisionalization | 24 | NR, NR, NR | NR | 1.08 ± 0.41 | NR | NR | NR | NR |
| Crespi, Cappare, Polizzi, & Gherlone, 2015 | Immediate implant placement (implant with short collar) + immediate provisionalization | 36 | NR, NR, NR | NR | 1.09 ± 0.38 | NR | NR | NR | NR |
| Crespi, Cappare, Polizzi, & Gherlone, 2015 | Immediate implant placement (implant with long collar) + immediate provisionalization | 24 | NR, NR, NR | NR | 0.5 ± 0.14 | NR | NR | NR | NR |
| Crespi, Cappare, Polizzi, & Gherlone, 2015 | Immediate implant placement (implant with long collar) + immediate provisionalization | 36 | NR, NR, NR | NR | 0.53 ± 0.12 | NR | NR | NR | NR |
| D'Elia et al., 2017 | Implant placement + GBR | 12 | 5.16 ± 1.22, 3.7 ± 1.1, 1.9 ± 0.42 | NR | NR | NR | NR | NR | NR |
| D'Elia et al., 2017 | Implant placement + CTG | 12 | 4.86 ± 0.83, 3.73 ± 1.13, 2.17 ± 0.67 | NR | NR | NR | NR | NR | NR |
| da Rosa, Rosa, Francischone, & Sotto-Maior, 2014 | Immediate implant placement (+ CTG) + immediate provisionalization | 58 | NR, NR, NR | NR | NR | NR | NR | NR | NR |
| de Albornoz et al., 2014 | Restoration with zirconia abutment | 12 | 5.4 ± 1.7, 2.3 ± 0.5, 2.9 ± 0.5 | NR | 0.06 ± NR | NR | 8.5 | NR | NR |
| de Albornoz et al., 2014 | Restoration with titanium abutment | 12 | 4.8 ± 1.3, 2.7 ± 1, 3.3 ± 0.8 | NR | 0.45 ± NR | NR | 8.5 | NR | NR |
| De Bruyckere et al., 2020 | Implant placement + CTG | 12 | NR, NR, 3.53 ± 0.40 | NR | NR | NR | NR | NR | NR |
| De Bruyckere et al., 2020 | Implant placement + GBR | 12 | NR, NR, 3.27 ± 0.75 | NR | NR | NR | NR | NR | NR |
| De Bruyn et al., 2013 | Immediate implant placement + immediate provisionalization | 60 | NR, NR, NR | NR | NR | NR | NR | NR | NR |
| De Bruyn et al., 2013 | Delayed implant placement | 60 | NR, NR, NR | NR | NR | NR | NR | NR | NR |
| De Rouck, Collys, & Cosyn, 2008 | Immediate implant placement + immediate provisionalization | 12 | NR, NR, 3.46 ± 0.7 | NR | 0.88 | NR | 9.3 | NR | NR |
| De Rouck, Collys, Wyn, & Cosyn, 2009 | Immediate implant placement + immediate provisionalization | 6 | NR, NR, 3.67 ± 0.75 | NR | NR | NR | NR | NR | NR |
| De Rouck, Collys, Wyn, & Cosyn, 2009 | Immediate implant placement + immediate provisionalization | 12 | NR, NR, 3.6 ± 0.61 | NR | NR | NR | NR | NR | NR |
| De Rouck, Collys, Wyn, & Cosyn, 2009 | Immediate implant placement with submerged healing and delayed restoration | 6 | NR, NR, 3.36 ± 0.62 | NR | NR | NR | NR | NR | NR |
| De Rouck, Collys, Wyn, & Cosyn, 2009 | Immediate implant placement with submerged healing and delayed restoration | 12 | NR, NR, 3.27 ± 0.53 | NR | NR | NR | NR | NR | NR |
| de Siqueira et al., 2017 | Implant placement (equicrestal) | 8 | NR, NR, NR | NR | 1.03 ± 0.60 | NR | NR | NR | NR |
| de Siqueira et al., 2017 | Implant placement (subcrestal) | 8 | NR, NR, NR | NR | 0.66 ± 0.38 | NR | NR | NR | NR |
| de Siqueira et al., 2020 | Implant placement (equicrestal) | 60 | NR, NR, NR | NR | 0.99 ± 0.55 | NR | NR | NR | NR |
| de Siqueira et al., 2020 | Implant placement (subcrestal) | 60 | NR, NR, NR | NR | 0.80 ± 0.52 | NR | NR | NR | NR |
| Degidi, Nardi, Daprile, & Piattelli, 2014 | Immediate implant placement + immediate provisionalization (and conventional final impression) | 6 | NR, NR, NR | NR | NR | NR | NR | NR | NR |
| Degidi, Nardi, Daprile, & Piattelli, 2014 | Immediate implant placement + immediate provisionalization (and conventional final impression) | 12 | NR, NR, NR | NR | NR | NR | NR | NR | NR |
| Degidi, Nardi, Daprile, & Piattelli, 2014 | Immediate implant placement + immediate provisionalization (and conventional final impression) | 24 | NR, NR, NR | NR | NR | NR | NR | NR | NR |
| Degidi, Nardi, Daprile, & Piattelli, 2014 | Immediate implant placement + immediate provisionalization (and final impression without abutment removal) | 6 | NR, NR, NR | NR | NR | NR | NR | NR | NR |
| Degidi, Nardi, Daprile, & Piattelli, 2014 | Immediate implant placement + immediate provisionalization (and final impression without abutment removal) | 12 | NR, NR, NR | NR | NR | NR | NR | NR | NR |
| Degidi, Nardi, Daprile, & Piattelli, 2014 | Immediate implant placement + immediate provisionalization (and final impression without abutment removal) | 24 | NR, NR, NR | NR | NR | NR | NR | NR | NR |
| den Hartog, Raghoebar, Stellingsma, Vissink, & Meijer, 2011 | Immediate implant placement + immediate provisionalization | 18 | NR, NR, 3.14 ± 0.92 | NR | 0.9 ± NR | NR | 9.15 ± 0.8 | NR | 7.1 ± 1.5 |
| den Hartog, Raghoebar, Stellingsma, Vissink, & Meijer, 2011 | Conventional Implant placement | 18 | NR, NR, 3.32 ± 0.79 | NR | 0.91 ± NR | NR | 8.95 ± 0.9 | NR | 6.5 ± 1.63 |
| Eghbali et al., 2018 | ARP + implant placement + CTG (3 months after implant placement) | 60 | NR, 2.42 ± 0.63, 3 ± 0.75 | NR | 0.47 ± 0.40 | NR | NR | 11.17 ± 1.91 | NR |
| Eghbali et al., 2018 | ARP + implant placement + CTG (3 months after implant placement) | 12 | NR, 2.5 ± 0.56, 3.15 ± 0.61 | NR | 0.53 ± 0.47 | NR | NR | 11 ± 1.64 | NR |
| Ekfeldt, Eriksson, & Johansson, 2003 | Implant placement (maxilla only) | 12 | NR, NR, NR | NR | NR | NR | NR | NR | NR |
| Ekfeldt, Eriksson, & Johansson, 2003 | Implant placement (mandible only) | 12 | NR, NR, NR | NR | NR | NR | NR | NR | NR |
| Esposito et al., 2017 | Implant placement + transmucosal abutment | 12 | 2.8 ± 1.7, NR, NR | NR | 0.23 ± 0.49 | NR | NR | 11.4 ± 1.5 | NR |
| Esposito et al., 2017 | Implant placement + definitive abutment + immediate provisionalization | 12 | 2.8 ± 1.5, NR, NR | NR | 0.06 ± 0.12 | NR | NR | 11 ± 2 | NR |
| Farrag & Khamis, 2023 | Implant placement + anodized titanium abutment collar | 12 | NR, NR, 2.62 ± 0.4 | NR | NR | NR | NR | NR | NR |
| Farrag & Khamis, 2023 | Implant placement + unanodized titanium abutment collar | 12 | NR, NR, 2.63 ± 0.39 | NR | NR | NR | NR | NR | NR |
| Farrag & Khamis, 2023 | Implant placement + anodized titanium abutment collar | 18 | NR, NR, 2.60 ± 0.4 | NR | NR | NR | NR | NR | NR |
| Farrag & Khamis, 2023 | Implant placement + unanodized titanium abutment collar | 18 | NR, NR, 2.65 ± 0.46 | NR | NR | NR | NR | NR | NR |
| Farronato et al., 2020 | Implant placement | 12 | NR, NR, NR | 0.5 | NR | SSD Correlation between pre-operative BBT and ML changes at posterior sites (p≤0.001), but not at anterior sites | NR | NR | NR |
| Farronato et al., 2020 | Implant placement | 24 | NR, NR, NR | 0.5 | NR |  | NR | NR | NR |
| Farronato et al., 2020 | Implant placement | 36 | NR, NR, NR | 0.5 | NR |  | NR | NR | NR |
| Farronato et al., 2020 | Implant placement | 12 | NR, NR, NR | 1.2 | NR |  | NR | NR | NR |
| Farronato et al., 2020 | Implant placement | 24 | NR, NR, NR | 1.2 | NR |  | NR | NR | NR |
| Farronato et al., 2020 | Implant placement | 36 | NR, NR, NR | 1.2 | NR |  | NR | NR | NR |
| Farronato et al., 2021 | Implant placement (platform-switching) | 12 | NR, NR, NR | NR | 0.24 ± 0.8 | NR | NR | NR | NR |
| Farronato et al., 2021 | Implant placement (non-platform-switching) | 12 | NR, NR, NR | NR | 0.72 ± 1.1 | NR | NR | NR | NR |
| Farronato et al., 2021 | Implant placement (platform-switching) | 24 | NR, NR, NR | NR | 0.23 ± 0.93 | NR | NR | NR | NR |
| Farronato et al., 2021 | Implant placement (non-platform-switching) | 24 | NR, NR, NR | NR | 0.78 ± 1.1 | NR | NR | NR | NR |
| Farronato et al., 2021 | Implant placement (platform-switching) | 36 | NR, NR, NR | NR | 0.37 ± 0.9 | NR | NR | NR | NR |
| Farronato et al., 2021 | Implant placement (non-platform-switching) | 36 | NR, NR, NR | NR | 0.8 ± 1.13 | NR | NR | NR | NR |
| Fenner, Hammerle, Sailer, & Jung, 2016 | Implant placement (and all-ceramic crowns on aluminium oxide-based abutments) | 86.4 | 3.72 ± 1.22, NR, 3.87 ± 0.76 | NR | 2.5 /- 0.9 | NR | 9.7 | NR | NR |
| Fenner, Hammerle, Sailer, & Jung, 2016 | Implant placement (and metal abutments on porcelain-fused-to-metal crowns) | 86.4 | 3.04 ± 1.15, NR, 4.16 ± 1.19 | NR | 2.2 ± 0.9 | NR | 9.7 | NR | NR |
| Fernandes, Marques, Borges, & Montero, 2023 | Immediate implant placement | 12 | NR, NR, NR | NR | NR | NR | NR | NR | NR |
| Fernandes, Marques, Borges, & Montero, 2023 | Immediate implant placement + CTG | 12 | NR, NR, NR | NR | NR | NR | NR | NR | NR |
| Ferrari, Cagidiaco, Garcia-Godoy, Goracci, & Cairo, 2015 | Implant placement and restoration with titanium abutment | 24 | NR, NR, NR | NR | NR | NR | NR | NR | NR |
| Ferrari, Cagidiaco, Garcia-Godoy, Goracci, & Cairo, 2015 | Implant placement and restoration with titanium nitride abutment | 24 | NR, NR, NR | NR | NR | NR | NR | NR | NR |
| Ferrari, Cagidiaco, Garcia-Godoy, Goracci, & Cairo, 2015 | Implant placement and restoration with zirconia abutment | 24 | NR, NR, NR | NR | NR | NR | NR | NR | NR |
| Finelle, Popelut, Knafo, & Sanz Martin, 2021 | Immediate implant placement + socket seal abutment | 24 | NR, NR, NR | NR | 0.89 ± 0.5 | NR | NR | NR | NR |
| Frizzera et al., 2019 | Immediate implant placement and immediate provisionalization | 12 | NR, 2.11 ± 0.60, NR | NR | NR | CTG groups showed significantly higher soft tissue stability compared to the control group (no soft tissue graft) that showed PSTD (p<0.05) | NR | 9.87 ± 1.64 | 6.62 ± 1.59 |
| Frizzera et al., 2019 | Immediate implant placement (+ CTG) and immediate provisionalization | 12 | NR, 3.04 ± 0.61, NR | NR | NR |  | NR | 10.75 ± 1.38 | 7.12 ± 0.99 |
| Frizzera et al., 2019 | Immediate implant placement (+ CMX) and immediate provisionalization | 12 | NR, 2.1 ± 0.54, NR | NR | NR |  | NR | 10 ± 1.3 | 7.87 ± 0.99 |
| Furhauser et al., 2017 | Immediate implant + immediate provisionalization | 24 | NR, NR, NR | NR | NR | NR | NR | 12.6 | NR |
| Furhauser et al., 2017 | Immediate implant + immediate provisionalization | 36 | NR, NR, NR | NR | NR | NR | NR | 12.6 | NR |
| Furhauser et al., 2017 | Immediate implant + immediate provisionalization | 48 | NR, NR, NR | NR | NR | NR | NR | 12.6 | NR |
| Furhauser et al., 2017 | Immediate implant + immediate provisionalization | 60 | NR, NR, NR | NR | NR | NR | NR | 12.6 | NR |
| Furhauser et al., 2017 | Immediate implant + immediate provisionalization | 12 | NR, NR, NR | NR | NR | NR | NR | 12.5 | NR |
| Gallucci, Grutter, Nedir, Bischof, & Belser, 2011 | Implant placement and all-ceramic restoration | 12 | 4.43 ± 1.71, NR, NR | NR | NR | Considering both groups, a slight recession (0.26 mm) was observed from 1y to 2y (p=0.005) | 8.1 ± 1.8 | NR | NR |
| Gallucci, Grutter, Nedir, Bischof, & Belser, 2011 | Implant placement and porcelain-fused-to-ceramic restoration | 12 | 4.57 ± 0.97, NR, NR | NR | NR |  | 8.6 ± 1.5 | NR | NR |
| Gallucci, Grutter, Nedir, Bischof, & Belser, 2011 | Implant placement and all-ceramic restoration | 24 | 4.67 ± 1.03, NR, NR | NR | NR |  | 9.18 ± 1 | NR | NR |
| Gallucci, Grutter, Nedir, Bischof, & Belser, 2011 | Implant placement and porcelain-fused-to-ceramic restoration | 24 | 4.83 ± 0.98, NR, NR | NR | NR |  | 9.18 ± 0.6 | NR | NR |
| Garaicoa-Pazmino et al., 2021 | Implant placement (STH ≤ 2 mm) | 6 | 3 ± 1.35, NR, 3.42 ± 0.37 | NR | NR | NR | NR | NR | NR |
| Garaicoa-Pazmino et al., 2021 | Implant placement (STH > 2 mm) | 6 | 3 ± 0.93, NR, 3.36 ± 0.42 | NR | NR | NR | NR | NR | NR |
| Garaicoa-Pazmino et al., 2021 | Implant placement (STH ≤ 2 mm) | 12 | 2.96 ± 1.13, NR, 3.13 ± 0.45 | NR | NR | NR | NR | NR | NR |
| Garaicoa-Pazmino et al., 2021 | Implant placement (STH > 2 mm) | 12 | 2.96 ± 1.12, NR, 3.46 ± 0.53 | NR | NR | NR | NR | NR | NR |
| Ghallab et al., 2023 | Immediate implant placement (vestibular extraction technique) | 12 | NR, NR, NR | NR | NR | NR | NR | 12.67 ± 1.59 | NR |
| Ghallab et al., 2023 | Immediate implant placement (conventional extraction technique) | 12 | NR, NR, NR | NR | NR | NR | NR | 11.4 ± 1.4 | NR |
| Girlanda et al., 2019 | Immediate implant placement (with DBBM-C) and immediate provisionalization | 6 | NR, NR, NR | NR | NR | NR | NR | NR | NR |
| Girlanda et al., 2019 | Immediate implant placement (without bone graft) and immediate provisionalization | 6 | NR, NR, NR | NR | NR | NR | NR | NR | NR |
| Gomez-Meda et al., 2022 | Immediate implant placement (+ CTG) | 12 | NR, NR, NR | NR | NR | NR | NR | NR | NR |
| Gomez-Meda et al., 2022 | Immediate implant placement (+ socket shield) | 12 | NR, NR, NR | NR | NR | NR | NR | NR | NR |
| Grandi, Guazzi, Samarani, & Grandi, 2013 | Immediate implant placement + immediate provisionalization | 12 | NR, NR, NR | NR | 0.35 ± 0.23 | NR | NR | NR | NR |
| Grandi, Guazzi, Samarani, & Grandi, 2013 | ARP + delayed implant placement (flapless) | 12 | NR, NR, NR | NR | 0.39 ± 0.16 | NR | NR | NR | NR |
| Grassi et al., 2015 | Implant placement (zirconia implant) + immediate provisionalization | 60 | NR, NR, NR | NR | 1.23 ± 0.29 | NR | NR | NR | NR |
| Groenendijk, Bronkhorst, & Meijer, 2021 | Immediate implant + immediate provisionalization | 12 | NR, NR, NR | NR | NR | NR | NR | NR | NR |
| Guarnieri et al., 2022 | Implant placement (submerged) | 60 | NR, NR, 2.24 ± 0.36 | NR | 0.87 ± 0.8 | NR | NR | NR | NR |
| Guarnieri et al., 2022 | Implant placement (nonsubmerged) | 60 | NR, NR, 2.11 ± 0.24 | NR | 0.56 ± 0.7 | NR | NR | NR | NR |
| Guarnieri, Ceccarelli, Ricci, & Testori, 2018 | Implant placement (implants with laser-microtextured collar surface) | 120 | 1.23 ± 0.21, NR, 2.3 ± 0.7 | NR | 1.23 ± 0.21 | NR | NR | NR | NR |
| Guarnieri, Ceccarelli, Ricci, & Testori, 2018 | Implant placement (implants with machined collar surface) | 120 | 2.8 ± 0.9, NR, 3.8 ± 0.8 | NR | 2.8 ± 0.9 | NR | NR | NR | NR |
| Guarnieri, Ceccherini, & Grande, 2015 | Immediate implant placement and early loading | 60 | NR, NR, 2.14 ± 0.38 | NR | 0.94 ± NR | NR | NR | 11.85 ± 3.4 | NR |
| Guarnieri, Di Nardo, Di Giorgio, Miccoli, & Testarelli, 2019 | Implant placement (submerged) | 36 | NR, NR, 0.7 ± 0.4 | NR | 0.27 ± 0.6 | NR | NR | NR | NR |
| Guarnieri, Di Nardo, Di Giorgio, Miccoli, & Testarelli, 2019 | Implant placement (nonsubmerged) | 36 | NR, NR, 0.8 ± 0.1 | NR | 0.26 ± 0.7 | NR | NR | NR | NR |
| Guarnieri, Di Nardo, Gaimari, Miccoli, & Testarelli, 2019 | Implant placement (short implant) | 36 | NR, NR, 1.12 ± 0.3 | NR | 0.24 | NR | NR | NR | NR |
| Guarnieri, Di Nardo, Gaimari, Miccoli, & Testarelli, 2019 | Implant placement (standard implant) | 36 | NR, NR, 1.01 ± 0.09 | NR | 0.26 | NR | NR | NR | NR |
| Hall et al., 2007 | Implant placement with delayed restoration | 12 | NR, NR, NR | NR | NR | NR | NR | NR | NR |
| Hall et al., 2007 | Implant placement with immediate provisionalization | 12 | NR, NR, NR | NR | NR | NR | NR | NR | NR |
| Hattingh, De Bruyn, Van Weehaeghe, Hommez, & Vandeweghe, 2020 | Immediate implant placement (ultra-wide implants in molar sites) | 12 | NR, NR, NR | NR | NR | NR | NR | NR | NR |
| Hof et al., 2015 | Implant placement + GBR | 46 | NR, NR, NR | NR | 1.7 ± 0.7 | PSTD depth significantly correlated by the time after implant placement (p=0.001), the presence of KM (p<0.001). PSTD associated with lower pink esthetic score (p<0.001) and papilla index (p<0.001) | 7.5 | 9.5 ± 2.2 | NR |
| Hof et al., 2015 | Delayed implant placement | 58 | NR, NR, NR | NR | 1.4 ± 0.8 |  | 8 | 11.2 ± 2 | NR |
| Hof et al., 2015 | Immediate implant placement | 56 | NR, NR, NR | NR | 1.5 ± 0.8 |  | 9.5 | 10.7 ± 2.4 | NR |
| Hof et al., 2015 | Early implant placement | 54 | NR, NR, NR | NR | 1.2 ± 0.6 |  | 8.4 | 10.4 ± 2.2 | NR |
| Hof et al., 2015 | Autogenous bone grafting + delayed implant placement | 42 | NR, NR, NR | NR | 1.8 ± 0.9 |  | 7.9 | 10.2 ± 2.1 | NR |
| Hollander et al., 2016 | Implant placement (zirconia implant) | 14 | NR, NR, 2.49 ± 0.7 | NR | NR | NR | NR | NR | NR |
| Hosseini et al., 2015 | Immediate implant placement + immediate provisionalization (with antibiotic therapy) | 6 | NR, NR, NR | NR | NR | SSD in favor of the group that received antibiotics in terms of the stability of the soft tissue margin (p=0.011) | NR | NR | NR |
| Hosseini et al., 2015 | Immediate implant placement + immediate provisionalization (without antibiotic therapy) | 6 | NR, NR, NR | NR | NR |  | NR | NR | NR |
| Humm et al., 2023 | Ceramic restoration with zirconia abutment | 156 | NR, 1.25 ± NR, 3.84 ± 1.7 | NR | 2.38 ± 1.97 | NR | NR | NR | NR |
| Humm et al., 2023 | Metal ceramic crown with titanium abutment | 156 | NR, 1.25 ± NR, 3.18 ± 0.65 | NR | 2 ± 1.03 | NR | NR | NR | NR |
| Huynh-Ba et al., 2019 | Immediate implant placement | 12 | NR, NR, NR | NR | NR | NR | 9.70 ± 0.3 | NR | NR |
| Huynh-Ba et al., 2019 | Early implant placement with GBR | 12 | NR, NR, NR | NR | NR | NR | 9.27 ± 0.38 | NR | NR |
| Iorio-Siciliano et al., 2016 | Implant placement | 24 | NR, NR, 2.55 ± 0.94 | NR | 0.7 | NR | NR | NR | NR |
| Jacobs, Zadeh, De Kok, & Cooper, 2020 | Immediate implant placement (+ DBBM) | 9.7 | NR, NR, NR | NR | NR | SSD correlation age with midfacial change. Older patients experienced greater soft tissue change (p=0.04) | NR | 8.2 ± 2.5 | NR |
| Jacobs, Zadeh, De Kok, & Cooper, 2020 | Immediate implant placement (without bone graft) | 9.7 | NR, NR, NR | NR | NR |  | NR | 8.2 ± 1.8 | NR |
| Jeffcoat, McGlumphy, Reddy, Geurs, & Proskin, 2003 | Implant placement | 60 | NR, NR, NR | NR | NR | NR | NR | NR | NR |
| Jeffcoat, McGlumphy, Reddy, Geurs, & Proskin, 2003 | Implant placement | 60 | NR, NR, NR | NR | NR | NR | NR | NR | NR |
| Jeffcoat, McGlumphy, Reddy, Geurs, & Proskin, 2003 | Implant placement | 60 | NR, NR, NR | NR | NR | NR | NR | NR | NR |
| Jemt, Ahlberg, Henriksson, & Bondevik, 2006 | Implant placement | 180 | NR, NR, NR | NR | NR | NR | NR | NR | NR |
| Jung et al., 2016 | Implant placement (zirconia implant) + immediate provisionalization | 12 | NR, NR, 3.5 ± 0.7 | NR | NR | NR | NR | NR | NR |
| Kan, Rungcharassaeng, & Lozada, 2003 | Immediate implant placement + immediate provisionalization | 12 | NR, NR, NR | NR | 0.26 ± 0.4 | NR | 9.9 | NR | NR |
| Kan, Rungcharassaeng, Lozada, & Zimmerman, 2011 | Immediate implant placement + immediate provisionalization | 48 | NR, NR, NR | NR | 0.68 | Sites with thick phenotype had significantly less ML changes than sites with thin phenotype (p<0.001) | NR | NR | NR |
| Kan, Rungcharassaeng, Sclar, & Lozada, 2007 | Immediate implant placement + immediate provisionalization | 12 | NR, NR, NR | NR | NR | Defect morphology had a correlation with the frequency of midfacial recession >1.5mm | NR | NR | NR |
| Karoussis et al., 2004 | Implant placement | 120 | 2.84, NR, 2.78 | NR | NR | NR | NR | NR | NR |
| Kobayashi et al., 2020 | Implant placement + GBR | 12 | NR, 1.99 ± 0.69, NR | 1.72 ± 1.07 | NR | SSD higher ML changes in the group that did not receive CTG (p<0.001) | NR | NR | NR |
| Kobayashi et al., 2020 | Implant placement + GBR + CTG (at second stage) | 12 | NR, 2.73 ± 0.84, NR | 2.26 ± 1.32 | NR |  | NR | NR | NR |
| Koh et al., 2011 | Immediate implant placement (at the level of the crest) | 6 | 4.4 ± 0.3, 2.4 ± 0.3, NR | 1.1 ± 0.2 | NR | NR | NR | NR | NR |
| Koh et al., 2011 | Immediate implant placement (subcrestal) | 6 | 5 ± 0.3, 3.1 ± 0.4, NR | 1.4 ± 0.2 | NR | NR | NR | NR | NR |
| Lago, da Silva, Gude, & Rilo, 2017 | Implant placement | 48 | NR, NR, NR | NR | 0.28 ± 0.45 | NR | NR | NR | NR |
| Lee et al., 2020 | Immediate implant placement (flapless) and immediate provisionalization | 12 | NR, NR, NR | NR | NR | Mucosal level changes SSD associated to soft tissue thickness (p=0.01) and buccal bone dehiscence (p=0.03) | NR | NR | NR |
| Lee et al., 2020 | Immediate implant placement (with a flap) and immediate provisionalization | 12 | NR, NR, NR | NR | NR |  | NR | NR | NR |
| Lee et al., 2023 | Immediate implant placement + CTG | 12 | 0.18 ± 1.07, 0.80 ± 0.26, NR | NR | 0.48 | NR | NR | NR | NR |
| Lee et al., 2023 | Immediate implant placement + ADM | 12 | 0.80 ± 1.11, 0.80 ± 0.36, NR | NR | 0.54 | NR | NR | NR | NR |
| Lee et al., 2023 | Immediate implant placement | 12 | 0.18 ± 0.91, 0.20 ± 0.17, NR | NR | 0.95 | NR | NR | NR | NR |
| Lilet et al., 2022 | Immediate implant placement + socket seal abutment | 12 | NR, NR, 3 ± 1.03 | NR | NR | NR | NR | 12.1 ± 1.55 | NR |
| Lindeboom, Tjiook, & Kroon, 2006 | Immediate implant placement | 12 | NR, NR, NR | NR | 0.51 | NR | NR | NR | NR |
| Lindeboom, Tjiook, & Kroon, 2006 | Delayed implant placement | 12 | NR, NR, NR | NR | 0.53 | NR | NR | NR | NR |
| Liu et al., 2019 | Immediate implant placement + GBR | 12 | NR, NR, 3.56 ± 0.68 | NR | NR | NR | 8.79 | 10.58 ± 2.47 | NR |
| Lops et al., 2015 | Implant placement and restoration with zirconia stock abutment | 24 | NR, 1.72, NR | NR | NR | NR | NR | NR | NR |
| Lops et al., 2015 | Implant placement and restoration with titanium stock abutment | 24 | NR, 2.91, NR | NR | NR | NR | NR | NR | NR |
| Lops et al., 2015 | Implant placement and restoration with zirconia cad-cam abutment | 24 | NR, 1.75, NR | NR | NR | NR | NR | NR | NR |
| Lops et al., 2015 | Implant placement and restoration with titanium cad-dam abutment | 24 | NR, 2.75, NR | NR | NR | NR | NR | NR | NR |
| Lops, Romeo, Chiapasco, Procopio, & Oteri, 2013 | Immediate implant placement | 12 | NR, NR, NR | NR | NR | NR | NR | NR | NR |
| Lorenz et al., 2019 | Implant placement (zirconia implant) | 93.6 | NR, NR, 2.57 ± 1.1 | NR | 1.2 ± 0.76 | NR | NR | 9 ± 2.7 | NR |
| Lorenz et al., 2022 | Implant placement (zirconia implant) | 15 | 3.79 ± 0.97, NR, 2.49 ± 0.49 | NR | NR | NR | NR | 11.67 ± 1.6 | NR |
| Lowy et al., 2019 | Implant placement (platform-matched implant) | 12 | 4 ± 1, 2.1 ± 0.5, NR | NR | 0.7 | NR | NR | NR | NR |
| Lowy et al., 2019 | Implant placement (platform-switched implant) | 12 | 3.8 ± 1.8, 2.2 ± 1, NR | NR | 0.1 | NR | NR | NR | NR |
| Malchiodi, Cucchi, Ghensi, & Nocini, 2013 | Immediate implant placement + immediate provisionalization | 36 | NR, NR, NR | NR | 0.8 | Statistically significant correlation between PSTD depth and interproximal crestal bone levels (p=0.02) | NR | NR | NR |
| Marconcini et al., 2018 | Implant placement (regular insertion torque) | 12 | NR, NR, NR | NR | 0.7 | Greater ML changes in the high insertion torque (p<0.001) and in the mandible (p=0.01) | NR | NR | NR |
| Marconcini et al., 2018 | Implant placement (high insertion torque) | 12 | NR, NR, NR | NR | 1.18 |  | NR | NR | NR |
| Marconcini et al., 2018 | Implant placement (regular insertion torque) | 36 | NR, NR, NR | NR | 0.99 |  | NR | NR | NR |
| Marconcini et al., 2018 | Implant placement (high insertion torque) | 36 | NR, NR, NR | NR | 1.35 |  | NR | NR | NR |
| Mau et al., 2019 | Early implant placement with FDBA | 12 | 4.42 ± 0.77, NR, 3.07 ± 0.49 | NR | 0.32 ± 0.56 | NR | NR | NR | 7.96 ± 0.81 |
| Mau et al., 2019 | Early implant placement with autogenous graft + DBBM | 12 | 5.07 ± 1.27, NR, 3.04 ± 0.63 | NR | 0.21 ± 0.41 | NR | NR | NR | 7.92 ± 1.44 |
| Meijndert et al., 2017 | Bone augmentation with autogenous bone graft and implant placement (after 3 months) | 120 | NR, NR, NR | NR | 0.22 | NR | 8.6 | NR | NR |
| Meijndert et al., 2017 | Bone augmentation with autogenous bone graft + collagen membrane and implant placement (after 3 months) | 120 | NR, NR, NR | NR | 0.49 | NR | 8.6 | NR | NR |
| Meijndert et al., 2017 | Bone augmentation with DBBM + collagen membrane and implant placement (after 6 months) | 120 | NR, NR, NR | NR | 0.51 | NR | 8.6 | NR | NR |
| Meijndert, Raghoebar, Meijer, & Vissink, 2008 | Bone augmentation with autogenous bone graft and implant placement (after 3 months) | 12 | NR, NR, NR | NR | 0.15 | NR | 8.5 | NR | NR |
| Meijndert, Raghoebar, Meijer, & Vissink, 2008 | Bone augmentation with autogenous bone graft + collagen membrane and implant placement (after 3 months) | 12 | NR, NR, NR | NR | 0.11 | NR | 8.4 | NR | NR |
| Meijndert, Raghoebar, Meijer, & Vissink, 2008 | Bone augmentation with DBBM + collagen membrane and implant placement (after 6 months) | 12 | NR, NR, NR | NR | 0.15 | NR | 8.7 | NR | NR |
| Meijndert, Raghoebar, Vissink, & Meijer, 2022 | Implant placement + GBR | 6 | NR, NR, NR | NR | 0.51 ± 1.06 | NR | 9.01 | NR | NR |
| Migliorati, Amorfini, Signori, Biavati, & Benedicenti, 2015 | Immediate implant placement + CTG | 12 | 3 ± 1.2, 1.8 ± 0.8, 3.3 ± 0.4 | NR | NR | Thin phenotype had significantly higher PSTD at 1 year (p<0.001) | NR | NR | NR |
| Migliorati, Amorfini, Signori, Biavati, & Benedicenti, 2015 | Immediate implant placement | 12 | 2.9 ± 1.2, 1.1 ± 0.5, 3.1 ± 1 | NR | NR |  | NR | NR | NR |
| Migliorati, Amorfini, Signori, Biavati, & Benedicenti, 2015 | Immediate implant placement + CTG | 24 | 3.7 ± 1.1, 1.5 ± 0.8, 3.4 ± 0.5 | NR | NR | Thin phenotype had significantly higher PSTD at 2 year (p=0.002) | NR | NR | 7.96 |
| Migliorati, Amorfini, Signori, Biavati, & Benedicenti, 2015 | Immediate implant placement | 24 | 3.6 ± 1.2, 1 ± 0.5, 3.2 ± 0.5 | NR | NR |  | NR | NR | 6.39 |
| Mizuno, Nakano, Shimomoto, Fujita, & Ishigaki, 2022 | Immediate implant + GBR | 12 | NR, 0.5 ± 0.3, NR | 2.1 ± 0.7 | NR | SSD correlation between the ML changes and pre-operative buccal bone dehiscence width (p=0.04), and between ML changes and pre-operative buccal bone dehiscence depth (p=0.03) | NR | NR | NR |
| Molina, Sanz-Sanchez, Martin, Blanco, & Sanz, 2017 | Implant placement (with healing cap) | 6 | NR, NR, 3.23 ± 0.74 | NR | 0.28 ± 0.43 | NR | NR | NR | NR |
| Molina, Sanz-Sanchez, Martin, Blanco, & Sanz, 2017 | Implant placement (with definitive abutment) | 6 | NR, NR, 3.03 ± 0.74 | NR | 0.01 ± 0.5 | NR | NR | NR | NR |
| Molina, Sanz-Sanchez, Martin, Blanco, & Sanz, 2017 | Implant placement (with healing cap) | 12 | NR, NR, 3.08 ± 0.77 | NR | 0.32 ± 0.58 | NR | NR | NR | NR |
| Molina, Sanz-Sanchez, Martin, Blanco, & Sanz, 2017 | Implant placement (with definitive abutment) | 12 | NR, NR, 3.19 ± 0.55 | NR | 0.01 ± 0.54 | NR | NR | NR | NR |
| Munoz-Camara, Gilbel-Del Aguila, Pardo-Zamora, & Camacho-Alonso, 2020 | Immediate implant placement + immediate provisionalization (at sites with peri-apical pathology) | 12 | 3.76 ± 0.62, NR, 3.16 ± 0.41 | NR | 0.35 ± 0.51 | NR | NR | NR | NR |
| Munoz-Camara, Gilbel-Del Aguila, Pardo-Zamora, & Camacho-Alonso, 2020 | Immediate implant placement + immediate provisionalization (at sites without peri-apical pathology) | 12 | 3.50 ± 1.44, NR, 3.31 ± 0.94 | NR | 0.15 ± 0.87 | NR | NR | NR | NR |
| Oates, West, Jones, Kaiser, & Cochran, 2002 | Implant placement | 24 | NR, NR, NR | NR | NR | NR | NR | NR | NR |
| Parvini, Muller, Cafferata, Schwarz, & Obreja, 2022 | Immediate implant placement + immediate provisionalization | 12 | 5.25 ± 1.18, NR, 3.25 ± 0.58 | NR | NR | NR | NR | NR | NR |
| Parvini, Muller, Cafferata, Schwarz, & Obreja, 2022 | Delayed implant placement + immediate provisionalization | 12 | 4.44 ± 1.03, NR, 3.28 ± 0.73 | NR | NR | NR | NR | NR | NR |
| Perez et al., 2020 | Immediate implant placement + customized healing abutment | 12 | 4.0 ± 1.1, NR, NR | NR | 1.2 ± 0.7 | NR | NR | NR | 8.7 ± 1 |
| Perez et al., 2020 | Immediate implant placement + standard healing abutment | 12 | 3.3 ± 0.8, NR, NR | NR | 0.8 ± 1.1 | NR | NR | NR | 8.2 ± 0.7 |
| Pieri, Aldini, Marchetti, & Corinaldesi, 2011 | Immediate implant placement + immediate provisionalization (abutment with morse connection and platform switch) | 12 | 3.86 ± 0.72, 2.72 ± 0.49, 2.58 ± 0.49 | NR | 0.19 ± 0.17 | NR | NR | NR | NR |
| Pieri, Aldini, Marchetti, & Corinaldesi, 2011 | Immediate implant placement + immediate provisionalization (abutment with internal connection and matching diameter) | 12 | 3.84 ± 0.57, 2.52 ± 0.48, 2.71 ± 0.48 | NR | 0.49 ± 0.25 | NR | NR | NR | NR |
| Pieri, Aldini, Marchetti, & Corinaldesi, 2013 | Staged bone augmentation and delayed implant placement | 60 | 4.08 ± 0.89, NR, 4.29 ± 0.67 | 2.44 ± 0.5 | 0.61 ± 0.33 | NR | NR | 8.61 ± 1.55 | NR |
| Pohl, Furhauser, Haas, & Pohl, 2020 | Immediate implant + immediate provisionalization (at sites with buccal bone dehiscence) | 12 | NR, NR, NR | NR | 1.58 ± 2.33 | NR | NR | 9.68 ± 2.52 | NR |
| Pohl, Furhauser, Haas, & Pohl, 2020 | Immediate implant + immediate provisionalization (at sites with intact buccal plate) | 12 | NR, NR, NR | NR | 1.42 ± 0.71 | NR | NR | 12.25 ± 1.29 | NR |
| Proussaefs, Kan, Lozada, Kleinman, & Farnos, 2002 | Implant placement + immediate provisionalization | 12 | NR, NR, 3.2 | NR | 0.9 | NR | NR | NR | NR |
| Puisys et al., 2022 | Immediate implant placement + CTG + immediate provisionalization | 12 | NR, NR, 1.6 ± 0.82 | NR | 0.15 | NR | NR | 12.6 ± 1.27 | NR |
| Puisys et al., 2022 | Extraction + CTG. Early implant placement + GBR + delayed loading | 12 | NR, NR, 1.8 ± 0.99 | NR | 0.25 | NR | NR | 12.1 ± 1.32 | NR |
| Qian et al., 2023 | Immediate implant placement + GBR + CTG | 12 | NR, NR, 3.54 ± 0.67 | 2.87 ± 0.3 | 0.21 ± 0.24 | NR | NR | NR | 9.17 ± 0.72 |
| Raes et al., 2015 | Implant placement + immediate provisionalization (in smokers) | 24 | NR, NR, NR | NR | 0.22 ± 0.42 | Lower ML changes in favor of the non-smoker group (p=0.004) | NR | NR | NR |
| Raes et al., 2015 | Implant placement + immediate provisionalization (in non-smokers) | 24 | NR, NR, NR | NR | -0.33 ± 1.19 |  | NR | NR | NR |
| Raes et al., 2018 | Immediate implant placement + immediate provisionalization | 96 | NR, 1.32 ± 0.55, NR | NR | NR | No significant association between buccal bone (thickness and dehiscence) and midfacial recession | NR | 10.36 | NR |
| Raes et al., 2018 | Conventional implant placement + immediate provisionalization | 96 | NR, 1.24 ± 0.77, NR | NR | NR |  | NR | 9.22 | NR |
| Raes, Cosyn, & De Bruyn, 2013 | Conventional implant placement + immediate provisionalization | 13 | NR, NR, NR | NR | 0.18 ± 1.26 | Conventional implant placement showed a significant recession after 13 months compared to the other groups | NR | 10.35 ± 1.58 | NR |
| Raes, Cosyn, & De Bruyn, 2013 | Immediate implant placement + immediate provisionalization | 13 | NR, NR, NR | NR | 1.05 ± 1.78 |  | NR | 10.33 ± 2.29 | NR |
| Raes, Cosyn, & De Bruyn, 2013 | Staged GBR and Implant placement (after 4-5 months) | 13 | NR, NR, NR | NR | 0.27 ± 0.7 |  | NR | 10.11 ± 11.9 | NR |
| Raes, Cosyn, Crommelinck, Coessens, & De Bruyn, 2011 | Conventional implant placement + immediate provisionalization | 13 | NR, NR, NR | NR | NR | Flapless approach for immediate implant placement was significantly correlated with lower ML changes, compared to flap approach (p=0.023) | NR | 10.35 ± 1.58 | NR |
| Raes, Cosyn, Crommelinck, Coessens, & De Bruyn, 2011 | Immediate implant placement + immediate provisionalization | 13 | NR, NR, NR | NR | NR |  | NR | 10.33 ± 2.29 | NR |
| Ribeiro dos Reis et al., 2023 | Implant placement (in presence of STH < 3 mm) + immediate provisionalization | 12 | NR, NR, NR | NR | 0.23 | NR | NR | NR | NR |
| Ribeiro dos Reis et al., 2023 | Implant placement (in presence of STH ≥ 3 mm) + immediate provisionalization | 12 | NR, NR, NR | NR | 0.04 | NR | NR | NR | NR |
| Rivara et al., 2020 | Implant placement (2 mm of distance between the two implants) | 6 | 2.96 ± NR, NR, NR | NR | 0.61 ± NR | NR | NR | NR | NR |
| Rivara et al., 2020 | Implant placement (3 mm of distance between the two implants) | 6 | 2.76 ± NR, NR, NR | NR | 0.48 ± NR | NR | NR | NR | NR |
| Rivara et al., 2020 | Implant placement (2 mm of distance between the two implants) | 12 | 2.78 ± NR, NR, NR | NR | 0.71 ± NR | NR | NR | NR | NR |
| Rivara et al., 2020 | Implant placement (3 mm of distance between the two implants) | 12 | 2.7 ± NR, NR, NR | NR | 0.46 ± NR | NR | NR | NR | NR |
| Romanos, Malmstrom, Feng, Ercoli, & Caton, 2014 | Implant placement (Ankylos plus, Dentsply) | 12 | 3.39 ± 0.68, NR, 1.99 ± 0.44 | NR | NR | NR | NR | NR | NR |
| Romanos, Malmstrom, Feng, Ercoli, & Caton, 2014 | Implant placement (Certain Prevail, Biomet 3i) | 12 | 3.41 ± 0.92, NR, 2.02 ± 0.5 | NR | NR | NR | NR | NR | NR |
| Romanos, Malmstrom, Feng, Ercoli, & Caton, 2014 | Implant placement (Certain Prevail, Biomet 3i) | 24 | 3.11 ± 0.8, NR, 1.76 ± 0.47 | NR | NR | NR | NR | NR | NR |
| Romanos, Malmstrom, Feng, Ercoli, & Caton, 2014 | Certain Prevail (Biomet 3i) | 24 | 3.17 ± 1.08, NR, 2.11 ± 0.73 | NR | NR | NR | NR | NR | NR |
| Salvi et al., 2020 | Implant placement (implant with modified SLA transmucosal neck surface) | 12 | NR, NR, 3.1 ± 0.6 | NR | 0.24 ± 0.59 | NR | NR | NR | NR |
| Salvi et al., 2020 | Implant placement (implant with machined transmucosal neck surface) | 12 | NR, NR, 2.9 ± 0.6 | NR | 0.14 ± 0.3 | NR | NR | NR | NR |
| Salvi et al., 2020 | Implant placement (implant with modified SLA transmucosal neck surface) | 36 | NR, NR, 3.2 ± 0.8 | NR | 0.33 ± 0.69 | NR | NR | NR | NR |
| Salvi et al., 2020 | Implant placement (implant with machined transmucosal neck surface) | 36 | NR, NR, 3 ± 0.6 | NR | 0.12 ± 0.3 | NR | NR | NR | NR |
| Santing, Raghoebar, Vissink, den Hartog, & Meijer, 2013 | Implant placement | 18 | NR, NR, 2.68 ± 0.62 | NR | 0.1 | NR | NR | NR | 6.9 ± 1.8 |
| Sanz Martin, Benic, Hammerle, & Thoma, 2016 | Implant placement (one-piece implant) | 12 | NR, NR, NR | NR | 0.35 ± 0.35 | NR | NR | NR | NR |
| Sanz Martin, Benic, Hammerle, & Thoma, 2016 | implant placement (two-piece implant) | 12 | NR, NR, NR | NR | 0.08 ± 0.2 | NR | NR | NR | NR |
| Sanz-Martin, Encalada, Sanz-Sanchez, Aracil, & Sanz, 2019 | Immediate implant placement (+ CMX) + immediate provisionalization | 6 | 2.25 ± 0.86, NR, 2.87 ± 0.71 | NR | 0.15 ± 0.57 | NR | NR | 11.33 ± 1.92 | NR |
| Sapata et al., 2018 | Implant placement (one-piece implant) | 60 | NR, NR, NR | NR | 0.5 | NR | NR | NR | NR |
| Sapata et al., 2018 | implant placement (two-piece implant) | 60 | NR, NR, NR | NR | 1.41 | NR | NR | NR | NR |
| Schropp & Isidor, 2008 | Early or delayed implant placement | 54 | NR, NR, NR | NR | NR | NR | NR | NR | NR |
| Schropp & Isidor, 2008 | Early implant placement | 24 | NR, NR, NR | NR | 1.3 ± 0.6 | NR | NR | NR | NR |
| Schropp & Isidor, 2008 | Delayed implant placement | 24 | NR, NR, NR | NR | 1.5 ± 0.5 | NR | NR | NR | NR |
| Schropp & Isidor, 2008 | Early implant placement | 60 | NR, NR, NR | NR | 1.2 ± 0.5 | NR | NR | NR | NR |
| Schropp & Isidor, 2008 | Delayed implant placement | 60 | NR, NR, NR | NR | 1.5 ± 0.7 | NR | NR | NR | NR |
| Schrott, Jimenez, Hwang, Fiorellini, & Weber, 2009 | Implant placement (KM ≥ 2 mm) | 60 | NR, NR, NR | NR | NR | SSD more recession at implant sites having < 2 mm of KM compared to sites with ≥ 2 mm of KM (p<0.001) | NR | NR | NR |
| Schrott, Jimenez, Hwang, Fiorellini, & Weber, 2009 | Implant placement (KM < 2 mm) | 60 | NR, NR, NR | NR | NR |  | NR | NR | NR |
| Schwarz, Sahm, & Becker, 2012 | Implant placement + GBR resulting in 0 mm of residual bone defect height after 4 months | 48 | NR, NR, 2.8 ± NR | NR | NR | NR | NR | NR | NR |
| Schwarz, Sahm, & Becker, 2012 | Implant placement + GBR resulting in 1 mm of residual bone defect height after 4 months | 48 | NR, NR, 2.0 ± NR | NR | NR | NR | NR | NR | NR |
| Schwarz, Sahm, & Becker, 2012 | Implant placement + GBR resulting in > 1 mm of residual bone defect height after 4 months | 48 | NR, NR, 2.5 ± NR | NR | NR | NR | NR | NR | NR |
| Schwarz, Schmucker, & Becker, 2017 | Implant placement + GBR (with native collagen membrane) | 96 | NR, NR, 3.0 ± 0.9 | NR | NR | NR | NR | NR | NR |
| Schwarz, Schmucker, & Becker, 2017 | Implant placement + GBR (with cross-linked collagen membrane) | 96 | NR, NR, 2.4 ± 0.4 | NR | NR | NR | NR | NR | NR |
| Seyssens, Eghbali, & Cosyn, 2020 | Immediate implant placement + immediate provisionalization | 120 | NR, NR, NR | NR | NR | Putative risk factors (descriptive analysis) for recession: buccal shoulder position, no CTG, convex emergence profile, and central incisor position | NR | NR | NR |
| Seyssens, Eghbali, & Cosyn, 2020 | Immediate implant placement + immediate provisionalization + CTG after 3 months | 120 | NR, NR, NR | NR | NR |  | NR | NR | NR |
| Siegenthaler et al., 2022 | Provisional crown with a concave contour | 12 | NR, 3.07 ± 1.24, NR | NR | 0.28 ± 0.27 | Convex emergence profile was found more prone to show recession than concave (OR 12.6, 95% CI 1.82-88.48, p=0.01) | NR | NR | 6.2 ± 1.6 |
| Siegenthaler et al., 2022 | Provisional crown with a convex contour | 6 | NR, 3.14 ± 1.34, NR | NR | 0.55 ± 0.40 |  | NR | NR | 6 ± 2.1 |
| Siegenthaler et al., 2022 | Healing abutment, no provisional crown | 6 | NR, 3.43 ± 1.48, NR | NR | 0.43 ± 0.20 |  | NR | NR | 5.9 ± 1.7 |
| Siegenthaler et al., 2022 | Provisional crown with a convex contour | 12 | NR, 2.86 ± 1.01, NR | NR | 0.41 ± 0.36 |  | NR | NR | 5.9 ± 1.7 |
| Siegenthaler et al., 2022 | Provisional crown with a concave contour | 6 | NR, 3.31 ± 1.38, NR | NR | 0.42 ± 0.25 |  | NR | NR | 5.6 ± 2.4 |
| Siegenthaler et al., 2022 | Healing abutment, no provisional crown | 12 | NR, 3.34 ± 1.12, NR | NR | 0.32 ± 0.24 |  | NR | NR | 5.4 ± 2.2 |
| Slagter, Meijer, Bakker, Vissink, & Raghoebar, 2015 | Immediate implant placement + immediate provisionalization | 12 | NR, NR, 3.05 ± 0.83 | NR | 0.73 ± NR | NR | 8.2 ± 0.9 | NR | 7.50 ± 1.59 |
| Slagter, Meijer, Bakker, Vissink, & Raghoebar, 2015 | Immediate implant placement + delayed provisionalization | 12 | NR, NR, 3.00 ± 0.59 | NR | 0.68 ± NR | NR | 9.1 ± 0.8 | NR | 7.40 ± 1.46 |
| Slagter, Meijer, Bakker, Vissink, & Raghoebar, 2016 | Immediate implant placement | 12 | NR, NR, NR | NR | 0.65 | NR | 8.4 ± 1.4 | NR | 7.5 ± 1.6 |
| Slagter, Meijer, Bakker, Vissink, & Raghoebar, 2016 | ARP + delayed implant placement | 12 | NR, NR, NR | NR | 0.53 | NR | 8.1 ± 1.3 | NR | 7.4 ± 1.5 |
| Slagter, Meijer, Hentenaar, Vissink, & Raghoebar, 2021 | ARP + delayed implant placement | 60 | NR, NR, NR | NR | 0.54 ± 0.41 | NR | 8.2 ± 1.4 | NR | 7.5 ± 1.3 |
| Slagter, Meijer, Hentenaar, Vissink, & Raghoebar, 2021 | Immediate implant placement | 60 | NR, NR, NR | NR | 0.71 ± 0.35 | NR | 7.7 ± 1.7 | NR | 7.4 ± 1.9 |
| Slagter, Raghoebar, Hentenaar, Vissink, & Meijer, 2021 | Immediate implant placement + immediate provisionalization | 60 | NR, NR, 2.44 ± 1.04 | NR | 0.71 ± NR | NR | 8.4 ± 1.1 | NR | 7.83 ± 1.69 |
| Slagter, Raghoebar, Hentenaar, Vissink, & Meijer, 2021 | Immediate implant placement + delayed provisionalization | 60 | NR, NR, 2.80 ± 1.04 | NR | 0.52 ± NR | NR | 8.7 ± 1.8 | NR | 7.07 ± 1.79 |
| Small & Tarnow, 2000 | Implant placement | 12 | NR, NR, NR | NR | NR | NR | NR | NR | NR |
| Stefanini et al., 2016 | Implant placement + CTG | 12 | 2.80 ± 0.7, 2.61 ± 0.24, 2.7 ± 0.47 | NR | 2.62 ± NR | NR | NR | NR | NR |
| Stefanini et al., 2016 | Implant placement + CTG | 36 | 3.05 ± 0.76, 2.73 ± 0.25, 2.6 ± 0.5 | NR | 2.69 ± NR | NR | NR | NR | NR |
| Stoupel et al., 2016 | Immediate implant placement and provisionalization (flapless) | 6 | NR, NR, NR | 0.7 ± 0.4 | 0.38 ± NR | NR | NR | NR | NR |
| Stoupel et al., 2016 | Immediate implant placement and provisionalization (with a flap) | 6 | NR, NR, NR | 0.8 ± 0.4 | 0.60 ± NR | NR | NR | NR | NR |
| Stoupel et al., 2016 | Immediate implant placement and provisionalization (flapless) | 12 | NR, NR, NR | 0.7 ± 0.4 | 0.53 ± NR | NR | NR | NR | NR |
| Stoupel et al., 2016 | Immediate implant placement and provisionalization (with a flap) | 12 | NR, NR, NR | 0.8 ± 0.4 | 1.03 ± NR | NR | NR | NR | NR |
| Strasding et al., 2023 | Immediate implant + immediate provisionalization | 12 | NR, NR, NR | NR | NR | NR | NR | NR | NR |
| Strasding et al., 2023 | Early implant placement + GBR | 12 | NR, NR, NR | NR | NR | NR | NR | NR | NR |
| Sun et al., 2020 | Immediate implant placement with Socket-shield approach | 12 | NR, NR, 1.22 ± 0.03 | 1.37 ± 0.23 | NR | NR | NR | 12.2 ± 1.57 | NR |
| Sun et al., 2020 | Immediate implant placement with Socket-shield approach | 6 | NR, NR, NR | 1.37 ± 0.23 | NR | NR | NR | 12 ± 1.77 | NR |
| Sun et al., 2020 | Immediate implant placement | 6 | NR, NR, NR | 1.36 ± 0.16 | NR | NR | NR | 11.73 ± 1.67 | NR |
| Sun et al., 2020 | Immediate implant placement | 12 | NR, NR, 1.90 ± 0.09 | 1.36 ± 0.16 | NR | NR | NR | 11.53 ± 1.73 | NR |
| Takuma, Oishi, Manabe, Yoneda, & Nagata, 2014 | Implant placement | 12 | NR, NR, NR | NR | 1.5 ± 0.4 | NR | NR | NR | NR |
| Tian et al., 2019 | Immediate implant placement + immediate provisionalization | 12 | NR, NR, 3.10 ± 1.12 | NR | NR | NR | NR | NR | NR |
| Todescan et al., 2023 | Immediate implant placement + immediate provisionalization (in patients with thin soft tissue phenotype) | 6 | NR, NR, NR | NR | 0.02 | NR | NR | NR | NR |
| Todescan et al., 2023 | Immediate implant placement + immediate provisionalization (in patients with thick soft tissue phenotype) | 6 | NR, NR, NR | NR | 0.1 | NR | NR | NR | NR |
| Tsuda et al., 2011 | Immediate implant placement (+ CTG) + immediate provisionalization | 12 | NR, NR, NR | NR | 0.14 ± 0.33 | NR | NR | NR | NR |
| van Kesteren, Schoolfield, West, & Oates, 2010 | Immediate implant placement | 6 | NR, NR, NR | NR | 0.1 | NR | NR | NR | NR |
| van Kesteren, Schoolfield, West, & Oates, 2010 | ARP + delayed implant placement | 6 | NR, NR, NR | NR | 0.14 ± 0.33 | NR | NR | NR | NR |
| van Nimwegen et al., 2018 | Immediate implant placement and provisionalization | 12 | NR, NR, 2.44 ± 1.19 | NR | NR | NR | 8.84 ± 1.23 | 11.36 ± 1.65 | NR |
| van Nimwegen et al., 2018 | Immediate implant placement and provisionalization + CTG | 12 | NR, NR, 2.28 ± 0.79 | NR | NR | NR | 8.38 ± 2.28 | 11.28 ± 1.67 | NR |
| Vandeweghe, Cosyn, Thevissen, Van den Berghe, & De Bruyn, 2012 | Implant placement + immediate provisionalization | 12 | NR, NR, NR | NR | 1.2 ± 0.22 | NR | NR | 8.53 ± 1.25 | NR |
| Wanis, Hosny, & ElNahass, 2022 | Immediate implant (dual zone approach) + immediate provisionalization | 12 | 4.55 ± 1.08, 2.06 ± 0.47, NR | NR | NR | NR | 8.7 ± 1.0 | 11.36 ± 1.69 | NR |
| Wanis, Hosny, & ElNahass, 2022 | Immediate implant (dual zone approach) + immediate provisionalization | 6 | 4.57 ± 1.05, 2.05 ± 0.46, NR | NR | NR | NR | NR | 11.09 ± 1.58 | NR |
| Wanis, Hosny, & ElNahass, 2022 | Immediate implant + immediate provisionalization | 12 | 4.20 ± 0.82, 1.90 ± 0.39, NR | NR | NR | NR | 8.5 ± 1.1 | 10.80 ± 1.55 | NR |
| Wanis, Hosny, & ElNahass, 2022 | Immediate implant + immediate provisionalization | 6 | 4.25 ± 0.75, 1.89 ± 0.40, NR | NR | NR | NR | NR | 10.40 ± 1.17 | NR |
| Weber, Kim, Ng, Hwang, & Fiorellini, 2006 | Implant placement and screw-retained restoration | 12 | 4.92 ± 1.04, NR, NR | NR | NR | NR | NR | NR | NR |
| Weber, Kim, Ng, Hwang, & Fiorellini, 2006 | Implant placement and cement-retained restoration | 12 | 4.93 ± 1.41, NR, NR | NR | NR | NR | NR | NR | NR |
| Weber, Kim, Ng, Hwang, & Fiorellini, 2006 | Implant placement and screw-retained restoration | 36 | 4.87 ± 1.21, NR, NR | NR | NR | NR | NR | NR | NR |
| Weber, Kim, Ng, Hwang, & Fiorellini, 2006 | Implant placement and cement-retained restoration | 36 | 5.09 ± 1.3, NR, NR | NR | NR | NR | NR | NR | NR |
| Wohrle, 1998 | Immediate implant placement + immediate provisionalization | 21.7 | NR, NR, NR | NR | NR | NR | NR | NR | NR |
| Yang, Zhou, Zhou, & Man, 2019 | Immediate implant + immediate provisionalization (in presence of buccal bone thickness < 0.5mm) | 12 | NR, NR, NR | 0.41 ± 0.11 | 1.17 ± 0.73 | Buccal bone thickness did not significantly affect ML changes | NR | NR | NR |
| Yang, Zhou, Zhou, & Man, 2019 | Immediate implant + immediate provisionalization (in presence of buccal bone thickness of 0.5-1mm) | 12 | NR, NR, NR | 0.63 ± 0.14 | 0.37 ± 0.39 |  | NR | NR | NR |
| Yang, Zhou, Zhou, & Man, 2019 | Immediate implant + immediate provisionalization (in presence of buccal bone thickness ≥ 1mm) | 12 | NR, NR, NR | 1.29 ± 0.32 | 0.46 ± 0.35 |  | NR | NR | NR |
| Yoshino, Kan, Rungcharassaeng, Roe, & Lozada, 2014 | Immediate implant placement (with CTG) + immediate provisionalization | 6 | NR, NR, NR | NR | 0.09 ± 0.12 | NR | NR | NR | NR |
| Yoshino, Kan, Rungcharassaeng, Roe, & Lozada, 2014 | Immediate implant placement + immediate provisionalization | 6 | NR, NR, NR | NR | 0.12 ± 0.40 | NR | NR | NR | NR |
| Yoshino, Kan, Rungcharassaeng, Roe, & Lozada, 2014 | Immediate implant placement (with CTG) + immediate provisionalization | 12 | NR, NR, NR | NR | 0.01 ± 0.27 | Less recession in the CTG group (p=0.049) | NR | NR | NR |
| Yoshino, Kan, Rungcharassaeng, Roe, & Lozada, 2014 | Immediate implant placement + immediate provisionalization | 12 | NR, NR, NR | NR | 0.14 ± 0.53 |  | NR | NR | NR |
| Yuenyongorarn et al., 2020 | Immediate implant placement (without socket grafting) + immediate provisionalization | 12 | NR, 1.55 ± 0.44, NR | NR | 0.5 | Less ML changes in the group where the socket was grafted compared to the non-grafted socket group (p=0.035) | NR | NR | NR |
| Yuenyongorarn et al., 2020 | Immediate implant placement (with socket grafting) + immediate provisionalization | 12 | NR, 1.68 ± 0.76, NR | NR | 0.41 |  | NR | NR | NR |
| Zembic, Philipp, Hammerle, Wohlwend, & Sailer, 2015 | Implant placement and restoration with zirconia abutment and all-ceramic crown | 132 | NR, NR, 3.4 ± 1.1 | NR | NR | NR | NR | NR | NR |
| Zhang et al., 2017 | Immediate implant (+ bone graft + PRF) + immediate provisionalization | 12 | NR, NR, NR | NR | 1.81 | NR | NR | NR | NR |
| Zhang et al., 2017 | Immediate implant (+ bone graft + PRF) + immediate provisionalization | 24 | NR, NR, NR | NR | 1.68 | NR | NR | NR | NR |
| Zhang et al., 2017 | Immediate implant (+ bone graft + PRF) + immediate provisionalization | 36 | NR, NR, NR | NR | 1.58 | NR | 9.8 | NR | NR |
| Zitzmann, Scharer, & Marinello, 2001 | Implant placement + GBR (with DBBM and CM) | 60 | 3.52 ± 2.03, NR, NR | NR | 1.34 ± 0.79 | NR | NR | NR | NR |
| Zitzmann, Scharer, & Marinello, 2001 | Implant placement + GBR (with DBBM and PTFE membrane) | 60 | 2.99 ± 1.73, NR, NR | NR | 1.51 ± 0.96 | NR | NR | NR | NR |
| Zitzmann, Scharer, & Marinello, 2001 | Implant placement (without bone augmentation) | 60 | 2.70 ± 1.86, NR, NR | NR | 1.24 ± 0.8 | NR | NR | NR | NR |
| Zuiderveld et al., 2021 | Immediate implant placement (with CTG) + immediate provisionalization | 12 | NR, NR, NR | 2.38 ± 0.81 | NR | NR | NR | NR | NR |
| Zuiderveld et al., 2021 | Immediate implant placement + immediate provisionalization | 12 | NR, NR, NR | 2.28 ± 0.92 | NR | NR | NR | NR | NR |
| Zuiderveld, Meijer, den Hartog, Vissink, & Raghoebar, 2018 | Immediate implant placement + immediate provisionalization | 12 | NR, NR, 2.5 ± 1.2 | NR | 0.8 ± NR | NR | NR | NR | 6.8 ± 1.5 |
| Zuiderveld, Meijer, den Hartog, Vissink, & Raghoebar, 2018 | Immediate implant placement (with CTG) + immediate provisionalization | 12 | NR, NR, 2.3 ± 0.9 | NR | 0.9 ± NR | NR | NR | NR | 6.4 ± 1.5 |
| Zuiderveld, Meijer, Vissink, & Raghoebar, 2018 | ARP + Implant placement + CTG | 12 | NR, NR, 3.1 ± 1.2 | NR | NR | NR | 8.5 | NR | 7 ± 2.4 |
| Zuiderveld, Meijer, Vissink, & Raghoebar, 2018 | ARP + Implant placement | 12 | NR, NR, 2.9 ± 0.9 | NR | NR | NR | 8.7 | NR | 6.6 ± 1.5 |
| Zuiderveld, Meijer, Vissink, & Raghoebar, 2018 | ARP + Implant placement + CMX | 12 | NR, NR, 2.3 ± 1.0 | NR | NR | NR | 9.3 | NR | 6.1 ± 1.7 |

**Legend.** BBT: buccal bone thickness; BL: baseline; CTG: connective tissue graft; mPES: modified pink esthetic score; MT: mucosal thickness; NR: not reported; OR: odds ratio; PES: pink esthetic score; PSTD: peri-implant soft tissue dehiscence; Rx: radiographic; SSD: statistically significant difference.

**Supplementary Table 10.** Risk indicators for PSTD and MREC reported in the included studies.

| **Peri-implant soft tissue dehiscence (PSTD)** | | | | | |
| --- | --- | --- | --- | --- | --- |
| Parameters associated with PSTD | | OR | 95% CI | p-value | Reference |
| Implant position | Implant Buccally positioned | 17.2 | 3.1 – 100.0 | 0.001 | (Cosyn et al., 2012) |
|  |  | / | / | 0.015 | (Elise G. Zuiderveld et al., 2014) |
|  | Fixture angle (“proclined”) | 0.9 | 0.8 – 1 | 0.02 | (Nisapakultorn et al., 2010) |
|  | Implant depth | 2.3 | 1.3 – 4.2 | 0.005 | (Nisapakultorn et al., 2010) |
| Soft tissue-related parameters | Absence of/ limited KM width | 0.73 | 0.55 – 0.97 | 0.03 | (Tavelli et al., 2022) |
|  | Limited MT (MT1) | 0.11 | 0.04 – 0.24 | <0.001 | (Tavelli et al., 2022) |
|  | Limited MT (MT3) | 0.34 | 0.14 – 0.82 | 0.01 | (Tavelli et al., 2022) |
|  | Thin soft tissue phenotype | 18.8 | 2 – 180 | 0.01 | (Nisapakultorn et al., 2010) |
| Bone-related parameters | Buccal bone dehiscence | 1.41 | 1.02 – 1.95 | 0.02 | (Tavelli et al., 2022) |
|  |  | 1.3 | 1 – 1.7 | 0.03 | (Nisapakultorn et al., 2010) |
|  | Interproximal bone levels | 3.4 | 1.3 – 8.8 | 0.01 | (Nisapakultorn et al., 2010) |
|  | Pre-implant bone augmentation procedures | / | / | 0.005 | (Elise G. Zuiderveld et al., 2014) |
| Others | Years in function of the implant | 1.4 | 0.71 – 2.73 | 0.001 | (Tavelli et al., 2022) |
|  | Presence adjacent implant(s) | 10.9 | 2.98 – 40.20 | <0.001 | (Tavelli et al., 2022) |
|  | Papilla esthetic index | / | / | 0.001 | (Hof et al., 2014) |
|  | Subjective esthetic index | / | / | 0.021 | (Hof et al., 2014) |
| **Mucosal Recession (MREC)** | | | | | |
| Parameters associated with MREC | | OR | 95% CI | p-value | Reference |
| Implant position | Implant Buccally positioned | 14.67 | 2.12 – 101.55 | 0.006 | (Romandini et al., 2021) |
|  |  | 34.65 | 6.31 – 90.31 | <0.001 | (Sanz-Martin et al., 2020) |
| Soft tissue-related parameters | Absence of/ limited KM width | 0.06 | 0.01 – 0.30 | 0.001 | (Sanz-Martin et al., 2020) |
|  |  | 3.20 | 1.03 – 9.90 | <0.05 | (Kungsadalpipob et al., 2020) |
|  |  | / | / | 0.03 | (Adibrad et al., 2009) |
|  |  | / | / | 0.038 | (Manopattanasoontorn et al., 2021) |
|  |  | / | / | 0.03 | (Obreja et al., 2021) |
|  |  | / | / | <0.001 | (Ramanauskaite et al., 2020) |
|  |  | / | / | 0.001 | (Tur & Sarıbaş, 2023) |
|  | Limited MT | / | / | 0.021 | (Mailoa et al., 2018) |
|  | Thin soft tissue phenotype | 8.31 | 1.75 – 39.41 | 0.008 | (Romandini et al., 2021) |
|  |  | / | / | 0.001 | (Tur & Sarıbaş, 2023) |
| Bone-related parameters | Buccal bone dehiscence | / | / | 0.024 | (Sanz-Martin et al., 2020) |
| Others | One-piece implant | 11.89 | 1.43 – 39.00 | 0.022 | (Sanz-Martin et al., 2020) |
|  | Missing adjacent teeth | 0.08 | 0.02 – 0.36 | 0.001 | (Romandini et al., 2021) |
|  | Lack of abutment | 0.12 | 0.02 – 0.71 | 0.02 | (Romandini et al., 2021) |
|  | Bleeding on probing | 2.60 | 1.82 – 3.72 | <0.001 | (Nettemu et al., 2021) |

**Legend.** CI: confidence interval; KM: keratinized mucosa; MREC: mucosal recession; MT: mucosal thickness; OR: odds ratio; PSTD: peri-implant soft tissue dehiscence.

**Supplementary Table 11.** Summary of the parameters correlated with PSTD/MREC/ML apical changes in the included prospective studies.

| **Parameters correlated to Peri-implant soft tissue dehiscence (PSTD) depth** |
| --- |
| Non-grafted sites (compared to connective tissue graft) (p<0.05, Frizzera et al., 2019)  Thin phenotype (p≤0.002, Migliorati et al., 2015)  Interproximal crestal bone levels (p=0.02, Malchiodi et al., 2013)  Buccally positioned implant (p=0.032, Chen et al., 2007)  Absence of keratinized mucosa (p<0.001, Hof et al., 2015)  Time after implant placement (p=0.001, Hof et al., 2015)  Lower Pink Esthetic Scores (p<0.001, Hof et al., 2015)  Lower Papilla index Scores (p<0.001, Hof et al., 2015) |
| **Parameters correlated to Mucosal Recession (MREC) depth** |
| Absence of KM (p<0.05, Bonino et al., 2018)  KM width < 2 mm (p<0.01 [Crespi et al., 2019] and p<0.001 [Schrott et al., 2009])  Lower patients-reported esthetic scores (p<0.05, Bonino et al., 2018) |
| **Parameters correlated to Mucosal Level (ML) apical changes** |
| Non-grafted sockets (p=0.035, Yuenyongorarn et al., 2014)  Flap approach (vs flapless) (p=0.023, Raes et al., 2011)  Sites that did not receive antibiotics (p=0.011, Hosseini et al., 2015)  Buccally positioned implant (descriptive analysis, Seyssens et al., 2020)  High insertion torque (p<0.001 [Barone et al., 2016 and Marconcini et al., 2018])  Non-grafted sites (without CTG) (p=0.049 [Yoshino et al., 2014], p<0.001 [Kobayashi et al., 2020], and descriptive analysis [Seyssens et al., 2020])  Limited soft tissue thickness (p=0.01, Lee et al. 2020)  Thin phenotype (p<0.001, Kan et al., 2011)  Mandibular sites (p=0.01 Marconcini et al., 2018)  Central incisor position (descriptive analysis, Seyssens et al., 2020)  Age (older patients) (p=0.04, Jacobs et al., 2020)  Smokers (p=0.004, Raes et al., 2015)  Bone defect morphology (descriptive analysis, Kan et al., 2007)  Presurgical BBD width (p=0.04, Mizuno et al., 2022)  Presurgical BBD depth (p=0.03 [Mizuno et al., 2022] and p=0.03 [Lee et al. 2020])*  Presurgical BBT for posterior sites (p≤0.001, Farronato et al., 2020)*, but not for anterior implants (Farronato et al., 2020)  Convex emergence profile (OR 12.6, 95% CI 1.82-88.48, p=0.01 [Siegenthaler et al., 2022], p=0.02 [Bushari et al., 2021], and descriptive analysis, [Seyssens et al., 2020]) |

**Legend**. BBD: buccal bone dehiscence; BBT: buccal bone thickness; CTG: connective tissue graft; KM: keratinized mucosa; *Other studies failed to find significant correlations between buccal bone thickness and ML changes (Arora & Ivanovski et al., 2017, Barone et al., 2015, Raes et al., 2018, Yang et al., 2019), nor between buccal bone dehiscence and ML changes (Raes et al., 2018).

**Supplementary Table 12.** General characteristics of the included studies reporting on soft tissue augmentation and treatment of soft tissue dehiscence.

| **Article** | **Intervention** | **Follow-up (months)** | **PSTD depth BL, PSTD depth Final** | **mean PSTD coverage (%)** | **MREC depth BL, MREC depth final** | **mean MREC coverage (%)** |
| --- | --- | --- | --- | --- | --- | --- |
| Anderson, Inglehart, El-Kholy, Eber, & Wang, 2014 | PSTD tx with CAF + hADM | 6 | NR, NR | NR | 1.16 ± NR, 0.83 ± NR | 28 ± NR |
| Anderson, Inglehart, El-Kholy, Eber, & Wang, 2014 | PSTD tx with CAF + SCTG | 6 | NR, NR | NR | 0.72 ± NR, 0.43 ± NR | 40 ± NR |
| Burkhardt, Joss, & Lang, 2008 | PSTD tx with CAF + SCTG | 6 | 3 ± 0.8, 1.02 ± NR | 66 ± 18 | NR, NR | NR |
| Clem et al., 2023 | STA with envelope flap + SCTG | 12 | NR, NR | NR | 0.24 ± 0.53, 0.12 ± 0.29 | 50 |
| Clem et al., 2023 | STA with envelope flap + CMX | 12 | NR, NR | NR | 0.32 ± 1.02, 0.29 ± 0.68 | 9.4 |
| Lorenzo, Garcia, Orsini, Martin, & Sanz, 2012 | STA with APF + SCTG | 6 | NR, NR | NR | 0.67 ± 1.07, 1.17 ± 1.27 | NR |
| Lorenzo, Garcia, Orsini, Martin, & Sanz, 2012 | STA with APF + CMX | 6 | NR, NR | NR | 1.08 ± 1.44, 1.5 ± 1.08 | NR |
| Oh, Ji, & Azad, 2020 | STA with APF + FGG | 12, 48 | NR, NR | NR | 0.9 ± 0.8, 0.36 ± NR | 60 ± NR |
| Oh, Ji, & Azad, 2020 | Control (no STA) | 12, 48 | NR, NR | NR | 0.8 ± 1, 1.03 ± NR | NR |
| Roccuzzo, Dalmasso, Pittoni, & Roccuzzo, 2019 | PSTD tx with CAF + CTG | 60 | NR, NR | NR | 1.9 ± 0.7, 0.2 ± 0.3 | 86 ± NR |
| Roccuzzo, Gaudioso, Bunino, & Dalmasso, 2014 | PSTD tx with CAF + CTG | 12 | NR, NR | NR | 2 ± 0.7, 0.3 ± 0.3 | 85 ± NR |
| Schallhorn, McClain, Charles, Clem, & Newman, 2015 | STA with CAF + CMX | 6 | NR, NR | NR | 1.5 ± 1.5, 1.5 ± 1.4 | 0 |
| Tavelli, Majzoub, et al., 2023 | PSTD tx with CAF + CTG | 6 | 2.46 ± 0.87, 0.29 ± 0.47 | 88.2 ± NR | NR, NR | NR |
| Tavelli, Majzoub, et al., 2023 | PSTD tx with TUN + CTG | 6 | 2.36 ± 0.46, 0.89 ± 0.9 | 62.3 ± NR | NR, NR | NR |
| Tavelli, Majzoub, et al., 2023 | PSTD tx with CAF + CTG | 12 | 2.46 ± 0.87, 0.25 ± 0.47 | 90.23 ± 19.85 | NR, NR | NR |
| Tavelli, Majzoub, et al., 2023 | PSTD tx with TUN + CTG | 12 | 2.36 ± 0.46, 1.00 ± 0.88 | 59.76 ± 34.94 | NR, NR | NR |
| Tavelli, Zucchelli, et al., 2023 | PSTD tx with CTGs for horizontal and vertical STA and submerged healing | 12 | 2.60 ± 0.61, 0.35 ± 0.47 | 85.14 ± 21.11 | NR, NR | NR |
| Zucchelli et al., 2013 | PSTD tx with CAF + CTG | 12 | 2.72 ± 0.68, 0.10 ± 0.44 | 96.3 ± NR | NR, NR | NR |
| Zucchelli et al., 2018 | PSTD tx with CAF + CTG | 60 | 2.72 ± 0.68, 0.023 ± NR | 99.2 ± NR | NR, NR | NR |

**Legend.** APF: apically positioned flap; BL: baseline; CAF: coronally advanced flap; CTG: connective tissue graft (obtained from the de-epithelialization of an epithelialized palatal graft); CMX: collagen matrix; FGG: free gingival graft; hADM: human acellular dermal matrix; MREC: mucosal recession; NR: not reported; PSTD: peri-implant soft tissue dehiscence; SCTG: sub-epithelial connective tissue graft; STA: soft tissue augmentation; TUN: tunnel technique; tx: treatment.

**Supplementary Table 13.** Clinical, esthetic, and patient-reported outcomes of soft tissue augmentation and treatment of soft tissue dehiscence at implant sites.

| **Article** | **Intervention** | **KM BL, KM final** | **MT1 BL, MT1 final** | **MT3 BL, MT3 final** | **PD BL, PD final** | **Patient-reported esthetics after tx (0-10 VAS)** | **Professional esthetic evaluation** |
| --- | --- | --- | --- | --- | --- | --- | --- |
| Anderson, Inglehart, El-Kholy, Eber, & Wang, 2014 | PSTD tx with CAF + hADM | NR, NR | 1.52 ± NR, 3.02 ± NR | 2.16 ± NR, 3.31 ± NR | NR, NR | NR | NR |
| Anderson, Inglehart, El-Kholy, Eber, & Wang, 2014 | PSTD tx with CAF + SCTG | NR, NR | 1.61 ± NR, 2.87 ± NR | 2.04 ± NR, 3 ± NR | NR, NR | NR | NR |
| Burkhardt, Joss, & Lang, 2008 | PSTD tx with CAF + SCTG | 1.3 ± 1, 1.1 ± 0.5 | NR, NR | NR, NR | 2.8 ± 1, 3 ± 0.8 | NR | NR |
| Clem et al., 2023 | STA with envelope flap + SCTG | 2.36 ± 1.60, 2.83 ± 1.17 | NR, NR | NR, NR | 2.73 ± 1.19, 2.54 ± 0.96 | 9 ± 1.58 | PES 10.41 ± 2.56 |
| Clem et al., 2023 | STA with envelope flap + CMX | 2.72 ± 1.61, 3.02 ± 1.36 | NR, NR | NR, NR | 3 ± 1.15, 2.67 ± 0.96 | 8.96 ± 2.30 | PES 11.33 ± 1.92 |
| Lorenzo, Garcia, Orsini, Martin, & Sanz, 2012 | STA with APF + SCTG | 0.42 ± 0.51, 2.75 ± 1.55 | NR, NR | NR, NR | 2.08 ± 0.99, 2.08 ± 1.08 | NR | NR |
| Lorenzo, Garcia, Orsini, Martin, & Sanz, 2012 | STA with APF + CMX | 0.50 ± 0.52, 2.8 ± 0.42 | NR, NR | NR, NR | 2 ± 0.73, 1.6 ± 0.52 | NR | NR |
| Oh, Ji, & Azad, 2020 | STA with APF + FGG | 0.5 ± 0.6, 3.6 ± NR | NR, NR | NR, NR | NR, NR | NR | NR |
| Oh, Ji, & Azad, 2020 | Control (no STA) | 0.4 ± 0.5, 0.4 ± 0.5 | NR, NR | NR, NR | NR, NR | NR | NR |
| Roccuzzo, Dalmasso, Pittoni, & Roccuzzo, 2019 | PSTD tx with CAF + CTG | NR, NR | NR, NR | NR, NR | 2.7 ± 0.4, 2.9 ± 0.6 | 9.5 ± 0.8 | VAS (operator) 8.1 ± 0.9 |
| Roccuzzo, Gaudioso, Bunino, & Dalmasso, 2014 | PSTD tx with CAF + CTG | NR, NR | NR, NR | NR, NR | 2.7 ± 0.4, 3.1 ± 0.5 | NR | VAS (operator) 8.5 ± 0.3 |
| Schallhorn, McClain, Charles, Clem, & Newman, 2015 | STA with envelope flap + CMX | 1.7 ± 1.8, 2.1 ± 1.0 | 1.5 ± 0.5, 2.2 ± 0.9 | NR, NR | 3.5 ± 1.7, 3 ± 1.6 | 9 ± 2 | NR |
| Tavelli, Majzoub, et al., 2023 | PSTD tx with CAF + CTG | 1.96 ± 1.35, 3.61 ± 1.06 | 1.18 ± 0.40, 2.62 ± 0.52 | 1.28 ± 0.29, 2.86 ± 0.59 | 2.14 ± 0.41, 2.04 ± 0.41 | NR | Total IDES 7 ± 2.45 |
| Tavelli, Majzoub, et al., 2023 | PSTD tx with TUN + CTG | 1.79 ± 0.99, 2.61 ± 1.36 | 1.42 ± 0.42, 2.41 ± 0.35 | 1.56 ± 0.47, 2.66 ± 0.61 | 2.04 ± 0.46, 2.04 ± 0.31 | NR | Total IDES 4.93 ± 2.53 |
| Tavelli, Majzoub, et al., 2023 | PSTD tx with CAF + CTG | 1.96 ± 1.35, 4.54 ± 1.05 | 1.18 ± 0.40, 2.65 ± 0.5 | 1.28 ± 0.29, 2.94 ± 0.59 | 2.14 ± 0.41, 2.18 ± 0.54 | 9.74 | Total IDES 7.29 ± 2.58 |
| Tavelli, Majzoub, et al., 2023 | PSTD tx with TUN + CTG | 1.79 ± 0.99, 3.43 ± 1.22 | 1.42 ± 0.42, 2.44 ± 0.34 | 1.56 ± 0.47, 2.82 ± 0.67 | 2.04 ± 0.46, 2.25 ± 0.51 | 7.17 | Total IDES 4.86 ± 2.41 |
| Tavelli, Zucchelli, et al., 2023 | PSTD tx with CTGs for horizontal and vertical STA and submerged healing | 2.40 ± 0.77, 3.55 ± 0.60 | 0.93 ± 0.12, 2.51 ± 0.53 | NR, NR | 2.35 ± 0.47, 2.17 ± 0.41 | 8.83 | Total IDES 6.90 ± 2.33 |
| Zucchelli et al., 2013 | PSTD tx with CAF + CTG | 1.72 ± 0.61, 2.30 ± 0.52 | 0.92 ± 0.27, 2.50 ± 0.39 | NR, NR | 1.87 ± 0.51, 2.27 ± 0.69 | 8.75 ± 1.02 | mPES 8.75 ± 0.78 |
| Zucchelli et al., 2018 | PSTD tx with CAF + CTG | 1.72 ± 0.61, 3 ± NR | 0.92 ± 0.27, 2.60 ± NR | NR, NR | 1.87 ± 0.51, 2 ± NR | 8.95 ± 0.91 | mPES 8.84 ± 0.9 |

**Legend.** APF: apically positioned flap; BL: baseline; CAF: coronally advanced flap; CTG: connective tissue graft (obtained from the de-epithelialization of an epithelialized palatal graft); CMX: collagen matrix; FGG: free gingival graft; hADM: human acellular dermal matrix; IDES: implant soft tissue dehiscence coverage esthetic score; KM: keratinized mucosa width; mPES: modified pink esthetic score; MREC: mucosal recession; NR: not reported; PD: probing depth; PES: pink esthetic score; PSTD: peri-implant soft tissue dehiscence; SCTG: sub-epithelial connective tissue graft; STA: soft tissue augmentation; TUN: tunnel technique; tx: treatment; VAS: visual analogue scale.

**Supplementary Table 14.** Risk of bias assessment of the included cross sectional studies using The Joanna Briggs Institute (JBI) Critical Appraisal checklist for analytical cross sectional studies

| **Study** | **D1** | **D2** | **D3** | **D4** | **D5** | **D6** | **D7** | **D8** | **Overall risk of bias** |
| --- | --- | --- | --- | --- | --- | --- | --- | --- | --- |
| Able, Sartori, Younes, & Bombarda, 2021 | Yes | Yes | Yes | Yes | No | No | Yes | Yes | High |
| Adibrad, Shahabuei, & Sahabi, 2009 | Yes | Yes | Yes | Yes | No | No | Yes | Yes | High |
| Apaza-Bedoya et al., 2023 | Yes | Yes | Yes | Yes | Yes | Yes | Yes | Yes | Low |
| Chang, Wennstrom, Odman, & Andersson, 1999 | Yes | Yes | Yes | Yes | No | No | Yes | Yes | High |
| Cosyn & De Rouck, 2009 | Yes | Yes | Yes | Yes | Yes | Yes | Yes | Yes | Low |
| Cosyn, Sabzevar, & De Bruyn, 2012 | Yes | Yes | Yes | Yes | Yes | Yes | Yes | Yes | Low |
| Duque, Aristizabal, Londono, Castro, & Alvarez, 2016 | Yes | Yes | Unclear | Yes | Yes | Unclear | Unclear | Yes | Unclear |
| Hof et al., 2014 | Yes | Yes | Yes | Yes | Yes | Yes | Yes | Yes | Low |
| Iglhaut et al., 2021 | Yes | Yes | Yes | Yes | Yes | Yes | Yes | Yes | Low |
| Kungsadalpipob et al., 2020 | Yes | Yes | Yes | Yes | Yes | Unclear | Unclear | Yes | Unclear |
| Mailoa et al., 2018 | Yes | Yes | Yes | Yes | Yes | Unclear | Yes | Yes | Unclear |
| Manopattanasoontorn et al., 2021 | Yes | Yes | Yes | Yes | Yes | Unclear | Yes | Yes | Unclear |
| Nettemu et al., 2021 | Yes | Yes | Yes | Yes | Yes | Unclear | Yes | Yes | Unclear |
| Nisapakultorn, Suphanantachat, Silkosessak, & Rattanamongkolgul, 2010 | Yes | Yes | Yes | Yes | Yes | Yes | Yes | Yes | Low |
| Obreja et al., 2021 | Yes | Yes | Yes | Yes | Yes | Yes | Yes | Yes | Low |
| Obreja et al., 2022 | Yes | Yes | Yes | Yes | Yes | Yes | Yes | Yes | Low |
| Parvini et al., 2023 | Yes | Yes | Yes | Yes | Yes | Yes | Yes | Yes | Low |
| Ramanauskaite et al., 2020 | Yes | Yes | Yes | Yes | Yes | Yes | Yes | Yes | Low |
| Romandini et al., 2021 | Yes | Yes | Yes | Yes | Yes | Yes | Yes | Yes | Low |
| Sanz-Martin et al., 2020 | Yes | Yes | Yes | Yes | Yes | Yes | Yes | Yes | Low |
| Suphanantachat, Thovanich, & Nisapakultorn, 2012 | Yes | Yes | Yes | Yes | Yes | Yes | Yes | Yes | Low |
| Tavelli et al., 2022 | Yes | Yes | Yes | Yes | Yes | Yes | Yes | Yes | Low |
| Thoma, Maggetti, Waller, Hammerle, & Jung, 2019 | Yes | Yes | Yes | Yes | Yes | Yes | Yes | Yes | Low |
| Tur & Saribas, 2023 | Yes | Yes | Yes | Yes | Yes | Yes | Yes | Yes | Low |
| Ueno et al., 2016 | Yes | Yes | Yes | Yes | No | No | Yes | Yes | High |
| Zuiderveld, den Hartog, Vissink, Raghoebar, & Meijer, 2014 | Yes | Yes | Yes | Yes | No | No | Yes | Yes | High |

**Legend.** D: domain; D1: Were the criteria for inclusion in the sample clearly defined?; D2: Were the study subjects and the setting described in detail?; D3: Was the exposure measured in a valid and reliable way?; D4: Were objective, standard criteria used for measurement of the condition?; D5: Were confounding factors identified?; D6: Were strategies to deal with confounding factors stated?; D7: Were the outcomes measured in a valid and reliable way?; D8: Was appropriate statistical analysis used? NA: not applicable; UN: unclear

**Supplementary Table 15.** Risk of bias assessment of the included randomized clinical trials using the risk-of-bias 2 (ROB2) tool.

| **Study** | **D1** | **D2** | **D3** | **D4** | **D5** | **Overall risk of bias** |
| --- | --- | --- | --- | --- | --- | --- |
| Anderson, Inglehart, El-Kholy, Eber, & Wang, 2014 | Unclear | Unclear | Low | Low | Low | Unclear |
| Atef, El Barbary, Dahrous, & Zahran, 2021 | Unclear | Low | Low | Low | Low | Unclear |
| Barone et al., 2016 | Low | Low | Low | Low | Low | Low |
| Benitez Silva et al., 2022 | Low | Low | Low | Low | Low | Low |
| Bianchi & Sanfilippo, 2004 | Unclear | Unclear | Unclear | Low | Low | Unclear |
| Bittner, Planzos, Volchonok, Tarnow, & Schulze-Spate, 2020 | Low | Low | Low | Low | Low | Low |
| Bittner, Schulze-Spate, et al., 2020 | Low | Low | Low | Low | Low | Low |
| Bittner et al., 2019 | Low | Low | Low | Low | Low | Low |
| Block et al., 2009 | Unclear | Unclear | Unclear | Low | Low | Unclear |
| Bressan et al., 2017 | Unclear | Low | Low | Low | Low | Unclear |
| Bushahri et al., 2021 | Low | Low | Low | Low | Low | Low |
| Canullo, Caneva, & Tallarico, 2017 | Low | Low | Low | Low | Low | Low |
| Canullo, Iurlaro, & Iannello, 2009 | Unclear | Unclear | Low | Low | Low | Unclear |
| Cardaropoli, Gaveglio, Gherlone, & Cardaropoli, 2014 | Unclear | Unclear | Low | Low | Low | Unclear |
| Cecchinato, Lops, Salvi, & Sanz, 2015 | Low | Low | Low | Low | Low | Low |
| Chan et al., 2019 | Low | Low | Low | Low | Low | Low |
| Clem et al., 2023 | Low | Low | Low | Low | Low | Low |
| Cooper et al., 2015 | Low | Low | Low | Low | Low | Low |
| Cooper et al., 2019 | Low | Low | Low | Low | Low | Low |
| Cordaro, Torsello, & Roccuzzo, 2009 | Unclear | Unclear | Low | Low | Low | Unclear |
| Cosyn et al., 2022 | Low | Low | Low | Low | Low | Low |
| Crespi et al., 2019 | Unclear | Unclear | High | Low | Low | High |
| Crespi, Cappare, & Gherlone, 2010b | Unclear | Unclear | High | Low | Low | High |
| D'Elia et al., 2017 | Unclear | Low | Low | Low | Low | Unclear |
| de Albornoz et al., 2014 | Low | Low | Low | Low | Low | Low |
| De Bruyckere et al., 2020 | Low | Low | Low | Low | Low | Low |
| De Rouck, Collys, Wyn, & Cosyn, 2009 | Low | Low | Low | Low | Low | Low |
| de Siqueira et al., 2017 | Low | Low | Low | Low | Low | Low |
| de Siqueira et al., 2020 | Low | Low | Low | Low | Low | Low |
| Degidi, Nardi, Daprile, & Piattelli, 2014 | Low | Low | Low | Low | Low | Low |
| den Hartog, Raghoebar, Stellingsma, Vissink, & Meijer, 2011 | Low | Low | Low | Low | Low | Low |
| Esposito et al., 2017 | Unclear | Unclear | Low | Low | Low | Unclear |
| Farronato et al., 2021 | High | High | Unclear | Low | Low | High |
| Fernandes, Marques, Borges, & Montero, 2023 | Low | Low | Low | Low | Low | Low |
| Ferrari, Cagidiaco, Garcia-Godoy, Goracci, & Cairo, 2015 | Unclear | Unclear | Low | Low | Low | Unclear |
| Frizzera et al., 2019 | Low | Low | Low | Low | Low | Low |
| Gallucci, Grutter, Nedir, Bischof, & Belser, 2011 | Low | Low | Low | Low | Low | Low |
| Ghallab et al., 2023 | Unclear | Unclear | Low | Low | Low | Unclear |
| Girlanda et al., 2019 | Unclear | Unclear | Low | Low | Low | Unclear |
| Givens et al., 2015 | Unclear | Unclear | Low | Low | Low | Unclear |
| Guarnieri, Di Nardo, Di Giorgio, Miccoli, & Testarelli, 2019 | Unclear | Low | Low | Low | Low | Unclear |
| Guarnieri et al., 2022 | Unclear | Unclear | Low | Low | Low | Unclear |
| Hall et al., 2007 | Unclear | Low | High | Low | Low | High |
| Hosseini et al., 2015 | Unclear | Unclear | Low | Low | Low | Unclear |
| Humm et al., 2023 | Low | Low | Low | Low | Low | Low |
| Huynh-Ba et al., 2019 | Low | Low | Low | Low | Low | Low |
| Jacobs, Zadeh, De Kok, & Cooper, 2020 | Unclear | Unclear | Low | Low | Low | Unclear |
| Jeffcoat, McGlumphy, Reddy, Geurs, & Proskin, 2003 | Unclear | Low | High | Low | Low | High |
| Koh et al., 2011 | Low | Low | Low | Low | Low | Low |
| Lee et al., 2020 | Low | Low | Low | Low | Low | Low |
| Lee et al., 2023 | Low | Low | Low | Low | Low | Low |
| Lindeboom, Tjiook, & Kroon, 2006 | Unclear | Low | Unclear | Low | Low | Unclear |
| Lorenzo, Garcia, Orsini, Martin, & Sanz, 2012 | Low | Low | Unclear | Low | Low | Unclear |
| Lowy et al., 2019 | Unclear | Low | Unclear | Low | Low | Unclear |
| Marconcini et al., 2018 | Low | Low | Low | Low | Low | Low |
| Mau et al., 2019 | Low | Low | Low | Low | Low | Low |
| Meijndert et al., 2017 | Low | Low | Low | Low | Low | Low |
| Meijndert, Raghoebar, Meijer, & Vissink, 2008 | Low | Low | Low | Low | Low | Low |
| Migliorati, Amorfini, Signori, Biavati, & Benedicenti, 2015 | Low | Low | Low | Low | Low | Low |
| Molina, Sanz-Sanchez, Martin, Blanco, & Sanz, 2017 | Low | Low | Low | Low | Low | Low |
| Oh, Ji, & Azad, 2020 | Low | Low | Low | Low | Low | Low |
| Perez et al., 2020 | Low | Low | Low | Low | Low | Low |
| Pieri, Aldini, Marchetti, & Corinaldesi, 2011 | Low | Low | Low | Low | Low | Low |
| Puisys et al., 2022 | Unclear | Low | Unclear | Low | Low | Unclear |
| Rivara et al., 2020 | Unclear | Low | Unclear | Low | Low | Unclear |
| Romanos, Malmstrom, Feng, Ercoli, & Caton, 2014 | Unclear | Low | Unclear | Low | Low | Unclear |
| Salvi et al., 2020 | Low | Low | Low | Low | Low | Low |
| Sanz Martin, Benic, Hammerle, & Thoma, 2016 | Low | Low | Low | Low | Low | Low |
| Sapata et al., 2018 | Low | Low | Low | Low | Low | Low |
| Schropp & Isidor, 2008 | Unclear | Low | Unclear | Low | Low | Unclear |
| Schwarz, Sahm, & Becker, 2012 | Low | Low | Low | Low | Low | Low |
| Schwarz, Schmucker, & Becker, 2017 | Low | Low | Low | Low | Low | Low |
| Siegenthaler et al., 2022 | Low | Low | Unclear | Low | Low | Unclear |
| Slagter, Meijer, Bakker, Vissink, & Raghoebar, 2016 | Unclear | Low | Low | Low | Low | Unclear |
| Slagter, Meijer, Bakker, Vissink, & Raghoebar, 2015 | Unclear | Low | Unclear | Low | Low | Unclear |
| Slagter, Meijer, Hentenaar, Vissink, & Raghoebar, 2021 | Unclear | Low | Unclear | Low | Low | Unclear |
| Slagter, Raghoebar, Hentenaar, Vissink, & Meijer, 2021 | Unclear | Low | Unclear | Low | Low | Unclear |
| Stoupel et al., 2016 | Low | Low | Low | Low | Low | Low |
| Strasding et al., 2023 | Low | Low | Low | Low | Low | Low |
| Sun et al., 2020 | Low | Low | Low | Low | Low | Low |
| Tavelli et al., 2023 | Low | Low | Low | Low | Low | Low |
| van Kesteren, Schoolfield, West, & Oates, 2010 | Low | Low | Low | Low | Low | Low |
| van Nimwegen et al., 2018 | Low | Low | Low | Low | Low | Low |
| Wanis, Hosny, & ElNahass, 2022 | Low | Low | Unclear | Low | Low | Unclear |
| Yoshino, Kan, Rungcharassaeng, Roe, & Lozada, 2014 | Unclear | Low | Low | Low | Low | Unclear |
| Yuenyongorarn et al., 2020 | Unclear | Low | Low | Low | Low | Unclear |
| Ziebolz et al., 2017 | Unclear | Low | Low | Low | Low | Unclear |
| Zitzmann, Scharer, & Marinello, 2001 | Unclear | Low | Unclear | Unclear | Low | Unclear |
| Zuiderveld, Meijer, den Hartog, Vissink, & Raghoebar, 2018 | Low | Low | Low | Low | Low | Low |
| Zuiderveld, Meijer, Vissink, & Raghoebar, 2018 | Low | Low | Low | Low | Low | Low |
| Zuiderveld et al., 2021 | Low | Low | Low | Low | Low | Low |

**Legend.** D: domain; D1: Bias arising from the randomization process; D2: Bias due to deviations from intended intervention; D3: Bias due to missing outcome data; D4: Bias in measurement of the outcome; D5: Bias in selection of the reported result.

**Supplementary Table 16.** Risk of bias assessment of the included non-randomized interventional studies using the ROBINS-I tool.

| **Study** | **D1** | **D2** | **D3** | **D4** | **D5** | **D6** | **D7** | **Overall risk of bias** |
| --- | --- | --- | --- | --- | --- | --- | --- | --- |
| Andersson, Odman, Lindvall, & Branemark, 1998 | High | Low | Low | Unclear | High | High | Low | High |
| Arora & Ivanovski, 2017 | Low | Low | Low | Low | Low | Low | Low | Low |
| Arora & Ivanovski, 2018 | Low | Low | Low | Low | Low | Low | Low | Low |
| Arora, Khzam, Roberts, Bruce, & Ivanovski, 2017 | Low | Low | Low | Low | Low | Low | Low | Low |
| Barone, Toti, Quaranta, Derchi, & Covani, 2015 | Low | Low | Unclear | Low | Low | Low | Low | Unclear |
| Bengazi, Wennströnm & Lekholm, 1996 | Low | Low | Low | Unclear | Unclear | Low | Low | Unclear |
| Benic et al., 2012 | Low | Low | Low | Low | Low | Low | Low | Low |
| Benic et al., 2017 | Low | Low | Low | Low | Low | Low | Low | Low |
| Blanes, Bernard, Blanes, & Belser, 2007 | Unclear | Low | Low | Unclear | Unclear | Low | Low | Unclear |
| Bonino et al., 2018 | Low | Low | Low | Low | Low | Low | Low | Low |
| Bragger, Hammerle, & Lang, 1996 | Unclear | Low | Low | Unclear | Unclear | Low | Low | Unclear |
| Brunello et al., 2022 | Low | Low | Low | Low | Low | Low | Low | Low |
| Burkhardt, Joss, & Lang, 2008 | Low | Low | Low | Low | Low | Low | Low | Low |
| Buser et al., 2009 | Low | Low | Low | Low | Low | Low | Low | Low |
| Buser et al., 2011 | Low | Low | Low | Low | Low | Low | Low | Low |
| Buser, Chappuis, Bornstein, et al., 2013 | Low | Low | Low | Low | Low | Low | Low | Low |
| Buser, Chappuis, Kuchler, et al., 2013 | Low | Low | Low | Low | Low | Low | Low | Low |
| Cabello, Rioboo, & Fabrega, 2013 | Unclear | Low | Low | Unclear | Unclear | Low | Low | Unclear |
| Chen, Darby, & Reynolds, 2007 | Low | Low | Low | Low | Low | Low | Low | Low |
| Chung, Rungcharassaeng, Kan, Roe, & Lozada, 2011 | Unclear | Low | Low | Unclear | Unclear | Low | Low | Unclear |
| Cooper et al., 2010 | Low | Low | Low | Low | Low | Low | Low | Low |
| Cooper et al., 2014 | Low | Low | Low | Low | Low | Low | Low | Low |
| Cosyn et al., 2011 | Low | Low | Low | Low | Low | Low | Low | Low |
| Cosyn et al., 2016 | Low | Low | Low | Low | Low | Low | Low | Low |
| Cosyn, De Bruyn, & Cleymaet, 2013 | Low | Low | Low | Low | Low | Low | Low | Low |
| Cosyn, Pollaris, Van der Linden, & De Bruyn, 2015 | Low | Low | Low | Low | Low | Low | Low | Low |
| Covani, Canullo, Toti, Alfonsi, & Barone, 2014 | Unclear | Low | Low | Low | Low | Low | Low | Unclear |
| Crespi, Cappare, & Gherlone, 2010a | Unclear | Low | Low | Unclear | Unclear | Low | Low | Unclear |
| Crespi, Cappare, Gherlone, & Romanos, 2012 | Unclear | Low | Low | Unclear | Low | Low | Low | Unclear |
| Crespi, Cappare, Polizzi, & Gherlone, 2015 | Unclear | Low | Unclear | Low | Low | Low | Low | Unclear |
| da Rosa, Rosa, Francischone, & Sotto-Maior, 2014 | Unclear | Low | Low | Low | Low | Unclear | Unclear | Unclear |
| De Bruyn et al., 2013 | Low | Low | Low | Low | Low | Low | Low | Low |
| De Rouck, Collys, & Cosyn, 2008 | Low | Low | Low | Low | Low | Low | Low | Low |
| Eghbali et al., 2018 | Low | Low | Low | Low | Low | Low | Low | Low |
| Ekfeldt, Eriksson, & Johansson, 2003 | Unclear | Low | Unclear | Low | Low | Low | Low | Unclear |
| Farrag & Khamis, 2023 | Low | Low | Unclear | Unclear | Unclear | Low | Low | Unclear |
| Farronato et al., 2020 | Unclear | Low | Unclear | Low | Low | Low | Low | Unclear |
| Fenner, Hammerle, Sailer, & Jung, 2016 | Low | Low | Low | Low | Low | Low | Low | Low |
| Finelle, Popelut, Knafo, & Sanz Martin, 2021 | Low | Low | Low | Low | Low | Low | Low | Low |
| Furhauser et al., 2017 | Unclear | Low | Low | Low | Low | Low | Low | Unclear |
| Garaicoa-Pazmino et al., 2021 | Unclear | Low | Low | Low | Low | Low | Low | Unclear |
| Gomez-Meda et al., 2022 | Unclear | Low | Low | Low | Low | Low | Unclear | Unclear |
| Grandi, Guazzi, Samarani, & Grandi, 2013 | Unclear | Low | Low | Low | Low | Low | Unclear | Unclear |
| Grassi et al., 2015 | Unclear | Low | Low | Low | Low | Low | Unclear | Unclear |
| Groenendijk, Bronkhorst, & Meijer, 2021 | Low | Low | Low | Low | Low | Low | Low | Low |
| Guarnieri, Ceccarelli, Ricci, & Testori, 2018 | Unclear | Low | Low | Low | Low | Unclear | Low | Unclear |
| Guarnieri, Ceccherini, & Grande, 2015 | Unclear | Low | Low | Low | Low | Unclear | Low | Unclear |
| Guarnieri, Di Nardo, Gaimari, Miccoli, & Testarelli, 2019 | High | Low | Low | Low | Low | Unclear | Low | High |
| Hattingh, De Bruyn, Van Weehaeghe, Hommez, & Vandeweghe, 2020 | Low | Low | Low | Low | Low | Low | Low | Low |
| Hof et al., 2015 | Low | Low | Low | Low | Low | Low | Low | Low |
| Hollander et al., 2016 | Low | Low | Low | Low | Low | Low | Low | Low |
| Iorio-Siciliano et al., 2016 | Low | Low | Low | Low | Low | Low | Low | Low |
| Jemt, Ahlberg, Henriksson, & Bondevik, 2006 | High | Low | Low | Low | Low | High | Unclear | High |
| Jung et al., 2016 | Low | Low | Low | Low | Low | Low | Low | Low |
| Kan, Rungcharassaeng, & Lozada, 2003 | Unclear | Low | Low | Low | Low | Unclear | Low | Unclear |
| Kan, Rungcharassaeng, Lozada, & Zimmerman, 2011 | Unclear | Low | Low | Low | Low | Unclear | Low | Unclear |
| Kan, Rungcharassaeng, Sclar, & Lozada, 2007 | Unclear | Low | Low | Low | Low | Unclear | Low | Unclear |
| Karoussis et al., 2004 | Low | Low | Low | Low | Low | Low | Low | Low |
| Kobayashi et al., 2020 | Low | Low | Low | Low | Low | Low | Low | Low |
| Lago, da Silva, Gude, & Rilo, 2017 | Unclear | Low | Low | Low | Low | Unclear | Low | Unclear |
| Lilet et al., 2022 | Low | Low | Low | Low | Low | Unclear | Low | Unclear |
| Liu et al., 2019 | Low | Low | Low | Low | Low | Low | Low | Low |
| Lops et al., 2015 | Unclear | Low | Unclear | Low | Low | Unclear | Low | Unclear |
| Lops, Romeo, Chiapasco, Procopio, & Oteri, 2013 | Unclear | Low | Low | Low | Low | Unclear | Low | Unclear |
| Lorenz et al., 2019 | Unclear | Low | Low | Low | Low | Unclear | Low | Unclear |
| Lorenz et al., 2022 | Unclear | Low | Low | Low | Low | Unclear | Low | Unclear |
| Malchiodi, Cucchi, Ghensi, & Nocini, 2013 | Unclear | Low | Unclear | Low | Low | Unclear | Low | Unclear |
| Meijndert, Raghoebar, Vissink, & Meijer, 2022 | Low | Low | Low | Low | Low | Low | Low | Low |
| Mizuno, Nakano, Shimomoto, Fujita, & Ishigaki, 2022 | Low | Low | Low | Low | Low | Low | Low | Low |
| Munoz-Camara, Gilbel-Del Aguila, Pardo-Zamora, & Camacho-Alonso, 2020 | Unclear | Unclear | Low | Low | Low | Unclear | Low | Unclear |
| Oates, West, Jones, Kaiser, & Cochran, 2002 | High | Low | Low | Low | Low | Low | Low | High |
| Parvini, Muller, Cafferata, Schwarz, & Obreja, 2022 | Low | Low | Low | Low | Low | Low | Low | Low |
| Pieri, Aldini, Marchetti, & Corinaldesi, 2013 | Low | Low | Low | Low | Low | Unclear | Low | Unclear |
| Pohl, Furhauser, Haas, & Pohl, 2020 | Low | Low | Low | Low | Low | Low | Low | Low |
| Proussaefs, Kan, Lozada, Kleinman, & Farnos, 2002 | Low | Low | Low | Low | Low | High | Low | High |
| Qian et al., 2023 | Low | Low | Low | Low | Low | Unclear | Low | Unclear |
| Raes et al., 2015 | Low | Low | Low | Low | Low | Low | Low | Low |
| Raes et al., 2018 | Low | Low | Low | Low | Low | Low | Low | Low |
| Raes, Cosyn, & De Bruyn, 2013 | Low | Low | Low | Low | Low | Low | Low | Low |
| Raes, Cosyn, Crommelinck, Coessens, & De Bruyn, 2011 | Low | Low | Low | Low | Low | Low | Low | Low |
| Ribeiro dos Reis et al., 2023 | Low | Low | Low | Low | Low | Low | Low | Low |
| Roccuzzo, Dalmasso, Pittoni, & Roccuzzo, 2019 | Low | Low | Low | Low | Low | Unclear | Low | Unclear |
| Roccuzzo, Gaudioso, Bunino, & Dalmasso, 2014 | Low | Low | Low | Low | Low | Unclear | Low | Unclear |
| Roccuzzo, Grasso, & Dalmasso, 2016 | Low | Low | Low | Low | Low | Unclear | Low | Unclear |
| Santing, Raghoebar, Vissink, den Hartog, & Meijer, 2013 | Low | Low | Low | Low | Unclear | Unclear | Low | Low |
| Sanz-Martin, Encalada, Sanz-Sanchez, Aracil, & Sanz, 2019 | Low | Low | Low | Low | Low | Low | Low | Low |
| Schallhorn, McClain, Charles, Clem, & Newman, 2015 | Low | Low | Low | Low | Low | Low | Low | Low |
| Schrott, Jimenez, Hwang, Fiorellini, & Weber, 2009 | Low | Low | Low | Low | Low | Low | Low | Low |
| Seyssens, Eghbali, & Cosyn, 2020 | Low | Low | Low | Low | Low | Low | Low | Low |
| Small & Tarnow, 2000 | Low | Low | Low | Low | Unclear | Low | Low | Unclear |
| Stefanini et al., 2016 | Low | Low | Low | Low | Low | Low | Low | Low |
| Takuma, Oishi, Manabe, Yoneda, & Nagata, 2014 | Low | Low | Low | Low | Unclear | Low | Low | Unclear |
| Tavelli, Zucchelli, et al., 2023 | Low | Low | Low | Low | Low | Low | Low | Low |
| Tian et al., 2019 | Low | Unclear | Low | Low | Unclear | Low | Low | Unclear |
| Todescan et al., 2023 | Low | Low | Low | Low | Unclear | Low | Low | Unclear |
| Tsuda et al., 2011 | Low | Unclear | Low | Low | Unclear | Low | Low | Unclear |
| Vandeweghe, Cosyn, Thevissen, Van den Berghe, & De Bruyn, 2012 | Low | Low | Low | Low | Low | Low | Low | Low |
| Weber, Kim, Ng, Hwang, & Fiorellini, 2006 | Low | Low | Low | Low | Low | Low | Low | Low |
| Wohrle, 1998 | Unclear | Unclear | Low | Low | High | Low | Low | High |
| Yang, Zhou, Zhou, & Man, 2019 | Low | Low | Low | Low | High | Low | Low | High |
| Zembic, Philipp, Hammerle, Wohlwend, & Sailer, 2015 | Low | Low | Low | Low | Unclear | Low | Low | Unclear |
| Zhang et al., 2017 | Low | Low | Low | Low | Unclear | Low | Low | Unclear |
| Zucchelli et al., 2013 | Low | Low | Low | Low | Low | Low | Low | Low |
| Zucchelli et al., 2018 | Low | Low | Low | Low | Low | Low | Low | Low |

**Legend.** D: domain; D1: Bias due to confounding; D2: Bias in selection of participants; D3: Bias in classification of intervention; D4: Bias due to deviations from intended interventions; D5: Bias due to missing data; D6: Bias in measurement of outcomes; D7: Bias in selection of the reported result.

**Supplementary Table 17.** Outcomes of the studies reporting on PSTD/MREC treatment and peri-implant soft tissue augmentation. Weighted averages were performed when the same treatment approach was executed in multiple studies.

| **Intervention** | **Studies (n), patients (N), implants (n)** | **Mean follow-up (months)** | **PSTD coverage**  **(mean ± SD) (%)** | **MREC coverage**  **(mean ± SD) (%)** | **KM width gain (mean ± SD)** | **MT gain**  **(mean ± SD)** | **PD change**  **(mean ± SD)** |
| --- | --- | --- | --- | --- | --- | --- | --- |
| **PSTD/MREC treatment** | | | | | | | |
| CAF + CTG | 6, 92, 92 | 28 | 92.7 ± 6.3 | 85.5 ± 0.7 | 1.4 ± 0.8 | 1.6 ± 0.9 | 0.2 ± 0.2 |
| CAF + SCTG | 2, 17, 17 | 6 | 66 ± N/A | 40 ± N/A | -0.2 ± N/A | 1.3 ± N/A | 0.2 ± N/A |
| CAF + ADM | 1, 6, 6 | 6 | N/A | 28 ± N/A | NR | 1.5 ± N/A | NR |
| TUN + CTG | 1, 14, 14 | 12 | 59.8 ± 34.9 | N/A | 1.6 | 1.0 ± 0.5 | 0.1 ± 0.5 |
| **Soft tissue augmentation** | | | | | | | |
| APF + FGG | 1, 11, 18 | 48 | N/A | 60 ± NR | 3.1 ± 1.3 | NR | NR |
| APF + SCTG | 1, 12, 12 | 6 | N/A | -74.6 ± NR | 2.3 ± NR | NR | 0 |
| APF + CMX | 1, 12, 12 | 6 | N/A | -38.9 ± NR | 2.3 ± NR | NR | -0.4 ± NR |
| Envelope flap + SCTG | 1, 29, 29 | 12 | N/A | 50 ± NR | 0.47 ± NR | 1.2 ± 0.5 | -0.19 ± NR |
| Envelope flap + CMX | 1, 30, 35 | 6 | N/A | 0 | 0.4 ± NR | 0.7 ± 0.8 | -0.5 ± 1.0 |
| Envelope flap + VCMX | 1, 28, 28 | 12 | N/A | 9.4 ± NR | 0.3 ± NR | 1.2 ± 0.7 | -0.3 ± NR |

**Legend**. ADM: human acellular dermal matrix; APF: apically positioned flap; CAF: coronally advanced flap; CMX: collagen matrix; CTG: connective tissue graft, obtained from the de-epithelialization of a soft tissue graft either from the lateral palate or the maxillary tuberosity; FGG: free gingival graft; KM: keratinized mucosa; MREC: mucosal recession; MT: mucosal thickness; N/A: not available; NR: not reported; PD: probing depth; PSTD: peri-implant soft tissue dehiscence; SCTG: subepithelial connective tissue graft; SD: standard deviation; TUN: tunnel technique; VCMX: volume-stable cross-linked collagen matrix.

REFERENCES

Abd-Elrahman, A., Shaheen, M., Askar, N., & Atef, M. (2020). Socket shield technique vs conventional immediate implant placement with immediate temporization. Randomized clinical trial. *Clin Implant Dent Relat Res, 22*(5), 602-611. doi:10.1111/cid.12938

Abdelsamie, P. M., Elarab, A. E., Ibrahim, R. O., & Rahman, A. R. A. (2022). Facial gingival level evaluation with and without connective tissue graft using tunnel technique on single immediate implants in the esthetic zone: A randomized controlled clinical trial. *JOURNAL OF OSSEOINTEGRATION, 14*(2), 69-80. doi:10.23805/JO.2022.14.11

Able, F. B., Sartori, A. D., Younes, I. A., & Bombarda, N. H. C. (2021). Behavior of Implants and Oral Tissues in Maxillary Fixed Prostheses, with a Follow-up of 1 to 12 Years: Analytical Cross-Sectional Study. *INTERNATIONAL JOURNAL OF ORAL & MAXILLOFACIAL IMPLANTS, 36*(4), 799-806. doi:10.11607/jomi.8542

Abrahamsson, P., Andersson, G., Wälivaara, D., & Isaksson, S. (2011). Soft-tissue expansion before local bone reconstruction using a new technique for measuring soft tissue profile stability. *Journal of Oral and Maxillofacial Surgery, 69*(9), e-50. doi:10.1016/j.joms.2011.06.097

Acham, S., Rugani, P., Truschnegg, A., Wildburger, A., Wegscheider, W. A., & Jakse, N. (2017). Immediate loading of four interforaminal implants supporting a locator-retained mandibular overdenture in the elderly. Results of a 3-year randomized, controlled, prospective clinical study. *Clin Implant Dent Relat Res, 19*(5), 895-900. doi:10.1111/cid.12513

Adibrad, M., Shahabuei, M., & Sahabi, M. (2009). SIGNIFICANCE OF THE WIDTH OF KERATINIZED MUCOSA ON THE HEALTH STATUS OF THE SUPPORTING TISSUE AROUND IMPLANTS SUPPORTING OVERDENTURES. *JOURNAL OF ORAL IMPLANTOLOGY, 35*(5), 232-237. doi:10.1563/AAID-JOI-D-09-00035.1

Amato, F., Amato, G., Campriani, S., Contessi, M., D'Amato, F., Fiorentini, A. G., . . . Spedicato, G. A. (2022). The Role of Different Healing Abutment Sizes in Tissue Volume Preservation of Molar Sockets After Immediate Tooth Extraction and Implant Placement: A Multicenter Clinical Study. *Int J Oral Maxillofac Implants, 37*(5), 891-904. doi:10.11607/jomi.9607

Amato, F., & Cracknell, T. J. (2022). Single-Tooth Immediate Placement and Provisionalization with Subcrestally Angulated Implants in Sites with Hard and Soft Tissue Facial Dehiscence in the Esthetic Zone: An Observational Study with 2 to 5 Years of Follow-up. *Int J Periodontics Restorative Dent, 42*(5), e133-e142. doi:10.11607/prd.6049

Amorfini, L., Storelli, S., Mosca, D., Scanferla, M., & Romeo, E. (2018). Comparison of Cemented vs Screw-Retained, Customized Computer-Aided Design/Computer-Assisted Manufacture Zirconia Abutments for Esthetically Located Single-Tooth Implants: A 10-Year Randomized Prospective Study. *INTERNATIONAL JOURNAL OF PROSTHODONTICS, 31*(4), 359-366. doi:10.11607/ijp.5305

Anderson, L. E., Inglehart, M. R., El-Kholy, K., Eber, R., & Wang, H. L. (2014). Implant Associated Soft Tissue Defects in the Anterior Maxilla: A Randomized Control Trial Comparing Subepithelial Connective Tissue Graft and Acellular Dermal Matrix Allograft. *Implant Dentistry, 23*(4), 416-425. doi:10.1097/ID.0000000000000122

Andersson, B., Odman, P., Lindvall, A. M., & Branemark, P. I. (1998). Cemented single crowns on osseointegrated implants after 5 years: Results from a prospective study on CeraOne. *INTERNATIONAL JOURNAL OF PROSTHODONTICS, 11*(3), 212-218.

Apaza-Bedoya, K., Galarraga-Vinueza, M. E., Correa, B. B., Schwarz, F., Bianchini, M. A., & Magalhaes Benfatti, C. A. (2023). Prevalence, risk indicators, and clinical characteristics of peri-implant mucositis and peri-implantitis for an internal conical connection implant system: A multicenter cross-sectional study. *J Periodontol*. doi:10.1002/JPER.23-0355

Arnhart, C., Kielbassa, A. M., Martinez-de Fuentes, R., Goldstein, M., Jackowski, J., Lorenzoni, M., . . . Strub, J. R. (2012). Comparison of variable-thread tapered implant designs to a standard tapered implant design after immediate loading. A 3-year multicentre randomised controlled trial. *Eur J Oral Implantol, 5*(2), 123-136.

Arora, H., & Ivanovski, S. (2017). Correlation between pre-operative buccal bone thickness and soft tissue changes around immediately placed and restored implants in the maxillary anterior region: A 2-year prospective study. *Clin Oral Implants Res, 28*(10), 1188-1194. doi:10.1111/clr.12939

Arora, H., & Ivanovski, S. (2018). Immediate and early implant placement in single-tooth gaps in the anterior maxilla: A prospective study on ridge dimensional, clinical, and aesthetic changes. *Clinical oral implants research, 29*(11), 1143-1154. doi:10.1111/clr.13378

Arora, H., Khzam, N., Roberts, D., Bruce, W. L., & Ivanovski, S. (2017). Immediate implant placement and restoration in the anterior maxilla: Tissue dimensional changes after 2-5 year follow up. *Clin Implant Dent Relat Res, 19*(4), 694-702. doi:10.1111/cid.12487

Artzi, Z., Carmeli, G., & Kozlovsky, A. (2006). A distinguishable observation between survival and success rate outcome of hydroxyapatite-coated implants in 5-10 years in function. *Clinical oral implants research, 17*(1), 85-93. doi:10.1111/j.1600-0501.2005.01178.x

Assaf, J. H., Assaf, D. D., Antoniazzi, R. P., Osório, L. B., & França, F. M. (2017). Correction of Buccal Dehiscence During Immediate Implant Placement Using the Flapless Technique: A Tomographic Evaluation. *J Periodontol, 88*(2), 173-180. doi:10.1902/jop.2016.160276

Atef, M., El Barbary, A., Dahrous, M. S. E., & Zahran, A. F. (2021). Comparison of the soft and hard peri-implant tissue dimensional changes around single immediate implants in the esthetic zone with socket shield technique versus using xenograft: A randomized controlled clinical trial. *Clin Implant Dent Relat Res, 23*(3), 456-465. doi:10.1111/cid.13008

Barone, A., Alfonsi, F., Derchi, G., Tonelli, P., Toti, P., Marchionni, S., & Covani, U. (2016). The Effect of Insertion Torque on the Clinical Outcome of Single Implants: A Randomized Clinical Trial. *Clin Implant Dent Relat Res, 18*(3), 588-600. doi:10.1111/cid.12337

Barone, A., Toti, P., Quaranta, A., Derchi, G., & Covani, U. (2015). The Clinical Outcomes of Immediate Versus Delayed Restoration Procedures on Immediate Implants: A Comparative Cohort Study for Single-Tooth Replacement. *Clin Implant Dent Relat Res, 17*(6), 1114-1126. doi:10.1111/cid.12225

Bartols, A., Kasprzyk, S., Walther, W., & Korsch, M. (2018). Lateral alveolar ridge augmentation with autogenous block grafts fixed at a distance versus resorbable Poly-D-L-Lactide foil fixed at a distance: A single-blind, randomized, controlled trial. *Clin Oral Implants Res, 29*(8), 843-854. doi:10.1111/clr.13303

Bengazi, F., Wennstrom, J. L., & Lekholm, U. (1996). Recession of the soft tissue margin at oral implants. A 2-year longitudinal prospective study. *Clin Oral Implants Res, 7*(4), 303-310. doi:10.1034/j.1600-0501.1996.070401.x

Benic, G. I., Ge, Y., Gallucci, G. O., Jung, R. E., Schneider, D., & Hammerle, C. H. (2017). Guided bone regeneration and abutment connection augment the buccal soft tissue contour: 3-year results of a prospective comparative clinical study. *Clin Oral Implants Res, 28*(2), 219-225. doi:10.1111/clr.12786

Benic, G. I., Mokti, M., Chen, C. J., Weber, H. P., Hammerle, C. H., & Gallucci, G. O. (2012). Dimensions of buccal bone and mucosa at immediately placed implants after 7 years: a clinical and cone beam computed tomography study. *Clin Oral Implants Res, 23*(5), 560-566. doi:10.1111/j.1600-0501.2011.02253.x

Benitez Silva, C. G., Sapata, V. M., Llanos, A. H., Romano, M. M., Jung, R. E., Hammerle, C. H. F., . . . Romito, G. A. (2022). Peri-implant tissue changes at sites treated with alveolar ridge preservation in the aesthetic zone: Twenty-two months follow-up of a randomized clinical trial. *Journal of Clinical Periodontology, 49*(1), 39-47. doi:10.1111/jcpe.13570

Bianchi, A. E., & Sanfilippo, F. (2004). Single-tooth replacement by immediate implant and connective tissue graft: a 1-9-year clinical evaluation. *Clin Oral Implants Res, 15*(3), 269-277. doi:10.1111/j.1600-0501.2004.01020.x

Bienz, S. P., Ruales-Carrera, E., Lee, W. Z., Hämmerle, C. H. F., Jung, R. E., & Thoma, D. S. (2023). Early implant placement in sites with ridge preservation or spontaneous healing: histologic, profilometric, and CBCT analyses of an exploratory RCT. *J Periodontal Implant Sci*. doi:10.5051/jpis.2300460023

Bienz, S. P., Sailer, I., Sanz-Martin, I., Jung, R. E., Hammerle, C. H. F., & Thoma, D. S. (2017). Volumetric changes at pontic sites with or without soft tissue grafting: a controlled clinical study with a 10-year follow-up. *Journal of Clinical Periodontology, 44*(2), 178-184. doi:10.1111/jcpe.12651

Bittner, N., Planzos, L., Volchonok, A., Tarnow, D., & Schulze-Spate, U. (2020). Evaluation of Horizontal and Vertical Buccal Ridge Dimensional Changes After Immediate Implant Placement and Immediate Temporization With and Without Bone Augmentation Procedures: Short-Term, 1-Year Results. A Randomized Controlled Clinical Trial. *Int J Periodontics Restorative Dent, 40*(1), 83-93. doi:10.11607/prd.4152

Bittner, N., Schulze-Spate, U., Cleber, S., Da Silva, J. D., Kim, D. M., Tarnow, D., . . . Gil, M. S. (2020). Comparison of Peri-implant Soft Tissue Color with the Use of Pink-Neck vs Gray Implants and Abutments Based on Soft Tissue Thickness: A 6-Month Follow-up Study. *INTERNATIONAL JOURNAL OF PROSTHODONTICS, 33*(1), 29-38. doi:10.11607/ijp.6205

Bittner, N., Schulze-Spate, U., Silva, C., Da Silva, J. D., Kim, D. M., Tarnow, D., . . . Ishikawa-Nagai, S. (2019). Changes of the alveolar ridge dimension and gingival recession associated with imp an position and tissue phenotype with immediate implant placement: A randomised controlled clinical trial. *INTERNATIONAL JOURNAL OF ORAL IMPLANTOLOGY, 12*(4), 469-480.

Blanes, R. J., Bernard, J. P., Blanes, Z. M., & Belser, U. C. (2007). A 10-year prospective study of ITI dental implants placed in the posterior region. I: Clinical and radiographic results. *Clinical oral implants research, 18*(6), 699-706. doi:10.1111/j.1600-0501.2006.01306.x

Block, M. S., Mercante, D. E., Lirette, D., Mohamed, W., Ryser, M., & Castellon, P. (2009). Prospective evaluation of immediate and delayed provisional single tooth restorations. *J Oral Maxillofac Surg, 67*(11 Suppl), 89-107. doi:10.1016/j.joms.2009.07.009

Bogaerde, L. V., Rangert, B., & Wendelhag, I. (2005). Immediate/early function of Branemark System (R) TiUnite (TM) implants in fresh extraction sockets in maxillae and posterior mandibles: An 18-month prospective clinical study. *Clinical implant dentistry and related research, 7*, S121-S130. doi:10.1111/j.1708-8208.2005.tb00084.x

Bonino, F., Steffensen, B., Natto, Z., Hur, Y., Holtzman, L. P., & Weber, H. P. (2018). Prospective study of the impact of peri-implant soft tissue properties on patient-reported and clinically assessed outcomes. *Journal of Periodontology, 89*(9), 1025-1032. doi:10.1002/JPER.18-0031

Bonnet, F., Karouni, M., & Antoun, H. (2018). Esthetic evaluation of periimplant soft tissue of immediate single-implant placement and provisionalization in the anterior maxilla. *INTERNATIONAL JOURNAL OF ESTHETIC DENTISTRY, 13*(3), 378-392.

Boven, G. C., Meijer, H. J., Slot, W., Vissink, A., & Raghoebar, G. M. (2015). Does a large dehiscent implant surface at placement affect the 5-year treatment outcome? An assessment of implants placed to support a maxillary overdenture. *J Craniomaxillofac Surg, 43*(9), 1758-1762. doi:10.1016/j.jcms.2015.08.009

Brägger, U., Hämmerle, C. H., & Lang, N. P. (1996). Immediate transmucosal implants using the principle of guided tissue regeneration (II). A cross-sectional study comparing the clinical outcome 1 year after immediate to standard implant placement. *Clin Oral Implants Res, 7*(3), 268-276. doi:10.1034/j.1600-0501.1996.070309.x

Brägger, U., Hugel-Pisoni, C., Bürgin, W., Buser, D., & Lang, N. P. (1996). Correlations between radiographic, clinical and mobility parameters after loading of oral implants with fixed partial dentures. A 2-year longitudinal study. *Clin Oral Implants Res, 7*(3), 230-239. doi:10.1034/j.1600-0501.1996.070305.x

Bressan, E., Grusovin, M. G., D'Avenia, F., Neumann, K., Sbricoli, L., Luongo, G., & Esposito, M. (2017). The influence of repeated abutment changes on peri-implant tissue stability: 3-year post-loading results from a multicentre randomised controlled trial. *Eur J Oral Implantol, 10*(4), 373-390.

Brunello, G., Rauch, N., Becker, K., Hakimi, A. R., Schwarz, F., & Becker, J. (2022). Two-piece zirconia implants in the posterior mandible and maxilla: A cohort study with a follow-up period of 9 years. *Clinical oral implants research, 33*(12), 1233-1244. doi:10.1111/clr.14005

Bruno, V., O'Sullivan, D., Badino, M., & Catapano, S. (2014). Preserving soft tissue after placing implants in fresh extraction sockets in the maxillary esthetic zone and a prosthetic template for interim crown fabrication: a prospective study. *J Prosthet Dent, 111*(3), 195-202. doi:10.1016/j.prosdent.2013.09.008

Burkhardt, R., Joss, A., & Lang, N. P. (2008). Soft tissue dehiscence coverage around endosseous implants: a prospective cohort study. *Clinical oral implants research, 19*(5), 451-457. doi:10.1111/j.1600-0501.2007.01497.x

Buser, D., Chappuis, V., Bornstein, M. M., Wittneben, J. G., Frei, M., & Belser, U. C. (2013). Long-Term Stability of Contour Augmentation With Early Implant Placement Following Single Tooth Extraction in the Esthetic Zone: A Prospective, Cross-Sectional Study in 41 Patients With a 5- to 9-Year Follow-Up. *Journal of Periodontology, 84*(11), 1517-1527. doi:10.1902/jop.2013.120635

Buser, D., Chappuis, V., Kuchler, U., Bornstein, M. M., Wittneben, J. G., Buser, R., . . . Belser, U. C. (2013). Long-term stability of early implant placement with contour augmentation. *J Dent Res, 92*(12 Suppl), 176s-182s. doi:10.1177/0022034513504949

Buser, D., Halbritter, S., Hart, C., Bornstein, M. A., Grutter, L., Chappuis, V., & Belser, U. C. (2009). Early Implant Placement With Simultaneous Guided Bone Regeneration Following Single-Tooth Extraction in the Esthetic Zone: 12-Month Results of a Prospective Study With 20 Consecutive Patients. *Journal of Periodontology, 80*(1), 152-162. doi:10.1902/jop.2009.080360

Buser, D., Wittneben, J., Bornstein, M. M., Grutter, L., Chappuis, V., & Belser, U. C. (2011). Stability of Contour Augmentation and Esthetic Outcomes of Implant-Supported Single Crowns in the Esthetic Zone: 3-Year Results of a Prospective Study With Early Implant Placement Postextraction. *Journal of Periodontology, 82*(3), 342-349. doi:10.1902/jop.2010.100408

Bushahri, A., Kripfgans, O. D., George, F., Wang, I. C., Wang, H. L., & Chan, H. L. (2021). Facial mucosal level of single immediately placed implants with either immediate provisionalization or delayed restoration: An intermediate-term study. *J Periodontol, 92*(9), 1213-1221. doi:10.1002/jper.20-0746

Cabello, G., Rioboo, M., & Fábrega, J. G. (2013). Immediate placement and restoration of implants in the aesthetic zone with a trimodal approach: soft tissue alterations and its relation to gingival biotype. *Clin Oral Implants Res, 24*(10), 1094-1100. doi:10.1111/j.1600-0501.2012.02516.x

Cairo, F., Barbato, L., Tonelli, P., Batalocco, G., Pagavino, G., & Nieri, M. (2017). Xenogeneic collagen matrix versus connective tissue graft for buccal soft tissue augmentation at implant site. A randomized, controlled clinical trial. *Journal of Clinical Periodontology, 44*(7), 769-776. doi:10.1111/jcpe.12750

Cairo, F., Nieri, M., Cavalcanti, R., Landi, L., Rupe, A., Sforza, N. M., . . . Barbato, L. (2020). Marginal soft tissue recession after lateral guided bone regeneration at implant site: A long-term study with at least 5 years of loading. *Clin Oral Implants Res, 31*(11), 1116-1124. doi:10.1111/clr.13658

Cannizzaro, G., Felice, P., Leone, M., Checchi, V., & Esposito, M. (2011). Flapless versus open flap implant surgery in partially edentulous patients subjected to immediate loading: 1-year results from a split-mouth randomised controlled trial. *Eur J Oral Implantol, 4*(3), 177-188.

Canullo, L., Caneva, M., & Tallarico, M. (2017). Ten-year hard and soft tissue results of a pilot double-blinded randomized controlled trial on immediately loaded post-extractive implants using platform-switching concept. *Clin Oral Implants Res, 28*(10), 1195-1203. doi:10.1111/clr.12940

Canullo, L., Iurlaro, G., & Iannello, G. (2009). Double-blind randomized controlled trial study on post-extraction immediately restored implants using the switching platform concept: soft tissue response. Preliminary report. *Clin Oral Implants Res, 20*(4), 414-420. doi:10.1111/j.1600-0501.2008.01660.x

Canullo, L., Tronchi, M., Kawakami, S., Iida, T., Signorini, L., & Mordini, L. (2019). Horizontal Bone Augmentation in the Anterior Esthetic Area of the Maxilla Using a Flap Design Adapted from Mucogingival Surgery in Association with PLA Membrane and beta-TCP. *INTERNATIONAL JOURNAL OF PERIODONTICS & RESTORATIVE DENTISTRY, 39*(2), 195-202. doi:10.11607/prd.3894

Cardaropoli, D., Gaveglio, L., Gherlone, E., & Cardaropoli, G. (2014). Soft tissue contour changes at immediate implants: a randomized controlled clinical study. *Int J Periodontics Restorative Dent, 34*(5), 631-637. doi:10.11607/prd.1845

Cecchinato, D., Lops, D., Salvi, G. E., & Sanz, M. (2015). A prospective, randomized, controlled study using OsseoSpeed implants placed in maxillary fresh extraction socket: soft tissues response. *Clin Oral Implants Res, 26*(1), 20-27. doi:10.1111/clr.12295

Chan, H. L., George, F., Wang, I. C., Suárez López Del Amo, F., Kinney, J., & Wang, H. L. (2019). A randomized controlled trial to compare aesthetic outcomes of immediately placed implants with and without immediate provisionalization. *J Clin Periodontol, 46*(10), 1061-1069. doi:10.1111/jcpe.13171

Chang, M., & Wennstrom, J. L. (2010). Peri-implant soft tissue and bone crest alterations at fixed dental prostheses: a 3-year prospective study. *Clinical oral implants research, 21*(5), 527-534. doi:10.1111/j.1600-0501.2009.01874.x

Chang, M., Wennstrom, J. L., Odman, P., & Andersson, B. (1999). Implant supported single-tooth replacements compared to contralateral natural teeth. Crown and soft tissue dimensions. *Clin Oral Implants Res, 10*(3), 185-194. doi:10.1034/j.1600-0501.1999.100301.x

Chappuis, V., Rahman, L., Buser, R., Janner, S. F. M., Belser, U. C., & Buser, D. (2018). Effectiveness of Contour Augmentation with Guided Bone Regeneration: 10-Year Results. *JOURNAL OF DENTAL RESEARCH, 97*(3), 266-274. doi:10.1177/0022034517737755

Chen, S. T., Darby, I. B., & Reynolds, E. C. (2007). A prospective clinical study of non-submerged immediate implants: clinical outcomes and esthetic results. *Clinical oral implants research, 18*(5), 552-562. doi:10.1111/j.1600-0501.2007.01388.x

Chen, S. T., Darby, I. B., Reynolds, E. C., & Clement, J. G. (2009). Immediate Implant Placement Postextraction Without Flap Elevation. *Journal of Periodontology, 80*(1), 163-172. doi:10.1902/jop.2009.080243

Cho, Y. B., Moon, S. J., Chung, C. H., & Kim, H. J. (2011). Resorption of labial bone in maxillary anterior implant. *JOURNAL OF ADVANCED PROSTHODONTICS, 3*(2), 85-89. doi:10.4047/jap.2011.3.2.85

Chu, S. J., Saito, H., Levin, B. P., Baumgarten, H., Egbert, N., Wills, M. J., . . . Nevins, M. (2021). Outcomes of a 1-Year Prospective Single-Arm Cohort Study Using a Novel Macro-Hybrid Implant Design in Extraction Sockets: Part 1. *Int J Periodontics Restorative Dent, 41*(4), 499-508. doi:10.11607/prd.5709

Chu, S. J., Saito, H., Östman, P. O., Levin, B. P., Reynolds, M. A., & Tarnow, D. P. (2020). Immediate Tooth Replacement Therapy in Postextraction Sockets: A Comparative Prospective Study on the Effect of Variable Platform-Switched Subcrestal Angle Correction Implants. *Int J Periodontics Restorative Dent, 40*(4), 509-517. doi:10.11607/prd.4440

Chung, S., Rungcharassaeng, K., Kan, J. Y., Roe, P., & Lozada, J. L. (2011). Immediate single tooth replacement with subepithelial connective tissue graft using platform switching implants: a case series. *J Oral Implantol, 37*(5), 559-569. doi:10.1563/aaid-joi-d-10-00110

Clauser, C., Sforza, N. M., Menini, I., Kalemaj, Z., & Buti, J. (2020). Immediate Postextraction Single-Tooth Implants and Provisional Crowns in the Esthetic Area: 2-year Results of a Cohort Prospective Multicenter Study- Patient-Centered Outcomes. *Int J Oral Maxillofac Implants, 35*(4), 833-840. doi:10.11607/jomi.7203

Clem, D. S., McClain, P. K., McGuire, M. K., Richardson, C. R., Santarelli, G. A., Schallhorn, R. A., . . . Morelli, T. (2023). Harvest graft substitute for soft tissue volume augmentation around existing implants: A randomized, controlled and blinded multicenter trial. *J Periodontol*. doi:10.1002/JPER.23-0305

Cooper, L. F., Raes, F., Reside, G. J., Garriga, J. S., Tarrida, L. G., Wiltfang, J., . . . de Bruyn, H. (2010). Comparison of radiographic and clinical outcomes following immediate provisionalization of single-tooth dental implants placed in healed alveolar ridges and extraction sockets. *Int J Oral Maxillofac Implants, 25*(6), 1222-1232.

Cooper, L. F., Reside, G., Stanford, C., Barwacz, C., Feine, J., Abi Nader, S., . . . McGuire, M. (2015). A multicenter randomized comparative trial of implants with different abutment interfaces to replace anterior maxillary single teeth. *Int J Oral Maxillofac Implants, 30*(3), 622-632. doi:10.11607/jomi.3772

Cooper, L. F., Reside, G., Stanford, C., Barwacz, C., Feine, J., Nader, S. A., . . . McGuire, M. (2019). Three-Year Prospective Randomized Comparative Assessment of Anterior Maxillary Single Implants with Different Abutment Interfaces. *INTERNATIONAL JOURNAL OF ORAL & MAXILLOFACIAL IMPLANTS, 34*(1), 150-158. doi:10.11607/jomi.6810

Cooper, L. F., Reside, G. J., Raes, F., Garriga, J. S., Tarrida, L. G., Wiltfang, J., . . . De Bruyn, H. (2014). Immediate provisionalization of dental implants placed in healed alveolar ridges and extraction sockets: a 5-year prospective evaluation. *Int J Oral Maxillofac Implants, 29*(3), 709-717. doi:10.11607/jomi.3617

Cordaro, L., Torsello, F., & Roccuzzo, M. (2009). Clinical outcome of submerged vs. non-submerged implants placed in fresh extraction sockets. *Clinical oral implants research, 20*(12), 1307-1313. doi:10.1111/j.1600-0501.2009.01724.x

Cosyn, J., De Bruyn, H., & Cleymaet, R. (2013). Soft tissue preservation and pink aesthetics around single immediate implant restorations: a 1-year prospective study. *Clin Implant Dent Relat Res, 15*(6), 847-857. doi:10.1111/j.1708-8208.2012.00448.x

Cosyn, J., & De Rouck, T. (2009). Aesthetic outcome of single-tooth implant restorations following early implant placement and guided bone regeneration: crown and soft tissue dimensions compared with contralateral teeth. *Clinical oral implants research, 20*(10), 1063-1069. doi:10.1111/j.1600-0501.2009.01746.x

Cosyn, J., Eeckhout, C., Christiaens, V., Eghbali, A., Vervaeke, S., Younes, F., & De Bruyckere, T. (2021). A multi-centre randomized controlled trial comparing connective tissue graft with collagen matrix to increase soft tissue thickness at the buccal aspect of single implants: 3-month results. *J Clin Periodontol, 48*(12), 1502-1515. doi:10.1111/jcpe.13560

Cosyn, J., Eeckhout, C., De Bruyckere, T., Eghbali, A., Vervaeke, S., Younes, F., & Christiaens, V. (2022). A multi-centre randomized controlled trial comparing connective tissue graft with collagen matrix to increase soft tissue thickness at the buccal aspect of single implants: 1-year results. *J Clin Periodontol, 49*(9), 911-921. doi:10.1111/jcpe.13691

Cosyn, J., Eghbali, A., De Bruyn, H., Collys, K., Cleymaet, R., & De Rouck, T. (2011). Immediate single-tooth implants in the anterior maxilla: 3-year results of a case series on hard and soft tissue response and aesthetics. *J Clin Periodontol, 38*(8), 746-753. doi:10.1111/j.1600-051X.2011.01748.x

Cosyn, J., Eghbali, A., Hermans, A., Vervaeke, S., De Bruyn, H., & Cleymaet, R. (2016). A 5-year prospective study on single immediate implants in the aesthetic zone. *J Clin Periodontol, 43*(8), 702-709. doi:10.1111/jcpe.12571

Cosyn, J., Pollaris, L., Van der Linden, F., & De Bruyn, H. (2015). Minimally Invasive Single Implant Treatment (M.I.S.I.T.) based on ridge preservation and contour augmentation in patients with a high aesthetic risk profile: one-year results. *J Clin Periodontol, 42*(4), 398-405. doi:10.1111/jcpe.12384

Cosyn, J., Sabzevar, M. M., & De Bruyn, H. (2012). Predictors of inter-proximal and midfacial recession following single implant treatment in the anterior maxilla: a multivariate analysis. *Journal of Clinical Periodontology, 39*(9), 895-903. doi:10.1111/j.1600-051X.2012.01921.x

Covani, U., Canullo, L., Toti, P., Alfonsi, F., & Barone, A. (2014). Tissue stability of implants placed in fresh extraction sockets: a 5-year prospective single-cohort study. *J Periodontol, 85*(9), e323-332. doi:10.1902/jop.2014.140175

Covani, U., Marconcini, S., Galassini, G., Cornelini, R., Santini, S., & Barone, A. (2007). Connective tissue graft used as a biologic barrier to cover an immediate implant. *Journal of Periodontology, 78*(8), 1644-1649. doi:10.1902/jop.2007.060461

Crespi, R., Capparè, P., Crespi, G., Gastaldi, G., Romanos, G. E., & Gherlone, E. (2019). Midfacial Tissue Assessment of the Effect of Amount of Keratinized Mucosa on Immediate Temporarization of Fresh Socket Implants: 8-Year Follow-up. *INTERNATIONAL JOURNAL OF PERIODONTICS & RESTORATIVE DENTISTRY, 39*(2), 227-232. doi:10.11607/prd.3292

Crespi, R., Capparè, P., & Gherlone, E. (2010a). A 4-year evaluation of the peri-implant parameters of immediately loaded implants placed in fresh extraction sockets. *J Periodontol, 81*(11), 1629-1634. doi:10.1902/jop.2010.100115

Crespi, R., Capparè, P., & Gherlone, E. (2010b). Fresh-socket implants in periapical infected sites in humans. *J Periodontol, 81*(3), 378-383. doi:10.1902/jop.2009.090505

Crespi, R., Capparè, P., Gherlone, E., & Romanos, G. (2012). Immediate provisionalization of dental implants placed in fresh extraction sockets using a flapless technique. *Int J Periodontics Restorative Dent, 32*(1), 29-37.

Crespi, R., Capparè, P., Polizzi, E., & Gherlone, E. (2015). Fresh-Socket Implants of Different Collar Length: Clinical Evaluation in the Aesthetic Zone. *Clinical implant dentistry and related research, 17*(5), 871-878. doi:10.1111/cid.12202

D'Elia, C., Baldini, N., Cagidiaco, E. F., Nofri, G., Goracci, C., & de Sanctis, M. (2017). Peri-implant Soft Tissue Stability After Single Implant Restorations Using Either Guided Bone Regeneration or a Connective Tissue Graft: A Randomized Clinical Trial. *INTERNATIONAL JOURNAL OF PERIODONTICS & RESTORATIVE DENTISTRY, 37*(3), 413-421. doi:10.11607/prd.2747

da Rosa, J. C., Rosa, A. C., Francischone, C. E., & Sotto-Maior, B. S. (2014). Esthetic outcomes and tissue stability of implant placement in compromised sockets following immediate dentoalveolar restoration: results of a prospective case series at 58 months follow-up. *Int J Periodontics Restorative Dent, 34*(2), 199-208. doi:10.11607/prd.1858

de Albornoz, A. C., Vignoletti, F., Ferrantino, L., Cardenas, E., de Sanctis, M., & Sanz, M. (2014). A randomized trial on the aesthetic outcomes of implant-supported restorations with zirconia or titanium abutments. *Journal of Clinical Periodontology, 41*(12), 1161-1169. doi:10.1111/jcpe.12312

De Bruyckere, T., Cabeza, R. G., Eghbali, A., Younes, F., Cleymaet, R., & Cosyn, J. (2020). A randomized controlled study comparing guided bone regeneration with connective tissue graft to reestablish buccal convexity at implant sites: A 1-year volumetric analysis. *Clin Implant Dent Relat Res, 22*(4), 468-476. doi:10.1111/cid.12934

De Bruyn, H., Raes, F., Cooper, L. F., Reside, G., Garriga, J. S., Tarrida, L. G., . . . Kern, M. (2013). Three-years clinical outcome of immediate provisionalization of single Osseospeed (TM) implants in extraction sockets and healed ridges. *Clinical oral implants research, 24*(2), 217-223. doi:10.1111/j.1600-0501.2012.02449.x

de Carvalho, B. C., de Carvalho, E. M., & Consani, R. L. (2013). Flapless single-tooth immediate implant placement. *Int J Oral Maxillofac Implants, 28*(3), 783-789. doi:10.11607/jomi.2140

de Oliveira Silva, T. S., de Freitas, A. R., de Albuquerque, R. F., Pedrazzi, V., Ribeiro, R. F., & do Nascimento, C. (2020). A 3-year longitudinal prospective study assessing microbial profile and clinical outcomes of single-unit cement-retained implant restorations: Zirconia versus titanium abutments. *Clinical implant dentistry and related research, 22*(3), 301-310. doi:10.1111/cid.12888

De Rouck, T., Collys, K., & Cosyn, J. (2008). Immediate single-tooth implants in the anterior maxilla: a 1-year case cohort study on hard and soft tissue response. *Journal of Clinical Periodontology, 35*(7), 649-657. doi:10.1111/j.1600-051X.2008.01235.x

De Rouck, T., Collys, K., Wyn, I., & Cosyn, J. (2009). Instant provisionalization of immediate single-tooth implants is essential to optimize esthetic treatment outcome. *Clinical oral implants research, 20*(6), 566-570. doi:10.1111/j.1600-0501.2008.01674.x

de Siqueira, R. A. C., Fontão, F., Sartori, I. A. M., Santos, P. G. F., Bernardes, S. R., & Tiossi, R. (2017). Effect of different implant placement depths on crestal bone levels and soft tissue behavior: a randomized clinical trial. *Clin Oral Implants Res, 28*(10), 1227-1233. doi:10.1111/clr.12946

de Siqueira, R. A. C., Savaget Gonçalves Junior, R., Dos Santos, P. G. F., de Mattias Sartori, I. A., Wang, H. L., & Fontão, F. (2020). Effect of different implant placement depths on crestal bone levels and soft tissue behavior: A 5-year randomized clinical trial. *Clin Oral Implants Res, 31*(3), 282-293. doi:10.1111/clr.13569

Degidi, M., Nardi, D., Daprile, G., & Piattelli, A. (2014). Nonremoval of immediate abutments in cases involving subcrestally placed postextractive tapered single implants: a randomized controlled clinical study. *Clin Implant Dent Relat Res, 16*(6), 794-805. doi:10.1111/cid.12051

Degidi, M., Novaes, A. B., Nardi, D., & Piattelli, A. (2008). Outcome analysis of immediately placed, immediately restored implants in the esthetic area: The clinical relevance of different interimplant distances. *Journal of Periodontology, 79*(6), 1056-1061. doi:10.1902/jop.2008.070534

den Hartog, L., Raghoebar, G. M., Stellingsma, K., Vissink, A., & Meijer, H. J. (2011). Immediate non-occlusal loading of single implants in the aesthetic zone: a randomized clinical trial. *J Clin Periodontol, 38*(2), 186-194. doi:10.1111/j.1600-051X.2010.01650.x

Doornewaard, R., Bruyn, H., Matthys, C., Bronkhorst, E., Vandeweghe, S., & Vervaeke, S. (2020). The Long-Term Effect of Adapting the Vertical Position of Implants on Peri-Implant Health: A 5-Year Intra-Subject Comparison in the Edentulous Mandible Including Oral Health-Related Quality of Life. *J Clin Med, 9*(10). doi:10.3390/jcm9103320

Doornewaard, R., Glibert, M., Matthys, C., Vervaeke, S., Bronkhorst, E., & de Bruyn, H. (2019). Improvement of Quality of Life with Implant-Supported Mandibular Overdentures and the Effect of Implant Type and Surgical Procedure on Bone and Soft Tissue Stability: A Three-Year Prospective Split-Mouth Trial. *J Clin Med, 8*(6). doi:10.3390/jcm8060773

Duque, A. D., Aristizabal, A. G., Londono, S., Castro, L., & Alvarez, L. G. (2016). Prevalence of peri-implant disease on platform switching implants: a cross-sectional pilot study. *BRAZILIAN ORAL RESEARCH, 30*(1). doi:10.1590/1807-3107BOR-2016.vol30.0005

Eeckhout, C., Bouckaert, E., Verleyen, D., De Bruyckere, T., & Cosyn, J. (2020). A 3-Year Prospective Study on a Porcine-Derived Acellular Collagen Matrix to Re-Establish Convexity at the Buccal Aspect of Single Implants in the Molar Area: A Volumetric Analysis. *J Clin Med, 9*(5). doi:10.3390/jcm9051568

Eeckhout, C., Vuylsteke, F., Seyssens, L., Christiaens, V., De Bruyckere, T., Eghbali, A., . . . Cosyn, J. (2023). A Multi-Centre Randomized Controlled Trial Comparing Connective Tissue Graft with Collagen Matrix to Increase Buccal Soft Tissue Thickness: A Cone-Beam CT Analysis. *J Clin Med, 12*(8). doi:10.3390/jcm12082977

Eghbali, A., De Bruyn, H., Cosyn, J., Kerckaert, I., & Van Hoof, T. (2016). Ultrasonic Assessment of Mucosal Thickness around Implants: Validity, Reproducibility, and Stability of Connective Tissue Grafts at the Buccal Aspect. *Clin Implant Dent Relat Res, 18*(1), 51-61. doi:10.1111/cid.12245

Eghbali, A., Seyssens, L., De Bruyckere, T., Younes, F., Cleymaet, R., & Cosyn, J. (2018). A 5-year prospective study on the clinical and aesthetic outcomes of alveolar ridge preservation and connective tissue graft at the buccal aspect of single implants. *Journal of Clinical Periodontology, 45*(12), 1475-1484. doi:10.1111/jcpe.13018

Ekfeldt, A., Eriksson, A., & Johansson, L. A. (2003). Peri-implant mucosal level in patients treated with implant-supported fixed prostheses: A 1-year follow-up study. *INTERNATIONAL JOURNAL OF PROSTHODONTICS, 16*(5), 529-532.

Eladl, N. M., Sholkamy, H. M., & Emara, A. S. (2021). 1-Year Post-loading of Short and Ultrashort Implants in Posterior Mandibular and Maxillary Regions. *Open Access Macedonian Journal of Medical Sciences, 9*(D), 264-269. doi:10.3889/OAMJMS.2021.7237

Elaskary, A., Abdelrahman, H., Elfahl, B., Elsabagh, H., El-Kimary, G., & Ghallab, N. A. (2023). Immediate Implant Placement in Intact Fresh Extraction Sockets Using Vestibular Socket Therapy Versus Partial Extraction Therapy in the Esthetic Zone: A Randomized Clinical Trial. *Int J Oral Maxillofac Implants, 38*(3), 468-478. doi:10.11607/jomi.9973

Esposito, M., Bressan, E., Grusovin, M. G., D'Avenia, F., Neumann, K., Sbricoli, L., & Luongo, G. (2017). Do repeated changes of abutments have any influence on the stability of peri-implant tissues? One-year post-loading results from a multicentre randomised controlled trial. *Eur J Oral Implantol, 10*(1), 57-72.

Esposito, M., Gonzalez-Garcia, A., Penarrocha Diago, M., Fernandez Encinas, R., Trullenque-Eriksson, A., Xhanari, E., & Penarrocha Oltra, D. (2018). Natural or palatal positioning of immediate post-extractive implants in the aesthetic zone? 1-year results of a multicentre randomised controlled trial. *Eur J Oral Implantol, 11*(2), 189-200.

Evans, C. D. J., & Chen, S. T. (2008). Esthetic outcomes of immediate implant placements. *Clinical oral implants research, 19*(1), 73-80. doi:10.1111/j.1600-0501.2007.01413.x

Farrag, K. M., & Khamis, M. M. (2023). Effect of anodized titanium abutment collars on peri-implant soft tissue: A split-mouth clinical study. *JOURNAL OF PROSTHETIC DENTISTRY, 130*(1), 59-67. doi:10.1016/j.prosdent.2021.09.019

Farronato, D., Manfredini, M., Farronato, M., Pasini, P. M., Orsina, A. A., & Lops, D. (2021). Behavior of soft tissue around platform-switched implants and non-platform-switched implants: A comparative three-year clinical study. *Journal of Clinical Medicine, 10*(13). doi:10.3390/jcm10132955

Farronato, D., Pasini, P. M., Orsina, A. A., Manfredini, M., Azzi, L., & Farronato, M. (2020). Correlation between Buccal Bone Thickness at Implant Placement in Healed Sites and Buccal Soft Tissue Maturation Pattern: A Prospective Three-Year Study. *Materials (Basel), 13*(3). doi:10.3390/ma13030511

Fenner, N., Hammerle, C. H. F., Sailer, I., & Jung, R. E. (2016). Long-term clinical, technical, and esthetic outcomes of all-ceramic vs. titanium abutments on implant supporting single-tooth reconstructions after at least 5 years. *Clinical oral implants research, 27*(6), 716-723. doi:10.1111/clr.12654

Fernandes, D., Marques, T., Borges, T., & Montero, J. (2023). Volumetric analysis on the use of customized healing abutments with or without connective tissue graft at flapless maxillary immediate implant placement: A randomized clinical trial. *Clin Oral Implants Res, 34*(9), 934-946. doi:10.1111/clr.14119

Ferrari, M., Cagidiaco, M. C., Garcia-Godoy, F., Goracci, C., & Cairo, F. (2015). Effect of different prosthetic abutments on peri-implant soft tissue. A randomized controlled clinical trial. *AMERICAN JOURNAL OF DENTISTRY, 28*(2), 85-89.

Finelle, G., Popelut, A., Knafo, B., & Martin, I. S. (2021). Sealing Socket Abutments (SSAs) in Molar Immediate Implants with a Digitalized CAD/CAM Protocol: Soft Tissue Contour Changes and Radiographic Outcomes After 2 Years. *Int J Periodontics Restorative Dent, 41*(2), 235-244. doi:10.11607/prd.4579

Frisch, E., & Ratka-Kruger, P. (2020). A new technique for peri-implant recession treatment: Partially epithelialized connective tissue grafts. Description of the technique and preliminary results of a case series. *Clin Implant Dent Relat Res, 22*(3), 403-408. doi:10.1111/cid.12897

Frizzera, F., de Freitas, R. M., Munoz-Chavez, O. F., Cabral, G., Shibli, J. A., & Marcantonio, E. M. (2019). Impact of Soft Tissue Grafts to Reduce Peri-implant Alterations After Immediate Implant Placement and Provisionalization in Compromised Sockets. *INTERNATIONAL JOURNAL OF PERIODONTICS & RESTORATIVE DENTISTRY, 39*(3), 381-390. doi:10.11607/prd.3224

Fürhauser, R., Fürhauser, L., Fürhauser, N., Pohl, V., Pommer, B., & Haas, R. (2022). Bucco-palatal implant position and its impact on soft tissue level in the maxillary esthetic zone. *Clin Oral Implants Res, 33*(11), 1125-1134. doi:10.1111/clr.13995

Fürhauser, R., Mailath-Pokorny, G., Haas, R., Busenlechner, D., Watzek, G., & Pommer, B. (2017). Immediate Restoration of Immediate Implants in the Esthetic Zone of the Maxilla Via the Copy-Abutment Technique: 5-Year Follow-Up of Pink Esthetic Scores. *Clin Implant Dent Relat Res, 19*(1), 28-37. doi:10.1111/cid.12423

Galindo-Moreno, P., Nilsson, P., King, P., Worsaae, N., Schramm, A., Padial-Molina, M., & Maiorana, C. (2017). Clinical and radiographic evaluation of early loaded narrow-diameter implants: 5-year follow-up of a multicenter prospective clinical study. *Clin Oral Implants Res, 28*(12), 1584-1591. doi:10.1111/clr.13029

Gallucci, G. O., Grutter, L., Nedir, R., Bischof, M., & Belser, U. C. (2011). Esthetic outcomes with porcelain-fused-to-ceramic and all-ceramic single-implant crowns: a randomized clinical trial. *Clinical oral implants research, 22*(1), 62-69. doi:10.1111/j.1600-0501.2010.01997.x

Garaicoa-Pazmino, C., Mendonca, G., Ou, A., Chan, H. L., Mailoa, J., Suarez-Lopez Del Amo, F., & Wang, H. L. (2021). Impact of mucosal phenotype on marginal bone levels around tissue level implants: A prospective controlled trial. *J Periodontol, 92*(6), 771-783. doi:10.1002/JPER.20-0458

Ghallab, N. A., Elaskary, A., Elsabagh, H., Toukhy, A. E., Abdelrahman, H., & El-Kimary, G. (2023). A novel atraumatic extraction technique using vestibular socket therapy for immediate implant placement: a randomized controlled clinical trial. *Oral Maxillofac Surg, 27*(3), 497-505. doi:10.1007/s10006-022-01089-4

Ghanaati, S., Lorenz, J., Obreja, K., Choukroun, J., Landes, C., & Sader, R. A. (2014). Nanocrystalline Hydroxyapatite-Based Material Already Contributes to Implant Stability After 3 Months: A Clinical and Radio logic 3-Year Follow-up Investigation. *JOURNAL OF ORAL IMPLANTOLOGY, 40*(1), 103-109. doi:10.1563/AAID-JOI-D-13-00232

Ghazal, S. S., Huynh-Ba, G., Aghaloo, T., Dibart, S., Froum, S., O’Neal, R., & Cochran, D. (2019). Randomized controlled multicenter clinical study evaluating crestal bone level change of narrow-diameter versus standard-diameter Ti-Zr implants for single tooth replacement in anterior and premolar region. *International Journal of Oral and Maxillofacial Implants, 34*(3), 708-718. doi:10.11607/jomi.6927

Girbes-Ballester, P., Vina-Almunia, J., Penarrocha-Oltra, D., & Penarrocha-Diago, M. (2016). Soft Tissue Response in Posterior Teeth Adjacent to Interdental Single Implants: A Controlled Randomized Clinical Trial Comparing Intrasulcular vs Trapezoidal Incision. *INTERNATIONAL JOURNAL OF ORAL & MAXILLOFACIAL IMPLANTS, 31*(3), 631-641. doi:10.11607/jomi.4178

Girlanda, F. F., Feng, H. S., Correa, M. G., Casati, M. Z., Pimentel, S. P., Ribeiro, F. V., & Cirano, F. R. (2019). Deproteinized bovine bone derived with collagen improves soft and bone tissue outcomes in flapless immediate implant approach and immediate provisionalization: a randomized clinical trial. *Clin Oral Investig, 23*(10), 3885-3893. doi:10.1007/s00784-019-02819-x

Givens, E., Jr., Bencharit, S., Byrd, W. C., Phillips, C., Hosseini, B., & Tyndall, D. (2015). Immediate Placement and Provisionalization of Implants Into Sites With Periradicular Infection With and Without Antibiotics: An Exploratory Study. *J Oral Implantol, 41*(3), 299-305. doi:10.1563/AAID-JOI-D-13-00002

Gómez-Meda, R., Rizo-Gorrita, M., Serrera-Figallo, M. A., Esquivel, J., Herraez-Galindo, C., & Torres-Lagares, D. (2022). Dimensional Changes in the Alveolus after a Combination of Immediate Postextraction Implant and Connective Grafting and/or Socket Shield Technique. *Int J Environ Res Public Health, 19*(5). doi:10.3390/ijerph19052795

Grandi, T., Guazzi, P., Samarani, R., & Grandi, G. (2013). Immediate provisionalisation of single post-extractive implants versus implants placed in healed sites in the anterior maxilla: 1-year results from a multicentre controlled cohort study. *Eur J Oral Implantol, 6*(3), 285-295.

Grassi, F. R., Capogreco, M., Consonni, D., Bilardi, G., Buti, J., & Kalemaj, Z. (2015). Immediate Occlusal Loading of One-Piece Zirconia Implants: Five-Year Radiographic and Clinical Evaluation. *INTERNATIONAL JOURNAL OF ORAL & MAXILLOFACIAL IMPLANTS, 30*(3), 671-680. doi:10.11607/jomi.3831

Groenendijk, E., Bronkhorst, E. M., & Meijer, G. J. (2021). Does the pre-operative buccal soft tissue level at teeth or gingival phenotype dictate the aesthetic outcome after flapless immediate implant placement and provisionalization? Analysis of a prospective clinical case series. *Int J Implant Dent, 7*(1), 84. doi:10.1186/s40729-021-00366-3

Groenendijk, E., Staas, T. A., Bronkhorst, E. M., Raghoebar, G. M., & Meijer, G. J. (2023). Factors Associated with Esthetic Outcomes of Flapless Immediate Placed and Loaded Implants in the Maxillary Incisor Region-Three-Year Results of a Prospective Case Series. *Journal of Clinical Medicine, 12*(7). doi:10.3390/jcm12072625

Guarnieri, R., Ceccarelli, R., Ricci, J. L., & Testori, T. (2018). Implants With and Without Laser-Microtextured Collar: A 10-Year Clinical and Radiographic Comparative Evaluation. *Implant Dentistry, 27*(1), 81-88. doi:10.1097/ID.0000000000000718

Guarnieri, R., Ceccherini, A., & Grande, M. (2015). Single-tooth replacement in the anterior maxilla by means of immediate implantation and early loading: clinical and aesthetic results at 5 years. *Clin Implant Dent Relat Res, 17*(2), 314-326. doi:10.1111/cid.12111

Guarnieri, R., Di Nardo, D., Di Giorgio, G., Miccoli, G., & Testarelli, L. (2019). Clinical and radiographics results at 3 years of RCT with split-mouth design of submerged vs. nonsubmerged single laser-microgrooved implants in posterior areas. *International journal of implant dentistry, 5*(1). doi:10.1186/s40729-019-0196-0

Guarnieri, R., Di Nardo, D., Gaimari, G., Miccoli, G., & Testarelli, L. (2019). Short vs. Standard Laser-Microgrooved Implants Supporting Single and Splinted Crowns: A Prospective Study with 3 Years Follow-Up. *JOURNAL OF PROSTHODONTICS-IMPLANT ESTHETIC AND RECONSTRUCTIVE DENTISTRY, 28*(2), E771-E779. doi:10.1111/jopr.12959

Guarnieri, R., Reda, R., Di Nardo, D., Miccoli, G., Zanza, A., & Testarelli, L. (2022). Clinical, radiographic, and biochemical evaluation of two-piece versus one-piece single implants with a laser-microgrooved collar surface after 5 years of functional loading. *Clinical implant dentistry and related research, 24*(5), 676-682. doi:10.1111/cid.13118

Hall, J. A., Payne, A. G., Purton, D. G., Torr, B., Duncan, W. J., & De Silva, R. K. (2007). Immediately restored, single-tapered implants in the anterior maxilla: prosthodontic and aesthetic outcomes after 1 year. *Clin Implant Dent Relat Res, 9*(1), 34-45. doi:10.1111/j.1708-8208.2007.00029.x

Hammerle, C. H. F., Jepsen, K., Sailer, I., Strasding, M., Zeltner, M., Cordaro, L., . . . Sanz, M. (2023). Efficacy of a collagen matrix for soft tissue augmentation after implant placement compared to connective tissue grafts: A multicenter, noninferiority, randomized controlled trial. *Clin Oral Implants Res, 34*(9), 999-1013. doi:10.1111/clr.14127

Hassan, K. S. (2009). Autogenous bone graft combined with polylactic polyglycolic acid polymer for treatment of dehiscence around immediate dental implants. *Oral Surg Oral Med Oral Pathol Oral Radiol Endod, 108*(5), e19-25. doi:10.1016/j.tripleo.2009.07.023

Hattingh, A., De Bruyn, H., Van Weehaeghe, M., Hommez, G., & Vandeweghe, S. (2020). Contour Changes Following Immediate Placement of Ultra-Wide Implants in Molar Extraction Sockets without Bone Grafting. *J Clin Med, 9*(8). doi:10.3390/jcm9082504

Hernández, A. E., de Azevedo Kinalski, M., de Andrade Leão, O. A., Bergoli, C. D., Faot, F., & dos Santos, M. B. F. (2022). Assessment of Surgical and Radiographic Parameters for Abutment Height Selection: A Prospective Study with 1-Year Follow-up. *International Journal of Oral and Maxillofacial Implants, 37*(5), 1037-1043. doi:10.11607/jomi.9446

Hinze, M., Janousch, R., Goldhahn, S., & Schlee, M. (2018). Volumetric alterations around single-tooth implants using the socket-shield technique: preliminary results of a prospective case series. *INTERNATIONAL JOURNAL OF ESTHETIC DENTISTRY, 13*(2), 146-170.

Hof, M., Pommer, B., Ambros, H., Jesch, P., Vogl, S., & Zechner, W. (2015). Does Timing of Implant Placement Affect Implant Therapy Outcome in the Aesthetic Zone? A Clinical, Radiological, Aesthetic, and Patient-Based Evaluation. *Clinical implant dentistry and related research, 17*(6), 1188-1199. doi:10.1111/cid.12212

Hof, M., Tepper, G., Koller, B., Krainhofner, M., Watzek, G., & Pommer, B. (2014). Esthetic evaluation of single-tooth implants in the anterior mandible. *Clinical oral implants research, 25*(9), 1022-1026. doi:10.1111/clr.12210

Hollander, J., Lorenz, J., Stubinger, S., Holscher, W., Heidemann, D., Ghanaati, S., & Sader, R. (2016). Zirconia Dental Implants: Investigation of Clinical Parameters, Patient Satisfaction, and Microbial Contamination. *INTERNATIONAL JOURNAL OF ORAL & MAXILLOFACIAL IMPLANTS, 31*(4), 855-864. doi:10.11607/jomi.4511

Hosseini, B., Byrd, W. C., Preisser, J. S., Khan, A., Duggan, D., & Bencharit, S. (2015). Effects of Antibiotics on Bone and Soft-Tissue Healing Following Immediate Single-Tooth Implant Placement Into Sites With Apical Pathology. *J Oral Implantol, 41*(5), e202-211. doi:10.1563/aaid-joi-D-14-00075

Hosseini, M., Worsaae, N., & Gotfredsen, K. (2020). Tissue changes at implant sites in the anterior maxilla with and without connective tissue grafting: A five-year prospective study. *Clin Oral Implants Res, 31*(1), 18-28. doi:10.1111/clr.13540

Hu, C., Lin, W., Gong, T., Zuo, Y., Qu, Y., & Man, Y. (2018). Early Healing of Immediate Implants Connected With Two Types of Healing Abutments: A Prospective Cohort Study. *Implant Dent, 27*(6), 646-652. doi:10.1097/id.0000000000000809

Huang, H. Y., Ogata, Y., Hanley, J., Finkelman, M., & Hur, Y. (2016). Crestal bone resorption in augmented bone using mineralized freeze-dried bone allograft or pristine bone during submerged implant healing: a prospective study in humans. *Clin Oral Implants Res, 27*(2), e25-30. doi:10.1111/clr.12512

Humm, V. L., Sailer, I., Thoma, D. S., Hammerle, C. H. F., Jung, R. E., & Zembic, A. (2023). 13-year follow-up of a randomized controlled study on zirconia and titanium abutments. *Clinical oral implants research, 34*(9), 911-919. doi:10.1111/clr.14117

Hutton, C. G., Johnson, G. K., Barwacz, C. A., Allareddy, V., & Avila-Ortiz, G. (2018). Comparison of two different surgical approaches to increase peri-implant mucosal thickness: A randomized controlled clinical trial. *Journal of Periodontology, 89*(7), 807-814. doi:10.1002/JPER.17-0597

Huynh-Ba, G., Hoders, A. B., Meister, D. J., Prihoda, T. J., Mills, M. P., Mealey, B. L., & Cochran, D. L. (2019). Esthetic, clinical, and radiographic outcomes of two surgical approaches for single implant in the esthetic area: 1-year results of a randomized controlled trial with parallel design. *Clin Oral Implants Res, 30*(8), 745-759. doi:10.1111/clr.13458

Huynh-Ba, G., Meister, D. J., Hoders, A. B., Mealey, B. L., Mills, M. P., Oates, T. W., . . . McMahan, C. A. (2016). Esthetic, clinical and patient-centered outcomes of immediately placed implants (Type 1) and early placed implants (Type 2): preliminary 3-month results of an ongoing randomized controlled clinical trial. *Clin Oral Implants Res, 27*(2), 241-252. doi:10.1111/clr.12577

Iglhaut, G., Salomon, S., Fretwurst, T., Thomas, P., Endres, J., Kessler, S., & Summer, B. (2021). Cross-sectional evaluation of clinical and immunological parameters at partially microgrooved vs machined abutments in humans. *International journal of implant dentistry, 7*(1). doi:10.1186/s40729-021-00329-8

Iorio-Siciliano, V., Marenzi, G., Blasi, A., Mignogna, J., Cafiero, C., Wang, H.-L., & Sammartino, G. (2016). Influence of Platform-Switched, Laser-Microtextured Implant on Marginal Bone Level: A 24-Month Case Series Study. *The International journal of oral & maxillofacial implants, 31*(1), 162-166. doi:<https://dx.doi.org/10.11607/jomi.4130>

Jacobs, B. P., Zadeh, H. H., De Kok, I., & Cooper, L. (2020). A Randomized Controlled Trial Evaluating Grafting the Facial Gap at Immediately Placed Implants. *Int J Periodontics Restorative Dent, 40*(3), 383-392. doi:10.11607/prd.3774

Janyaphadungpong, R., Serichetaphongse, P., & Pimkhaokham, A. (2019). A Clinical Resonance Frequency Analysis of Implants Placed at Dehiscence-type Defects with Simultaneous Guided Bone Regeneration During Early Healing. *Int J Oral Maxillofac Implants, 34*(3), 772–777. doi:10.11607/jomi.6834

Jeffcoat, M. K., McGlumphy, E. A., Reddy, M. S., Geurs, N. C., & Proskin, H. M. (2003). A comparison of hydroxyapatite (HA)-coated threaded, HA-coated cylindric, and titanium threaded endosseous dental implants. *INTERNATIONAL JOURNAL OF ORAL & MAXILLOFACIAL IMPLANTS, 18*(3), 406-410.

Jemt, T., Ahlberg, G., Henriksson, K., & Bondevik, O. (2006). Changes of anterior clinical crown height in patients provided with single-implant restorations after more than 15 years of follow-up. *INTERNATIONAL JOURNAL OF PROSTHODONTICS, 19*(5), 455-461.

Jeng, M. D., & Chiang, C. P. (2020). Autogenous bone grafts and titanium mesh-guided alveolar ridge augmentation for dental implantation. *J Dent Sci, 15*(3), 243-248. doi:10.1016/j.jds.2020.06.012

Jensen, O. T., Cullum, D. R., & Baer, D. (2009). Marginal Bone Stability Using 3 Different Flap Approaches for Alveolar Split Expansion for Dental Implants-A 1-Year Clinical Study. *Journal of Oral and Maxillofacial Surgery, 67*(9), 1921-1930. doi:10.1016/j.joms.2009.04.017

Jeong, S. M., Choi, B. H., Kim, J., Xuan, F., Lee, D. H., Mo, D. Y., & Lee, C. U. (2011). A 1-year prospective clinical study of soft tissue conditions and marginal bone changes around dental implants after flapless implant surgery. *Oral Surg Oral Med Oral Pathol Oral Radiol Endod, 111*(1), 41-46. doi:10.1016/j.tripleo.2010.03.037

Jiang, X., Di, P., Ren, S., Zhang, Y., & Lin, Y. (2020). Hard and soft tissue alterations during the healing stage of immediate implant placement and provisionalization with or without connective tissue graft: A randomized clinical trial. *J Clin Periodontol, 47*(8), 1006-1015. doi:10.1111/jcpe.13331

Jonker, B. P., Gil, A., Naenni, N., Jung, R. E., Wolvius, E. B., & Pijpe, J. (2021). Soft tissue contour and radiographic evaluation of ridge preservation in early implant placement: A randomized controlled clinical trial. *Clin Oral Implants Res, 32*(1), 123-133. doi:10.1111/clr.13686

Jonker, B. P., Strauss, F. J., Naenni, N., Jung, R. E., Wolvius, E. B., & Pijpe, J. (2021). Early implant placement with or without alveolar ridge preservation in single tooth gaps renders similar esthetic, clinical and patient-reported outcome measures: One-year results of a randomized clinical trial. *Clin Oral Implants Res, 32*(9), 1041-1051. doi:10.1111/clr.13796

Jovanovic, S. A., Spiekermann, H., & Richter, E. J. (1992). Bone regeneration around titanium dental implants in dehisced defect sites: a clinical study. *Int J Oral Maxillofac Implants, 7*(2), 233-245.

Jung, R. E., Grohmann, P., Sailer, I., Steinhart, Y. N., Feher, A., Hammerle, C., . . . Kohal, R. (2016). Evaluation of a one-piece ceramic implant used for single-tooth replacement and three-unit fixed partial dentures: a prospective cohort clinical trial. *Clinical oral implants research, 27*(7), 751-761. doi:10.1111/clr.12670

Jung, R. E., Hälg, G. A., Thoma, D. S., & Hämmerle, C. H. F. (2009). A randomized, controlled clinical trial to evaluate a new membrane for guided bone regeneration around dental implants. *Clinical oral implants research, 20*(2), 162-168. doi:10.1111/j.1600-0501.2008.01634.x

Jung, R. E., Herzog, M., Wolleb, K., Ramel, C. F., Thoma, D. S., & Hämmerle, C. H. (2017). A randomized controlled clinical trial comparing small buccal dehiscence defects around dental implants treated with guided bone regeneration or left for spontaneous healing. *Clin Oral Implants Res, 28*(3), 348-354. doi:10.1111/clr.12806

Kan, J. Y., Rungcharassaeng, K., Lozada, J. L., & Zimmerman, G. (2011). Facial gingival tissue stability following immediate placement and provisionalization of maxillary anterior single implants: a 2- to 8-year follow-up. *Int J Oral Maxillofac Implants, 26*(1), 179-187.

Kan, J. Y. K., Rungcharassaeng, K., & Lozada, J. (2003). Immediate placement and provisionalization of maxillary anterior single implants: 1-year prospective study. *INTERNATIONAL JOURNAL OF ORAL & MAXILLOFACIAL IMPLANTS, 18*(1), 31-39.

Kan, J. Y. K., Rungcharassaeng, K., Sclar, A., & Lozada, J. L. (2007). Effects of the Facial Osseous Defect Morphology on Gingival Dynamics After Immediate Tooth Replacement and Guided Bone Regeneration: 1-Year Results. *Journal of Oral and Maxillofacial Surgery, 65*(7 SUPPL.), 13-19. doi:10.1016/j.joms.2007.04.006

Karkar, K. T., Metwally, S. A., & Mohsen, K. M. A. (2023). Anterior Aesthetic Zone Reconstruction with Allogenic Bone Shell and Autogenous Bone Chips - An Evaluative Study. *Ann Maxillofac Surg, 13*(1), 13-18. doi:10.4103/ams.ams_34_23

Karoussis, I. K., Muller, S., Salvi, G. E., Heitz-Mayfield, L. J. A., Bragger, U., & Lang, N. P. (2004). Association between periodontal and peri-implant conditions: a 10-year prospective study. *Clinical oral implants research, 15*(1), 1-7. doi:10.1111/j.1600-0501.2004.00982.x

Khzam, N., Mattheos, N., Roberts, D., Bruce, W. L., & Ivanovski, S. (2014). Immediate placement and restoration of dental implants in the esthetic region: clinical case series. *J Esthet Restor Dent, 26*(5), 332-344. doi:10.1111/jerd.12083

Kim, B. S., Kim, Y. K., Yun, P. Y., Yi, Y. J., Lee, H. J., Kim, S. G., & Son, J. S. (2009). Evaluation of peri-implant tissue response according to the presence of keratinized mucosa. *ORAL SURGERY ORAL MEDICINE ORAL PATHOLOGY ORAL RADIOLOGY AND ENDODONTOLOGY, 107*(3), E24-E28. doi:10.1016/j.tripleo.2008.12.010

Kim, Y. K., & Yun, P. Y. (2014). Risk Factors for Wound Dehiscence after Guided Bone Regeneration in Dental Implant Surgery. *Maxillofac Plast Reconstr Surg, 36*(3), 116-123. doi:10.14402/jkamprs.2014.36.3.116

King, P., Maiorana, C., Luthardt, R. G., Sondell, K., Øland, J., Galindo-Moreno, P., & Nilsson, P. (2016). Clinical and Radiographic Evaluation of a Small-Diameter Dental Implant Used for the Restoration of Patients with Permanent Tooth Agenesis (Hypodontia) in the Maxillary Lateral Incisor and Mandibular Incisor Regions: A 36-Month Follow-Up. *Int J Prosthodont, 29*(2), 147-153. doi:10.11607/ijp.4444

Kniha, K., Bock, A., Peters, F., Heitzer, M., Modabber, A., Kniha, H., . . . Mohlhenrich, S. C. (2020). Aesthetic aspects of adjacent maxillary single-crown implants-influence of zirconia and titanium as implant materials. *International Journal of Oral and Maxillofacial Surgery, 49*(11), 1489-1496. doi:10.1016/j.ijom.2020.04.003

Kniha, K., Schlegel, K. A., Kniha, H., Modabber, A., Neukam, F., & Kniha, K. (2019). Papilla-Crown Height Dimensions around Zirconium Dioxide Implants in the Esthetic Area: A 3-Year Follow-Up Study. *JOURNAL OF PROSTHODONTICS-IMPLANT ESTHETIC AND RECONSTRUCTIVE DENTISTRY, 28*(2), E694-E698. doi:10.1111/jopr.12766

Kobayashi, T., Nakano, T., Ono, S., Matsumura, A., Yamada, S., & Yatani, H. (2020). Quantitative evaluation of connective tissue grafts on peri-implant tissue morphology in the esthetic zone: A 1-year prospective clinical study. *Clinical implant dentistry and related research, 22*(3), 311-318. doi:10.1111/cid.12898

Koh, R. U., Oh, T. J., Rudek, I., Neiva, G. F., Misch, C. E., Rothman, E. D., & Wang, H. L. (2011). Hard and soft tissue changes after crestal and subcrestal immediate implant placement. *J Periodontol, 82*(8), 1112-1120. doi:10.1902/jop.2011.100541

Kolerman, R., Tal, H., Guirado, J. L. C., Barnea, E., Chaushu, L., Abu Wasel, M., & Nissan, J. (2018). Aesthetics and Survival of Immediately Restored Implants in Partially Edentulous Anterior Maxillary Patients. *APPLIED SCIENCES-BASEL, 8*(3). doi:10.3390/app8030377

Konstantinidis, I. K., Siormpas, K. D., Kontsiotou-Siormpa, E., Mitsias, M. E., & Kotsakis, G. A. (2016). Long-Term Esthetic Evaluation of the Roll Flap Technique in the Implant Rehabilitation of Patients with Agenesis of Maxillary Lateral Incisors: 10-Year Follow-Up. *INTERNATIONAL JOURNAL OF ORAL & MAXILLOFACIAL IMPLANTS, 31*(4), 820-826. doi:10.11607/jomi.4494

Kuchler, U., Chappuis, V., Gruber, R., Lang, N. P., & Salvi, G. E. (2016). Immediate implant placement with simultaneous guided bone regeneration in the esthetic zone: 10-year clinical and radiographic outcomes. *Clin Oral Implants Res, 27*(2), 253-257. doi:10.1111/clr.12586

Kungsadalpipob, K., Supanimitkul, K., Manopattanasoontorn, S., Sophon, N., Tangsathian, T., & Arunyanak, S. P. (2020). The lack of keratinized mucosa is associated with poor peri-implant tissue health: a cross-sectional study. *International journal of implant dentistry, 6*(1). doi:10.1186/s40729-020-00227-5

Lago, L., da Silva, L., Gude, F., & Rilo, B. (2017). Bone and Soft Tissue Response in Bone-Level Implants Restored with Platform Switching: A 5-Year Clinical Prospective Study. *INTERNATIONAL JOURNAL OF ORAL & MAXILLOFACIAL IMPLANTS, 32*(4), 919-926. doi:10.11607/jomi.5859

Lambert, P. M., Morris, H. F., & Ochi, S. (2000). The influence of smoking on 3-year clinical success of osseointegrated dental implants. *Ann Periodontol, 5*(1), 79-89. doi:10.1902/annals.2000.5.1.79

Lazzari, T. R., Jardini, M. A. N., dos Santos, N. C., Neves, F. L. D., Lima, V. C. D., de Melo, A. B., & Santamaria, M. P. (2022). Single implant placement in the maxillary aesthetic area with or without connective tissue grafting: A 1-year follow-up randomised clinical trial. *INTERNATIONAL JOURNAL OF ORAL IMPLANTOLOGY, 15*(1), 57-67.

Lee, C. T., Sanz-Miralles, E., Zhu, L., Glick, J., Heath, A., & Stoupel, J. (2020). Predicting bone and soft tissue alterations of immediate implant sites in the esthetic zone using clinical parameters. *Clin Implant Dent Relat Res, 22*(3), 325-332. doi:10.1111/cid.12910

Lee, C. T., Tran, D., Tsukiboshi, Y., Min, S., Kim, S. K., Ayilavarapu, S., & Weltman, R. (2023). Clinical efficacy of soft-tissue augmentation on tissue preservation at immediate implant sites: A randomized controlled trial. *J Clin Periodontol, 50*(7), 1010-1020. doi:10.1111/jcpe.13816

Lee, D.-W., Lee, J.-W., Lim, H.-C., Kang, K. L., Choi, S.-H., & Yu, J.-A. (2015). Simultaneous Block Bone Grafting Using "L-Shaped Notch" Preparation in Mandible: Case Series and 1-Year Follow-up. *Implant Dentistry, 24*(5), 625-630. doi:<https://dx.doi.org/10.1097/ID.0000000000000311>

Lee, D. H., Choi, B. H., Jeong, S. M., Xuan, F., & Kim, H. R. (2011). Effects of Flapless Implant Surgery on Soft Tissue Profiles: A Prospective Clinical Study. *Clinical implant dentistry and related research, 13*(4), 324-329. doi:10.1111/j.1708-8208.2009.00217.x

Lee, H., Fehmer, V., Hicklin, S., Noh, G., Hong, S. J., & Sailer, I. (2020). Three-Dimensional Evaluation of Peri-implant Soft Tissue When Tapered Implants Are Placed: Pilot Study with Implants Placed Immediately or Early Following Tooth Extraction. *INTERNATIONAL JOURNAL OF ORAL & MAXILLOFACIAL IMPLANTS, 35*(5), 1036-1043. doi:10.11607/jomi.7879

Lee, Y. M., Kim, D. Y., Kim, J. Y., Kim, S. H., Koo, K. T., Kim, T. I., & Seol, Y. J. (2012). Peri-implant soft tissue level secondary to a connective tissue graft in conjunction with immediate implant placement: a 2-year follow-up report of 11 consecutive cases. *Int J Periodontics Restorative Dent, 32*(2), 213-222.

Lertwongpaisan, T., Amornsettachai, P., Panyayong, W., & Suphangul, S. (2023). Soft tissue dimensional change using customized titanium healing abutment in immediate implant placement in posterior teeth. *BMC oral health, 23*(1), 384. doi:10.1186/s12903-023-03060-5

Levin, L., & Clark-Perry, D. (2022). Use of a Novel In Situ Hardening Biphasic Alloplastic Bone Grafting Material for Guided Bone Regeneration Around Dental Implants: A Prospective Case Series. *Clin Adv Periodontics, 12*(1), 12-16. doi:10.1002/cap.10141

Li, S., Gao, M., Zhou, M., & Zhu, Y. (2021). Bone augmentation with autologous tooth shell in the esthetic zone for dental implant restoration: a pilot study. *Int J Implant Dent, 7*(1), 108. doi:10.1186/s40729-021-00389-w

Liaje, A., Ozkan, Y. K., Ozkan, Y., & Vanlioğlu, B. (2012). Stability and marginal bone loss with three types of early loaded implants during the first year after loading. *Int J Oral Maxillofac Implants, 27*(1), 162-172.

Lilet, R., Desiron, M., Finelle, G., Lecloux, G., Seidel, L., & Lambert, F. (2022). Immediate implant placement combining socket seal abutment and peri-implant socket filling: A prospective case series. *Clin Oral Implants Res, 33*(1), 33-44. doi:10.1111/clr.13852

Lin, C. Y., Kuo, P. Y., Chiu, M. Y., Chen, Z. Z., & Wang, H. L. (2023). Soft tissue phenotype modification impacts on peri-implant stability: a comparative cohort study. *Clinical oral investigations, 27*(3), 1089-1100. doi:10.1007/s00784-022-04697-2

Lindeboom, J. A., Tjiook, Y., & Kroon, F. H. (2006). Immediate placement of implants in periapical infected sites: a prospective randomized study in 50 patients. *Oral Surg Oral Med Oral Pathol Oral Radiol Endod, 101*(6), 705-710. doi:10.1016/j.tripleo.2005.08.022

Liu, R., Yang, Z., Tan, J., Chen, L., Liu, H., & Yang, J. (2019). Immediate implant placement for a single anterior maxillary tooth with a facial bone wall defect: A prospective clinical study with a one-year follow-up period. *Clin Implant Dent Relat Res, 21*(6), 1164-1174. doi:10.1111/cid.12854

Lops, D., Bressan, E., Parpaiola, A., Sbricoli, L., Cecchinato, D., & Romeo, E. (2015). Soft tissues stability of cad-cam and stock abutments in anterior regions: 2-year prospective multicentric cohort study. *Clin Oral Implants Res, 26*(12), 1436-1442. doi:10.1111/clr.12479

Lops, D., Romeo, E., Chiapasco, M., Procopio, R. M., & Oteri, G. (2013). Behaviour of soft tissues healing around single bone-level-implants placed immediately after tooth extraction A 1 year prospective cohort study. *Clin Oral Implants Res, 24*(11), 1206-1213. doi:10.1111/j.1600-0501.2012.02531.x

Lorenz, J., Giulini, N., Holscher, W., Schwiertz, A., Schwarz, F., & Sader, R. (2019). Prospective controlled clinical study investigating long-term clinical parameters, patient satisfaction, and microbial contamination of zirconia implants. *Clinical implant dentistry and related research, 21*(2), 263-271. doi:10.1111/cid.12720
[truncated: 51,958 more chars]
